# Targeting Mitochondrial Redox Signaling Promotes Neuroregeneration and Functional Recovery in a Multiple Sclerosis Model

**DOI:** 10.1002/advs.78560

**Published:** 2026-10-30

**Authors:** Yuki Narushima, Joshua Saylor, Shun Saito, Jinyan Zhou, Haruka Kawano, Shehata Anwar, Haruki Oba, Rachel Huezo, Miyu Okano, Haruki Matsuda, Makoto Kawatani, Kaori Honda, Hiroyuki Osada, Yuta Kikuchi, Masahito Ohue, Masami Ishibashi, Takayoshi Arai, Makoto Inoue, Midori A. Arai

**Affiliations:** ^1^ Faculty of Science and Technology Keio University Yokohama‐shi Kanagawa Japan; ^2^ Department of Comparative Biosciences University of Illinois at Urbana‐Champaign Urbana Illinois USA; ^3^ Neuroscience Program University of Illinois at Urbana‐Champaign Urbana Illinois USA; ^4^ Graduate School of Pharmaceutical Sciences Chiba University Chiba‐shi Chiba Japan; ^5^ Department of Pathology Faculty of Veterinary Medicine Beni‐Suef University (BSU) Beni‐Suef Egypt; ^6^ RIKEN CSRS Wako‐shi Saitama Japan; ^7^ Institute of Microbial Chemistry (BIKAKEN) Shinagawa‐ku Tokyo Japan; ^8^ Department of Computer Science School of Computing Institute of Science Tokyo Yokohama‐shi Kanagawa Japan; ^9^ Graduate School of Science Chiba University Chiba‐shi Chiba Japan; ^10^ Beckman Institute for Advanced Science and Technology Urbana Illinois USA

**Keywords:** CYB5R3, experimental autoimmune encephalomyelitis, neurogenesis, notch signaling inhibition, remyelination, spirooxindole

## Abstract

Neurodegenerative diseases, including multiple sclerosis (MS), are characterized by progressive neuronal loss and limited regenerative capacity. To stimulate in vivo neurogenesis and oligodendrogenesis by harnessing the untapped regenerative potential of endogenous neural progenitor cells (NPCs) that are upregulated in the CNS under disease conditions, we developed a novel compound that targets Notch signaling, a pathway known to suppress neuronal differentiation of NPCs. Furapyrrodole, which was identified by screening a natural product‐like synthetic chemical library, binds to and activates the mitochondrial reductase CYB5R3, which naturally downregulates Notch signaling downstream of γ‐secretase. The in vivo efficacy of furapyrrodole was evaluated in mice with experimental autoimmune encephalomyelitis (EAE), a mouse model of MS. Furapyrrodole treatment after the peak of EAE disease significantly improved motor function and promoted recovery of motor neurons and myelination by stimulating endogenous NPC‐derived neurogenesis and oligodendrogenesis. In addition, furapyrrodole supresses neuroinflammation in the CNS. Collectively, these findings indicate furapyrrodole is a promising therapeutic candidate for MS by harnessing endogenously upregulated NPCs and inducing anti‐inflammation. Our study demonstrates for the first time that modulation of mitochondrial redox signaling promotes neuroregeneration and remyelination without causing severe adverse effects.

## Graphical abstract

Modulation of mitochondrial redox signaling by furapyrrodole activates CYB5R3 and suppresses Notch signaling, unlocking the regenerative potential of endogenous neural progenitor cells. This approach promotes neurogenesis, oligodendrogenesis, and remyelination, thereby improving neuronal function in a mouse model of multiple sclerosis and identifying mitochondrial redox signaling as a potential therapeutic target for neuroregeneration in neurodegenerative diseases.

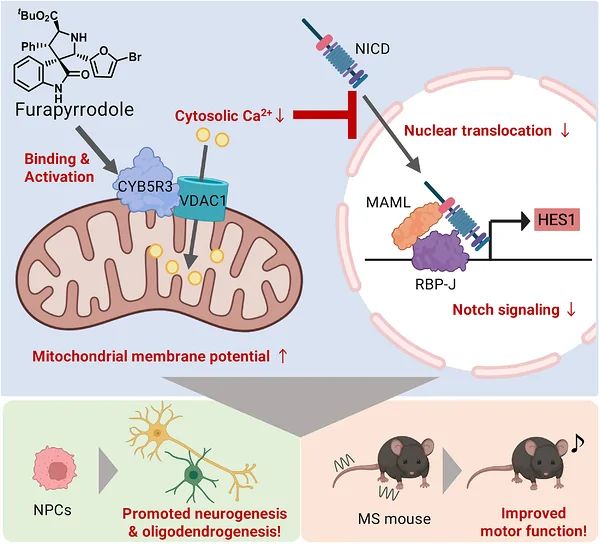

## 1 Introduction

Neurodegenerative diseases such as Huntington's disease, Alzheimer's disease, Parkinson's disease, amyotrophic lateral sclerosis, and multiple sclerosis (MS) are caused by damage to neurons. More than 3 billion people worldwide live with a neurological disorder. A primary goal of therapies aimed at enhancing neuroregeneration and remyelination in the CNS is to promote the differentiation of neural progenitor cells (NPCs) or neural stem cells (NSCs) into neurons (a process known as neurogenesis) or oligodendrocytes (referred to as oligodendrogenesis) [1]. Various strategies have been proposed for treating neurodegeneration through NPC transplantation [2, 3, 4, 5]. Increasing evidence suggests that exogenously transplanted NPCs/NSCs can improve functional recovery following central nervous system (CNS) injury [6]. However, transplantation of NPCs/NSCs faces significant challenges, including ethical concerns, tumor formation, immunogenicity, and unintended migration of implanted cells [7, 8, 9, 10, 11, 12]. NPCs/NSCs are upregulated in several neurological disorders, such as MS [13, 14, 15], spinal cord injury, and amyotrophic lateral sclerosis [16]. Despite their presence, however, these NPCs largely fail to differentiate into neurons in vivo [16, 17, 18]. Therefore, therapies that stimulate in vivo neurogenesis and oligodendrogenesis by harnessing the untapped regenerative potential of upregulated endogenous NPCs/NSCs in the CNS could hold significant promise in treating neurodegenerative diseases, as they avoid complications associated with transplantation. By promoting the natural repair mechanisms in the brain and spinal cord, these approaches could restore neural function, enhance myelin repair, and improve long‐term outcomes for patients.

The fate of NPCs, whether they self‐renew or differentiate into neurons, oligodendrocytes, and astrocytes, is primarily regulated by basic helix‐loop‐helix (bHLH) transcription factors [19]. Proneural bHLH factors, such as ASCL1/MaSH1 and NGN2 (known as neurogenesis/oligodendrogenesis‐accelerating molecules), promote neurogenesis and oligodendrogenesis. In contrast, repressor‐type bHLH factors, such as HES1, support the self‐renewal of NPCs and the generation of astrocytes. HES1 also dimerizes and binds to the promoter regions of genes encoding neurogenesis/oligodendrogenesis‐accelerating molecules, thereby suppressing their expression [20, 21]. Because Notch signaling promotes HES1 expression [22], inhibition of Notch signaling enhances neurogenesis and oligodendrogenesis by upregulating the expression of these accelerating molecules. Current data suggest that Notch1 is the primary regulator of adult neurogenesis [23]. Consequently, ongoing research is focused on discovering drugs and gene therapies that target Notch signaling [24]. However, an effective therapeutic strategy lacking significant side effects remains elusive. Among available drugs that target the Notch signaling pathway, γ‐secretase inhibitors are commonly used. However, because γ‐secretase also cleaves several essential cellular molecules, such as ephrin B2 [25], CD44 [26], N‐cadherin, E‐cadherin, and ErbB4 [27, 28, 29, 30], γ‐secretase inhibitors are associated with severe side effects. Drugs acting downstream of γ‐secretase (e.g., RBPJ and HES1) may offer safer alternatives, but none have reached the stage of therapeutic application.

Of the 1881 new drugs developed between 1981 and 2019, 46% were based on natural products (23.5%) or synthetic mimetics of natural products (22.5%) [31]. Natural product–derived drugs exhibit wide structural diversity, a characteristic that is advantageous for drug discovery [32] and may also be associated with fewer side effects because the molecules are originally expressed within living cells. We report that furapyrrodole, which was identified by screening a natural product‐like synthetic chemical library, enhances an endogenous suppressive pathway that naturally downregulates Notch signaling downstream of γ‐secretase. To elucidate the potential of furapyrrodole, we used MS models. MS is a chronic autoimmune and neurodegenerative disease of the CNS that affects over three million people globally [33, 34, 35, 36]. MS is characterized by neuroinflammation, demyelination, and neuronal degeneration/loss, with significant heterogeneity in disease presentation, progression, and treatment efficacy [34, 37]. There is an urgent need for CNS‐targeting drugs that promote neuroregeneration and remyelination and can be administered after disease progression occurs. Here, we report that furapyrrodole promotes the neuronal differentiation of NPCs both in in vitro and in a mouse model of MS, as well as its anti‐inflammatory effectswhich lead to disease recovery in mice.

## 2 Results

### 2.1 Furapyrrodole is a Potent Notch Signaling Inhibitor

Notch signaling pathway inhibits differentiation of NPCs into neurons (Figure 1A). We screened the Chiba Chemical Library for novel Notch signaling inhibitors using a previously established luciferase‐based Notch reporter assay [4, 38] (Figure 1B). In this assay, Notch signaling is transiently activated by doxycycline (Dox) treatment of cells harboring the luciferase gene downstream of 12 × RBP‐J binding sites. The level of luminescence serves as a quantitative measure of Notch signaling pathway activity. In the absence of Dox, the tetracyclin regulator (TetR) binds to the tetracyclin operator (TetO_2_) to stop the transcription of Notch1. In the presence of Dox, TetR binds Dox, causing it to dissociate from TetO_2_. This dissociation leads to Notch1 overexpression, followed by cleavage of Notch1 by γ‐secretase to generate the Notch intracellular domain (NICD). After migration to the nucleus, NICD forms a trimer with mastermind‐like protein (MAML) and recombination signal binding protein for immunoglobulin kappa J region (RBP‐J). This trimer binds to 12 × RBP‐J–binding sites, leading to luciferase expression. Using this system, we evaluated the inhibitory effect of each compound (10 µm) from the Chiba Chemical Library of synthetic compounds, which was developed by organic chemists at Chiba University. Two spirooxindole compounds, Chiba Chemical Library ID 126 and 129, were identified as novel inhibitors of the Notch pathway.

**FIGURE 1:**
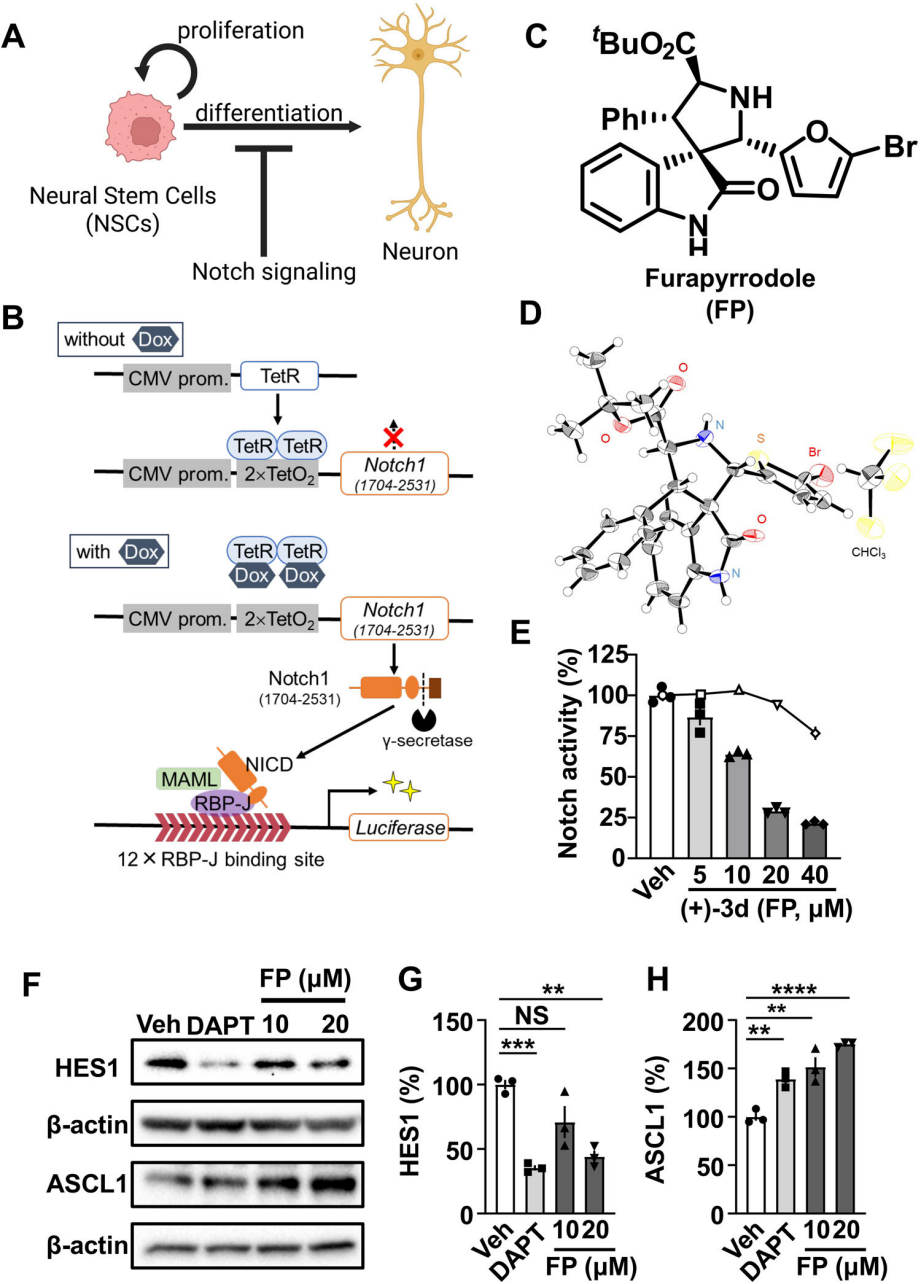
Activity of the novel Notch inhibitor furapyrrodole. (A) Relationship between Notch signaling and neuronal differentiation of NPCs. Created in BioRender. Arai, M. (2027) https://BioRender.com/i8zldnw. (B) Notch reporter assay. Without doxycycline (Dox), Notch1 (1704‐2531) expression is stopped by binding of the tetracycline repressor (TetR) to TetO_2_. Administration of Dox reverses the TetR inhibition and allows the expression of Notch1 (1704‐2531). γ‐Secretase cuts Notch1 (1704‐2531) to produce the Notch intracellular domain (NICD), which forms a trimer with mastermind‐like protein (MAML) and recombination signal binding protein for immunoglobulin kappa J region (RBP‐J), leading to the expression of luciferase. TetO_2_: tetracycline operator; CMV prom.: human cytomegalovirus promoter. (C) Structure of furapyrrodole. (D) x‐ray structural analysis of **3c**. The R‐value was 9.17%, Crystal Color, Habit: colorless, platelet, Crystal Dimensions: 0.10 × 0.05 × 0.01 mm, Space Group: P‐1 (#2). (E) Evaluation of Notch inhibition by (+)‐**3d** (furapyrrodole: FP) obtained by optical resolution. LS174T reporter cells were seeded in a 96‐well plate (2.0 × 10^4^ cells per well) and treated with doxycycline (50 ng/mL) for 12 h to induce Notch1 (1704–2531) expression, followed by incubation with (+)‐**3d** for 12 h. Luciferase activity was then measured to evaluate Notch signaling inhibition. Cell viability was assessed in parallel by fluorescein diacetate staining under the same treatment conditions (*n* = 3). (F–H) Western blotting of HES1 and ASCL1 in C17.2 cells treated with vehicle (Veh), DAPT (10 µM), or FP (10 or 20 µm) for 12 h at 37°C (*n* = 3). The same samples were used to nalyze HES1 in Figure 1F and RBP‐J in Figure S10 simultaneously; therefore, the beta‐actin loading‐control data are identical. All data are shown as mean ± SEM. Statistical significance was determined by one‐way ANOVA followed by Tukey's post hoc test; ^*^ *p* < 0.05, ^**^ *p* < 0.01, ^***^ *p* < 0.001, ^****^ *p* < 0.0001. NS indicates not significant.

Spirooxindole compounds 126 and 129 are synthesized by [3+2] cycloaddition of methyleneindolinone and iminoester via the Michael‐Mannich tandem reaction (Figures S1 and S2). Spirooxindoles can be stereoselectively synthesized with 99% enantiomeric excess (ee) by asymmetric synthesis using the imidazoline‐aminophenol (IAP)‐nickel catalyst [39]. Although 16 diastereomers were possible, the IAP‐nickel catalyst primarily generated conformer A (2’*R*,3*R*,4’*S*,5’*S*). This spirooxindole core structure has been identified in several natural products, such as spirotryprostatin A and B [40] and strychnofoline [41] (Figure S3). Therefore, these spirooxindole compounds are natural product‐like small molecules.

To enhance the interaction with a target protein, a heterocyclic moiety was incorporated into these spiro compounds. The inhibitory activity was enhanced by synthesizing derivatives in which the aromatic ring moiety was replaced with various heterocyclic rings and then evaluating the Notch inhibitory activity (Figures S4–S8). As a result, we identified compound **3d** with a bromofuran ring as the most active Notch inhibitor (Table 1, Figure 1C). The relative conformations of the derivative **3c** was confirmed by x‐ray crystallographic analysis (Figure 1D). Hydrolysis of **3d** with trifluoroacetic acid to remove the *t*‐butyl group resulted in **4d**, which did not inhibit Notch (Figure S8). This lack of inhibitory activity may be attributable to an inability to penetrate the cell membrane due to an increase in hydrophilicity after transformation to a carboxylic acid. We also evaluated the Notch signaling inhibitory activity of derivative **3d'**, in which the conformation of R^2^ was reversed [42], but the compound showed weak inhibition, with a 50% inhibitory concentration (IC_50_) of 37.7 µm (Figure S8). This suggests that the conformation of the R^2^ site plays an important role in Notch signaling inhibitory activity.

**TABLE 1:** Synthesis of Spirooxindoles and Evaluation of Notch Inhibition.

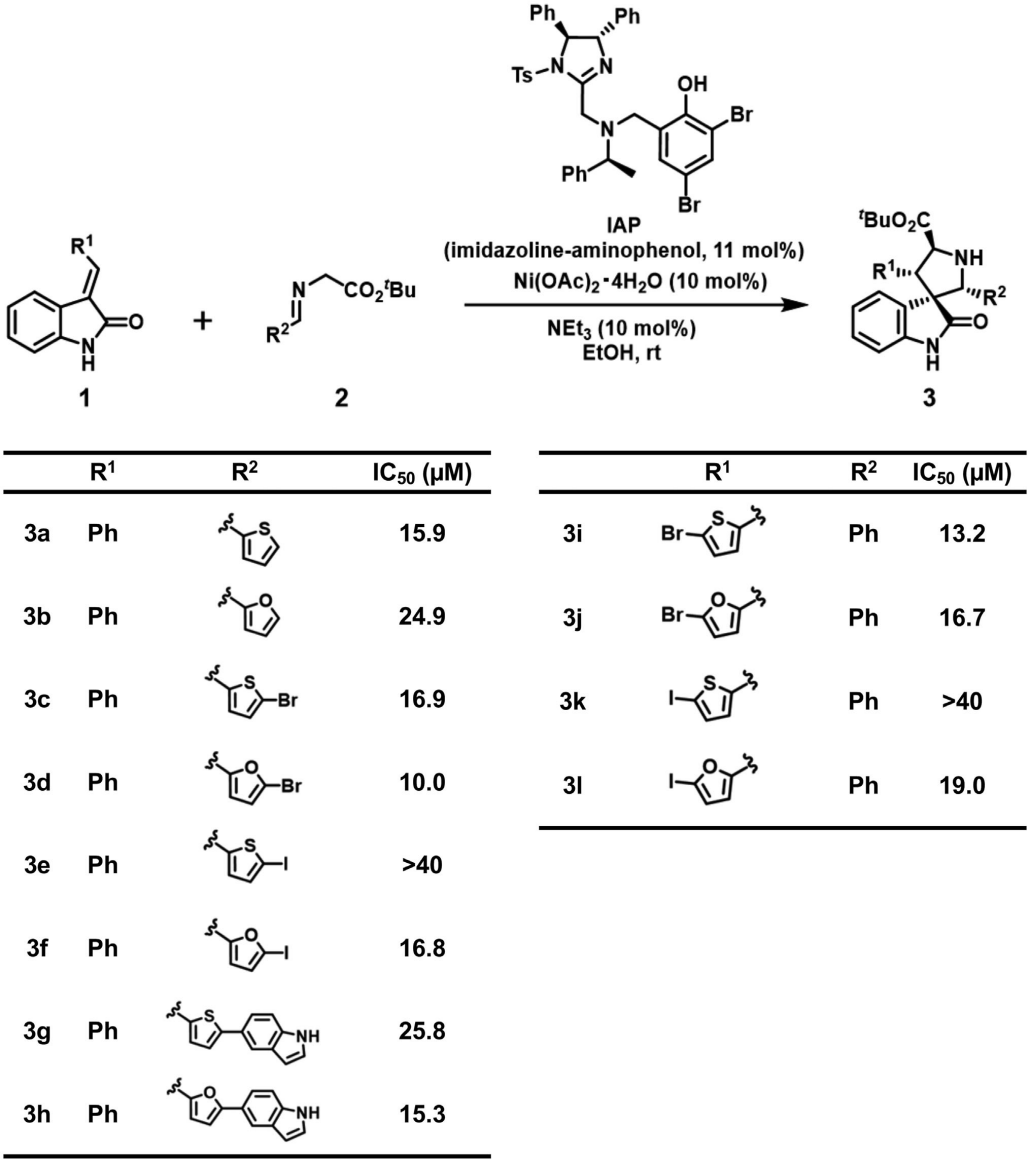

The most active compound, **3d**, was also subjected to optical resolution and inhibitory activity analysis. The IC_50_ of (+)‐**3d** (termed furapyrrodole) was 13.1 µm (Figure 1E), superior to the 24.4 µm IC_50_ of (−)‐**3d** (Figure S9). To confirm the inhibitory effect of furapyrrodole on Notch signaling, we examined the expression level of HES1, which is upregulated by Notch signaling [43], and ASCL1, which is inhibited by HES1. Treatment of C17.2 pluripotent mouse NPCs with furapyrrodole for 12 h resulted in a significant decrease in HES1 expression (Figure 1F,G), whereas ASCL1 expression was upregulated (Figure 1F,H). These results suggest that furapyrrodole is a potent functional inhibitor of Notch signaling.

### 2.2 Furapyrrodole Targets CYB5R3, which Functions Downstream of γ‐Secretase

The mechanism of Notch signaling inhibition was elucidated by determining the targeting signals of furapyrrodole. Because existing major Notch inhibitors, such as [*N*‐(3, 5‐difluorophenacetyl)‐L‐alanyl]‐*S*‐phenylglycine *t*‐butyl ester (DAPT) [44], target γ‐secretase, we first evaluated whether furapyrrodole acts upstream or downstream of γ‐secretase in Notch signaling pathway. Analysis of the expression of NICD, the cleaved intracellular domain of Notch1, revealed that furapyrrodole did not affect NICD levels (Figure 2A,B). Further analysis confirmed that furapyrrodole did not affect expression levels of the transcription factors MAML and RBP‐J, which form a complex with NICD in the nucleus to mediate Notch signaling (Figure S10). These results suggest that furapyrrodole targets Notch signaling downstream of γ‐secretase but does not alter transcription factor expression levels.

**FIGURE 2:**
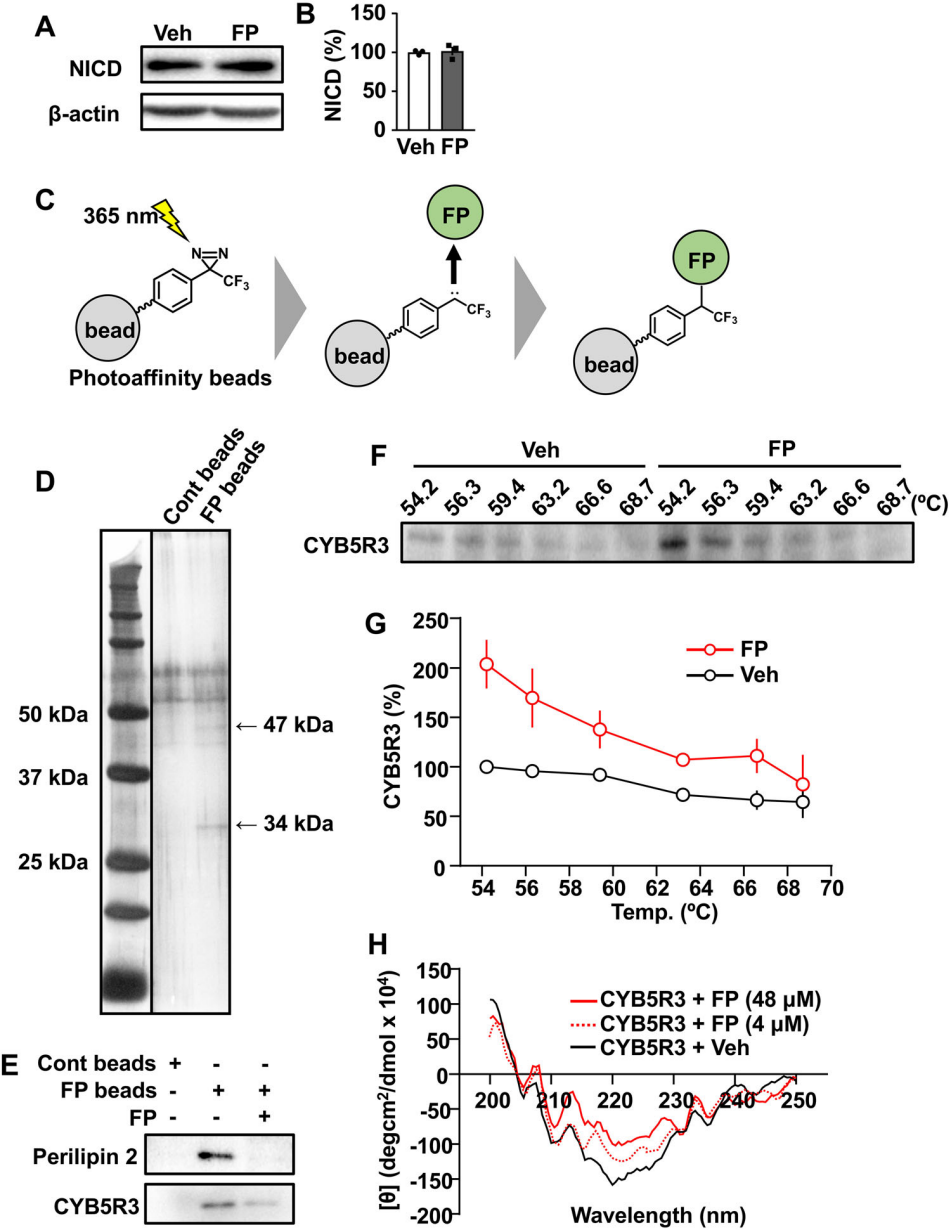
Target protein determination. (A, B) Western blotting of NICD in C17.2 cells treated with furapyrrodole (FP, 10 µm) or vehicle (Veh) (*n* = 3). C17.2 cells were treated with FP for 12 h at 37°C. (C) Preparation of FP‐conjugated beads. Photoaffinity linker–coated agarose beads and FP were mixed in a glass vial and dried in vacuo. The beads were then irradiated with 365‐nm UV light to facilitate covalent coupling with FP. (D) Pull‐down assay. FP‐conjugated beads or control beads were incubated with C17.2 cell lysate for 6 h at 4°C. After centrifugation and washing, bound proteins were eluted, separated using SDS‐PAGE, and visualized by silver staining. Representative results are shown (*n* = 3). (E) Competition assay. FP (1 µmol) was added to C17.2 cell lysate and incubated with rotation at 4°C for 4 h, after which control or FP‐conjugated beads were added and incubated for an additional 6 h. A pull‐down assay was subsequently performed under the same conditions. CYB5R3 and perilipin 2 bound to the beads were quantified by Western blotting. Representative results are shown (*n* = 3).(F, G) Cellular thermal shift assay CYB5R3 treated with FP (*n* = 3). C17.2 cell lysate was treated with FP or Veh for 2 h at room temperature and then thermally denatured by incubation for 3 min at 54.2°C, 56.3°C, 59.4°C, 63.2°C, 66.6°C, and 68.7°C. The level of CYB5R3 was measured by Western blotting. (H) Circular dichroism (CD) spectrum of treatment with CYB5R3 and FP (4 or 48 µm) or Veh. Representative results are shown (*n* = 3). Recombinant mouse CYB5R3 was expressed in *E. coli*. Purified protein in PBS was mixed with FP at the indicated final concentrations by sequential addition, followed by gentle inversion mixing and analysis. All data are shown as mean ± SD. Statistical significance was determined using one‐way ANOVA followed by Tukey's post hoc test; ^*^ *p* < 0.05.

To identify the actual targets of furapyrrodole in C17.2 cells, a pull‐down assay was performed to isolate the direct binding protein. Furapyrrodole‐immobilized beads were prepared by photoaffinity coupling (Figure 2C). Upon UV irradiation at 365 nm, diazirine‐functionalized beads generate reactive carbene intermediates that covalently bind to various positions on furapyrrodole, allowing immobilization of furapyrrodole in diverse orientations [45]. Both furapyrrodole‐immobilized and control beads were incubated with C17.2 cell lysate for 5 h at 4°C to capture potential binding proteins. After centrifugation, proteins bound to the beads were eluted, separated using SDS‐PAGE, and visualized by silver staining. Distinct protein bands at approximately 30 and 50 kDa were specifically enriched in furapyrrodole‐immobilized bead samples (Figure 2D). These proteins were identified using liquid chromatography–tandem mass spectrometry (LC‐MS/MS) as nicotinamide adenine dinucleotide (NADH)–cytochrome b5 reductase 3 (CYB5R3) and perilipin 2, respectively (Table S1). In a competitive binding assay with free furapyrrodole, binding of CYB5R3 and perilipin 2 to the furapyrrodole‐immobilized beads was markedly reduced (Figure 2E). Because the exponentially modified protein abundance index value (indicating protein amount) of CYB5R3 (1.88) was higher than that of perilipin 2 (0.68) (Table S1), we focused on CYB5R3 to further characterize the interaction between furapyrrodole and CYB5R3 as well as the role of CYB5R3 in the function of furapyrrodole. Cellular thermal shift assays were conducted to confirm a direct interaction between furapyrrodole and CYB5R3. Lysates of C17.2 cells were treated with furapyrrodole for 2 h at room temperature and then subjected to thermal denaturation. Subsequent Western blot analysis revealed that furapyrrodole increased the thermal stability of CYB5R3. CYB5R3 present in C17.2 cell lysate treated with furapyrrodole was heat‐tolerant (54.2°C, 3 min) (Figure 2F,G). Compared with the control, 203.8% of protein was retained. These results suggest that direct ligand binding occurs between furapyrrodole and CYB5R3.

Next, we expressed and then purified recombinant mouse CYB5R3 in *E. coli* (Figure S11). The dissociation constant (*K*_d_) of furapyrrodole was determined using circular dichroism spectroscopy. Compared to glutathione *S*‐transferase as a control (Figure S12), furapyrrodole treatment induced a spectral shift (Figure 2H), with a binding affinity (*K*_d_) of 3.8 µM (Figure S13). To gain structural insights, we used AlphaFold3 to generate a predicted structure of the complex consisting of mouse CYB5R3, flavin adenine dinucleotide as a cofactor [46], and NADH [47]. This was followed by docking simulations with furapyrrodole. The resulting model suggested that furapyrrodole binds near the cofactor NADH‐binding pocket [48], forming hydrogen bonds with Arg160 and Lys295 (Figure S13). These results suggest that the binding of furapyrrodole at this site stabilizes the structure of CYB5R3, thereby affecting its activity.

### 2.3 Furapyrrodole Activates CYB5R3 and Modulates Mitochondrial Redox Signaling, Leading to Notch Signaling Inhibition via Ca^2+^‐Dependent Nuclear Translocation of NICD

CYB5R3 is a flavin‐dependent reductase located in the mitochondrial outer membrane [49], but its relationship to Notch signaling has not been previously reported. We therefore investigated the functional relationship between furapyrrodole, CYB5R3, and Notch signaling. To assess the role of CYB5R3 in Notch signaling, we performed knockdown experiments using a CYB5R3‐specific siRNA in C17.2 cells. Knockdown of CYB5R3 in C17.2 cells significantly increased HES1 expression (Figure 3A–C), suggesting that CYB5R3 negatively regulates Notch signaling, which in turn increases HES1 expression. These results suggest that furapyrrodole interacts with CYB5R3 and enhances its activity to suppress Notch signaling. To evaluate this possibility, CYB5R3 enzymatic activity was analyzed in C17.2 cell lysates. Because CYB5R3 is known to reduce cytochrome c in an NADH‐dependent manner [50], cytochrome c levels were measured using spectrophotometry to evaluate CYB5R3 activity by quantifying the characteristic absorption peak at 550 nm specific to reduced cytochrome c (Figure 3D). Mixing cytochrome c with NADPH, C17.2 cell lysate, and furapyrrodole for 5 min resulted in an upregulation of the absorption peak, compared with vehicle mixing (Figure 3E), suggesting that furapyrrodole enhances CYB5R3 enzymatic activity.

FIGURE 3Analysis of the activity mechanism. (A–C) Western blotting of HES1 in CYB5R3‐knockdown C17.2 cells. C17.2 cells were seeded in a 6‐well plate and incubated for 12 h at 37°C. The cells were then transfected with CYB5R3 siRNA (CYB5R3 KD) or control siRNA (Cont) using Lipofectamine 2000 for 24 h at 37°C. Representative results are shown (*n* = 3). (D) Overview of the CYB5R3 enzymatic assay. CYB5R3 reduces cytochrome c in an NADPH‐dependent manner, and the reduction is quantified by measuring its characteristic absorption peak. (E) Evaluation of CYB5R3 enzymatic activity in C17.2 cell lysate by measuring a characteristic 550‐nm absorbance peak of reduced cytochrome c (*n* = 3). Cytochrome solution, NADPH solution, C17.2 cell lysate, and furapyrrodole (FP) or vehicle (Veh) were mixed. After incubation for 5 min at room temperature, the components were quantified spectrophotometrically. (F) Measurement of mitochondrial membrane potential using JC‐1. When the mitochondrial membrane potential increases, JC‐1 forms polymers (J‐aggregates) and emits red fluorescence. Created in BioRender. Arai, M. (2027) https://BioRender.com/i8zldnw. (G) The fluorescence image of JC‐1 after treatment with FP. Scale bar = 200 µm. (H) Measurement of mitochondrial membrane potential (*n* = 3). CYB5R3 KD or cont. C17.2 cells were treated with FP (0, 5, 10, 20 µm) for 1 h at 37°C. JC‐1 fluorescent dye was then added and incubated for 1 h at 37°C. Mitochondrial membrane potential was quantified by calculating the fluorescence ratio of JC‐1 aggregates (emission: 595 nm, excitation: 535 nm) to monomers (emission: 535 nm, excitation: 485 nm). (I, J) Detection of Ca^2+^ incorporation into mitochondria. Mito‐GCaMP5G construct was introduced transiently to C17.2 cells. The change in fluorescence over time (0–270 s) was recorded and quantified. Representative results are shown (*n* = 3). The time‐course data (30–270 s) of Δfluorescence intensity (vs 0 s) are shown. Scale bar = 50 µm. (K, L) Ca^2+^ levels were measured in C17.2 cells treated with thapsigargin (TG) or FP immediately after compound addition using Fura‐2 AM. The change in fluorescence over time (0–40 s) was recorded and quantified by subtracting the value for the DMSO control. Representative results are shown (*n* = 3). The bar graph represents the fluorescence intensity at 10 s from the time‐course data (0–40 s) (*n* = 3). (M, N) Level of nuclear NICD in C17.2 cells treated with Veh, FK506 (10 µm), or FP (10 or 20 µm). Nuclear fractionation was performed 12 h after compound treatment at 37°C. The nuclear protein fraction was collected and analyzed by Western blotting. Representative results are shown (*n* = 3). (O) The rescue of Notch reporter activity by co‐treatment under the TG treatment. Assay cells (LS174T Notch cells) were treated with FP (20 µm) with or without TG (10 nm). (P) The rescue of FP (20 µm) inhibition of NICD nuclear translocation under TG (1 µm) treatment. (Q) Mode of action of Notch inhibition by FP. Created in BioRender. Arai, M. (2027) https://BioRender.com/i8zldnw. All data are shown as mean ± SEM. For panels B, C, E, and G, statistical significance was determined using Student's *t*‐test. For panel (K), statistical significance was determined using one‐way ANOVA followed by Tukey's post hoc test. ^*^ *p* < 0.05, ^**^ *p* < 0.01, ^***^ *p* < 0.001. NS indicates not significant.

**Figure:**
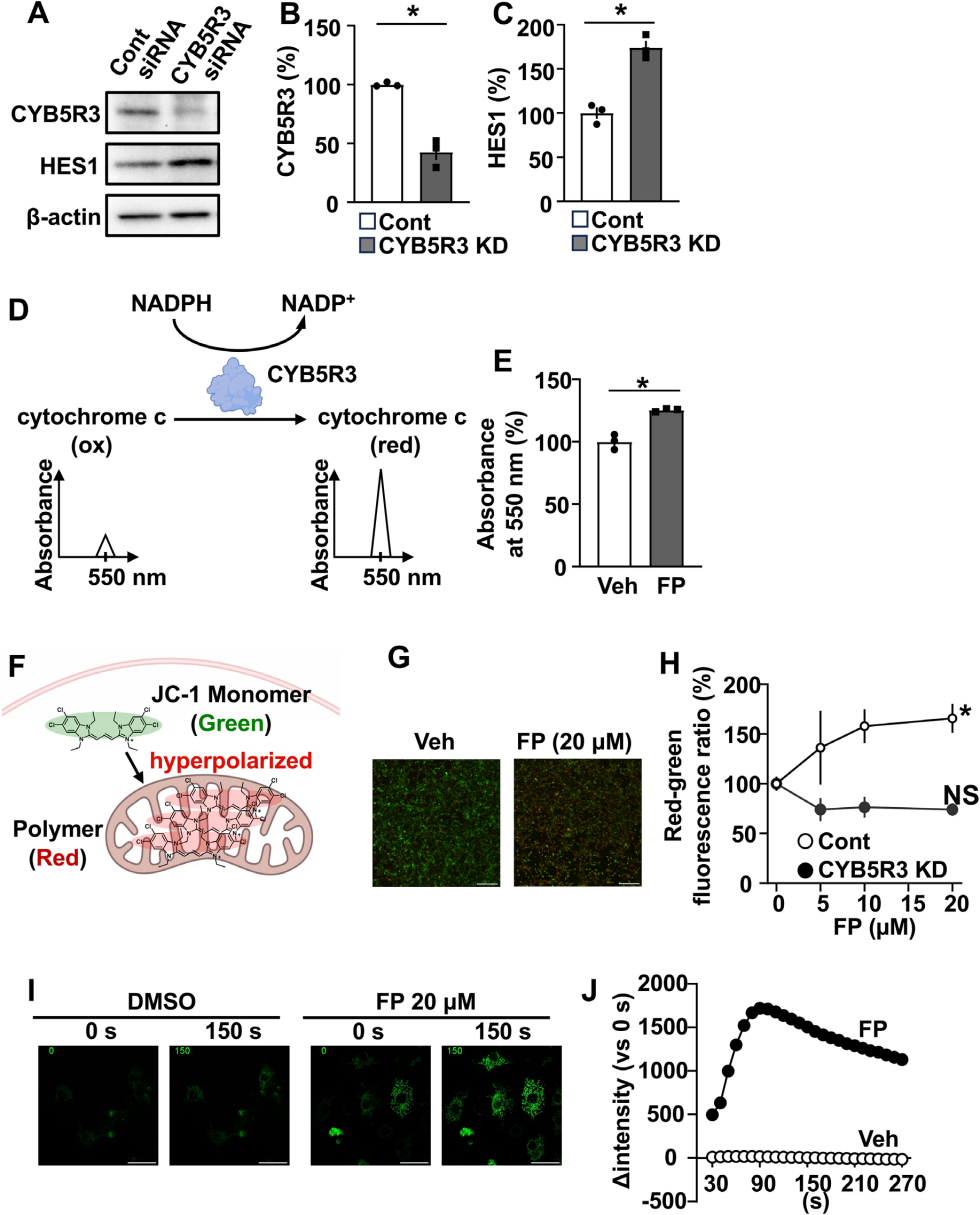

**Figure:**
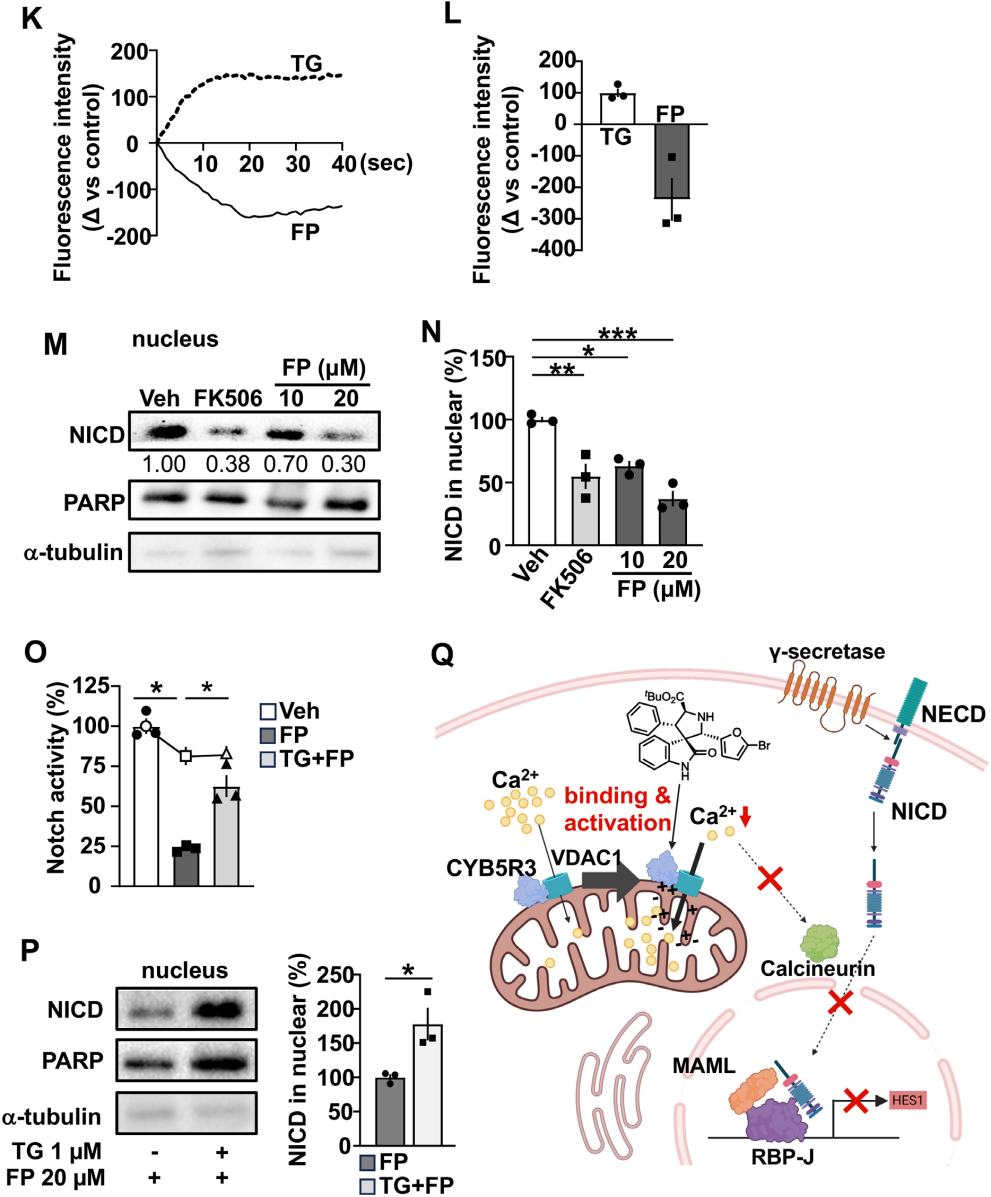

Because CYB5R3 is also known to mediate electron transfer into mitochondria [51], we hypothesized that furapyrrodole increases mitochondrial membrane potential by activating CYB5R3. To test this hypothesis, we used the fluorescent dye JC‐1, which exhibits green fluorescence in the monomeric form but shifts to red fluorescence upon aggregation in response to high mitochondrial membrane potential [52] (Figure 3F). Thus, mitochondrial membrane potential was quantitatively assessed by measuring the red‐to‐green fluorescence ratio. C17.2 cells were treated with furapyrrodole for 1 h, followed by JC‐1 staining and fluorescence measurement. The increased red fluorescence by furapyrrodole was detected by a confocal fluorescence microscope (Figure 3G). Further, a dose‐dependent increase in mitochondrial membrane potential (red‐to‐green fluorescence ratio) was observed following furapyrrodole treatment (Figure 3H). By contrast, furapyrrodole did not increase the mitochondrial membrane potential in CYB5R3‐knockdown cells (Figure 3H), confirming that the increase in membrane potential induced by furapyrrodole treatment is CYB5R3‐dependent. These results indicate that furapyrrodole alters the mitochondrial redox signaling by CYB5R3 activation.

Next, we focused on the voltage‐dependent anion channel 1 (VDAC1), which is located on the outer mitochondrial membrane, and this channel regulates Ca^2+^ concentration in mitochondria. An increase in mitochondrial membrane potential promotes the closure of VDAC1 [53]. The closed conformation of VDAC1 favors cation influx, thus potentially enhancing the uptake of Ca^2+^ into the mitochondrial matrix from the cytosol [52]. A reduction in cytosolic Ca^2+^ levels prevents the activation of calcineurin, which is required for the nuclear translocation of NICD [54]. Based on these observations, we hypothesized that furapyrrodole inhibits NICD nuclear translocation by reducing cytosolic Ca^2+^ levels through an increase in mitochondrial membrane potential. To test this hypothesis, the construct of Mito‐GCaMP5G [55], which codes for a mitochondria‐targeted calcium indicator, was introduced transiently to C17.2 cells to detect mitochondrial Ca^2+^ uptake. After the addition of furapyrrodole, the fluorescence, indicating Ca^2+^, was increased in a time‐dependent manner, which indicated that Ca^2+^ was incorporated into mitochondria by the addition of FP (Figure 3I,J and Movie S1). These results indicate that furapyrrodole accelerates the incorporation of Ca^2+^ into mitochondria. Next, we measured intracellular Ca^2+^ levels in C17.2 cell suspensions using the Ca^2+^‐sensitive fluorescent probe Fura‐2. Thapsigargin, a sarco‐endoplasmic reticulum calcium ATPase inhibitor, was used as a positive control, preventing Ca^2+^ uptake into the endoplasmic reticulum. The cytosolic Ca^2+^ concentration was increased by thapsigargin treatment but, as we expected, significantly reduced by furapyrrodole (Figure 3K,L). We then measured NICD levels in the nuclear fractions of C17.2 cells treated for 12 h with furapyrrodole and FK506, a calcineurin inhibitor [56], as a positive control. Furapyrrodole significantly reduced the nuclear NICD level to a similar extent as FK506 (Figure 3M,N). We further examined whether cytosolic Ca^2+^ upregulation suppresses furapyrrodole function. We conducted a luciferase‐based Notch reporter assay (Figure 1B) and a NICD nuclear translocation assay and found that furapyrrodole‐mediated Notch inhibition (Figure 3O) and NICD nuclear translocation (Figure 3P) were significantly suppressed under thapsigargin treatment in C17.2 cells. This indicates that Notch inhibition by furapyrrodole is related to cytosolic Ca^2+^ concentration.

Taken together, these results indicate that furapyrrodole interacts with and activates CYB5R3, leading to an increase in mitochondrial membrane potential and a concomitant reduction in cytosolic Ca^2+^ levels. The reduction in cytosolic Ca^2+^ inhibits calcineurin‐dependent nuclear translocation of NICD, thereby suppressing Notch signaling (Figure 3Q). This is the first report that modulation of mitochondrial redox signaling leads to Notch signaling inhibition.

### 2.4 Furapyrrodole Promotes the Differentiation of NPCs into Neurons and Oligodendrocytes

Because furapyrrodole is a potent Notch signaling inhibitor and increases the expression of ASCL1, we next investigated the function of furapyrrodole in NPC differentiation. C17.2 cells were treated for 12 h with vehicle (1% DMSO), DAPT (a representative Notch inhibitor), or furapyrrodole. The cells were then stained for Tuj1, a neuron‐specific marker, and GFAP, an astrocyte‐specific marker, with specific secondary antibodies, and fluorescence signals were detected by confocal microscopy. Quantification of the ratio of neurons to the number of nuclei revealed that furapyrrodole increased the number of neurons in a concentration‐dependent manner (Figure 4A). The proportion of neurons differentiated with 10 µm furapyrrodole was 60.8%, representing a 3.32‐fold increase compared with the vehicle‐treated group (Figure 4B). Furapyrrodole‐induced neuronal differentiation was stronger than that in the DAPT‐treated group (Figure 4A,B). By contrast, no astrocytes were detected at this stage (Figure 4A). To confirm that these effects were mediated by activation of CYB5R3, the same experiments were performed in CYB5R3‐knockdown C17.2 cells. As a result, the effects of furapyrrodole were markedly attenuated in C17.2 cells with CYB5R3 knockdown (Figure 4C,D). This finding indicates that CYB5R3 is a functional target of furapyrrodole in promoting neurogenesis.

**FIGURE 4:**
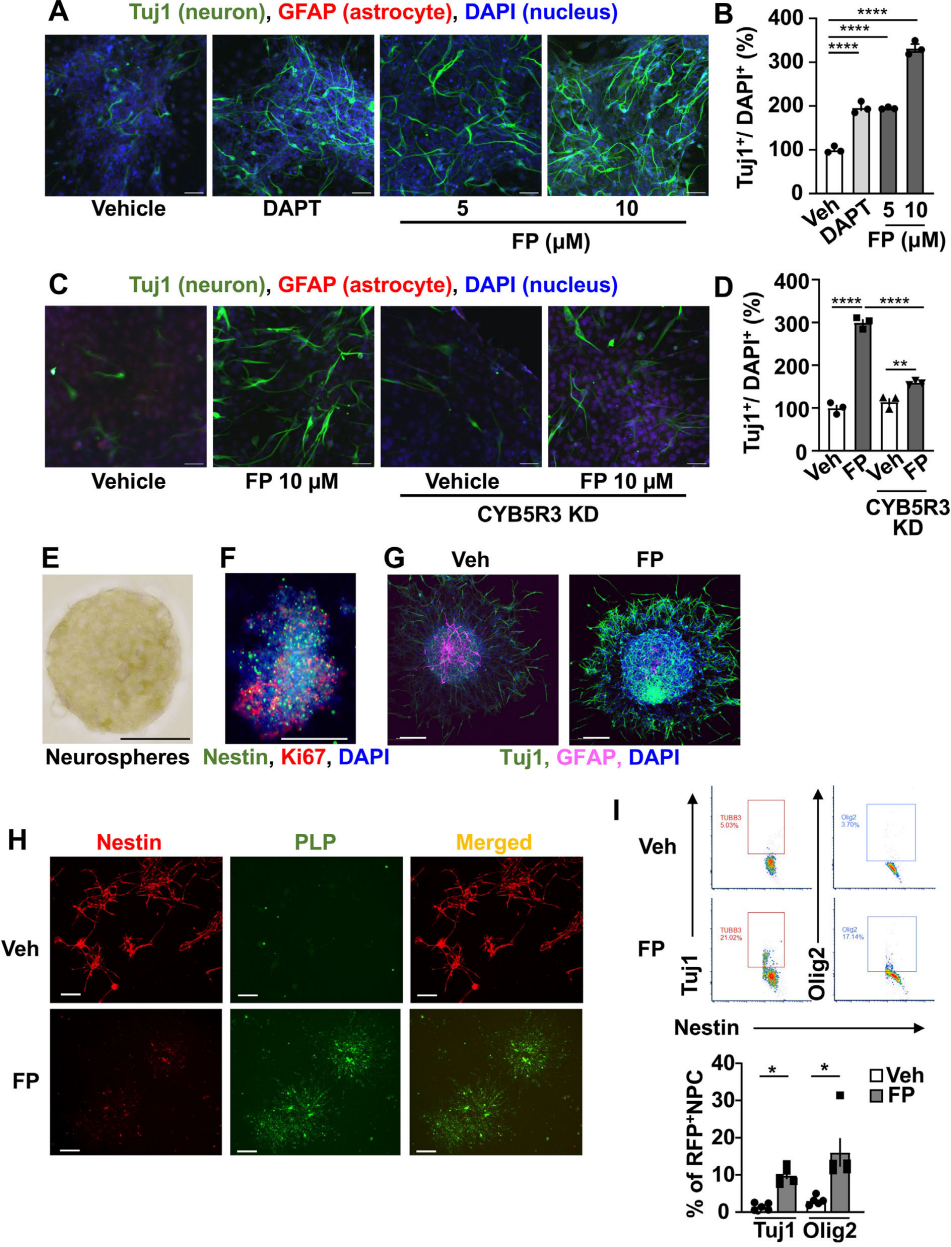
Induction of NPC differentiation by furapyrrodole. (A, B) Fluorescence immunostaining of C17.2 cells after treatment with Vehicle (Veh), DAPT (10 µm), or furapyrrodole (FP, 5 or 10 µm) for 4 days. Neurons were immunostained with Tuj1 (green). Astrocytes were immunostained with GFAP (red). Nuclei were immunostained with DAPI (blue). Scale bars = 50 µm. (C, D) Fluorescence immunostaining of CYB5R3 knockdown C17.2 cells after treatment with vehicle (Veh) or FP 10 µm for 4 days (*n* = 3). Scale bars = 50 µm. Data were evaluated using one‐way ANOVA followed by Tukey's multiple comparison test. ^**^ *p* < 0.01, ^***^ *p* < 0.001. (E, F) NPC spheres (E) were stained for Nestin (green), Ki67 (red), and DAPI (blue) (F). Scale bar = 50 µm. (G, H) Efficacy of treatment with FP (10 µm) for 4 days in sphered NPCs (G) or for 7 days in dissociated NSCs (H). NPCs/NSCs were stained for Tuj1 (green), GFAP (purple), and DAPI (blue) (G) or Nestin (red) and PLP (green) (H). Scale bars = 50 µm (G) and 20 µmm (H). (I) Flow cytometry analysis of NPC differentiation after treatment with FP (10 µm) for 4 days (*n* = 5). Student's *t*‐test. ^*^ *p* < 0.05.

We further investigated furapyrrodole‐induced neuronal differentiation of primary NPCs isolated from EAE mice. NPCs were isolated from the spinal cord of EAE mice at 20 days post–EAE induction (dpi) (peak stage of disease, Figure 5) using the Percoll gradient method [58]. NPCs were cultured in Neurobasal‐A medium supplemented with growth factors. After neurospheres (Figure 4E) were formed, they were harvested, dissociated, and re‐cultured for 7 days. The presence of an NPC marker (nestin) and proliferation marker (Ki67) was assessed using immunocytochemistry (Figure 4F). When furapyrrodole was added to sphere‐forming NPCs in Neurobasal‐A medium without growth factors, the population of neurons (Tuj1) increased dramatically compared with vehicle treatment (Figure 4G). In the vehicle‐treated group, NPCs exhibited strong fluorescence and retained high nestin expression (Figure 4H). In contrast, furapyrrodole treatment reduced nestin expression while increasing the expression of proteolipid protein (PLP), a marker of mature oligodendrocytes (Figure 4H), suggesting that furapyrrodole promotes oligodendrogenesis. Conversely, furapyrrodole suppressed astrocytic differentiation, as indicated by reduced GFAP expression, compared with vehicle treatment (Figure 4G). To quantify these effects, we performed flow cytometric analysis. Consistent with the immunofluorescence findings, furapyrrodole significantly increased the proportions of neurons and oligodendrocytes differentiated from NPCs compared with vehicle treatment (Figure 4I). Mitochondrial metabolic reprogramming, such as an oxidative phosphorylation (OXPHOS) shift, is known to accelerate neuron differentiation [57]. To address this point, we measured the oxygen consumption rate [59] in C17.2 cells upon furapyrrodole treatment and found that furapyrrodole did not increase OCR, suggesting that OXPHOS would not shift in response to furapyrrodole (Figure S14). Therefore, the acceleration of the mitochondrial metabolic pathway is not involved in the furapyrrodole‐mediated NPC differentiation. In summary, furapyrrodole is a strong candiate as a promoter of neurogenesis and oligodendrogenesis.

**FIGURE 5:**
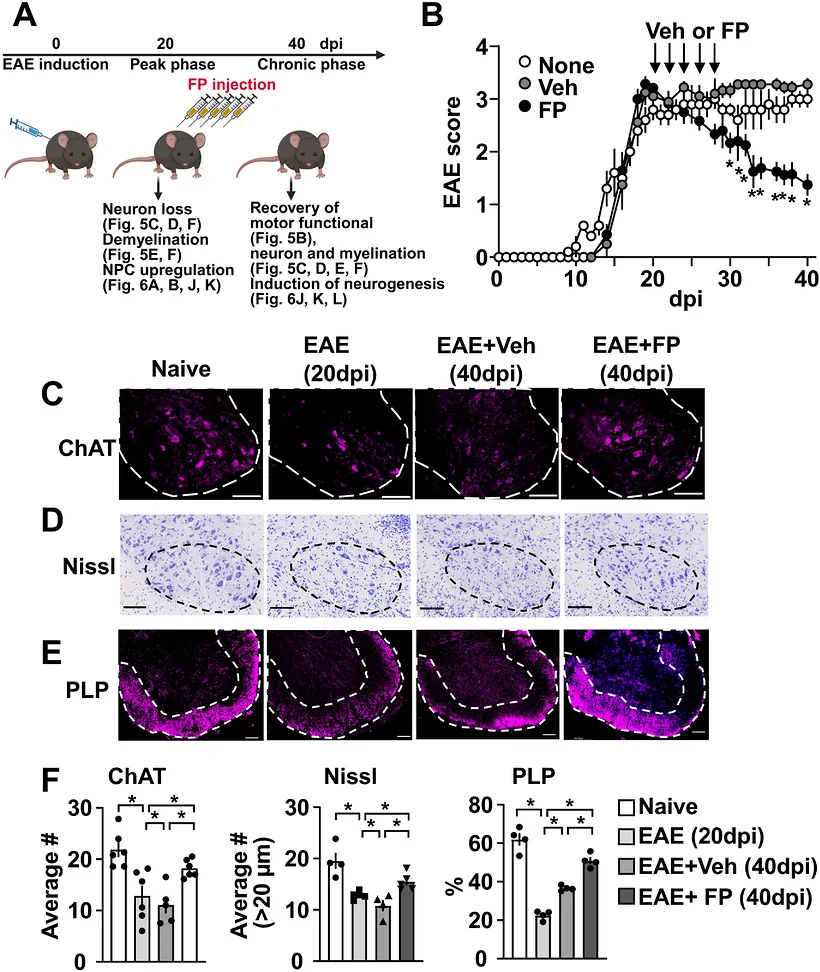
Promotion of recovery by furapyrrodole in EAE disease. (A) Drug treatment paradigm. Created in BioRender. Inoue, M (2026), https://BioRender.com/5drl62n. (B) EAE behavior scores of mice treated with vehicle (Veh) or furapyrrodole (FP, 1 µg/5 µL, intrathecal injection, every other day from 20 to 28 dpi) (*n* = 10). (C–F) Status of motor neurons (ChAT^+^) (C, F) and Nissl‐stained neurons (>20 µm as motor neurons) (D, F) in the ventral horn (dotted lines) and myelination (proteolipid protein [PLP^+^]) (E, F) in the ventral side (within the dotted lines) of the lumbar spinal cord of naïve mice, EAE mice at 20 dpi, and EAE mice with vehicle or FP at 40 dpi. Bars = 100 µm (*n* = 4‐6). Data indicate the average number of ChAT^+^ cells in the image and the percentage of PLP‐stained area (24 slice images/mouse were evaluated). Nissl‐stained neurons >20 µm with nuclei (e.g., motor neurons) were counted in every 10th section of the lumbar spinal cord to calculate the average number of neurons. Conditions were the same as those for data shown in (C) and (D) (*n* = 4–6). Data were evaluated using one‐way ANOVA followed by Tukey's multiple comparison test.

### 2.5 Furapyrrodole Significantly Improves Motor Function and Restores Motor Neurons and Myelination in EAE Mice

EAE mice show severe neuronal loss and demyelination in the CNS [60, 61]. EAE was induced in C57BL6 mice using myelin oligodendrocyte glycoprotein (MOG)_35‐55_ peptide, according to a previous report [60, 61]. EAE symptoms, measured on the standard neurological scale (0 = asymptomatic, 5 = death) [62], began approximately 10 dpi, peaked at 20 dpi, and persisted without remission until at least 40 dpi (Figure 5B, None). At 20 dpi, EAE mice showed a significant reduction in the number of motor neurons, as indicated by choline acetyltransferase (ChAT) immunostaining in the ventral horn of the spinal cord, compared with naïve mice (Figure 5C,F). To confirm actual motor neuron loss, stereological Nissl‐stained neuron counting was conducted in the entire lumbar spinal cord. A similar decrease was observed in the number of Nissl‐stained neurons [63] with a cell body diameter exceeding 20 µm, which is indicative of motor neurons [64, 65] (Figure 5D,F). EAE mice at 20 dpi also exhibited severe demyelination in the ventral spinal cord, as shown by proteolipid protein (PLP) staining (Figure 5E,F). These results suggest that EAE mice lose motor neurons in the ventral horn, and demyelination in the ventral side of the spinal cord occurs at 20 dpi (peak stage of disease).

To determine whether furapyrrodole improves motor function by promoting the restoration of motor neurons and myelin, mice were administered furapyrrodole (1 µg/5 µL per mouse) intrathecally after the occurrence of peak disease (20 dpi) and continuously treated every other day until 28 dpi (Figure 5A). This treatment significantly improved motor function compared to vehicle‐treated mice (Figure 5B). At 40 dpi, furapyrrodole‐treated mice showed a marked increase in motor neurons (Figure 5C,D, and F) and improved myelination by evaluating PLP expression (Figure 5E,F) and Luxol Fast Blue staining (Figure S15), compared to both vehicle‐treated mice and/or the pre‐treatment condition (at 20 dpi). Because the loss of neurons at 20 dpi is considered irreversible in EAE mice, the recovery of motor function following furapyrrodole treatment indicates that the compound promotes NPC‐derived neurogenesis and oligodendrogenesis.

### 2.6 Furapyrrodole Induces NPC Differentiation under the EAE Condition

To demonstrate furapyrrodole‐induced neurogenesis and oligodendrogenesis in vivo, we assessed NPC status in naïve mice and EAE mice with and without furapyrrodole treatment. The expression levels of nestin, an NPC marker, were dramatically upregulated in the central canal of the spinal cord in EAE mice at 20 dpi, compared with naïve mice (Figure 6A,B). The expression of CYB5R3, which is a binding partner of furapyrrodole and an essential mediator of its activity (Figures 2 and 3), was also upregulated in the central canal of EAE mice relative to naïve mice (Figure 6A,C). Notably, CYB5R3 is highly localized to nestin^+^ NPCs (Figure 6D), and CYB5R3 levels within NPCs were also significantly higher in EAE mice than in naïve mice (Figure 6E). These results suggest that upregulated CYB5R3 in NPCs may be involved in the furapyrrodole‐induced neurogenesis and oligodendrogenesis.

**FIGURE 6:**
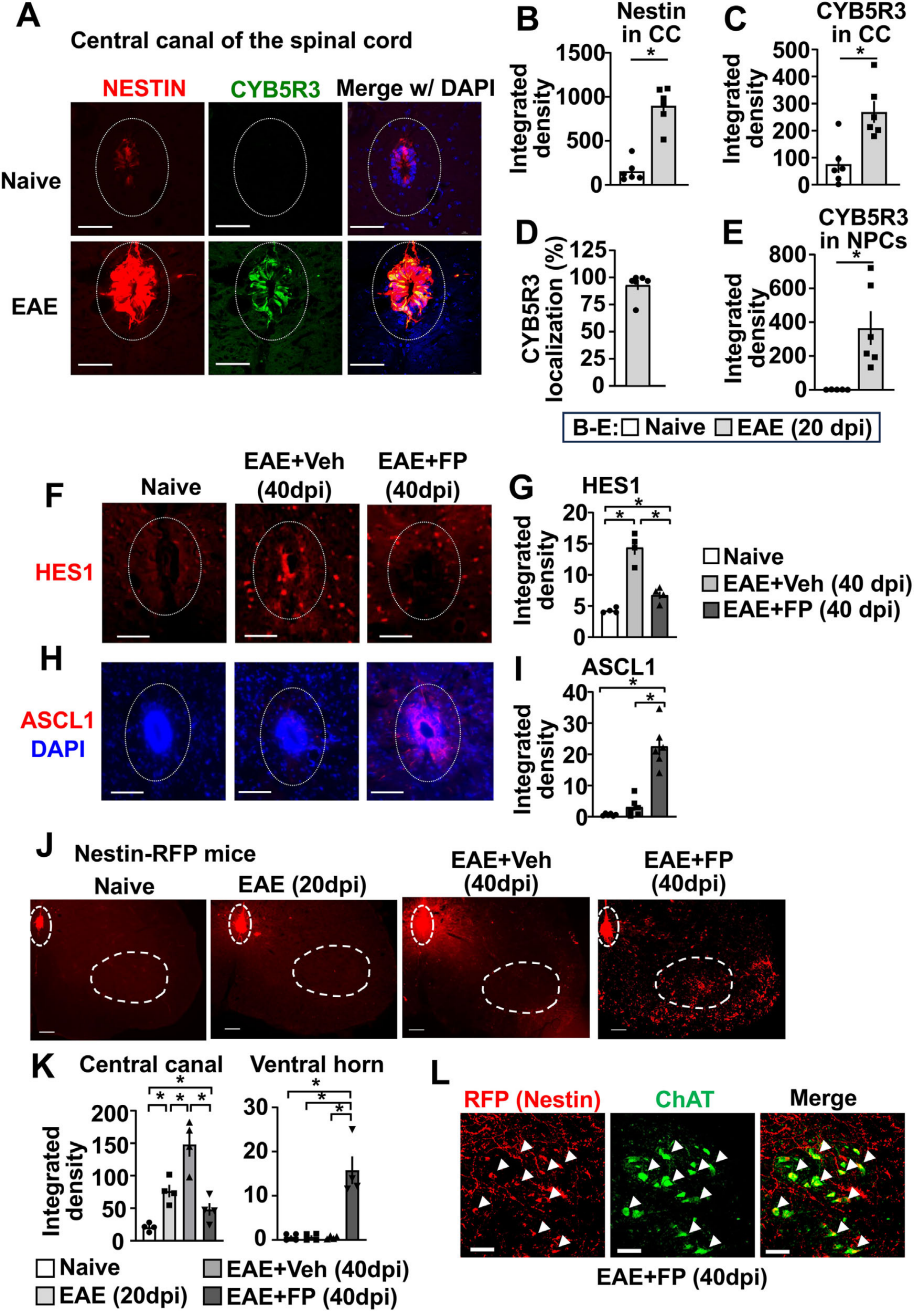
Furapyrrodole induces neurogenesis and oligodendrogenesis. (A–E) Representative images and expression levels of Nestin (A, B) and CYB5R3 (A, C–E) around the central canal of the spinal cord in naïve and EAE at 20 dpi. Expression levels of Nestin (B) and CYB5R3 (C) in the central canal (CC). (D) Frequency of CYB5R3's NPC localization measured by the ratio of Nestin signal to the CYB5R3 signal. Expression levels of CYB5R3 in NPCs (Nestin^+^) in EAE mice (E). (F–I) Representative images and expression levels of HES1 (F, G) and ASCL1 (H, I) around the central canal of the spinal cord in naïve (white columns) and EAE mice treated with vehicle (Veh) or furapyrrodole (FP, 1 µg/5 µL, every other day from 20 to 28 dpi) at 40 dpi. (J) Red fluorescent protein (RFP) signals in naïve Nestin‐RFP mice, EAE Nestin‐RFP mice at 20 dpi, and EAE Nestin‐RFP mice treated with Veh or FP at 40 dpi. Nestin‐RFP mice were orally administered tamoxifen for 5 consecutive days from the EAE induction date (0 to 4 dpi). Naïve mice were also treated with tamoxifen without EAE induction. (K) Quantification of RFP signals in the central canal and ventral horn (dotted lines). (L) RFP (Nestin) and ChAT co‐staining in the ventral horn of the spinal cord in EAE mice treated with FP at 40 dpi. Quantification in the central canal and ventral horn (dotted lines) was performed using 4‐6 mice (at least 10 sections/mouse). Bars = 100 µm. *p* < 0.05, determined using one‐way ANOVA followed by Tukey's multiple comparison test.

We also evaluated the role of furapyrrodole in the expression of HES1 and ASCL1. Similar to the increase in nestin (Figure 6A), HES1 expression was also significantly upregulated in EAE mice (Figure 6F,G). By contrast, ASCL1 was expressed at low levels in naïve mice and remained unchanged (Figure 6H,I). These results suggest that although NPCs are upregulated in EAE, their differentiation into neurons and oligodendrocytes may be impaired due to persistently high HES1 expression and low ASCL1 expression. Furapyrrodole intrathecal treatment from 20 to 28 dpi (same as in the behavior study illustrated in Figure 5A) significantly reduced HES1 expression and markedly increased ASCL1 expression (Figure 6F–I). This finding suggests that furapyrrodole may drive the differentiation of disease‐upregulated NPCs.

To confirm that furapyrrodole promotes neurogenesis and oligodendrogenesis in the spinal cord of EAE mice, lineage‐tracing studies were conducted using Nestin–red fluorescent protein (RFP) mice, which were generated by mating NestinCre/ERT2 and RFP flox mice. Because RFP expression in Nestin‐RFP mice is controlled by tamoxifen‐inducible Cre expression, these mice were orally administered tamoxifen (3 mg/0.1 mL in corn oil) for 5 consecutive days [61], starting from the date of EAE induction (0 to 4 dpi). This approach allowed labeling of NPCs with an RFP signal after EAE induction, as RFP remains stable once its expression is activated. Similar to nestin immunostaining (Figure 6A), RFP signals were primarily localized around the central canal and significantly upregulated at 20 dpi compared with tamoxifen‐treated naïve mice (Figure 6J,K). By 40 dpi, RFP signals were further upregulated in the central canal (Figure 6J,K). By contrast, EAE mice that received intrathecal furapyrrodole treatment from 20 to 28 dpi exhibited a distinct RFP distribution at 40 dpi. RFP signals were significantly reduced around the central canal and increased outside of the central canal, including the ventral horn (Figure 6J,K). Some RFP signals were co‐localized with the ChAT signal in the ventral horn in furapyrrodole‐treated EAE mice (Figure 6L). This result supports the findings above, demonstrating that furapyrrodole promotes neurogenesis and oligodendrogenesis.

### 2.7 Systemic Injection of Furapyrrodole Recovers EAE Disease by Promoting Neurogenesis and Oligodendrogenesis

We also tested the efficacy of furapyrrodole in EAE mice following systemic injection. First, we used LC‐MS to determine whether furapyrrodole injected intraperitoneally reaches the spinal cord. A detection method for furapyrrodole was developed using an Agilent 1290 Infinity II UHPLC and Sciex 6500+ triple quadrupole mass spectrometer, and we successfully generated a standard curve that was linear from 0.05 to 100 ng/mL (R^2^ = 0.998) (Figure S16A,B). We removed the spinal cord from mice at 15, 30, and 60 min after intraperitoneal (i.p.) injection of furapyrrodole (1 mg/kg), which is an effective dose in EAE (Figure 7B). Furapyrrodole was detected in the spinal cord in a time‐dependent manner from 15 to 60 min (Figure 7A). These results suggest that furapyrrodole penetrates the spinal cord.

**FIGURE 7:**
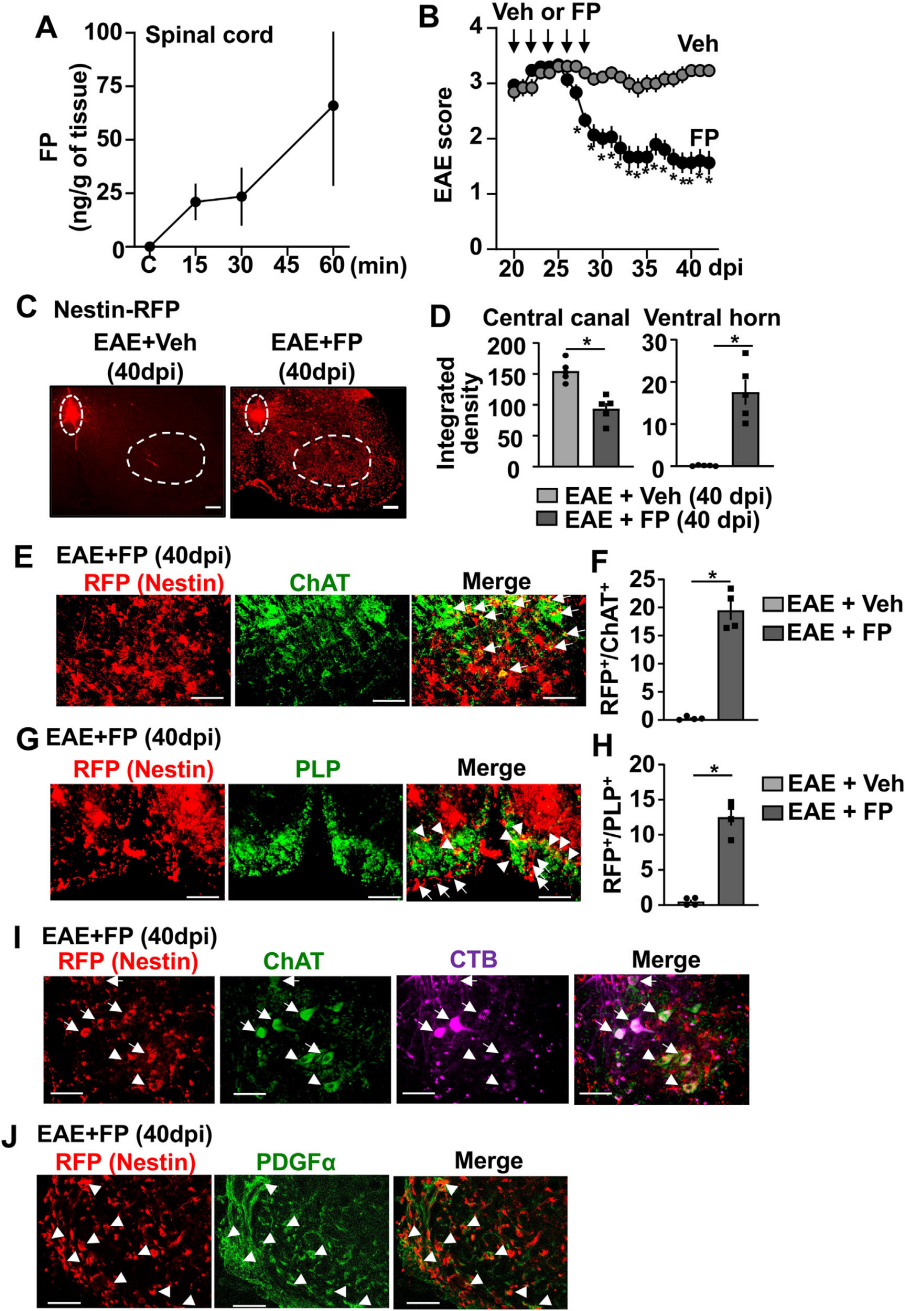
Effect of systemic injection of furapyrrodole on EAE and NPC differentiation. (A) LC‐MS analysis of FP in the lumbar spinal cord at 15, 30, and 60 min after i.p. injection (*n* = 3). (B) EAE behavior scores following i.p. injection of vehicle (Veh) or furapyrrodole (FP, 1 mg/kg, every other day from 20 to 28 dpi) (*n* = 8). (C, D) RFP signals in EAE Nestin‐RFP mice with Veh or FP treatment at 40 dpi. Quantification in the central canal and ventral horn in 5 mice (at least 10 sections/mouse). (E, F) ChAT and RFP (Nestin) signals in the ventral horn of the spinal cord in EAE mice with FP (E, F) or vehicle (F) treatment at 40 dpi. Arrows indicate colocalization of ChAT and RFP. Quantifications of the RFP signals in ChAT^+^ cells in the ventral horn of the spinal cord were performed using 4 mice (at least 10 sections/mouse). (G, H) PLP and RFP signals in the ventral side of the spinal cord in EAE mice with FP treatment at 40 dpi. Arrows indicate colocalization of PLP and RFP. Arrowheads indicate RFP in demyelination regions (PLP^−^ white matter). Quantifications of the RFP signals in PLP^+^ cells in the ventral horn of the spinal cord were performed using 4 mice (at least 10 sections/mouse). (I) ChAT, RFP, and cholera toxin subunit B (CTB) retrograde dye signals in the ventral horn of the spinal cord in EAE mice with FP treatment at 40 dpi. Arrows indicate colocalization of ChAT, RFP, and CTB. Arrowheads indicate colocalization of ChAT and RFP. (J) PDGFα and RFP signals in the ventral side of the spinal cord in EAE mice with FP treatment at 40 dpi. Arrowheads indicate co‐localization. ^*^ *p* < 0.05, determined using Student's *t*‐test.

Injection of furapyrrodole (1 mg/kg; i.p.) after the peak stage of disease (20 dpi), which is characterized by severe neuron loss and demyelination in the spinal cord (Figure 5), resulted in significant recovery from EAE symptoms, accompanied by improved motor function (Figure 7B). Additionally, systemic furapyrrodole treatment resulted in notable distribution of RFP in the spinal ventral horn in Nestin‐RFP mice (Figure 7E), similar to mice given furapyrrodole via intrathecal injection (Figure 5). Importantly, some RFP^+^ NPCs in the spinal ventral horn colocalized with ChAT staining in EAE mice that were treated with furapyrrodole, but not vehicle (Figure 7E and S17). The ratio of colocalization (RFP^+^ in ChAT^+^ cells) in the ventral horn was significantly higher than in vehicle‐treated EAE mice (Figure 7F). In addition, some RFP signals in the ventral white matter of the spinal cord colocalized with PLP staining in FP‐treated EAE mice (Figure 7G and Figure S17). The ratio of colocalization (RFP^+^ in PLP^+^ cells) in the ventral white matter was also significantly higher than in vehicle‐treated mice (Figure 7H). These results suggest that furapyrrodole facilitates the differentiation of NPCs into motor neurons and oligodendrocytes. Further, we examined whether NPC‐derived motor neurons in the ventral horn of the spinal cord innervate skeletal muscle. For this purpose, Alexa 647–conjugated cholera toxin subunit B, a retrograde neuron tracer widely used for motor neuron mapping [66], was injected into the hind limb muscle [67] of EAE Nestin‐RFP mice treated with furapyrrodole (1 mg/kg; i.p., 20–28 dpi, Figure 7B) at 40 dpi. 7 days after Alexa 647 CTB dye injection, the spinal cords were isolated, and the Alexa 647 signals were assessed with RFP and ChAT signals within the ventral horn. Alexa 647 signals co‐localized with RFP^+^ChAT^+^ NPC‐derived motor neurons (Figure 7I), suggesting that some NPC‐derived motor neurons innervate skeletal muscle. It should be noted that Alexa 647 dye injected into limited regions of skeletal muscle does not label all motor neurons in the spinal cord [66]. Further, because some RFP^+^ cells were located within demyelinated (PLP^−^) regions of the white matter (Figure 7G), we stained PDGFα, an OPC marker, in the ventral spinal cord and found that some RFP^+^ cells are colocalized with PDGFα (Figure 7J). These results suggest that some NPC‐derived cells are still in the process of maturing into oligodendrocytes capable of generating myelin.

### 2.8 Furapyrrodole Suppresses Neuroinflammation in the Spinal Cord

Systemic and intrathecal administration of furapyrrodole produced sustained suppression of EAE, even after cessation of treatment (Figures 5 and 7). These findings suggest that furapyrrodole not only promotes neuroregeneration and oligodendrogenesis but also suppresses prolonged neuroinflammation within the CNS following immune cell infiltration, a key driver of MS progression and neurodegeneration [68, 69]. To investigate this possibility, we evaluated the effects of furapyrrodole on spinal cord inflammation in EAE mice. Intrathecal furapyrrodole treatment (20–28 dpi) significantly reduced the levels of multiple proinflammatory mediators, including chitinase 3‐like 1, C‐reactive protein, ICAM‐1, lipocalin‐2, MOP, and pentraxin 3, compared with vehicle‐treated mice at 40 dpi (Figure 8A,B). These inflammatory mediators have been implicated in the pathogenesis of MS [70, 71, 72, 73, 74, 75, 76, 77, 78] and EAE [79, 80, 81] and are produced predominantly by activated microglia and infiltrating macrophages during neuroinflammation [74, 82, 83, 84, 85, 86, 87, 88, 89, 90]. Consistent with these findings, intrathecal furapyrrodole treatment (1 µg/5 µl, 20–28 dpi) significantly reduced the number of Iba1^+^ microglia/macrophages in the ventral horn of the spinal cord (Figure 8C). Likewise, systemic administration of furapyrrodole (1 mg/kg, i.p., 20–28 dpi) also decreased the number of microglia in the spinal cord (Figure 8D). Because activated microglia and infiltrating macrophages promote CD4^+^ T‐cell activation and differentiation in the CNS and contribute to neuronal damage and tissue atrophy in progressive MS and EAE [68, 69, 91], we next examined T‐cell number in the spinal cord. Furapyrrodole significantly reduced the numbers of CD4^+^ T cells and Th17 cells in the spinal cord at 40 dpi compared with vehicle‐treated mice (Figure 8E). In contrast, systemic furapyrrodole treatment did not alter CD4^+^ T cell or Th17 cell populations in the spleen (Figure S18), suggesting that its anti‐inflammatory effects are primarily mediated within the CNS rather than through systemic immunosuppression. In sum, furapyrrodole also has anti‐neuroinflammatory effects in the CNS.

**FIGURE 8:**
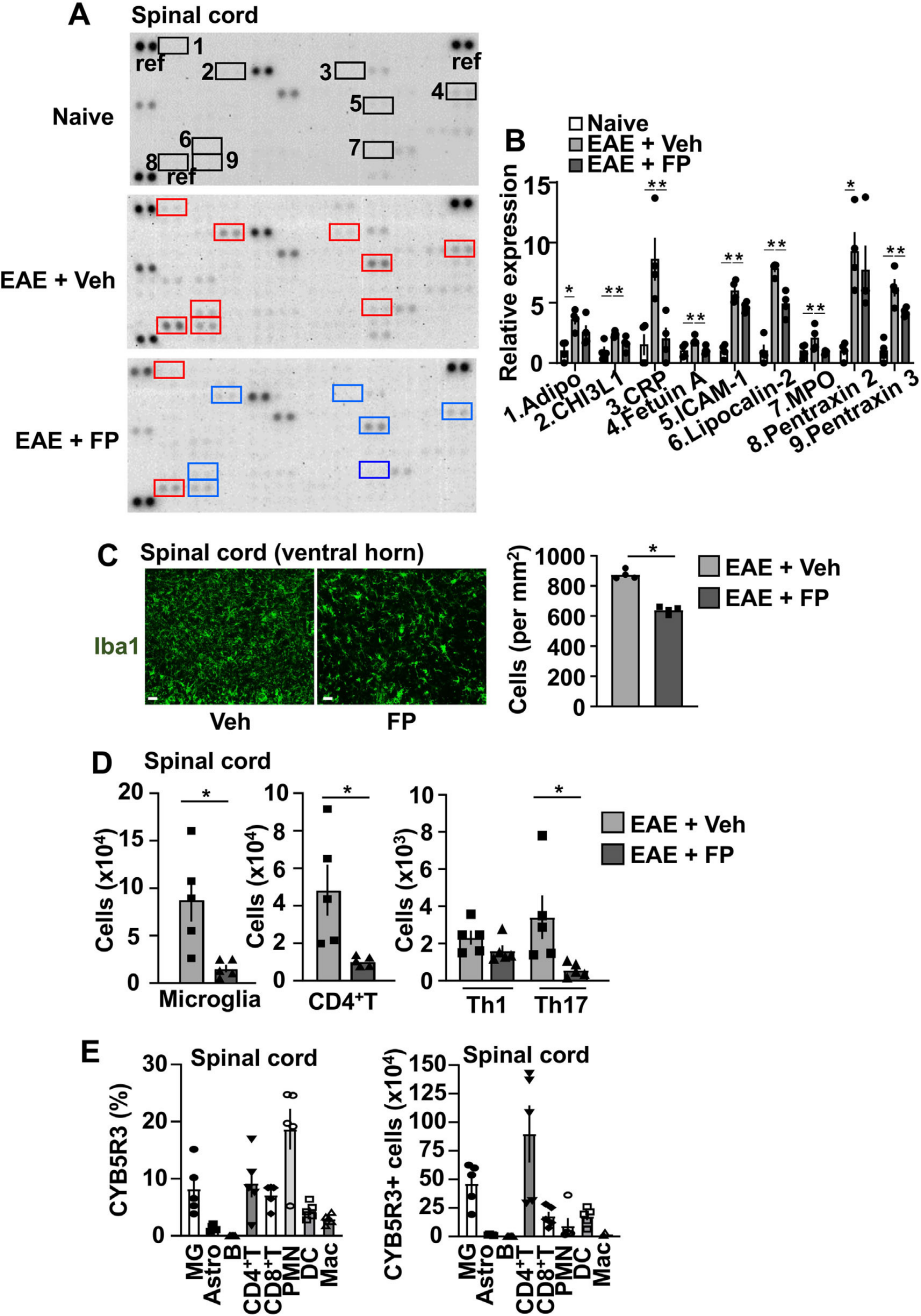
Anti‐inflammatory effect of furapyrrodole. (A, B) Representative images of cytokine array (A) and results of quantification assays (B) of cytokine expression in the spinal cord (*n* = 4). The lumbar spinal cord of naïve mice and EAE mice with vehicle (Veh) or furapyrrodole (FP, 1 µg, every other day from 20 to 28 dpi) at 40 dpi. Data were normalized according to three reference (ref) signals, and then the data in naive mice. (C) Representative images and results of quantification assays of Iba1 staining (microglia and infiltrating macrophages) in the spinal cord of EAE mice treated with Veh or FP at 40 dpi (*n* = 4). (D) Flow cytometry analysis of microglia (CD45^low^, CD11b^+^), CD4^+^ T cells (CD45^+^, CD3^+^, CD4^+^), Th1 cells (CD45^+^, CD3^+^, CD4^+^, IFNγ^+^), and Th17 cells (CD45^+^, CD3^+^, CD4^+^, IL‐17^+^) in the spinal cord of EAE mice treated with Veh or FP at 40 dpi. (E) Flow cytometry analysis of CYB5R3 expression (frequency and cell number) in glia and immune cells in the spinal cord. The gating strategy is shown in Figure S19. ^*^ *p* < 0.05, determined using one‐way ANOVA followed by Tukey's multiple comparison test (B) and Student's *t*‐test (C, D).

Because furapyrrodole directly binds to CYB5R3, we next evaluated CYB5R3 expression in glia and immune cells in the spinal cord under EAE conditions by flow cytometry. High levels of CYB5R3 expression were detected in microglia, polymorphonuclear leukocytes, CD4^+^ T cells, CD8^+^ T cells, and macrophages in the spinal cord of EAE mice (Figure 8E and Figure S19). Among these populations, CYB5R3^+^ microglia and CYB5R3^+^ CD4^+^ T cells were the most abundant (Figure 8E). Interestingly, peripheral immune cells, including CD4^+^ T cells,  in the spleen of EAE mice exhibited relatively low CYB5R3 expression (Figure S19), suggesting that CYB5R3 is upregulated after infiltrating immune cells into the CNS during EAE disease. Together, these findings suggest that the anti‐inflammatory effects of furapyrrodole may be mediated through its interaction with CYB5R3 in CNS‐resident and infiltrating immune cells, particularly microglia and CD4^+^ T cells.

### 2.9 Furapyrrodole is Associated with Fewer Adverse Effects

A common problem with Notch inhibitors is the occurrence of severe adverse effects. For example, through Notch‐mediated differentiation of intestinal stem cells, γ‐secretase inhibitors such as DAPT and dibenzazepine trigger various gastrointestinal side effects, including metaplasia and hyperplasia of secretory goblet cells [92]. Gastrointestinal side effects are a concern during clinical trials, and patients are carefully monitored for these issues [93]. We therefore used AB‐PAS staining to examine whether systemic injection of furapyrrodole triggers hyperplasia of goblet cells (Figure S20A) in comparison with DAPT. Mice were treated with furapyrrodole at 1 mg/kg (i.p.), which is effective in ameliorating EAE symptoms (Figure 7B). Although a previous study used 10 mg/kg of DAPT [92], we treated mice with DAPT at 1 mg/kg to match the dosing of furapyrrodole. Vehicle, furapyrrodole, and DAPT were administered intraperitoneally to groups of EAE mice every other day from 20 to 28 dpi using the same injection paradigm (Figure 7B), and the intestines were collected at 30 dpi. Even though we used a lower dose of DAPT than in the previous report [92], DAPT‐treated mice showed an increased number of goblet cells in intestinal crypts compared with the vehicle‐treated group (Figure S20B). By contrast, there was no difference in goblet cell number between the furapyrrodole‐treated and vehicle‐treated groups. CYB5R3, which is significantly upregulated in NPCs (Figure 6A,C), was relatively rare in the intestine (Figure S20C). In addition, CYB5R3 is also relatively rare in colocalizing with intestinal stem cells (Figure S20D,E). These results suggest that furapyrrodole does not induce adverse gastrointestinal effects, even when administered systemically, potentially due to the minimal expression of CYB5R3 in intestinal stem cells.

## 3 Discussion

The results of our study demonstrate that spirooxindole compounds exhibit potent Notch‐inhibitory activity and thus represent a promising scaffold for the development of regenerative therapeutics targeting the CNS. Notably, the spirooxindole framework is frequently found in natural products, and our findings underscore the substantial benefits of leveraging natural product–inspired scaffolds for drug discovery and therapeutic development. We successfully synthesized a series of heterocyclic spirooxindole derivatives and identified furapyrrodole as a lead compound. Nevertheless, the current chemical space remains underexplored, and ample opportunity exists for structural diversification through the introduction of alternative substituents, which may further enhance pharmacological profiles.

In this study, we found that furapyrrodole directly binds to CYB5R3. To our knowledge, no other small‐molecule ligands that directly bind to CYB5R3 have been reported, and thus, its pharmacological modulation remains largely uncharacterized. Our solid studies demonstrate that furapyrrodole acts as a CYB5R3 activator, inducing hyperpolarization of the mitochondrial membrane potential, which may, in turn, alter the conductance state of VDAC1 and reduce cytosolic Ca^2+^ levels. This reduction in Ca^2+^ inhibits calcineurin‐dependent nuclear translocation of the NICD, thereby suppressing Notch signaling. This is a novel regulatory mechanism of Notch signaling. Importantly, endogenous CYB5R3 functions as a regulatory modulator of Notch1 signaling, as evidenced by the upregulation of HES1 following CYB5R3 knockdown. Unlike γ‐secretase inhibitors, which directly block Notch signaling, furapyrrodole enhances an endogenous suppressive pathway that naturally downregulates Notch1 signaling. This mechanism enables more precise modulation of Notch1 activity, thereby achieving improved target specificity and minimizing potential off‐target effects. In this way, furapyrrodole fine‐tunes physiological Notch signaling rather than completely shutting it down. These results indicate that modulating the mitochondria redox balance can accelerate differentiation of NPCs/NSCs and restore neuronal function in neurodegenerative diseases. Further, importantly, we found that CYB5R3 is preferentially expressed and upregulated in disease‐activated spinal NPCs, but not in intestinal stem cells (Figure 6 and Figure S20). In addition to its fine‐tune regulation of Notch signaling, this selective expression pattern may contribute to the favorable therapeutic profile of furapyrrodole, including its lower likelihood of adverse effects, such as gastrointestinal toxicity (Figure S20).

The efficacy of currently approved therapeutic agents for MS, including interferon‐β [94], glatiramer acetate [95], and natalizumab [96], varies considerably among patients. Notably, few effective treatments are available for severe forms of the disease, such as primary progressive MS [97], which is characterized by pronounced neurodegeneration [98]. Moreover, existing disease‐modifying therapies are often associated with an increased risk of infections and do not adequately target immune cell function within the CNS. In the present study, we aimed to harness endogenously upregulated NPCs in the CNS. Several studies have reported the presence of NPCs in the brain and spinal cord of various animal models of neurodegenerative diseases [99, 100] as well as in postmortem tissues of patients [93, 101]. Despite the presence of NPCs in the adult spinal cord, they are likely not related to the repair of damaged neurons or oligodendrocytes [102]. Non‐invasive approaches that promote neural repair and reduce neuroinflammation could prove to be safe and effective treatment options for MS and other neurodegenerative diseases. This strategy has been proposed for promoting motor recovery following ischemia‐related damage [103] and for the repair of cochlear hair cells following acoustic trauma [104].

In the present study, we observed significant therapeutic effects of furapyrrodole administered via direct CNS delivery (intrathecal route). Given that CNS delivery has been successfully employed in clinical settings for CNS drug delivery [105, 106, 107], such approaches may also be applicable to the treatment of MS. For enhanced convenience and improved patient compliance, systemic administration is preferable. Furapyrrodole penetrates the spinal cord following systemic injection, enhances NPC differentiation into neurons and oligodendrocytes, and induces clinical recovery in the EAE model. Importantly, furapyrrodole‐produced NPC‐derived motor neurons exhibited the capacity to extend projections toward skeletal muscle, suggesting their potential to contribute to motor circuitry. Functional validation of these newly generated motor neurons will be an important future project. We observed that CYB5R3 is upregulated in NPCs under EAE conditions. Accordingly, it is likely that furapyrrodole promotes neurogenesis and oligodendrogenesis in vivo through CYB5R3. Although the present study demonstrates that CYB5R3 is required for furapyrrodole‐induced NPC differentiation in vitro, definitive evidence for its role in vivo will require NPC‐specific CYB5R3 knockout mice. This will be an important direction for future studies.

Furapyrrodole also exerts anti‐neuroinflammatory effects within the CNS following immune cell infiltration at the peak stage of EAE. This anti‐inflammatory activity may limit further activation, proliferation, and differentiation of encephalitogenic CD4^+^ T cells in the CNS. Because calcineurin, which is activated by cytosolic Ca^2+^, is known to trigger many pro‐inflammatory cytokines via NFAT activation [108, 109, 110, 111], it is plausible that furapyrrodole‐induced anti‐inflammatory function may also be mediated by furapyrrodole‐mediated CYB5R3 function activation, which suppresses cytosolic Ca^2+^ levels, in these cells. Defining the cell‐type–specific roles of CYB5R3 and the downstream signaling pathways underlying these anti‐inflammatory effects will be an important focus of future studies.

Although it is also possible that furapyrrodole targets peripheral immune cells, because Notch signaling is also involved in immune function [112, 113], furapyrrodole did not suppress the numbers of CD4^+^ T cells, Th1 cells, or Th17 cells in the spleen of EAE mice. In addition, CYB5R3 is not well expressed in immune cells, including CD4^+^ T cells, in the spleen. These findings suggest that furapyrrodole has minimal impact on the immune system in the periphery, which is crucial for protecting against pathogens. In summary, the neuroregenerative and anti‐inflammatory functions of furapyrrodole in the CNS, in conjunction with its minimal adverse effects and limitted impact on peripheral immune responses, highlight its strong potential as a therapeutic candidate for MS.

NPCs are exposed to inflammatory cytokines, neurotransmitters, and ROS, which can alter their genomic profile due to the absence of protective stem cell niches maintaining their undifferentiated states [114]. Considering that our study targeted NPCs in the spinal cord, we anticipate that these cells will respond similarly to NPCs in the brains of MS patients, as NPCs are present at MS lesions in humans [13]. Therefore, it is reasonable to expect that furapyrrodole would exert comparable therapeutic effects in MS. In addition, given that furapyrrodole promotes neurogenesis and oligodendrogenesis and suppresses neuroinflammation within the spinal cord, it holds strong potential for application in the treatment of other neurodegenerative conditions involving spinal neurons, such as spinal cord injury and amyotrophic lateral sclerosis, in which NPCs are also upregulated [115, 116]. Furthermore, because furapyrrodole supports NSC differentiation, it may be beneficial for treating other neurodegenerative diseases in the brain, such as Alzheimer's disease. These possibilities will be explored in future studies.

There are some limitations to our study. The molecular mechanism by which furapyrrodole activates CYB5R3 remains elusive. Clarifying the nature of CYB5R3 activation, whether through allosteric modulation, conformational stabilization, or direct interaction with the flavin active site, will be critical to understanding its broader pharmacodynamic impact. Furthermore, although we demonstrated that systemically administered furapyrrodole reaches the spinal cord, its cell‐type‐specific and subcellular distribution in vivo remains unknown. Visualizing the localization of fluorescence‐labeled furapyrrodole relative to CYB5R3 in NPCs and other CNS cell populations will further strengthen the mechanistic and translational significance of our findings. Comprehensive pharmacokinetic and pharmacodynamic analyses will also be necessary to optimize dosing strategies and evaluate its therapeutic potential.

## 4 Methods

### 4.1 Cell Culture

Assay cells (LS174T Notch cells) were established as previously described [38]. Mouse pluripotent NSC‐like C17.2 cells were purchased from KAC Co., Ltd. (Kyoto, Japan). Both cell types were maintained in proliferation medium consisting of Dulbecco's modified Eagle medium (DMEM; Wako Pure Chemical Industries, Osaka, Japan) supplemented with 10% fetal bovine serum (BioWest, Nuaillé, France; Sigma–Aldrich, St. Louis, MO, USA), 100 units/mL penicillin (Sigma–Aldrich), and 100 µg/mL streptomycin (Sigma–Aldrich). Neural differentiation of C17.2 cells was induced by transferring the cells to differentiation medium, which consisted of Neurobasal medium (Gibco, Waltham, MA, USA) supplemented with 2% B‐27 supplement without vitamin A (Gibco) and 1% antibiotic‐antimycotic (Gibco). All cells were cultured at 37°C in a humidified atmosphere of 5% CO_2_ and 95% air.

### 4.2 Notch Signaling–Mediated Transcriptional Activity Assay

Assay cells (LS174T Notch cells; 2.0  ×  10^4^ cells per well) were constructed with a 12 × RBP‐J binding site (12 copies of CGTGGGAA) before the luciferase‐encoding gene. Because the trimer complex of NICD, MAML, and RBP‐J accelerates luciferase expression, this assay detects the activation of Notch signaling. Assay cells were seeded in a 96‐well white plate and incubated for 12 h at 37°C. Dox (50 ng/mL) was added to each well to induce the expression of exogenous Notch1 (1704–2531) protein, and the cells were incubated at 37°C for 12 h. The medium was then changed to Dox‐free medium containing test samples, and the cells were incubated at 37°C for an additional 12 h. Luciferase activity was measured using the Bright‐Glo Luciferase Assay System (Promega Corp., Madison, WI, USA) according to the manufacturer's protocol. Cell viability was determined simultaneously using a fluorometric microculture cytotoxicity assay. After the assay, the cells were seeded in a 96‐well black plate at 2.0  ×  10^4^ cells per well and then incubated at 37°C for 24 h. Samples were added, and the cells were incubated at 37°C for 12 h. Next, the medium was removed, and fluorescein diacetate solution in PBS (10 µg/mL) was added to each well, followed by incubation at 37°C for 1 h. Cell viability was determined based on the intensity of fluorescein fluorescence. Luminescence and fluorescence measurements were performed using a SpectraMax i3x instrument (Molecular Devices LLC, San Jose, CA, USA).

### 4.3 X‐ray Crystallographic Analysis

Crystals of compound **3c** were prepared as follows. First, a total of 8 mg of **3c** was dissolved in 1 mL of hexane containing one drop of chloroform in a 5‐mL vial. The solution was kept at 4°C in the dark for 4 days to allow for crystallization. The resulting crystals were subjected to x‐ray crystallographic analysis, and the structural data were deposited in the Cambridge Crystallographic Data Centre (accession number CCDC2478375).

### 4.4 Western Blotting Analysis

C17.2 cells (10^6^ cells per well) were suspended in 2 mL of proliferation medium and incubated at 37°C for 12 h. The medium was then removed and replaced with differentiation medium, and the cells were incubated at 37°C for an additional 12 h. After incubation, the medium was removed, replaced with differentiation medium containing samples, and incubated at 37°C for 12 h. Finally, the cells were collected by centrifugation and washed twice with PBS. Whole‐cell lysates were prepared using lysis buffer (20 mm Tris, 150 mm NaCl, 0.5% sodium deoxycholate, 10 mM EDTA, 1 mm sodium orthovanadate, and 0.1 mm NaF [pH 7.4]) supplemented with protease inhibitor cocktail (Nacalai Tesque, Kyoto, Japan). The resulting lysates were electrophoresed on 10% or 12.5% polyacrylamide gels under reducing and denaturing conditions, and the resolved proteins were electrophoretically transferred onto a PVDF membrane (Bio‐Rad Laboratories, Hercules, CA, USA). After blocking of the membrane with 5% skim milk in Tris‐buffered saline (TBS) with 0.1% Tween 20 (TBST) at room temperature for 1 h, the blots were incubated overnight at 4°C with the following primary antibodies; anti–β‐actin (#A2228, Sigma–Aldrich) at 1:5000 in TBS (20 mm Tris, 150 mm NaCl [pH 7.6]), anti‐HES1 (#11988S, Cell Signaling Technology, Danvers, MA, USA) at 1:1000 (XL‐Enhancer Solution 1st; APRO Science, Tokushima, Japan), anti‐NICD (#3608S, Cell Signaling Technology) at 1:1000 (XL‐Enhancer Solution 1st), anti‐ASCL1 (#556604, BD Biosciences, Franklin Lakes, NJ, USA) at 1:1000 (XL‐Enhancer Solution 1st), anti‐MAML (#4608S, Cell Signaling Technology) at 1:1000 (XL‐Enhancer Solution 1st), anti–RBP‐J (#2ARBP3, Institute of Immunology, Tokyo, Japan) at 1:1000 (XL‐Enhancer Solution 1st), anti‐CYB5R3 (#sc‐398043, Santa Cruz Biotechnology, Dallas, TX, USA) at 1:200 (XL‐Enhancer Solution 1st), anti–perilipin 2 (#ab108323, Abcam, Cambridge, UK) at 1:1000 (XL‐Enhancer Solution 1st), anti‐PARP (#sc‐7150, Santa Cruz Biotechnology) at 1:1000 (XL‐Enhancer Solution 1st), and anti–α‐tubulin (#A11126, Invitrogen, Waltham, MA, USA) at 1:1000 (XL‐Enhancer Solution 1st). β‐Actin was evaluated as an internal control. After washing with TBST, each membrane was incubated at room temperature with horseradish peroxidase–conjugated anti‐mouse IgG (#NA931VS, GE Healthcare Japan, Tokyo, Japan) at 1:5000 (β‐actin, Ascl1, CYB5R3, α‐tubulin: 3% skim milk in TBST), anti‐rabbit IgG at 1:5000 (NICD, Hes1, perilipin 2, PARP: 3% skim milk in TBST) or anti‐rat IgG at 1:5000 (RBP‐J: 3% skim milk in TBST). The blots were then washed with TBST and incubated at room temperature for 1 h with HRP‐linked secondary antibodies. After washing the blots with TBST, chemiluminescence was evaluated using ECL Select reagent (#RPN2235, GE Healthcare Japan) or Immobilon reagent (#WBKLS0100; Merck Millipore, Darmstadt, Germany) on a ChemiDoc XRS+ system (Bio‐Rad Laboratories).

### 4.5 Preparation of Furapyrrodole‐Conjugated Beads

Furapyrrodole‐conjugated beads were prepared as previously described [117]. Briefly, photoaffinity linker–coated agarose beads were washed with methanol and subsequently with Milli‐Q water using a spin column. The beads were then transferred to a glass vial, and furapyrrodole (in methanol) was added. The mixture was concentrated and dried in vacuo, and the resulting beads were irradiated at 365 nm (4 J/cm^2^) using a UV cross‐linker. Finally, the beads were washed with methanol and Milli‐Q water and suspended in PBS.

### 4.6 Identification of the Target Protein of Furapyrrodole by Pull‐Down Assay

Prepared beads (100 µL) were washed twice with wash buffer (PBS + 0.1% Tween 20 + Protease Inhibitor EDTA‐free [1 tablet]). Next, 1 mL of C17.2 cell lysate (protein concentration: 5 mg/mL) was added to the beads and incubated with rotation at 4°C for 6 h. After centrifugation at 1000 rpm, the supernatant was discarded. The pellet containing the beads was washed four times with wash buffer. To elute bound proteins, 30 µL of 2 × SDS sample buffer was added, and the mixture was heated at 100°C for 5 min. The supernatant was collected after centrifugation and analyzed by SDS‐PAGE followed by silver staining. For the competition assay, 1 µmol of furapyrrodole was pre‐incubated with cell lysate at 4°C for 4 h with rotation prior to mixing with the beads.

### 4.7 Identification of Furapyrrodole‐Binding Proteins by Mass Spectrometry

The protein‐containing region of the SDS‐PAGE gel was excised and washed successively with water, decolorizing solution (50% acetonitrile and 50 µm NH_4_HCO_3_), and acetonitrile. The gel pieces were then dried in vacuo. Aqueous alkylating solution (100 µm iodoacetamide and 100 µm NH_4_HCO_3_) was added to the dried gel pieces, and the mixture was incubated at 37°C for 30 min. The gel pieces were subsequently washed with 100 µm NH_4_HCO_3_, dehydrated with acetonitrile, and dried in vacuo. The dried gel pieces were rehydrated in digestion buffer (10 µm Tris‐HCl [pH 8.8], 25 ng/mL trypsin) at 4°C for 30 min and incubated at 37°C overnight. The resulting digested peptides were extracted using extraction solution (0.05% trifluoroacetic acid and 50% acetonitrile), dried in vacuo, and analyzed by LC‐MS/MS using an Easy‐nLC 1000 system coupled to a Q Exactive hybrid quadrupole‐Orbitrap mass spectrometer (Thermo Fisher Scientific). Peptides were separated on a reverse‐phase nanospray column (NTCC‐360/75‐3‐105, Nikkyo Technos) using a linear gradient of solvent A (water with 0.1% formic acid) and solvent B (acetonitrile with 0.1% formic acid), with solvent B increasing from 0% to 35% over 10 min. MS and MS/MS data were acquired using the Top10 method. The acquired data were searched using Mascot server 2.6 (Matrix Science) with the following parameters: database; NCBIprot (mouse); enzyme = trypsin; max missed cleavages = 3; variable modifications = acetyl (protein N‐terminal), oxidation (M), Gln‐ > pyro‐Glu (N‐terminal Q), carbamidomethyl (C); peptide mass tolerance = ±15 ppm; fragment mass tolerance = ±30 mmu.

### 4.8 Cellular Thermal Shift Assay

C17.2 cell lysates were incubated with either DMSO or 20 µm furapyrrodole at room temperature for 2 h, aliquoted into microtubes, and heated at defined temperatures (54.2°C, 56.3°C, 59.4°C, 63.2°C, 66.6°C, or 68.7°C) for 3 min. The samples were then cooled to 20°C for 30 s and centrifuged, and the resulting supernatants were analyzed by Western blotting to evaluate target protein thermal stability.

### 4.9 Expression of GST and CYB5R3

pGEX6p‐1‐CYB5R3 ΔN26 was a gift from Masaaki Komatsu (Addgene plasmid #204557) (Ishimura et al., 2022). GST‐CYB5R3 was transformed into *E. coli* BL21, which was cultured overnight at 37°C in 30 mL of LB broth supplemented with 2.5 mg of ampicillin. The culture was then diluted with LB broth to adjust the optical density at 600 nm to 0.1, resulting in a final volume of 250 mL, and then 12.5 mg of ampicillin was added. After the addition of isopropyl‐1‐thio‐β‐D‐galactopyranoside (final concentration 0.1 mm) to induce recombinant protein expression, the cells were further cultured at 37°C for 4 h. The cells were then harvested by centrifugation at 6000 rpm for 5 min at 4°C, and the resulting pellets were lysed using lysis buffer (30 mm Tris‐HCl [pH 7.5], 0.1 m NaCl, 1% Triton X‐100, 1 mm DTT, protease inhibitor cocktail [Roche, Basel, Switzerland]). The lysates were sonicated, mixed by rotation for 30 min at 4°C, and centrifuged at 13000 rpm for 10 min at 4°C. The resulting pellets were resuspended in SDS sample buffer, vortexed, boiled at 100°C for 5 min, and centrifuged at 13000 rpm for 10 min at 4°C. The resulting supernatants were collected as the pellet (P) samples. A portion of each supernatant was also mixed with SDS sample buffer and used as the supernatant (A) samples. For affinity purification, 10 mL of the supernatant was incubated with 600 µL of a 50% slurry of glutathione‐Sepharose 4B resin (# 17075605; Cytiva, Marlborough, MA, USA) for 3 h at 4°C with rotation. After centrifugation at 2000 rpm for 5 min at 4°C, SDS sample buffer was added to each supernatant, which was used as the pass‐through sample (B). The beads were then washed, and a portion of the beads was mixed with SDS sample buffer and used as the bead‐bound (C) samples. To elute GST alone, glutathione was added to the beads and incubated with rotation at 4°C for 10 min. The beads were then centrifuged, and the resulting pellets and supernatants were collected as samples (D) and (E), respectively, after the addition of SDS sample buffer. For GST‐CYB5R3, the GST tag was cleaved using PreScission protease (#27084301; Cytiva, Marlborough, MA, USA), and the recombinant protein was subsequently eluted. After centrifugation at 2000 rpm for 5 min at 4°C, each pellet (D’) and supernatant were collected as samples after adding SDS sample buffer. Each purified recombinant protein was dialyzed against PBS. The expression and purification of GST and CYB5R3 were confirmed by SDS‐PAGE followed by Coomassie Brilliant Blue staining.

### 4.10 CD Spectroscopy Analysis of CYB5R3

Circular dichroism (CD) spectra were recorded using a spectropolarimeter (J‐1500, JASCO, Tokyo, Japan). A 300‐µL solution of protein in PBS was placed in a quartz cuvette, and measurements were acquired over the range of 200–250 nm, with a scan speed of 50 nm/min, resolution of 0.5 nm, and a constant temperature of 24°C. Following the initial measurement, furapyrrodole (dissolved in ethanol) was added sequentially in 1‐µL increments to achieve final concentrations of 4, 8, 16, 24, 32, 48, 64, and 128 µm. After each addition, the sample was mixed by gentle inversion, and CD spectra were recorded under the same conditions. Changes in CD signal upon compound binding were monitored by measuring the ellipticity of GST at 208.5 nm and that of CYB5R3 at 210 nmm. Binding constants (Kd) were determined using Lineweaver–Burk analysis by plotting the reciprocal of the compound concentration against the reciprocal of the change in ellipticity. The Kd values were calculated from the x‐intercepts of the linear regression lines.

### 4.11 In silico Prediction of the Interaction Between CYB5R3 and Furapyrrodole

The full‐length amino acid sequence of mouse CYB5R3 (NCBI reference sequence: NP_084063.1) was used to generate a multiple sequence alignment using MMseqs2 (version: 17.b804f) [118] through colabfold_search [119]. The structure of the complex consisting of CYB5R3 with FAD and NADH was predicted using a local version of AlphaFold3 (version 3.0.1) based on the obtained alignment and SMILES representations of FAD and NADH. The resulting structure model was refined using the Protein Preparation Wizard on Maestro (version 2025‐3, Schrödinger, New York, NY, USA). Potential ligand‐binding sites of CYB5R3 were identified using SiteMap (version 2025‐3, Schrödinger). These sites were then subjected to Induced Fit Docking (IFD) (version 2025‐3, Schrödinger) to predict the binding mode of furapyrrodole.

SiteMap was configured to generate five potential ligand‐binding sites. For each binding site, the receptor grid box used for the IFD calculation was defined such that it fully encompassed the corresponding site. The final binding mode was selected as the pose providing the most favorable docking score, using a receptor grid box centered on Val168 and Cys298 (grid box size: 18 Å^3^; docking score: −6.059 kcal/mol). All other software settings were retained at the default values.

### 4.12 CYB5R3 Knockdown in C17.2 Cells

C17.2 cells were transfected with CYB5R3 siRNA (#sc‐62174, Santa Cruz Biotechnology) or control siRNA (#sc‐37007, Santa Cruz Biotechnology) using Lipofectamine 2000 (Invitrogen) according to the manufacturer's recommendations. Briefly, Lipofectamine 2000 was gently mixed with antibiotic‐free Opti‐MEM (Invitrogen) at a ratio of 9 µL:150 µL. An aliquot of CYB5R3 or control siRNA was mixed with the Lipofectamine 2000/Opti‐MEM mixture to achieve a final concentration of 100 nm. Following incubation at room temperature for 5 min, 250 µL of each mixture was added to C17.2 cells in 2 mL of proliferation medium. CYB5R3 and control siRNA–transfected cells were incubated at 37°C for 24 h. After incubation, the cells were collected for further studies, and protein expression was evaluated by Western blotting.

### 4.13 Measurement of CYB5R3 Enzyme Activity

CYB5R3 activity in C17.2 cell lysates was measured using a Cytochrome c Reductase (NADPH) Assay Kit (#CY0100, Sigma–Aldrich) following the manufacturer's protocol. Briefly, C17.2 cells were collected and lysed to prepare a lysate with a protein concentration of 5 mg/mL. To 950 µL of working solution (prepared by dissolving 9 mg of cytochrome c in 20 mL of assay buffer), 30 µL of cell lysate, 20 µL of oxidase inhibitor, and furapyrrodole (final conc. 40 µm) were added. Subsequently, 100 µL of 0.85 mg/mL NADPH solution was added, and the mixture was gently pipetted and incubated at room temperature for 5 min. The absorbance at 550 nm, which is characteristic of the reduced form of cytochrome c, was then measured using a spectrophotometer (U‐5100, Hitachi, Tokyo, Japan).

### 4.14 Measurement of Mitochondrial Membrane Potential

Following the specific treatments, the mitochondrial membrane potential of various cell lines was evaluated using a JC‐1 MitoMP Detection Kit (#MT09, Dojindo), which detects potential‐dependent accumulation of the JC‐1 dye within mitochondria. C17.2 cells (10^5^ cells/mL) were treated with each compound for 1 h at 37°C and then incubated with JC‐1 for an additional 1 h at 37°C. After incubation, the imaging buffer solution was added, and the cells were observed under a fluorescence microscope. Fluorescence was detected using a SpectraMax i3x plate reader (Molecular Devices). Emission wavelengths of 535 and 595 nm were used to detect the monomeric and aggregated forms of JC‐1, respectively. The ratio of aggregated to monomeric JC‐1 fluorescence was used as an indicator of mitochondrial membrane potential.

### 4.15 Measurement of Intracellular Calcium Concentration

Confluent C17.2 cells, cultured in six 10‐cm dishes, were collected into a 50‐mL tube. After centrifugation, the cell pellet was washed with recording medium (20 mmol/L HEPES, 115 mmol/L NaCl, 5.4 mmol/L KCl, 0.8 mmol/L MgCl_2_, 1.8 mmol/l CaCl_2_, 13.8 mmol/L glucose [pH 7.4]). The cells were suspended in 10 mL of recording medium containing 5 µm fura‐2‐AM (Dojindo) and incubated at 37°C in the dark for 1 h. The cells were then washed twice with recording medium to remove excess fura‐2‐AM and extracellular Ca^2+^, and the washed cells were resuspended in recording medium and kept at room temperature. Changes in the intracellular Ca^2+^ level immediately after the addition of DMSO, 1 µm thapsigargin, or 20 µm furapyrrodole were measured at 25°C using a spectrofluorometer (RF‐6000, Shimadzu, Kyoto, Japan). Fura‐2 fluorescence intensity was monitored at an excitation wavelength of 340 nm and an emission wavelength of 510 nm.

### 4.16 Measurement of Mitochondrial Calcium Ion Concentration

C17.2 cells were seeded in 35‐mm dishes at a density of 2.0 × 10^5^ cells/mL in 2 mL of proliferation medium and cultured for 12 h at 37°C in a humidified atmosphere containing 5% CO_2_. The medium was then removed and replaced with differentiation medium. For transfection, 2.5 µL of PLUS Reagent and pCAG mito‐GCaMP5G plasmid DNA (Addgene, #105009) were added to 500 µL of Opti‐MEM and mixed by gentle inversion, followed by incubation for 10 min at room temperature. Lipofectamine LTX was then added, and the mixture was gently inverted and incubated for 25 min at room temperature. The transfection mixture was subsequently added to the cells, which were cultured for an additional 48 h. For mitochondrial Ca^2^^+^ imaging, cells were observed before and after treatment with furapyrrodole (FP; 20 µm final concentration, 0.5% DMSO). Fluorescence images were acquired by confocal microscopy (FluoView FV4000, Evident Scientific) before FP addition and from 30 s after FP addition at 10‐s intervals.

### 4.17 Nuclear Fractionation of C17.2 Cells

C17.2 cells treated with compounds for 12 h in 6‐well plates were collected, centrifuged, and resuspended in Buffer A (20 mm HEPES‐KOH [pH 7.5], 10 mm KCl, 1.5 mm MgCl_2_, 1 mM EDTA, 1 mm EGTA, 250 mm sucrose, 1 mm DTT). After incubation for 10 min at room temperature, the cells were disrupted by repeated passage through a syringe, and nuclei were pelleted by centrifugation and washed twice with Buffer A. Nuclear lysis was achieved by incubation in Buffer C (20 mm HEPES‐KOH [pH 7.5], 10 mm KCl, 1.5 mm MgCl_2_, 1 mm EDTA, 1 mm EGTA, 250 mm sucrose, 1 mm DTT, 500 mm NaCl) for 10 min at room temperature. After centrifugation, the supernatant containing nuclear proteins was collected for Western blot analysis.

### 4.18 NSC Differentiation Assay

C17.2 cells were plated using proliferation medium at 2.0  ×  10^5^ cells/mL on cover glass (Matsunami Glass, Osaka, Japan) coated with poly‐L‐lysine and fibronectin/laminin. After incubation for 24 h, the cells were washed with DMEM and treated with 10 µm DAPT or furapyrrodole (5, 10 µm) in differentiation medium for 4 days. Neurons were immunostained with Tuj1 (green) (#MAB1195, R&D Systems, Minneapolis, MN, USA). Nuclei were immunostained with ProLong Gold antifade reagent with DAPI (# P36935, Invitrogen). Astrocytes were immunostained with GFAP (#ST‐01415, Stem Cell Technologies, Vancouver, BC, Canada) and detected using confocal microscopy.

### 4.19 OCR Experiments

Oxygen consumption rate [59] was measured using a Seahorse XFe96 Analyzer (Agilent Technologies) with Seahorse XFe96 microplates (Agilent Technologies, #103792‐100) and the Seahorse XF Cell Mito Stress Test kit (Agilent Technologies). C17.2 cells were seeded at 1.0 × 10^4^ cells per well in 80 µL of culture medium consisting of Dulbecco's modified Eagle medium (DMEM; Wako Pure Chemical Industries, Osaka, Japan) supplemented with 10% fetal bovine serum (FBS; BioWest, Nuaillé, France; Sigma–Aldrich, St. Louis, MO, USA), 100 units/mL penicillin (Sigma–Aldrich), and 100 µg/mL streptomycin (Sigma–Aldrich), and cultured for 24 h at 37°C in a humidified atmosphere containing 5% CO_2_ [120].

For OCR measurements, the culture medium was replaced twice with DMEM‐based assay medium (Agilent Technologies, #103575‐100) supplemented with 1% GlutaMAX (Thermo Fisher Scientific, #35050‐061) and 1% sodium pyruvate (Thermo Fisher Scientific, #11360‐070). The final volume was adjusted to 150 µL per well. The cells were then incubated for 1 h at 37°C in a CO_2_‐free atmosphere. During the OCR measurement, 25 µL of each compound solution prepared in assay medium was injected into the wells using the Seahorse XFe96 Analyzer. The final concentration of DMSO was adjusted to 0.5% in all treatment conditions.

The final concentrations of FCCP (TCI, C3463), furapyrrodole (FP), DAPT, and antimycin A were 0.0625, 0.125, 0.25, 0.5, 1.0, and 2.0 µm; 1.1, 2.2, 3.3, 6.6, 10, 20, and 30 µm; 1.1, 3.3, 10, and 30; and 1 µm, respectively. OCR was measured at 6‐min intervals for 1 h, resulting in a total of 11 measurement cycles, including the initial measurement. For data analysis, the OCR value obtained immediately before compound injection was set to 100% for each well, and subsequent OCR measurements were normalized to this baseline value. Each condition was measured in three technical replicates (*n* = 3), and data are presented as the mean ± SD.

### 4.20 Animals

Healthy male and female C57BL/6J mice (Jackson Laboratories, #000664) were used in the experiments. Breeder mice were housed in solid‐bottom caging with standard bedding under temperature‐controlled conditions (23°C ± 1°C) with 12‐h reversed light/ dark cycles (6 am–6 pm). Standard rodent chow and water were provided ad libitum. C57BL/6‐Tg(Nes‐cre/ERT2)KEisc/J (# 016261) [121] and *Tomato^fl^* mice (# 007914) in a C57BL/6J background were purchased from Jackson Laboratories. The studies described here were approved by the University of Illinois at Urbana‐Champaign Institutional Animal Care and Use Committee (protocol no. 25135 and 24179).

### 4.21 Active EAE Induction

Six‐ to eight‐week‐old mice were randomly subjected to EAE. EAE was induced via two subcutaneous injections of 100 µg of MOG_35‐55_ peptide (United Peptides) emulsified in complete Freund's adjuvant (#F5881, Sigma), including 400 µg/mouse of heat‐killed mycobacteria (#DF3114‐33‐8, Fisher) at 0 and 7 dpi. Pertussis toxin (List Biological Laboratories, 200 ng/mouse) was injected i.p. on 0, 2, and 7 dpi. EAE behavioral scores were defined as follows: 0.5, partial tail limpness; 1, tail limpness; 1.5, reversible impaired righting reflex; 2, impaired righting reflex; 2.5, paralysis of one hind limb; 3, paralysis of both hind limbs; 3.5, paralysis of both hind limbs and one forelimb; 4, hind limb and forelimb paralysis; 5, death. Wet food and access to water gel were supplied when the mice exhibited a score of 2.

### 4.22 Tissue Collection and Processing

Mice were anesthetized using isoflurane and transcardially perfused with TBS followed by 4% paraformaldehyde (PFA). Spinal cords were extracted, post‐fixed in 4% PFA overnight, and cryoprotected in 30% sucrose. Tissues were then embedded in Tissue‐Tek Optimal Cutting Temperature compound, frozen on dry ice, and stored at −80°C until sectioning. Coronal cryosections (30 µm) were prepared for immunohistochemistry, stored in 0.02% sodium azide at 4°C, and mounted onto poly‐L‐lysine–coated glass slides.

### 4.23 Immunohistochemistry and Antibodies

Spinal cord sections were mounted on poly‐L‐lysine–coated glass slides, permeabilized with 0.05% Triton‐X for 15 min at room temperature, and blocked with 2% bovine serum albumin (BSA) for 1 h at room temperature. Slices were incubated with primary antibodies overnight at 4°C, followed by incubation with species‐specific secondary antibodies for 2 h at room temperature. The following antibodies were used in this study: anti‐CYB5R3 (Novus, #NBP151984, 1:300), anti‐GFAP (Invitrogen, #13‐030‐0, 1:300), anti‐PLP (Millipore, #MABN2620 1, 1:200), anti‐beta Tubulin3/Tuj1 (Genetex, #GTX85469, 1:1000), anti‐Olig‐2 (Millipore, #AB9610, 1:300), anti‐Nestin (Invitrogen, #MA5‐32272, 1:200), anti‐ PDGF Receptor alpha (Cell Signaling Technology, #3174, 1:300), anti‐ASCL1 (Invitrogen, #PIPA540527, 1:300), anti‐Notch1 (Novus, #NBP178292, 1:300), anti‐HES1 (Invitrogen, #PIMA532258, 1:200), and anti‐Ki67 (Invitrogen, #PA5143573, 1:1000). Secondary antibodies used (all at 1:500 dilution) in the study included goat anti‐rat Alexa Fluor 555 (Invitrogen, #A21434), chicken anti‐goat Alexa Fluor 647 (Invitrogen, #A21469), donkey anti‐mouse Alexa Fluor 488 (Invitrogen, #A21202), donkey anti‐rabbit Alexa Fluor 568 (Invitrogen, #A10042), and donkey anti‐goat Alexa Fluor 568 (Invitrogen, #A11057). Prolong Gold mounting medium was applied to the dried mounted sections, and coverslips were gently placed across all samples. Slides were stored at −20°C.

### 4.24 Nissl Staining and Analysis

The lumbar regions of spinal cords were harvested after perfusion with 4% PFA in PBS and embedded in paraffin blocks. Tissue slices (10 µm) were stained with 0.1% Cresyl Violet (Nissl staining) for visualization of neuronal cell bodies, rinsed briefly in distilled water, differentiated in 95% ethanol, dehydrated through a graded alcohol series, cleared in xylene, and coverslipped with permanent mounting medium. Images were acquired using a brightfield microscope with a 4 × objective. Vacuolization and neuronal loss were quantified using ImageJ software (US National Institutes of Health) by manually outlining the region of interest (ROI) using the wand tool. For each mouse, one datum point represented the average value of the whole lumbar spinal cord analysis.

### 4.25 Luxol Fast Blue Staining and Analysis

Transverse paraffin sections (8 µm) of spinal cord from EAE mice with vehicle or furapyrrodole at 40 dpi were mounted onto poly‐L‐lysine‐coated slides and left to dry overnight. Sections were hydrated with 95% ethyl alcohol, then placed in 1:1 ethanol/chloroform for 5 min. Sections were rehydrated with 95% ethyl alcohol, then incubated in 0.1% Luxol fast blue solution for 12 h. After 16 h, 95% ethyl alcohol was used to wash away excess dye solution, followed by a rinsing step with distilled water. 0.05% Li_2_CO_3_ solution was applied to sections for 30 s, followed by a 70% ethyl alcohol wash until a sharp contrast between gray and white matter became apparent. The sections were immersed in 90%, 95%, and 100% ethyl alcohol, and in 100% xylene, in a gradient dehydration wash, followed by the application of a coverslip. For image analysis, grayscale images were opened in ImageJ, and an automatic threshold was applied. The region of interest was manually drawn to restrict the analysis to the area of white matter regions. Analyses were performed on at least 20 serial sections per sample. The integrated density/area in selected white matters were measured.

### 4.26 Alcian Blue/Periodic Acid‐Schiff Staining

The duodenum was collected following perfusion with 4% PFA in PBS, post‐fixed, and embedded in paraffin. Tissue sections (5 µm) were stained sequentially with Alcian Blue (Sigma), Gill's hematoxylin (Fisher), and periodic acid–Schiff (PAS) reagents (periodic acid 0.5% aqueous, Fisher; Schiff's reagent, Sigma) to visualize goblet cells and the overall tissue architecture. Hematoxylin served as a nuclear counterstain, providing structural context for Alcian Blue– and PAS‐positive mucins. Images were acquired using an EVOS Cell Imaging System (Thermo Fisher). Goblet cell density was quantified as the number of Alcian Blue–/PAS‐positive cells per square millimeter of intestinal crypt area and compared between experimental groups.

### 4.27 Visualization and Image Analysis

Confocal images of lumbar spinal cord coronal sections (L4–L6) were acquired using a Nikon A1 confocal fluorescence microscope equipped with a 20 × objective (numerical aperture 0.75). All images in each set of micrographs were acquired using the same laser intensity and gain values. For all analyses, spinal cord lumbar coronal sections were analyzed based on selected areas per tissue section. Integrated density was determined by selecting an ROI on raw images and then measuring the integrated density/ROI using ImageJ software. A standardized threshold was applied across all ROIs. A colocalization assay was performed using the Coloc2 plugin in Fiji.

### 4.28 Mouse Primary NPC/NSC Culture

EAE was induced in mice using the protocol described in the Active EAE induction section, and the spinal cord was isolated at 20 days after disease onset. The spinal cord was finely minced and incubated for 30 min at 37°C in 1 mL of enzyme solution containing 2.5 U/mL activated papain (Sigma), 2 mm glucose (Millipore), and 200 U/mL DNase I [122] in Neurobasal A medium (Thermo Fisher). The digested tissues were passed through a 70 µm strainer and rinsed with 1 mL of Neurobasal A medium. The 2 mL of liquid was then pipetted into a 15‐mL conical tube and equilibrated at room temperature. Next, 1.1 mL of Percoll (Fisher) was added to achieve a 22% final concentration. The cells were then centrifuged at 2250 rpm at room temperature for 15 min. The resulting supernatant was removed, and the pellet was washed with 2 mL of Neurobasal A medium and centrifuged at 1500 rpm for 5 min. The cells were then resuspended in 500 µL of Neurobasal A medium for fluorescence‐activated cell sorting. The cells were sorted using a Sony SH800 and collected in Neurobasal A medium. Following sorting, cells were centrifuged at 2250 rpm for 10 min and resuspended in 1 mL of Neurobasal A medium with 2% B27 (Thermo Fisher), 1% Glutamax (Thermo Fisher), 100 units of penicillin‐streptomycin, 20 ng/mL human heat‐stable bFGF Recombinant Protein (Fisher), and 20 ng/mL EGF (Sigma). Finally, the cells were seeded at an initial density of 3.0 × 10^6^ cells/well and incubated for 1 week at 37°C with 5% CO_2_ following isolation and then re‐seeded at a density of 3.0 × 10^5^ cells/well for expansion. Mouse cortical neural stem cells were purchased from Millipore and were used for a cell culture study according to the manufacturer's protocol.

### 4.29 Immunocytochemistry

A total of 15 neurospheres were collected from suspensions and placed in a Nunc Lab‐Tek II Chamber Slide System (Thermo Fisher) well. A total of 300 µL of the culture medium was used, with no addition of growth factors. To each well, 10 µm furapyrrodole, 10 µm DAPT (Fisher), or 0.5% DMSO (Fisher) was added, and the cells were cultured for 10 days following treatment. After culture, the medium was removed by aspiration, and the cells were fixed with 4% cold PFA for 20 min at 4°C. The cells were then washed with TBST 3 times for 5 min each. Next, the cells were permeabilized with 0.5% Triton X in TBST for 15 min and washed 3 times for 5 min each. The slides were blocked for 1 h in 2% BSA in TBST and then incubated overnight with primary antibodies. Finally, the cells were stained with anti–beta Tubulin3/Tuj1 (Genetex, #GTX85469, 1:1000), anti‐Ki67 (Thermo Fisher, #MA5‐14520, 1:1000), anti‐GFAP (Thermo Fisher, #13‐0300, 1:300), anti‐Nestin (R&D Biosciences, #MAB2736, 1:200), or anti‐PLP (Millipore, #MABN2620 1:200) antibodies and counterstained with DAPI (Thermo Fisher, 1:10,000).

### 4.30 Measurement of Olfm4 and CYB5R3 Expression in Intestinal Crypts

The duodenum was collected following perfusion with 4% paraformaldehyde in PBS, post‐fixed, and embedded in OCT. Tissue sections (20 µm) were stained with Anti‐Olfm4 rabbit monoclonal antibody (Cell Signaling Technology, #39141, 1:200), Anti‐CYB5R3 (Novus, #NBP151984, 1:300), and counterstained with DAPI. ROIs were drawn around the Olfm4‐positive crypts, and CYB5R3 expression was measured inside the positive areas.

### 4.31 Treatment with a Retrograde Tracer

EAE was randomly induced in 6‐ to 8‐week‐old Nestin‐RFP mice, and the mice were injected with furapyrrodole every other day from 20 dpi to 28 dpi. At 40 dpi, the mice were injected intramuscularly with cholera toxin subunit B (Recombinant), Alexa Fluor 647 conjugate (Fisher) in the tibialis anterior of the right legs. After 1 week, samples and the spinal cord were collected. Spinal cord sections were stained with anti‐ChAT antibody (Fisher Scientific, #11‐058‐02) and imaged via confocal microscopy. Confocal images of lumbar spinal cord coronal sections (L1–L6) were acquired using a Nikon A1 confocal fluorescence microscope equipped with a 60 × objective.

### 4.32 Flow Cytometry

Spleens were suspended in hemolysis buffer to remove red blood cells. The cells were washed, and Fc receptors were blocked by incubation on ice for 7 min. Fluorescent antibodies (Biolegend) were added (1:200) and incubated on ice in the dark for 20 min. Stained cells were washed and then stored in 4% PFA at 4°C. For intracellular cytokine detection, cells were incubated with a stimulation cocktail (eBioscience) and Golgi‐Plug Transport Inhibitor (BD Biosciences), permeabilized, and fixed using a Cytofix/Cytoperm Fixation/Permeabilization Solution Kit (BD Biosciences). Cells were acquired on a Cytek Aurora flow cytometer and analyzed using FCS Express 6 software (De Novo Software). Antibodies for flow cytometry were purchased from BioLegend or eBioscience.

### 4.33 Cytokine Arrays

The spinal cords were pulverized in a mixture of RIPA buffer and protease inhibitor. Samples were centrifuged for 20 min at 4°C, and the supernatant was collected for total protein levels by BCA protein assay. A similar amount of lysate was used for the cytokine array kit (R&D Systems, Minneapolis, MN). Samples were exposed to x‐ray for 20 min, and signals were visualized using the FluorChem R (ProteinSimple, San Jose, CA). The intensity of signals was measured and analyzed by ImageJ software to obtain an average value from duplicate spots. Cytokine expression was calculated as a relative expression to control gp130 expression, and then as the ratio of experimental to control conditions.

### 4.34 LC‐MS/MS Analysis

Mouse spinal cord samples were analyzed for the target compound by LC‐MS at the Carver Metabolomics Core Facility of the Roy J. Carver Biotechnology Center, University of Illinois Urbana‐Champaign. Samples were thawed, and 500 µL of methanol and 5–6 ceramic beads were added to each sample. The samples were then homogenized for 2–3 min using a bead mill. After centrifugation, 50 µL of the supernatant was transferred to a vial pre‐spiked with 5 µL of 10 ng/mL 12‐[[(cyclohexylamino)carbonyl]amino]‐dodecanoic acid (CUDA). A calibration curve was generated ranging from 0.05–100 ng/mL of the target compound in methanol. The target compound was isolated using an Agilent 1290 Infinity II UHPLC system (Agilent Technologies, Santa Clara, CA, USA) equipped with an Agilent Poroshell 120 EC‐C18 (1.9 µm, 2.1 × 50 mm) column (Agilent Technologies). The target was isolated via gradient elution, with mobile phase A consisting of water with 0.1% formic acid and mobile phase B consisting of methanol with 0.1% formic acid. The gradient program was as follows: 0–1 min = 10% B; 1–2 min = 50% B; 2–2.5 min = 95% B; 2.5–4 min = 95%; 4.1–5 min = 10%. The flow rate was 0.30 mL/min, with the column temperature set at 30°C. A Sciex 5500 triple quadrupole mass spectrometer (Sciex, Framingham, MA, USA) operating in positive ionization mode with multiple reaction monitoring was employed to screen for the target compound. The isolated compound was quantified using Sciex MultiQuant 3.1 (Sciex) based on the area ratio of the target compound and the spiked standard, CUDA.

### 4.35 Statistical Analysis

The data were evaluated using unpaired, two‐tailed, Student's *t*‐tests or one‐way ANOVA followed by Tukey's post‐hoc test. Differences were considered significant at *p* < 0.05. No animals were excluded from the analyses. GraphPad Prism software (GraphPad, La Jolla, CA, USA) was used to conduct statistical analyses and generate graphs. Data are presented as the mean ± SEM; number (*n*) and *p*‐values are indicated in the figure legends.

## Author Contributions

Y.N., H. Oba, H.M., M.K., and K.H. conducted in vitro experiments. T.A., H.K., and M. Okano developed chemical reactions and synthesized compounds. Y.N., Y.K., and M. Ohue performed computational analyses. J.S., J.Z., S.A., R.H., and M. Inoue conducted all in vivo EAE studies. J.S. and M. Inoue conducted the primary NPC culture study. Y.N., J.S., S.S., H. Osada, M. Ishibashi, T.A., M. Inoue, and M.A.A. wrote the original manuscript. All authors reviewed and provided feedback on the manuscript.

## Conflicts of Interest

The authors declare no conflicts of interest.

## Supporting information




**Supporting File 1**: advs78560‐sup‐0001‐SuppMat.pdf.

**Supporting File 2**: advs78560‐sup‐0002‐MovieS1.pptx.

## Data Availability

All experimental data, materials, and methods are available in the main text or the Supplementary Information. NMR data of synthetic compounds are available as Figures S21–S45. CCDC 2478375 (for Figure 1D) contains the supplementary crystallographic data for this paper. These data are provided free of charge by the joint Cambridge Crystallographic Data Centre and Fachinformationszentrum Karlsruhe Access Structures service (https://www.ccdc.cam.ac.uk/structures).
