# Supplementary material for: Targeting Mitochondrial Redox Signaling Promotes Neuroregeneration and Functional Recovery in a Multiple Sclerosis Model
Source: Adv Sci (Weinh). 2026 Oct 30:e78560. Online ahead of print. doi: 10.1002/advs.78560 (PMC13721028; doi:10.1002/advs.78560)
Supplement: Supplementary file 1 [file ADVS-9999-e78560-s001.pdf]

Supporting Information

Targeting Mitochondrial Redox Signaling Promotes Neuroregeneration and Functional Recovery in a Multiple Sclerosis Model

Yuki Narushima[1#], Joshua Saylor[2,3#], Shun Saito[1], Jinyan Zhou[2,3], Haruka Kawano[4], Shehata Anwar[2,5], Haruki Oba[1], Rachel Huezo[2], Miyu Okano[4], Haruki Matsuda[1], Makoto Kawatani[6], Kaori Honda[6], Hiroyuki Osada[6,7], Yuta Kikuchi[8], Masahito Ohue[8], Masami Ishibashi[4], Takayoshi Arai[9*], Makoto Inoue[2,3,10*], Midori A. Arai[1*]

[1] Faculty of Science and Technology, Keio University, 3-14-1 Hiyoshi, Kohoku-ku, Yokohama-shi, Kanagawa 223-0061, Japan.

[2] Department of Comparative Biosciences, University of Illinois at Urbana-Champaign, 2001 South Lincoln Avenue, Urbana, Illinois 61802, USA.

[3] Neuroscience Program, University of Illinois at Urbana-Champaign, 405 North Matthews Avenue, Urbana, Illinois 61801, USA.

[4] Graduate School of Pharmaceutical Sciences, Chiba University, 1-8-1 Inohana, Chuo-ku, Chiba-shi, Chiba 260-0856, Japan.

[5] Department of Pathology, Faculty of Veterinary Medicine, Beni-Suef University (BSU), Beni-Suef 62511, Egypt.

[6] RIKEN CSRS, 2-1 Hirosawa, Wako-shi, Saitama 351-0198, Japan.

[7] Institute of Microbial Chemistry (BIKAKEN), 3-14-23 Kamiosaki, Shinagawa-ku, Tokyo 141-0021, Japan.

[8] Department of Computer Science, School of Computing, Institute of Science Tokyo, 4259 Nagatsuta-cho, Midori-ku, Yokohama-shi, Kanagawa 226-0051, Japan.

[9] Graduate School of Science, Chiba University, 1-33 Yayoi-cho, Inage-ku, Chiba-shi, Chiba 263-8522, Japan.

[10] Beckman Institute for Advanced Science and Technology, 405 North Matthews Avenue, Urbana, Illinois 61801, USA.

[#] Equal contribution

Contents

S3-S23 Supplemental Figures (Fig. S1-S20, Table S1)

S24-S40 Experimental data

S41-S65 ^{1}H-NMR and ^{13}C-NMR (Fig. S21-S45)

S66 X-ray report

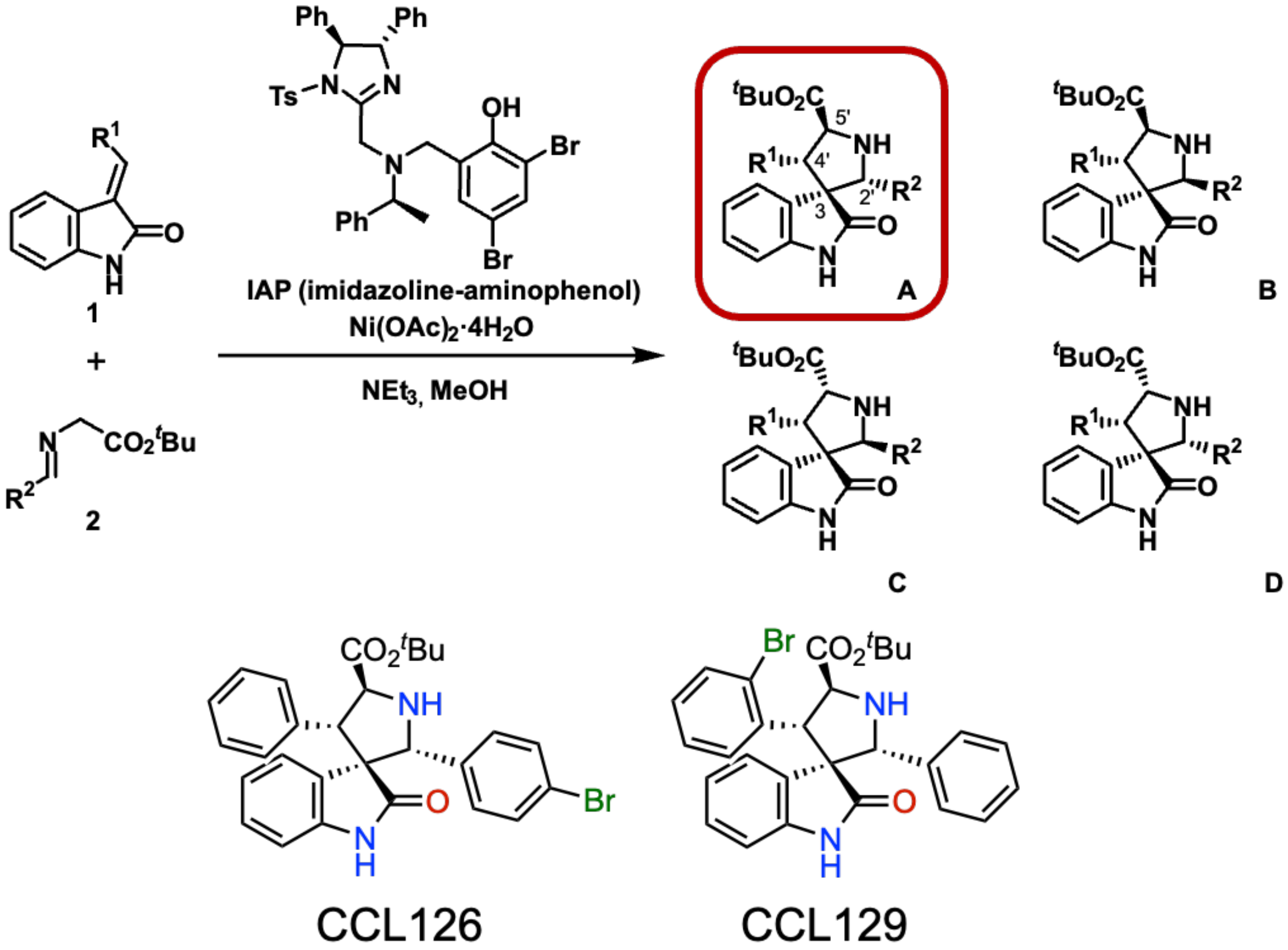

Fig. S1. Synthesis of spirooxindoles. Spirooxindoles were synthesized by [3+2] cycloaddition of methyleneindolinone **1** and iminoester **2** via the Michael-Mannich tandem reaction. Although 16 diastereomers of these spirooxindoles are possible, the use of IAP-nickel catalyst primarily produced conformer A (2'*R*,3*R*,4'*S*,5'*S*).

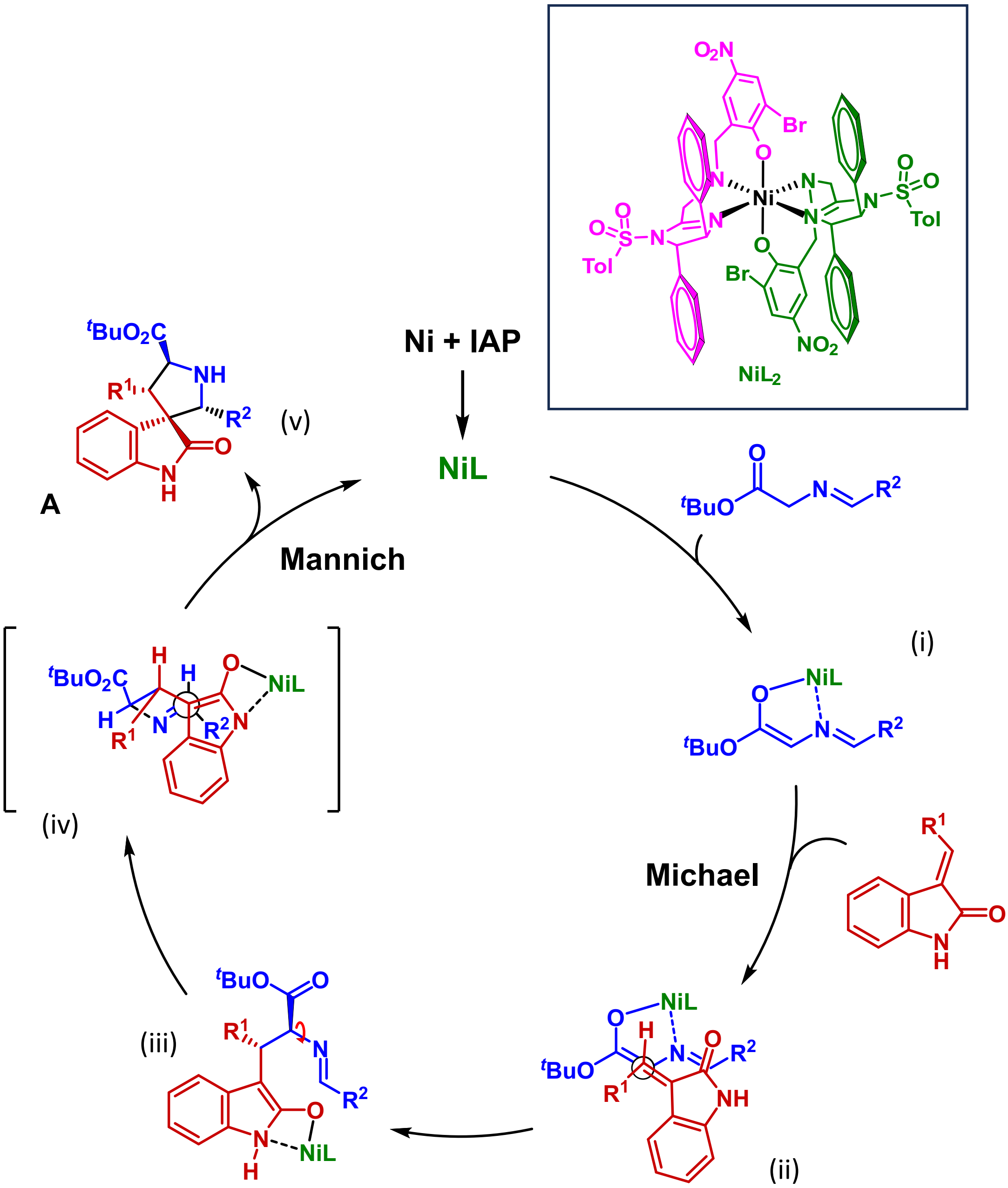

Fig. S2. Mechanism of spirooxindole synthesis. The iminoester coordinates with the IAP–nickel catalyst to form intermediate (i). Michael addition occurs from the iminoester to the methyleneindolinone, producing intermediate (ii). Subsequently, the IAP–nickel catalyst migrates to the methyleneindolinone moiety (iii), and through a sterically stable intermediate (iv), the Mannich reaction ultimately generates compound (v) with the conformer of A.

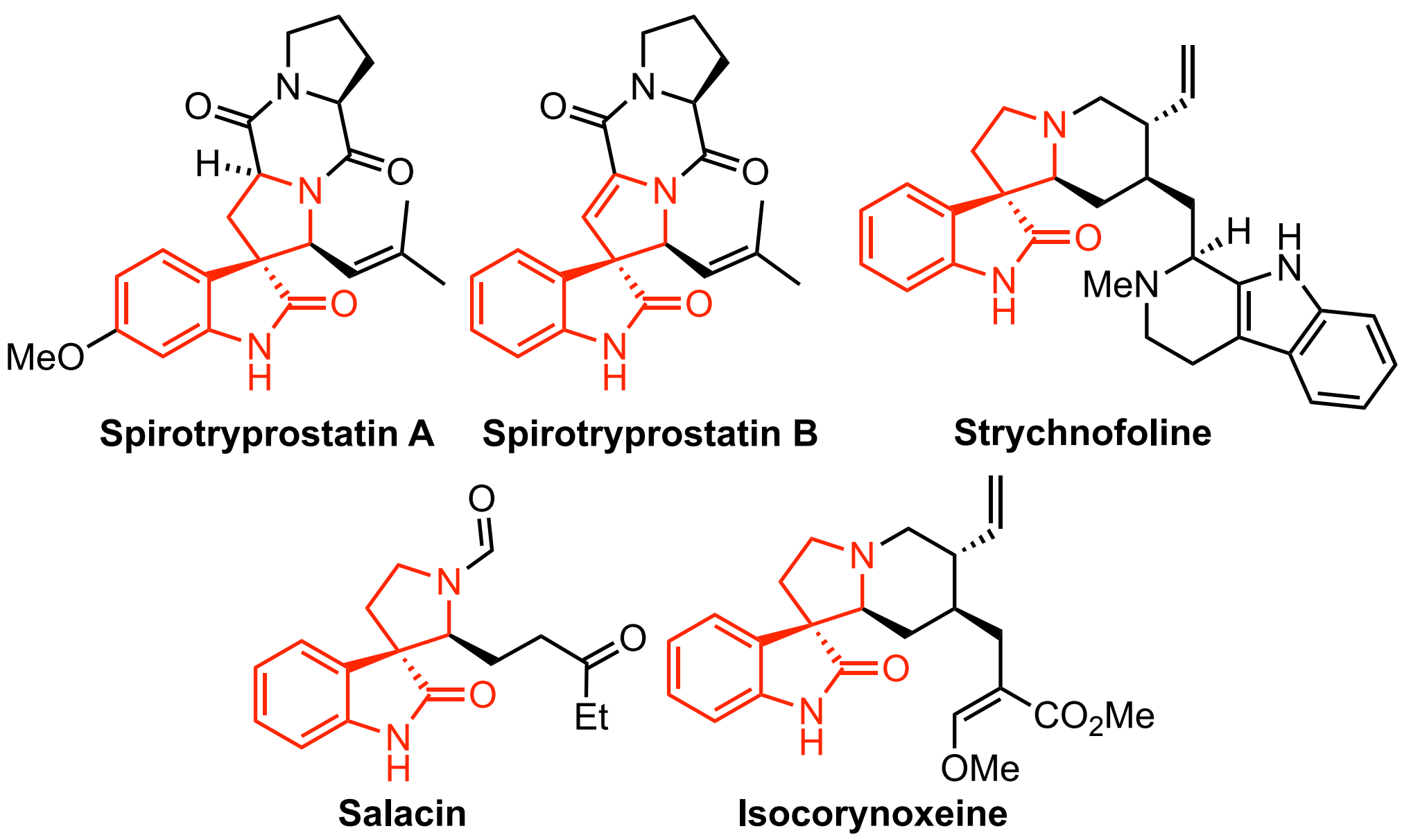

Fig. S3. Spirooxindoles in natural products.

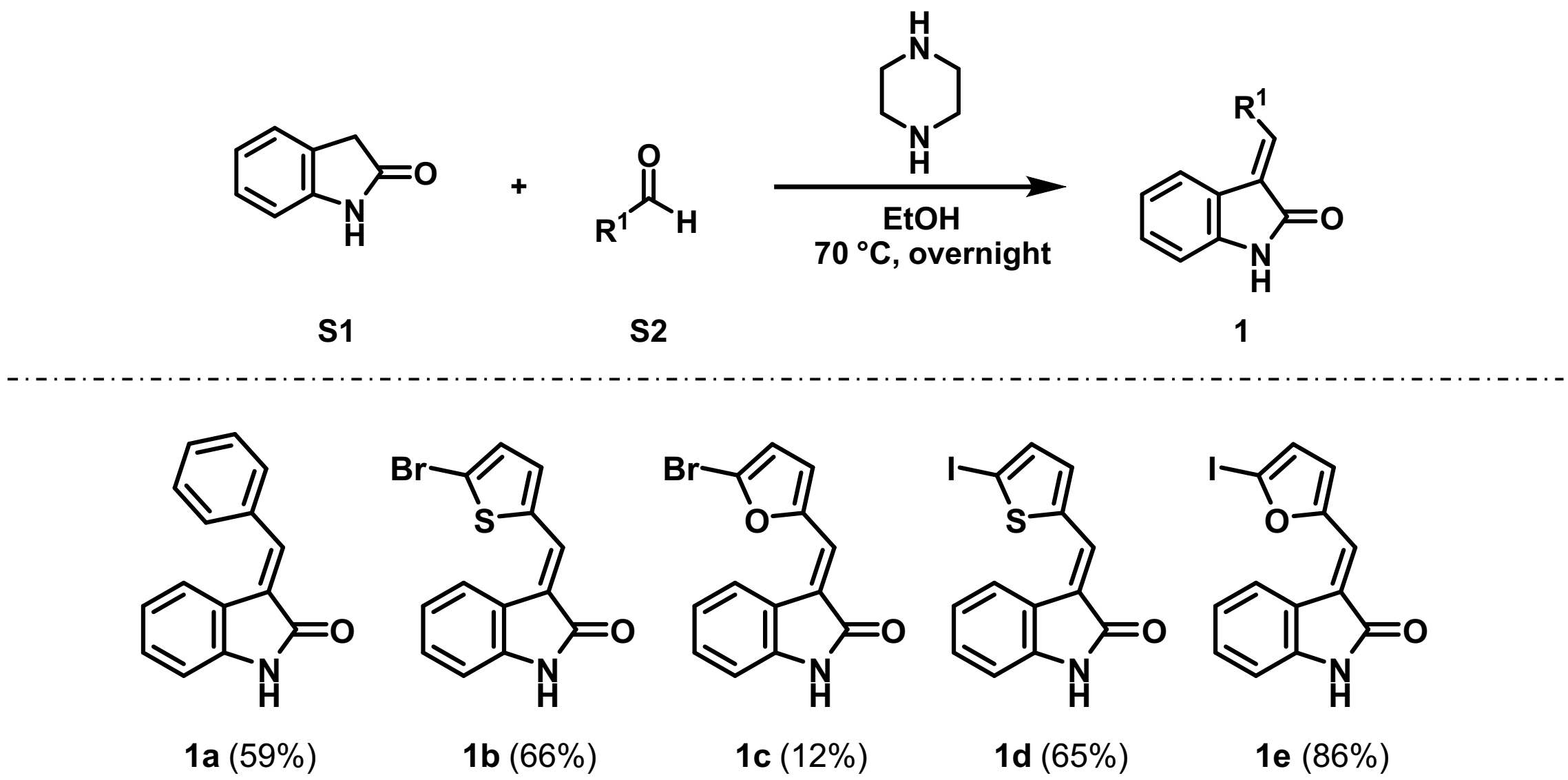

Fig. S4. Synthesis of methyleneindolinones. The desired methyleneindolinones **1a-e** were obtained by reacting oxindole with each heteroaromatic aldehyde in ethanol under basic conditions.

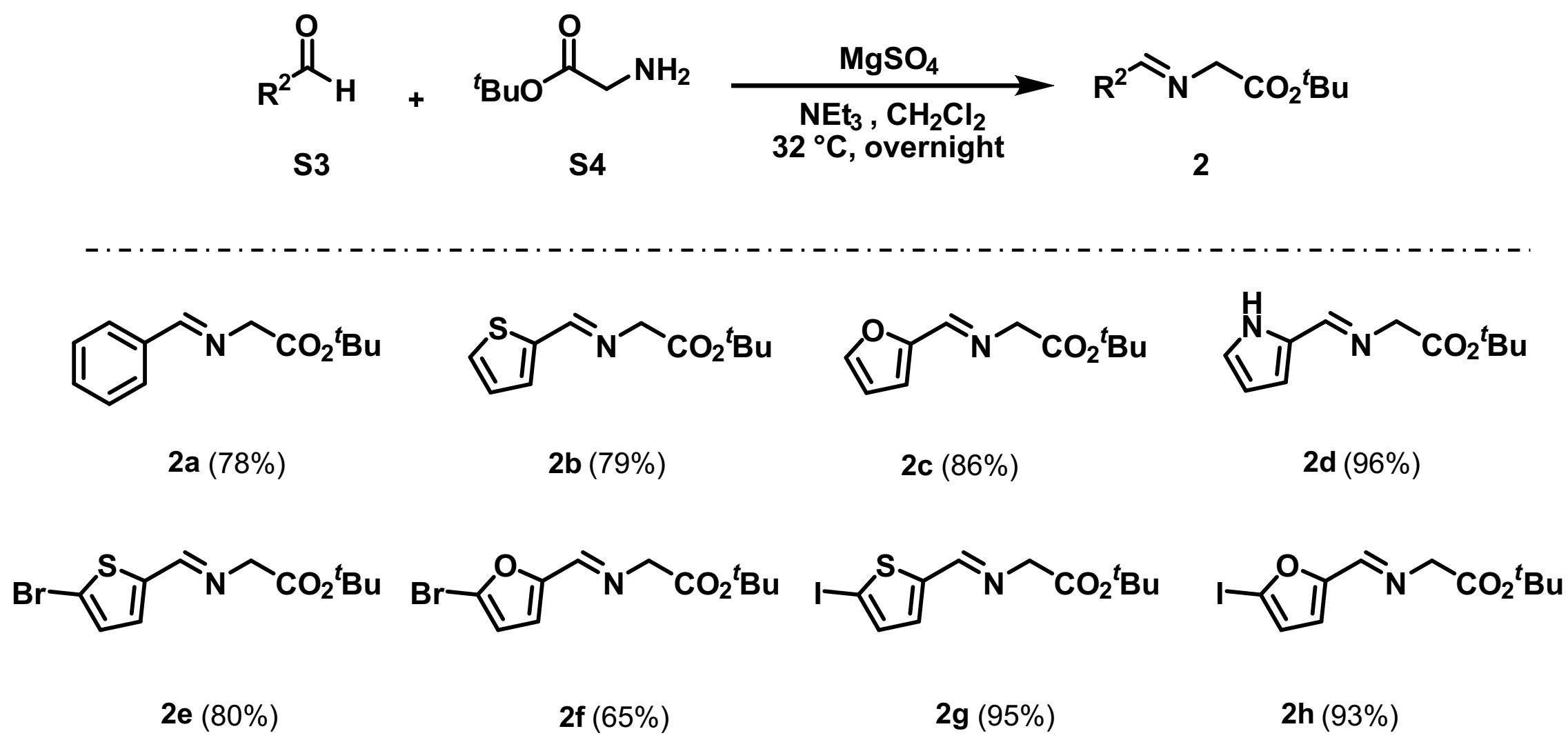

Fig. S5. Synthesis of iminoesters. Iminoesters **2a-h** were obtained by reacting the *tert*-butyl glycine ester with each aromatic aldehyde in dichloromethane under basic conditions.

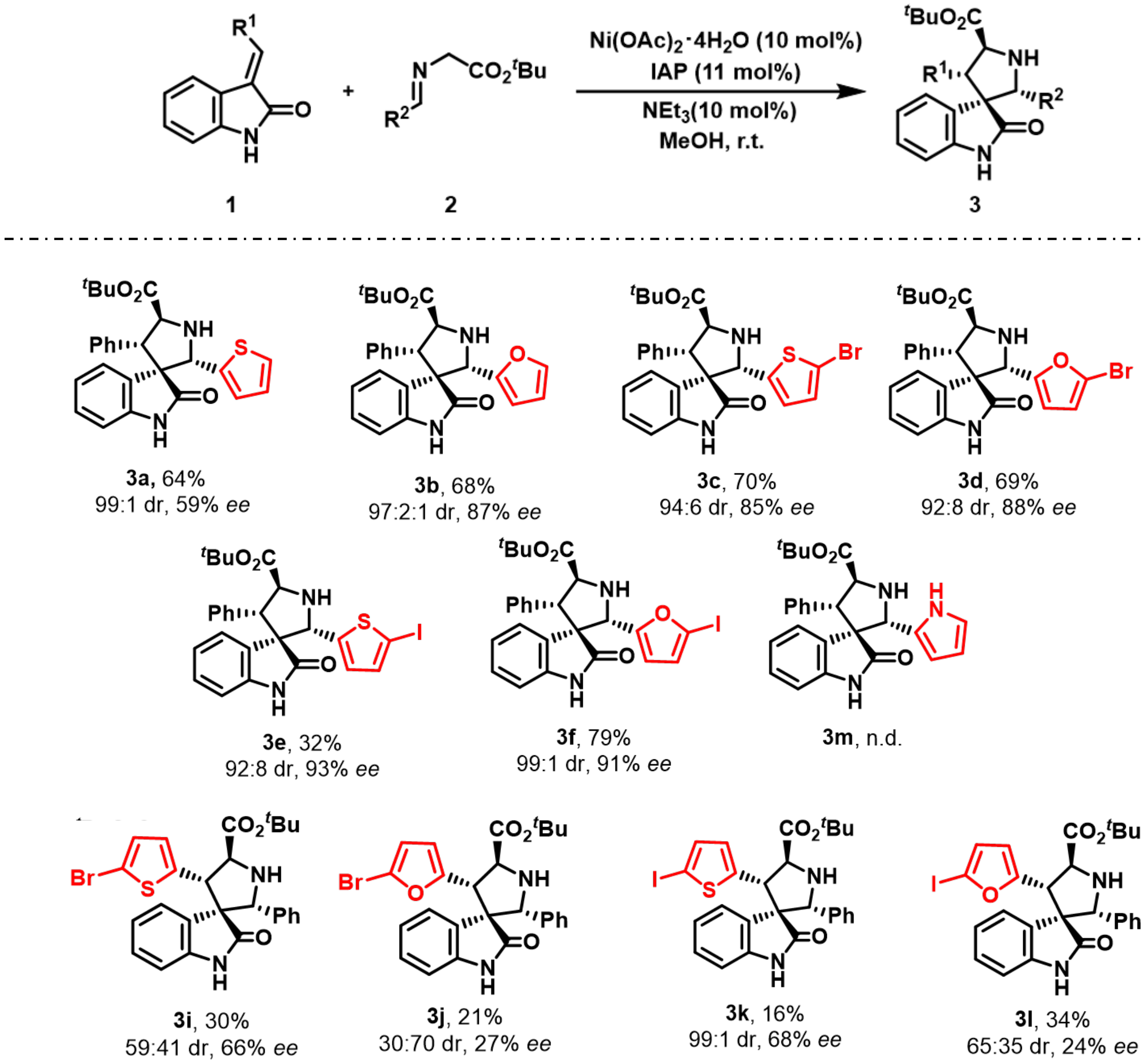

Fig. S6. Synthesis of spirooxindoles. Methyleneindolinones **1a-e** and iminoesters **2a-h** were reacted in methanol under basic conditions in the presence of IAP–nickel catalyst to afford the desired spirooxindoles **3a–i**.

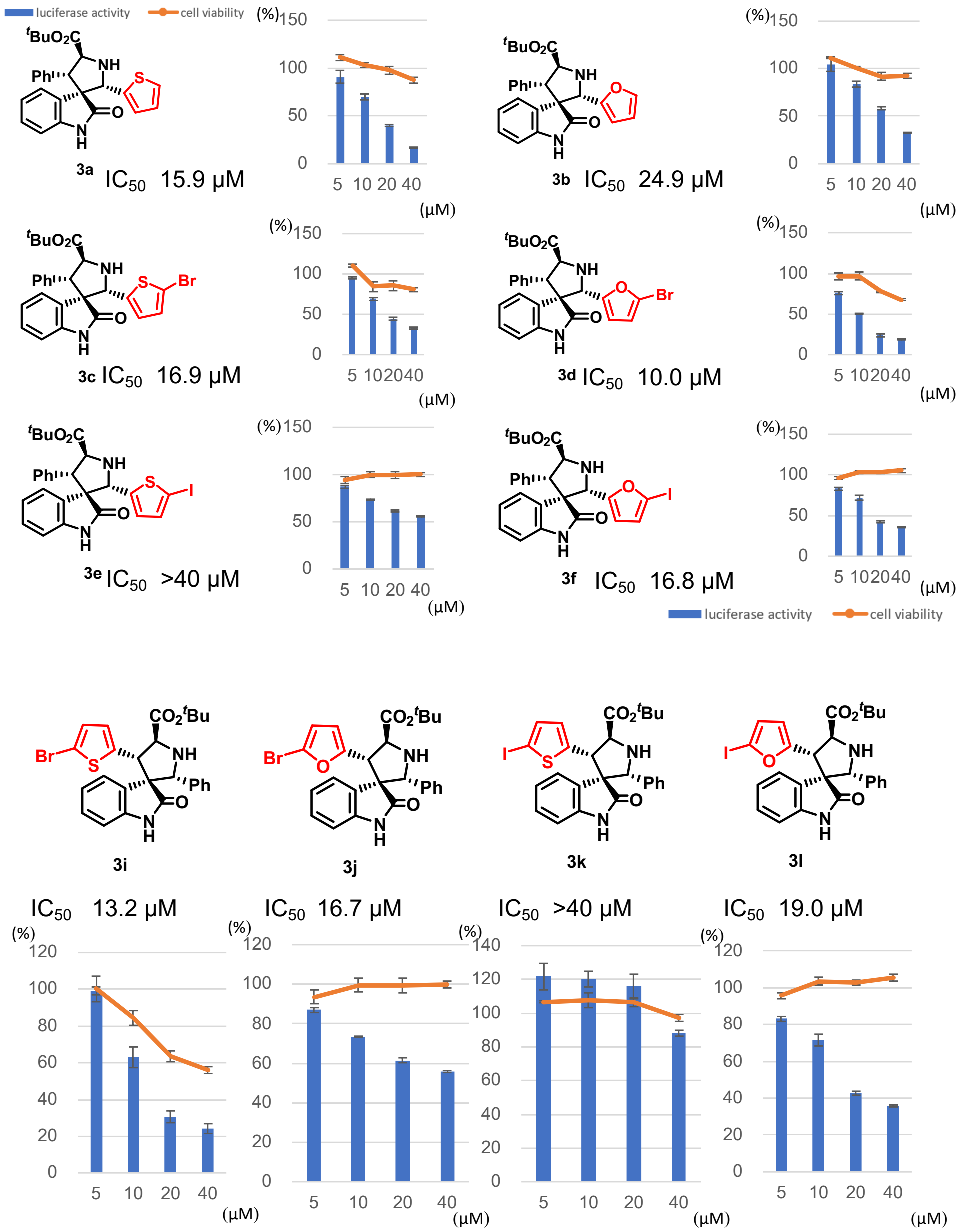

Fig. S7. Evaluation of Notch inhibition by spirooxindoles (1). LS174T reporter cells were seeded in a 96-well plate (2.0×10^4 cells per well) and treated with doxycycline (50 ng/mL) for 12 h to induce Notch1 (1704–2531) expression, followed by incubation with spirooxindoles **3a-i** for 12 h. Luciferase activity was then measured to evaluate Notch signaling inhibition. Cell viability was assessed in parallel using fluorescein diacetate staining under the same treatment conditions (n = 3). All data are shown as mean ± SD.

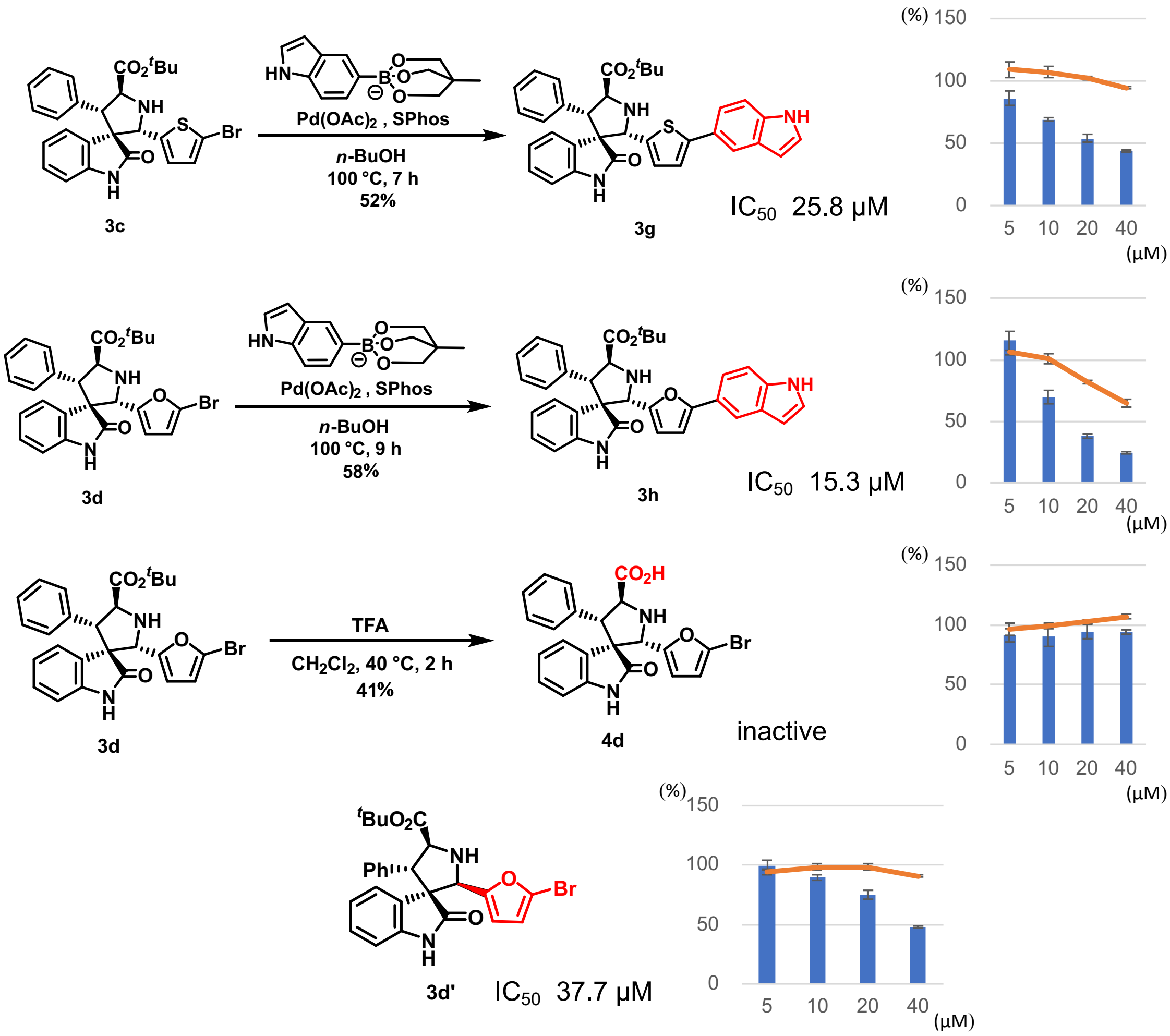

Fig. S8. Evaluation of Notch inhibition by spirooxindoles (2). Derivatives **3g** and **3h**, bearing an additional indole moiety, were obtained from **3c** and **3d** via Suzuki–Miyaura coupling. Furthermore, hydrolysis of the *tert*-butyl group in **3d** yielded derivative **4d**. In addition, diastereomer **3d′** was obtained using Waldman's synthesis [42]. The Notch signaling inhibitory activity of each compound was subsequently evaluated. LS174T reporter cells were seeded in a 96-well plate (2.0×10^4 cells per well) and treated with doxycycline (50 ng/mL) for 12 h to induce Notch1 (1704–2531) expression, followed by incubation with each derivative for 12 h. Luciferase activity was then measured to evaluate Notch signaling inhibition. Cell viability was assessed in parallel by fluorescein diacetate staining under the same treatment conditions (n = 3). All data are shown as mean ± SD.

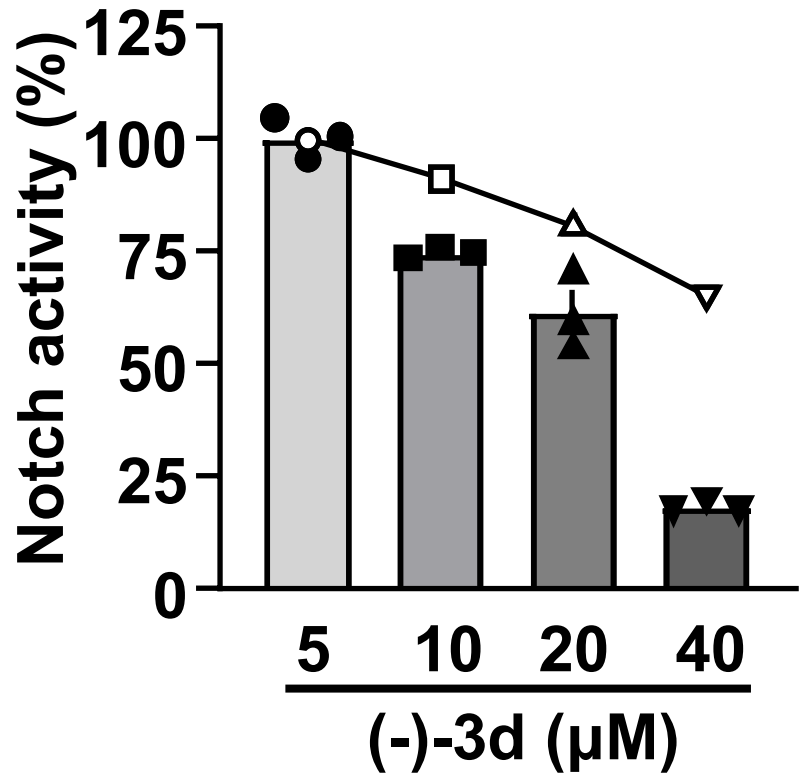

Fig. S9. Evaluation of Notch inhibition by (−)-3d. The Notch signaling inhibitory activity of enantiomers of **3d** obtained by optical resolution was evaluated individually. LS174T reporter cells were seeded in a 96-well plate (2.0×10^4 cells per well) and treated with doxycycline (50 ng/mL) for 12 h to induce Notch1 (1704–2531) expression, followed by incubation with (−)-**3d** for 12 h. Luciferase activity was then measured to evaluate Notch signaling inhibition. Cell viability was assessed in parallel by fluorescein diacetate staining under the same treatment conditions (n = 3). All data are shown as mean ± SEM.

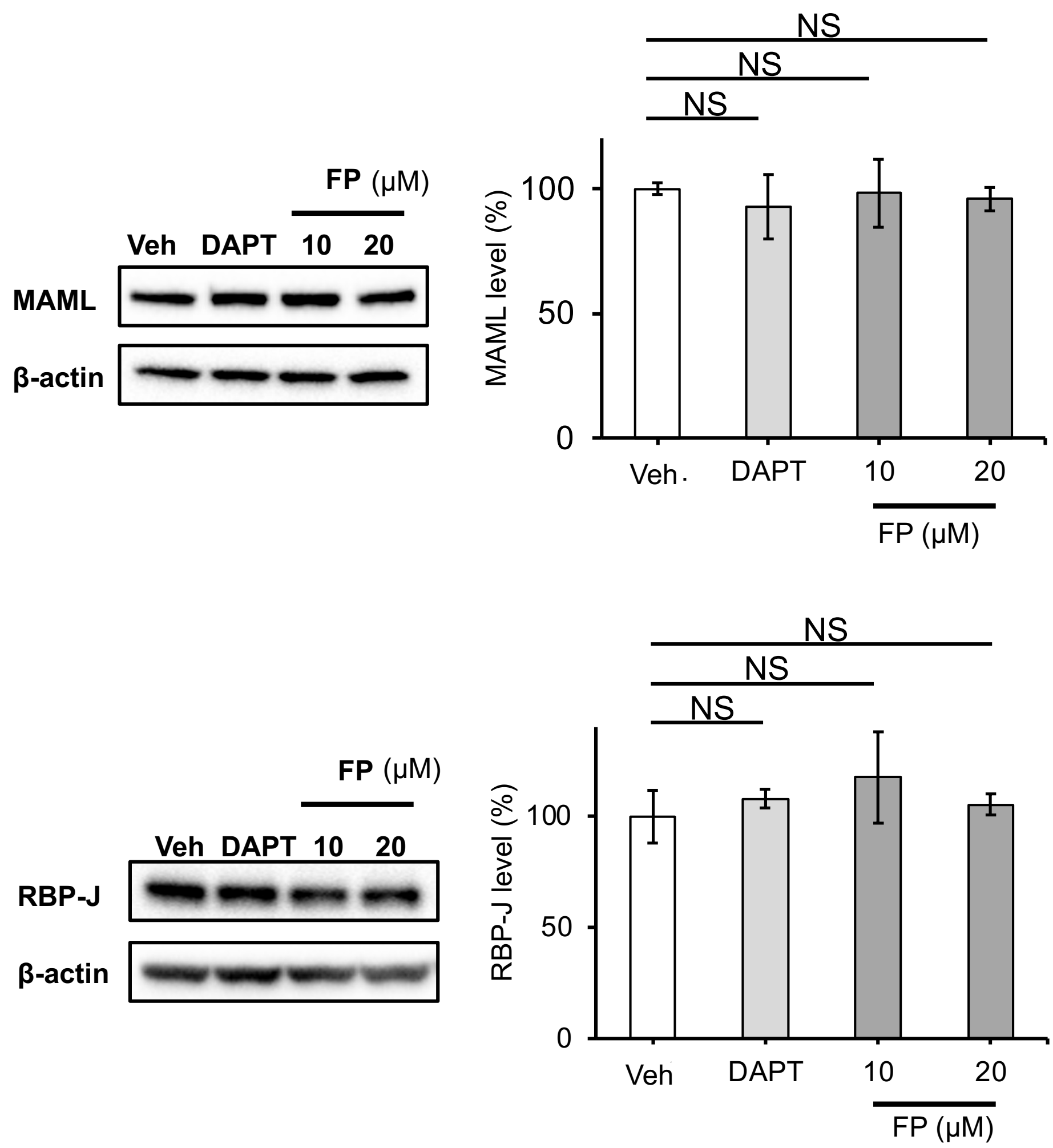

Fig. S10. Measure of MAML and RBP-J level by western blotting. Western blots of Hes1 and ASCL1 in C17.2 cells treated with furapyrrodole (FP, 0, 10, or 20 μM) or DAPT (10 μM) (n = 3). C17.2 cells were treated with FP for 12 h at 37°C. All data are shown as mean ± SD, analyzed using one-way ANOVA followed by Tukey's multiple comparison test.

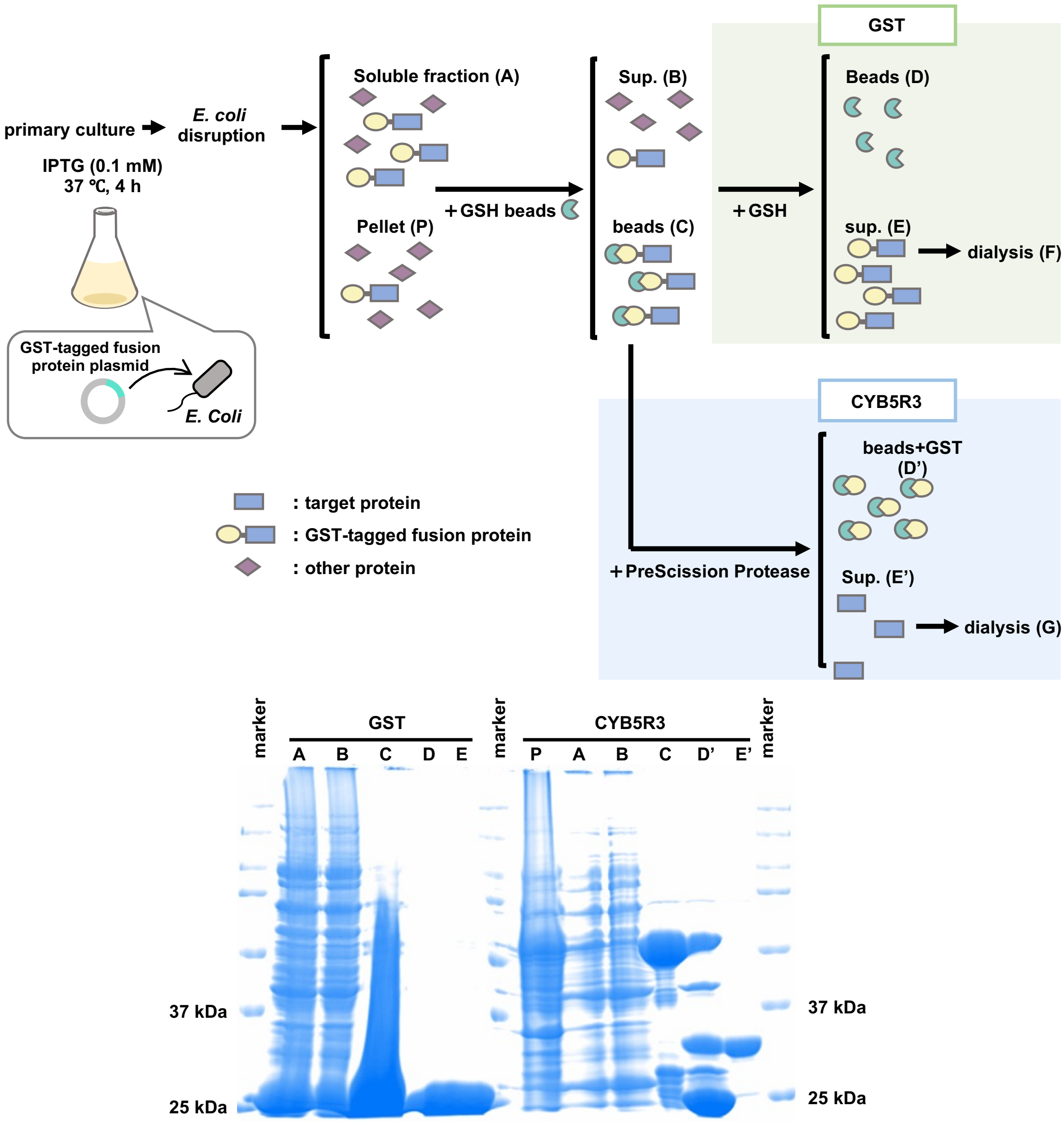

Fig. S11. Confirmation of GST (control) and CYB5R3 expression by Coomassie Brilliant Blue staining. GST–CYB5R3 (ΔN26) was expressed in *E. coli* BL21 cells and purified using glutathione-Sepharose 4B resin. Samples collected during each purification step were analyzed by SDS-PAGE followed by Coomassie Brilliant Blue staining. **(A)** Supernatant after cell lysis. **(B)** Pass-through fraction after affinity binding. **(C)** Bead-bound fraction. **(D)** Pellet after glutathione elution. **(E)** Glutathione eluate containing purified GST. **(D′)** Pellet after PreScission Protease cleavage. **(E′)** Eluate containing cleaved CYB5R3. Each purified recombinant protein was dialyzed against PBS.

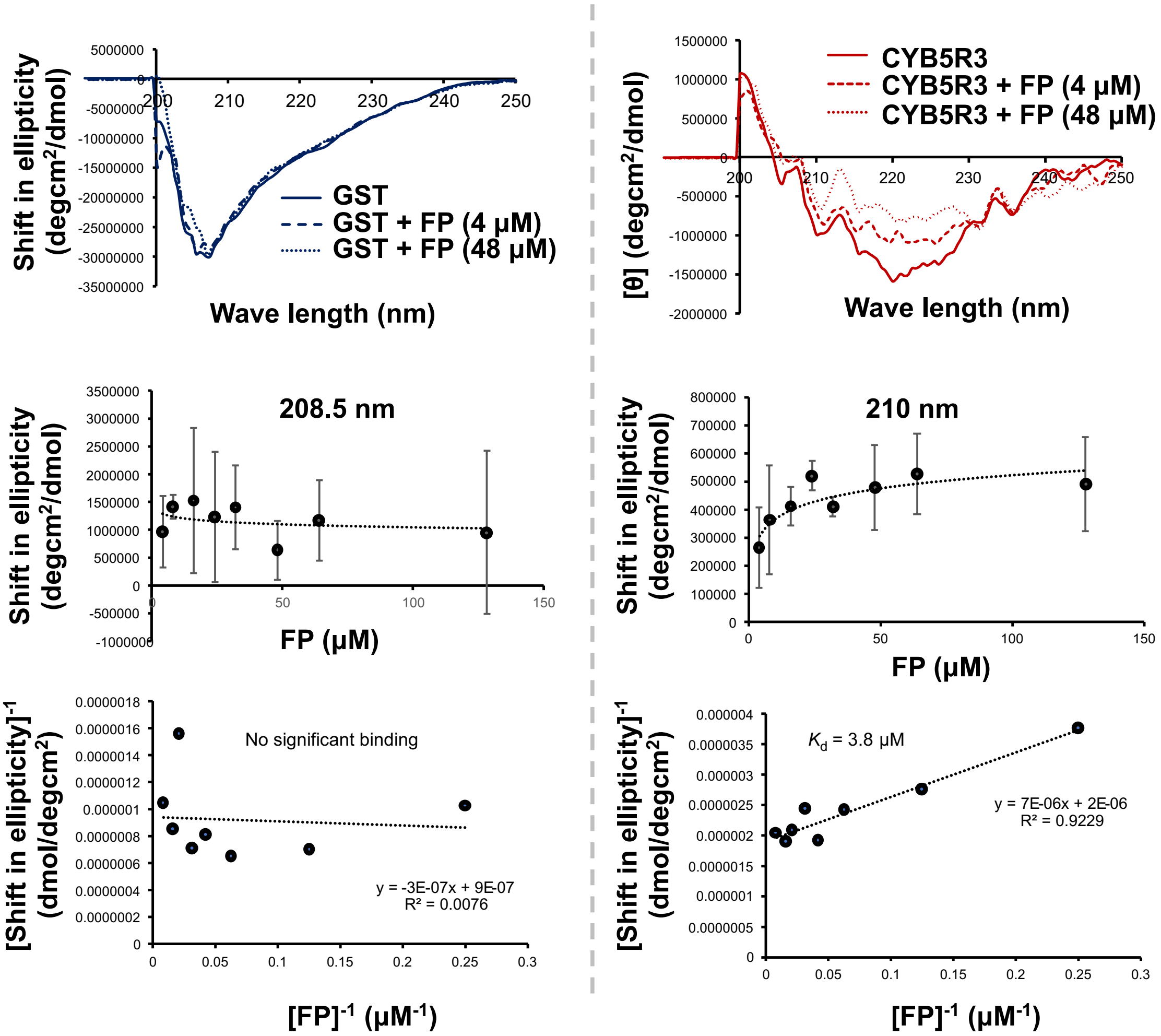

Fig. S12. Calculation of K_d value with the CD spectroscopy. Circular dichroism (CD) spectra were recorded using a J-1500 spectropolarimeter at 24°C (n = 3). Protein samples in PBS were scanned from 200 to 250 nm after sequential addition of FP (4–128 μM). Changes in ellipticity at 208.5 nm (GST) and 210 nm (CYB5R3) were monitored, and dissociation constants (K_d) were calculated using Lineweaver–Burk plots. All data are shown as mean ± SD.

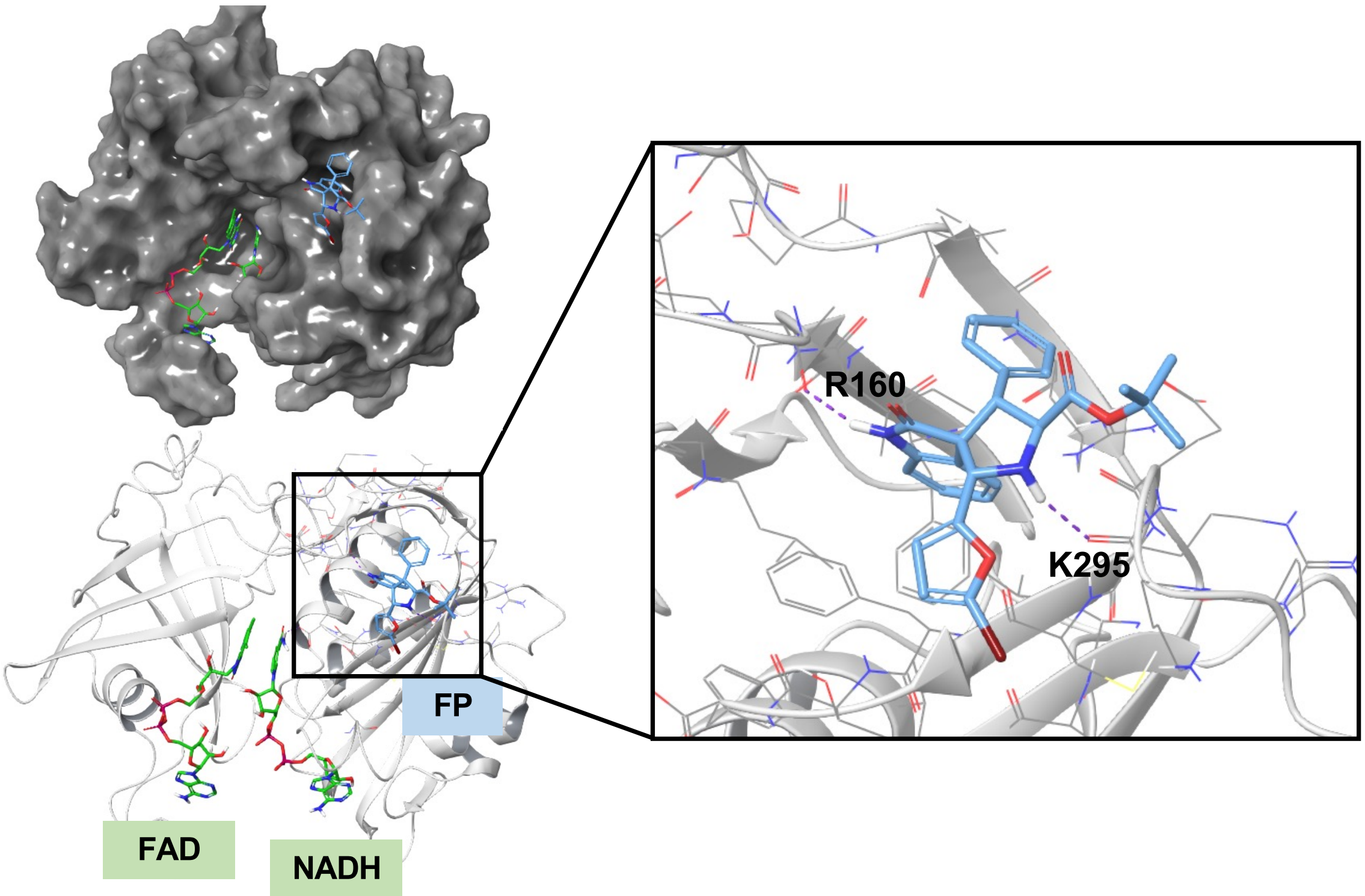

Fig. S13. Prediction of interactions between FP and CYB5R3 by docking simulation. The complex structure of CYB5R3 with FAD and NADH was predicted using AlphaFold3. The mode by which FP binds to the modeled CYB5R3 structure was predicted using Schrödinger Induced Fit Docking. The predicted binding mode of FP revealed interactions with Arg160 and Lys295 of CYB5R3.

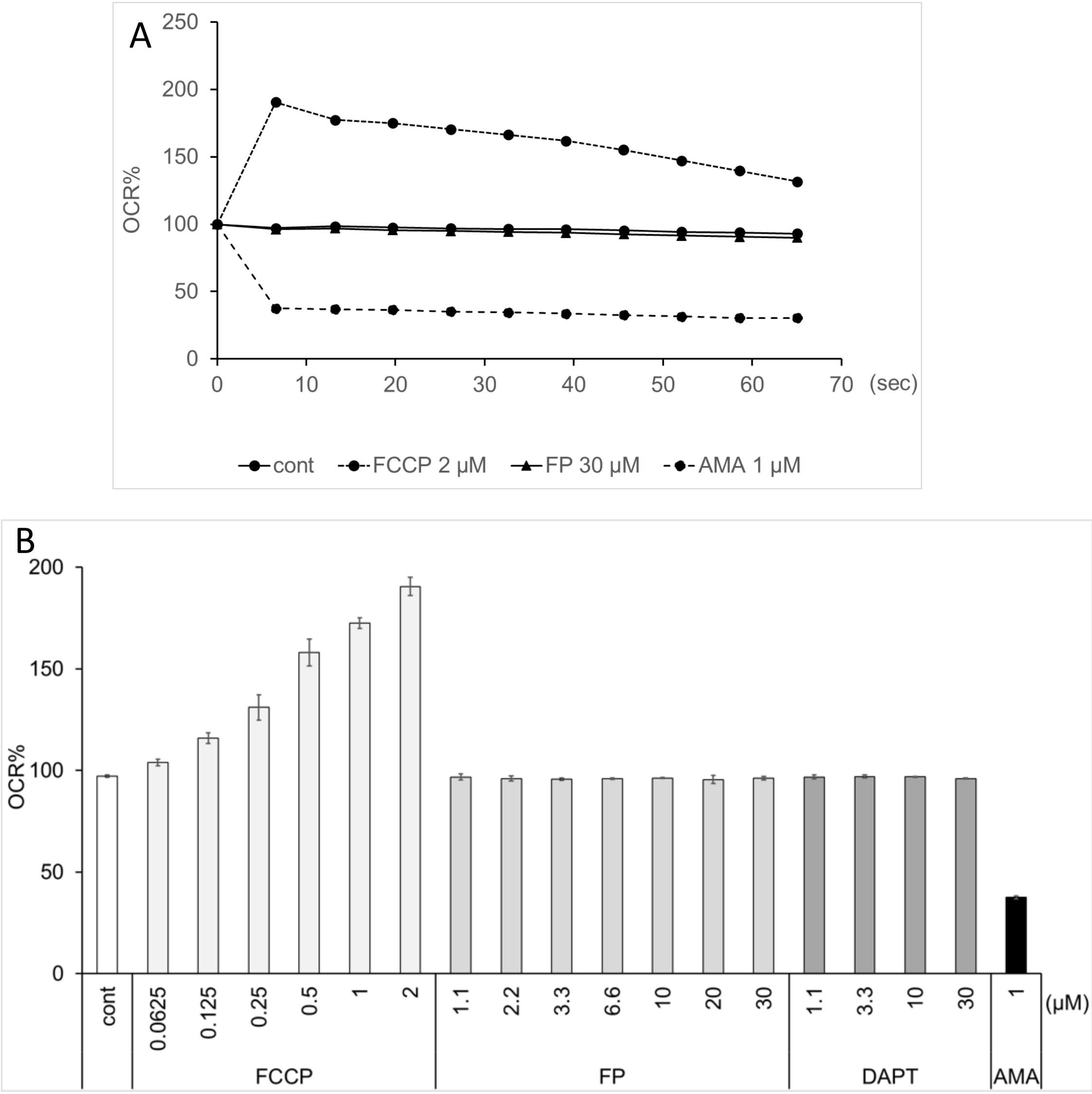

Fig. S14. Measure of OCR level. The oxygen consumption rate [58] was measured after the addition of FP. Positive control (FCCP) and negative control (antimycin; AMA) were used. No significant increase in OCR was detected. **(A)** The OCR level of FCCP (2 μM), FP (30 μM), AMA (1 μM) **(B)** The OCR level of FCCP, FP and DAPT in different concentration. Data are shown as mean ± SD.

Table S1. Detecting target protein of FP by LC-MSMS analysis

Spot No.	No.	Score	emPAI	MW	Uniprot ID	Protein Name
band① (47 kDa)	1	707	0.68	46617	PLIN2_MOUSE	Perilipin-2
	2	31	0.05	59768	TMPSD_MOUSE	Transmembrane protease serine 13
band② (34 kDa)	1	612	1.88	34106	NB5R3_MOUSE	NADH-cytochrome b5 reductase 3
	2	182	0.45	32331	VDAC1_MOUSE	Voltage-dependent anion-selective channel protein 1

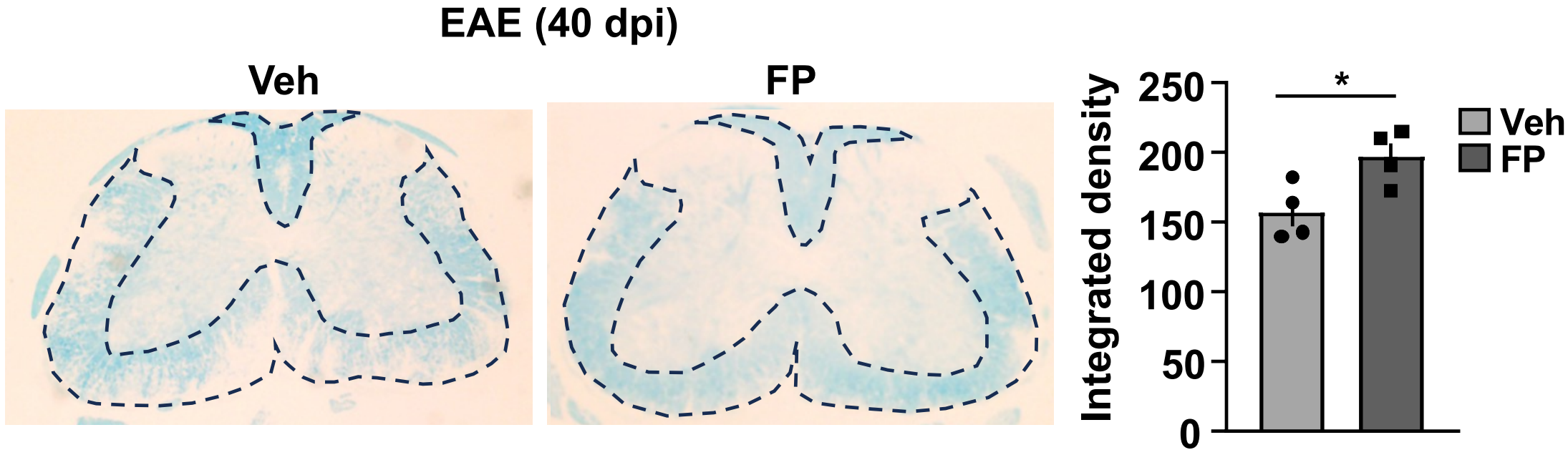

Fig. S15. FP increases myelination in the spinal cord of EAE mice. Myelin status in EAE mice treated with vehicle or FP at 40 dpi was evaluated by LFB staining. Quantification of LFB signals in the white matter (dotted lines) was performed using 4 mice (at least 20 sections/mouse). *$p<0.05$, as determined using Student's *t*-test.

A

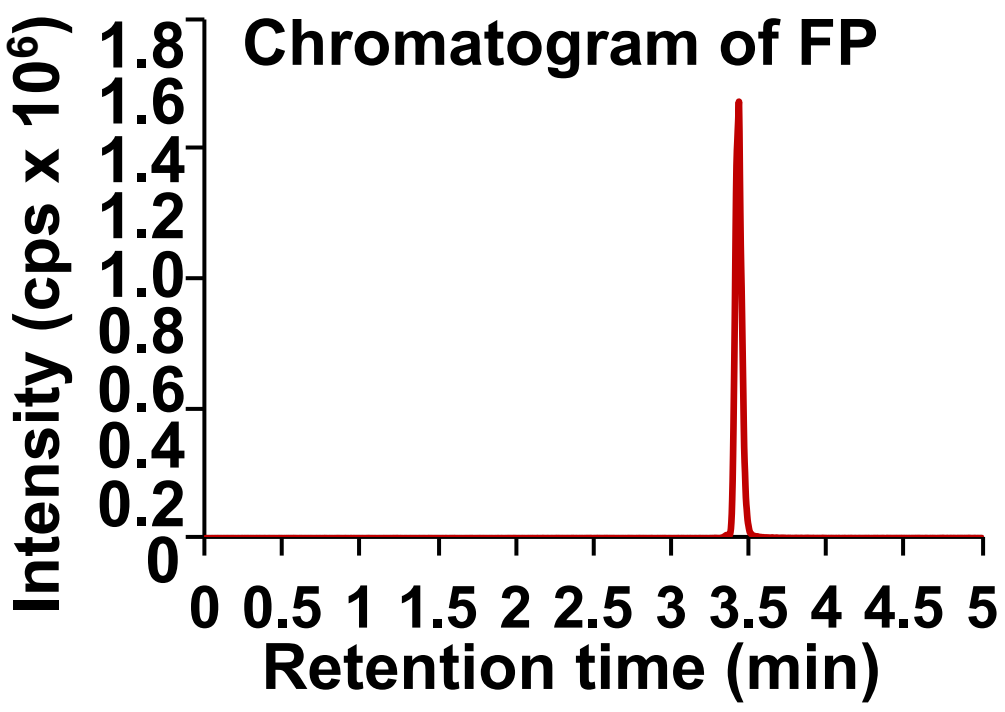

B

Compound	Retention Time (min)	Q1 (m/z)	Q3 (m/z)	Collision Energy (V)
FP	3.43	509.1	453.0	22
CUDA	3.43	341.0	198.0	48

Fig. S16. LC-MS analysis of standard FP.

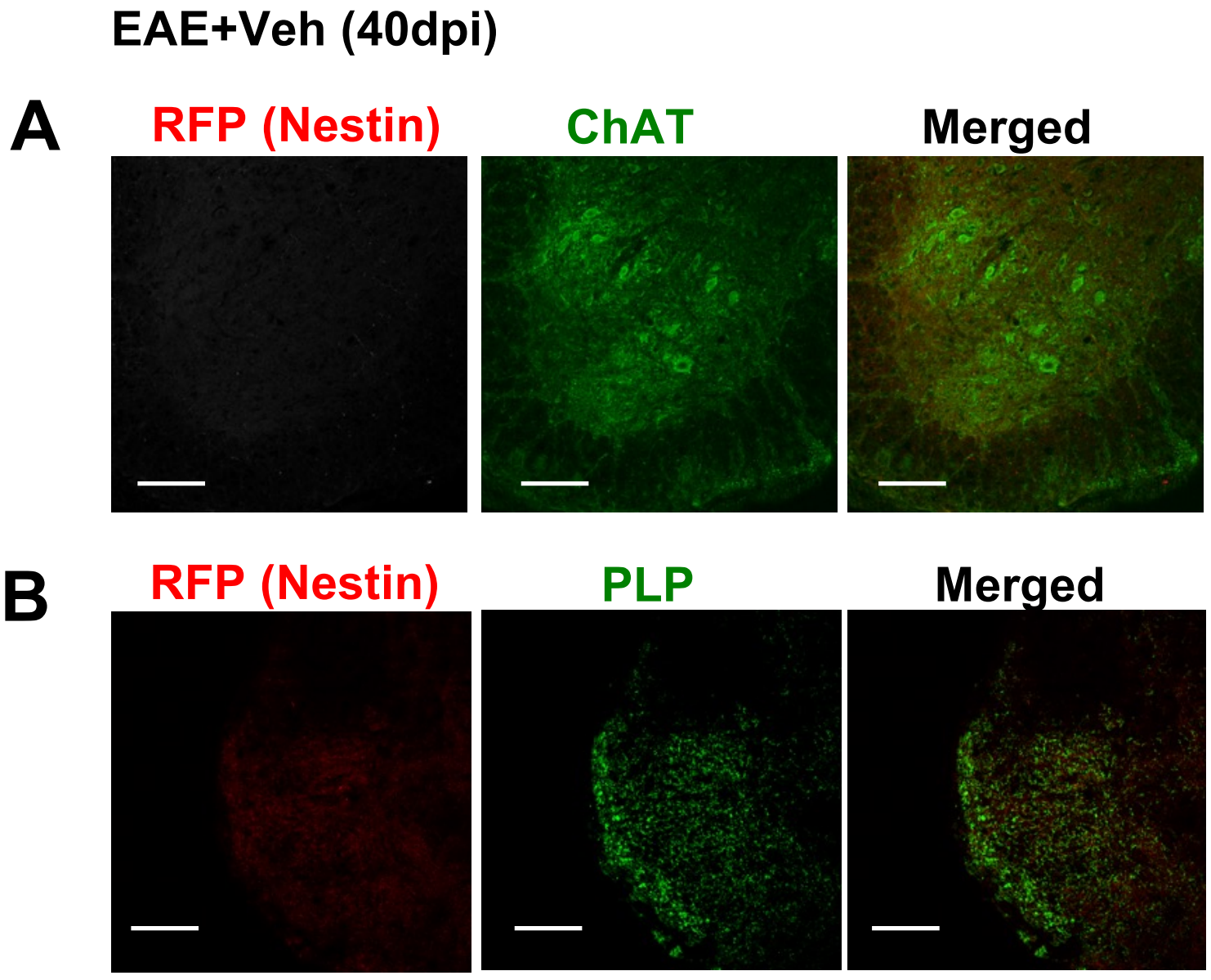

Fig. S17. RFP distribution in the ventral horn and ventral side of the spinal cord in EAE mice with vehicle treatment. Representative images of RFP and ChAT in the ventral horn **(A)** and RFP and PLP in the ventral side **(B)** of the spinal cord in EAE mice treated with vehicle at 40 dpi.

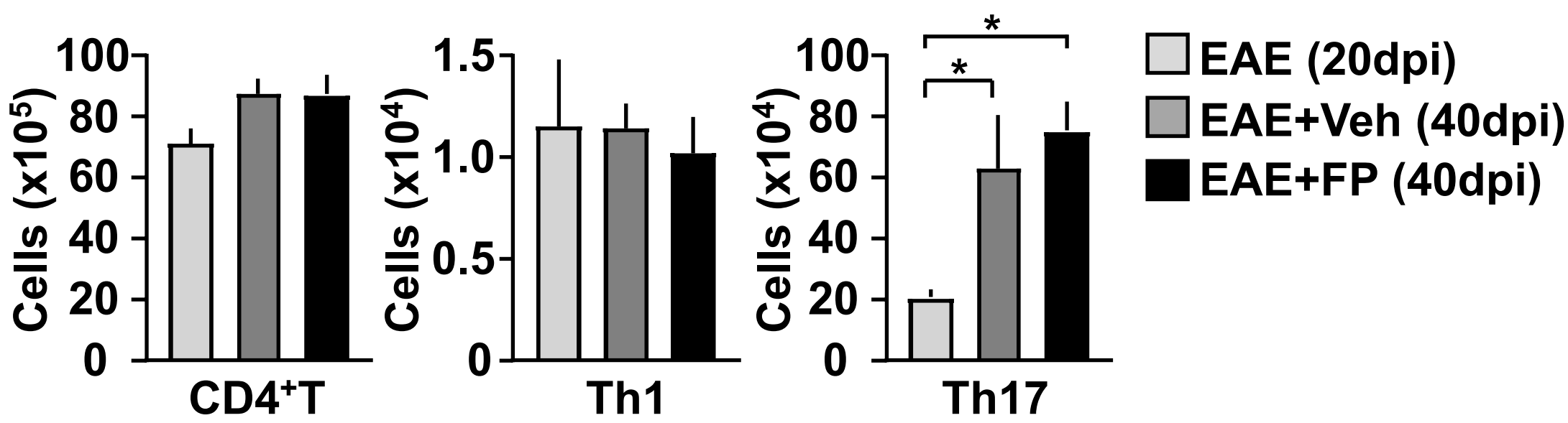

Fig. S18. Flow cytometry analysis of cells in the spleen. Numbers of $CD4^+$ T cells ($CD3^+$, $CD4^+$), Th1 cells ($CD3^+$, $CD4^+$, $IFN\gamma^+$), and Th17 cells ($CD3^+$, $CD4^+$, $IL\text{-}17^+$) in the spleen of EAE at 20 dpi and EAE mice treated with Veh or FP at 40 dpi.

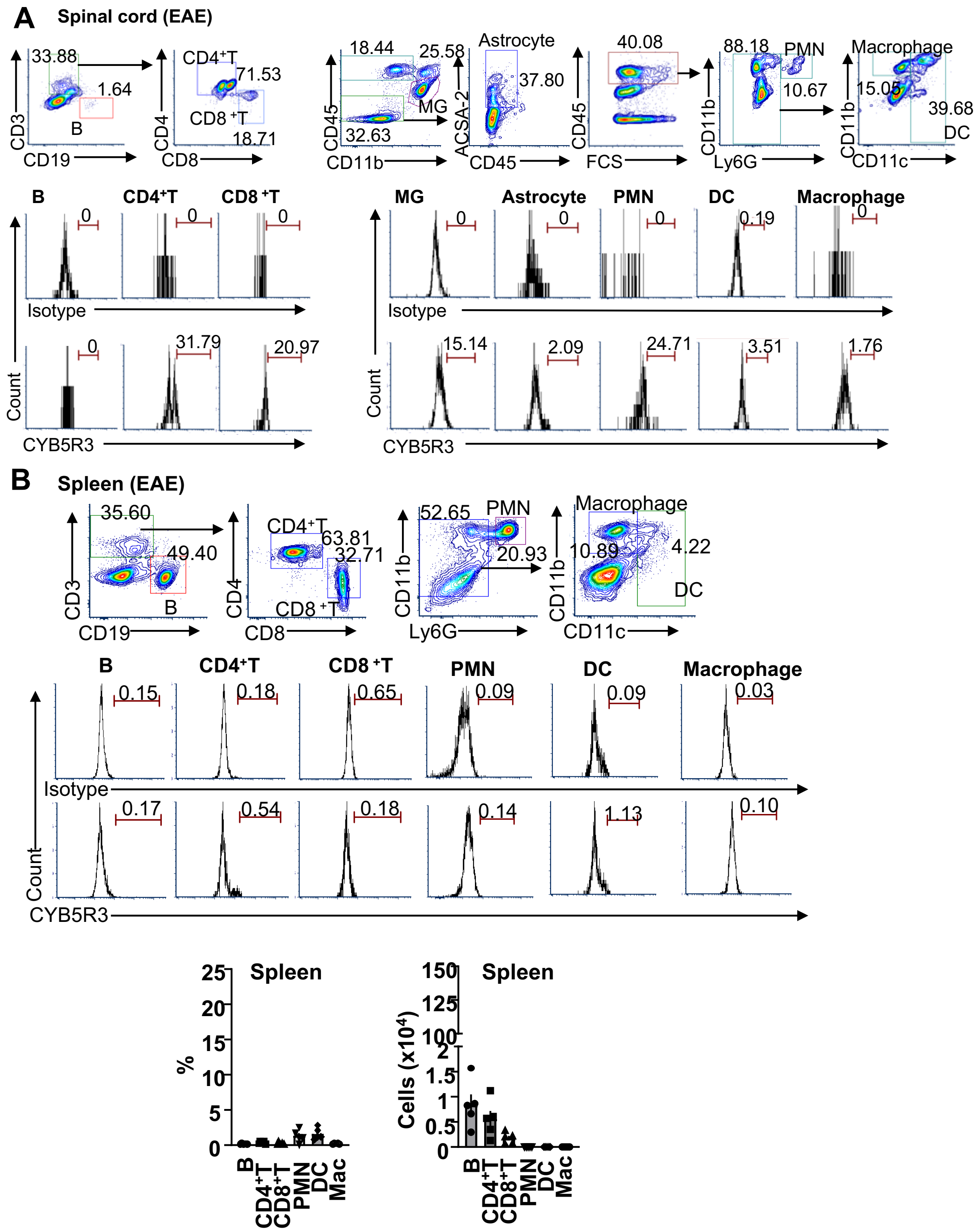

Fig. S19. CYB5R3 expression. The gating strategy in the spinal cord (A) and spleen (B). Frequency and cell number of CYB5R3 in immune cells in the spleen of EAE mice at 20 dpi were indicated in B.

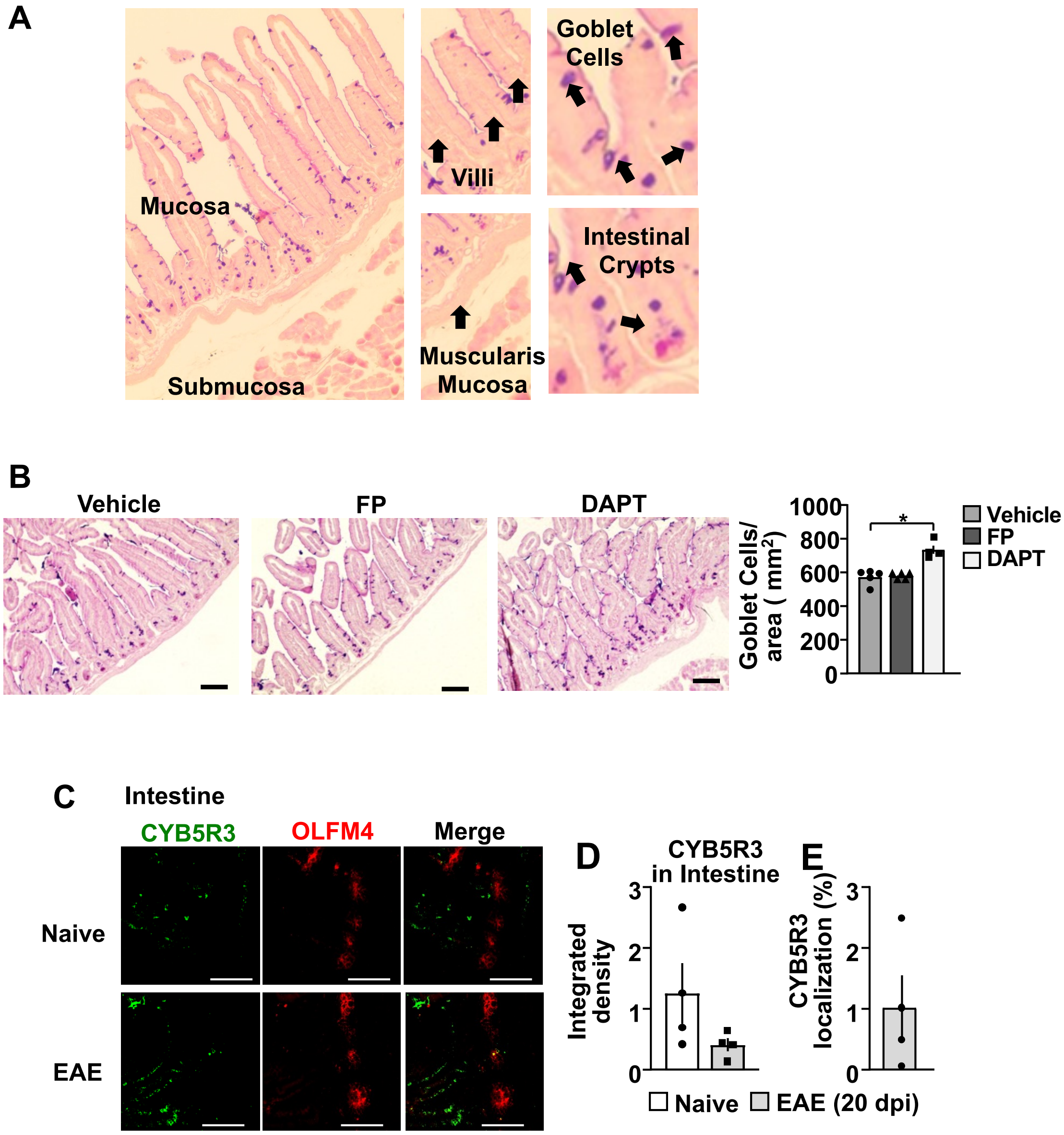

Fig. S20. Furapyrrodole is associated with fewer adverse effects. (A) Representative signals in the intestine. **(B)** Goblet cell staining in the intestine. Intestines were isolated from EAE mice treated with vehicle, DAPT (1 mg/kg, i.p., every other day from 20-28 dpi), or furapyrrodole (FP, 1 mg/kg, i.p., every other day from 20-28 dpi) at 30 dpi (n = 4-5; at least 22 sections/mouse) *$p<0.05$. **(C-E)** Representative images and quantification of CYB5R3 and OLFM4 (stem cell marker) signals in the intestine of naïve and EAE mice at 20 dpi. Expression levels of CYB5R3 in the intestine (C). Frequency of CYB5R3's intestinal stem cell localization measured by the ratio of ORMF4 signal to the CYB5R3 signal in EAE mice (D, E). Bars = 100 µm.

Chemical Synthesis

General experimental procedure

NMR spectra were recorded on JEOL ECZ400, ECZ500 and ECZ600 spectrometers in a deuterated solvent whose chemical shift was taken as an internal standard. Mass spectra were obtained using AccuTOF LC-plus JMS-T100LP (JEOL). IR spectra were measured on ATR on a JASCO FT-IR 230 spectrophotometer. Column chromatography was performed using silica gel PSQ100B (Fuji Silysia Chemical Ltd., Kasugai, Japan) and silica gel 60N (Kanto Chemical Co., Inc., Tokyo, Japan).

Compound **1a-e**

General procedure

To a solution of oxindole (**S1**; 665mg, 5 mmol) in EtOH (2.5 mL) were added furfral (**S2**; 556 mg, 5.5 mmol) and piperadine (430 mg, 5 mmol). The reaction mixture was stirred for 1.5 h at 70 °C and the reaction was cooled in freezer overnight. The reaction mixture was rinsed with EtOH and pump up to afford **1a** (650 mg, 2.9 mmol, 59%) as a yellow solid.

(*E*)-3-Benzylideneindolin-2-one (**1a**); 59%

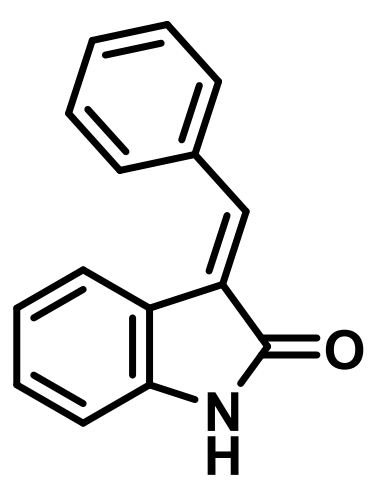

IR (ATR): 1703, 1608, 1464, 805, 781, 746 cm^{-1}.

^{1}H-NMR (400 MHz, $CDCl_3$):8.95 (s, 1H), 7.84 (s, 1H), 6.84-7.67 (m, 9H).

^{13}C-NMR (100 MHz $CDCl_3$): δ 170.2, 141.5, 137.5, 134.7, 129.8, 129.6, 129.3 (2C), 128.6 (2C), 127.4, 123.0, 121.8, 121.6, 110.1.

ESI-HRMS $[M+Na]^+$: calcd for $C_{40}H_{36}NaO_{11}$ 715.2155, found 715.2067.

(*E*)-3-(Bromothiophen-2-ylmethylene)indolin-2-one **(1b)**; 66%

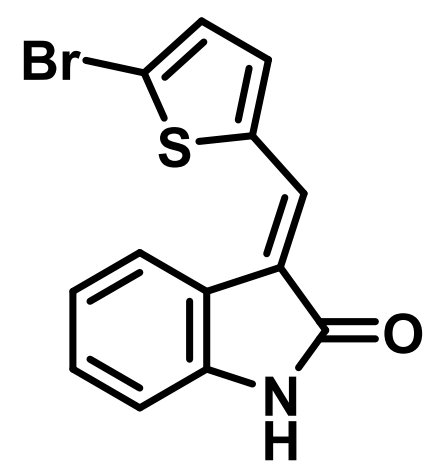

IR (ATR): 1737, 1633, 1431, 1366, 1217, 1147, 801, 770, 745, 698 cm^{-1}.

^{1}H-NMR (400 MHz, $CDCl_3$): δ10.69 (s, 1H), 8.06 (s, 1H), 7.66 (d, *J* = 7.8 Hz, 1H), 7.61 (d, *J* = 4.1 Hz, 1H), 7.36 (d, *J* = 4.1 Hz, 1H), 7.22 (t, *J* = 7.8 Hz, 1H), 7.00 (t, *J* = 7.8 Hz, 1H), 6.86 (d, *J* = 7.8 Hz, 1H).

^{13}C-NMR (100 MHz, $(CD_3)_2SO$): δ 168.06, 141.15, 139.42, 138.53, 130.96, 129.35, 128.00, 124.48, 122.44, 121.69, 121.06, 120.16, 110.21.

ESI-HRMS $[M+H]^+$: calcd for $C_{13}H_9BrNOS$ 305.9588, found 305.9533.

(*E*)-3-(Bromofuran-2-ylmethylene)indolin-2-one **(1c)**; 81%

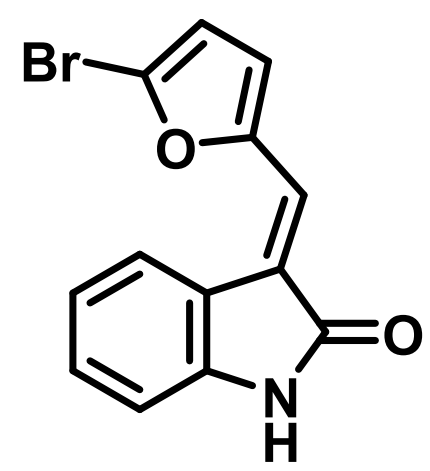

IR (ATR): 1702, 1609, 1469,1139, 778, 743, 671 cm^{-1}.

^{1}H-NMR (400 MHz, $CDCl_3$): δ 8.36 (d, *J* = 7.8 Hz, 1H), 7.68 (br, 1H), 7.36 (s, 1H), 7.31-7.27 (1H), 7.11 (t, *J* = 7.8 Hz, 1H), 6.88 (d, *J* = 7.8 Hz, 1H), 6.85 (d, *J* = 3.2 Hz, 1H), 6.57 (d, *J* = 3.2 Hz, 1H).

^{13}C-NMR (100 MHz, $(CD_3)_2SO$): δ 169.60, 153.15, 143.35, 130.53, 126.99, 124.75, 123.28, 123.08, 121.90, 121.55, 118.67, 116.22, 110.46.

ESI-HRMS $[M+H]^+$: calcd for $C_{13}H_9BrNO_2$ 289.9817, found 289.9853.

(*E*)-3-(Iodothiophene-2-ylmethylene)indolin-2-one **(1d)**; 65%

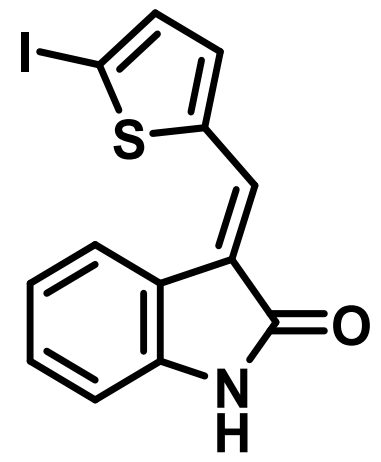

IR (ART): 1754, 1681, 1595, 1464, 1372, 1202, 903, 786, 727, 645, 635, 625 cm^{-1}.

^{1}H-NMR (400 MHz, $(CD_3)_2SO$): δ 10.59 (s, 1H), 8.24 (d, J = 7.8 Hz, 1H), 7.24-7.28 (m, 2H), 7.16 (m, 1H), 7.01-7.04 (m, 2H), 6.87 (d, J = 7.8 Hz, 1H).

^{13}C-NMR (600 MHz, $(CD_3)_2SO$): δ 169.66, 156.03, 143.29, 130.46, 124.80, 124.24, 123.17, 123.08, 121.83, 121.71, 118.56, 110.40, 99.28.

ESI-HRMS $[M+Na]^+$: calcd for $C_{13}H_8I_1N_1Na_1O_1S_1$ 375.9269, found 375.9266.

(*E*)-3-(Iodofuran-2-ylmethylene)indolin-2-one **(1e)**; 86%

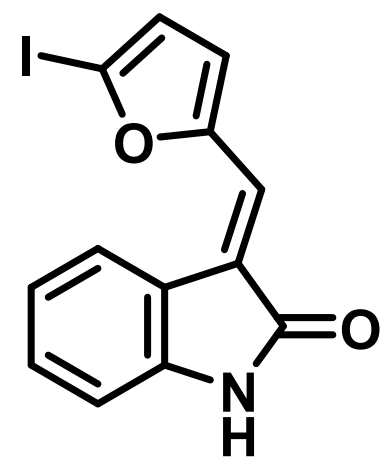

IR (ART): 1705, 1606, 1464, 1348, 1205, 780, 739, 670 cm^{-1}.

^{1}H-NMR (400 MHz, $(CD_3)_2SO$): δ 10.68 (s, 1H), 8.06 (br, 1H), 7.65 (d, J = 7.8 Hz, 1H), 7.45 (m, 2H), 7.19 (t, J = 7.6 Hz, 1H), 6.98 (t, J = 7.6 Hz, 1H), 6.84 (d, J = 7.8 Hz, 1H).

^{13}C-NMR (600 MHz, $(CD_3)_2SO$): δ 168.00, 143.59, 141.12, 139.30, 137.33, 129.27, 127.37, 124.55, 122.42, 121.63, 120.13, 110.14, 89.25.

ESI-HRMS $[M+Na]^+$: calcd for $C_{13}H_8I_1N_1Na_1O_2$ 359.9497, found 359.9415.

Compound **2a-h**

General procedure

To a solution of glycine-^{t}Bu ester (**S4**; 553mg, 3.3 mmol) in CH_2Cl_2 (12 mL) were added $MgSO_4$ (2.7 g, 22.5 mmol) and NEt_3 (0.92 mL, 6.6 mmol). The reaction mixture was stirred for 1 h at room temperature and benzaldehyde (**S3**; 0.3 mL, 3.0 mmol) was added to the reaction mixture. The reaction mixture was stirred for overnight at 32 °C, then the mixture was filtrated and washed with CH_2Cl_2 and H_2O. The mixture was extracted with CH_2Cl_2. The organic layer was washed with brine and dried over Na_2SO_4. After filtration and concentration to afford **2a** (620mg, 2.8 mmol, 94%) as a colorless oil.

tert-Butyl (*E*)-2-(benzylideneamino)acetate **(2a)**; 94%

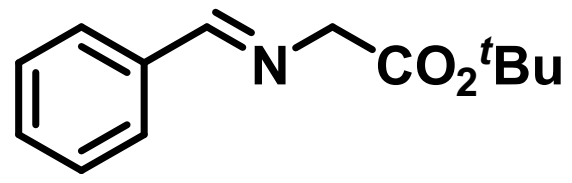

IR (ART): 3748, 1740, 1647, 1152, 882, 790, 773, 761, 751, 715, 690 cm^{-1}.

^{1}H-NMR (400 MHz, $CDCl_3$): δ 8.25 (s, 1H), 7.67 (dd, *J* = 2.4, 7.2Hz, 2H), 7.41 (m, 3H), 4.30 (s, 2H), 1.48 (s, 9H).

^{13}C-NMR (150 MHz, $CDCl_3$): δ 169.3, 165.1, 135.6, 131.0, 128.4 (2C), 128.3 (2C), 81.3, 62.5, 28.0 (3C).

ESI-HRMS $[M+Na]^+$: calcd for $C_{13}H_{17}N_1Na_1O_2$ 242.11570, found 242.11072.

tert-Butyl (*E*)-2-((thiophen-2-ylmethylene)amino)acetate **(2b)**; 79%

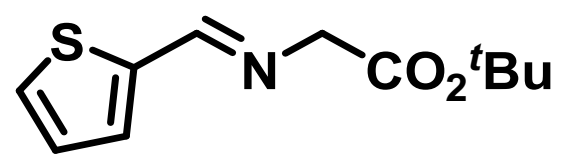

IR (ART): 2977, 2931, 1730, 1636, 1455, 1431, 1392, 1368, 1218, 1151, 1064, 999, 950, 883, 842, 710 cm^{-1}.

^{1}H-NMR (400 MHz, $CDCl_3$): δ 8.14 (s, 1H), 7.26-7.22 (m, 1H), 7.17-7.14 (m, 1H), 6.85 (s, 1H), 4.07 (s, 2H), 1.31 (s, 9H).

^{13}C-NMR (150 MHz, $CDCl_3$): δ 169.1, 158.3, 131.3, 129.5, 127.3, 81.4, 61.9, 28.0 (3C).

ESI-HRMS $[2M+H]^+$: calcd for $C_{22}H_{31}N_2O_4S_2$ 451.17252, found 451.17140.

tert-Butyl (*E*)-2-((furan-2-ylmethylene)amino)acetate **(2c)**; 86%

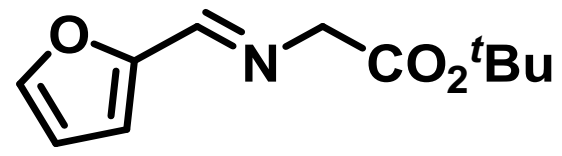

IR (ART): 2145, 1734, 1158, 1009, 851, 774, 761, 751, 719 cm^{-1}.

^{1}H-NMR (400 MHz, $CDCl_3$): δ 7.96 (s, 1H), 7.40 (s, 1H), 6.72 (s, 1H), 6.36 (s, 1H), 4.16 (s, 2H), 1.34 (s, 9H).

^{13}C-NMR (150 MHz, $CDCl_3$): δ 168.7, 153.2, 144.8, 114.6, 111.4, 81.3, 62.7, 27.7 (3C).

ESI-HRMS $[2M+Na]^+$: calcd for $C_{22}H_{30}N_2Na_1O_6$ 441.20016, found 441.20347.

tert-Butyl (*E*)-2-(((1H-pyrrol-2-yl)methylene)amino)acetate **(2d)**; 96%

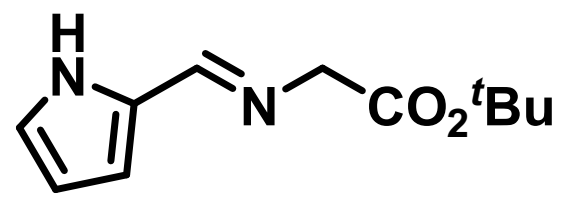

IR (ART): 2976, 1732, 1638, 1368, 1154, 1033 cm^{-1}.

^{1}H-NMR (400 MHz, $CDCl_3$): δ 7.99 (s, 1H), 6.86 (s, 1H), 6.50 (d, *J* = 2.0, 1H), 6.18 (t, 1H), 4.18 (s, 2H), 1.44 (s, 9H).

^{13}C-NMR (600 MHz, $CDCl_3$): δ 169.9, 155.0, 129.7, 122.3, 115.2, 109.8, 81.4, 61.9, 28.0 (3C).

ESI-HRMS $[M+Na]^+$: calcd for $C_{22}H_{33}NaO_4$ 417.25044, found 417.25018.

tert-Butyl(*E*)-2-((5-bromothiophene-2ylmethylene)amino)acetate **(2e)**; 80%

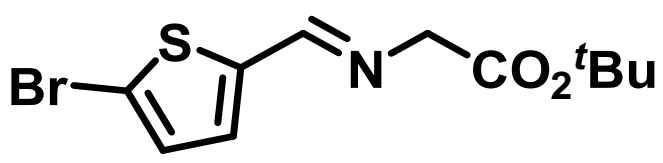

IR (ART): 2978, 2932, 2872, 1734, 1632, 1431, 1392, 1367, 1221, 1147, 1065, 999, 971, 918, 844, 795 cm^{-1}.

^{1}H-NMR (400 MHz, $CDCl_3$): δ 8.22 (s, 1H), 7.08 (d, *J* = 4.1 Hz, 1H), 7.03 (d, *J* = 4.1 Hz, 1H), 4.23 (s, 2H), 1.48 (s, 9H).

^{13}C-NMR (600 MHz, $CDCl_3$): δ 168.99, 157.55, 143.55, 131.54, 130.46, 117.90, 81.65, 61.90, 28.15 (3C).

ESI-HRMS $[M+H]^+$: calcd for $C_{11}H_{15}Br_1N_1O_2S_1$ 304.0007, found 304.0067.

tert-Butyl(*E*)-2-((5-bromofuran-2-ylmethylene)amino)acetate **(2f)**; 65%

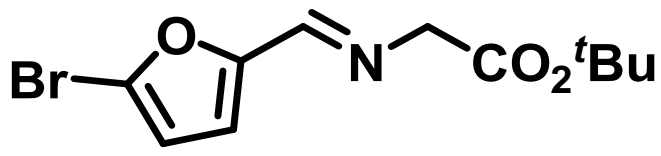

IR (ART): 2940, 1738, 1642, 1480, 1367, 1156, 774,719 cm^{-1}.

^{1}H-NMR (400 MHz, $CDCl_3$): δ 7.91 (s, 1H), 6.73 (d, *J* = 3.7 Hz, 1H), 6.36 (d, *J* = 3.7 Hz, 1H), 4.20 (s, 2H), 1.42 (s, 9H).

^{13}C-NMR (600 MHz, $CDCl_3$): δ 168.88, 153.03, 152.50, 126.34, 117.01, 113.79, 81.65, 62.22, 28.09(3C).

ESI-HRMS $[M+H]^+$: calcd for $C_{11}H_{15}Br_1N_1O_3$ 288.0235, found 288.0252.

tert-Butyl(*E*)-2-((5-Iodothiophene-2-ylmethylene)amino)acetate **(2g)**; 95%

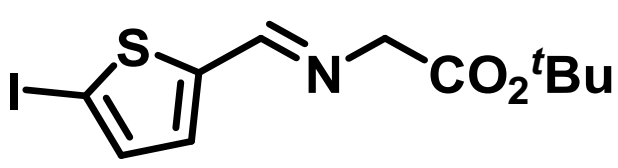

^{1}H-NMR (400 MHz, $CDCl_3$): δ 8.25 (s, 1H), 7.23 (d, *J* = 3.7 Hz, 1H), 6.98 (d, *J* = 3.7 Hz, 1H), 4.24 (s, 2H), 1.48 (s, 9H).

^{13}C-NMR (600 MHz, $CDCl_3$): δ 168.98, 157.22, 147.78, 137.44, 132.56, 81.60, 80.36, 61.94, 28.19(3C).

tert-Butyl (*E*)-2-((5-Iodofuran-2-ylmethylene)amino)acetate **(2h)**; 93%

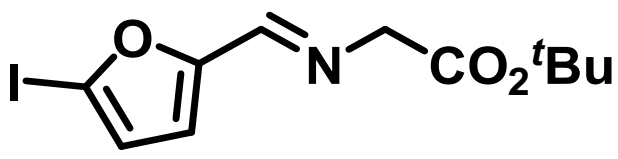

IR (ART): 2977, 1729, 1644, 1459, 1367, 1148, 912, 843, 788 cm^{-1}.

^{1}H-NMR (400 MHz, $CDCl_3$): δ 8.61 (s, 1H), 8.06 (d, *J*= 6.0 Hz, 1H), 7.52 (d, *J*= 7.6 Hz, 1H), 7.30 (t, *J*= 6.8 Hz, 1H), 7.23 (t, *J*= 7.6 Hz, 1H), 4.33 (s, 2H), 1.47 (s, 9H).

^{13}C-NMR (600 MHz, $CDCl_3$): δ 169.0, 164.0, 134.0, 132.9, 132.2, 129.0, 127.5, 125.2, 81.5, 62.6, 28.0 (3C).

ESI-HRMS $[M+H]^+$: calcd for $C_{11}H_{15}I_1N_1O_3$ 336.0097, found 336.0073.

Compound **3a-f, 3i-l**

General procedure

A solution of IAP (12.7 mg, 0.0165 mmol) and $Ni(OAc)_2 \cdot 4H_2O$ (3.7 mg, 0.015 mmol) in MeOH (0.75 mL) were stirred for 2 h at room temperature. To the reaction mixture were added subsequently iminoester (**2b**; 33.8 mg, 0.15 mmol) and DIPEA (2.1 µL, 0.15 mmol) at room temperature, then methyleneindolinone (**1a**; 33.2 mg, 0.15 mmol) was added at 0 °C. After being stirred for appropriate time, the reaction mixture was quenched by water. The organic layer was dried over Na_2SO_4. After filtration and concentration, the resulting residue was purified by silicagel column chromatography (hexane/EtOAc = 4/1) to afford **3a** (34.2 mg, 0.07 mmol, 51%) as a yellow amorphous solid.

tert-Butyl(2'*S*,3*R*,4'*S*,5'*S*)-2-oxo-4'-phenyl-2'-(thiophen-2-yl)spiro[indoline-3,3'pyrrolidine]-5'-carboxylate (**3a**); 64%

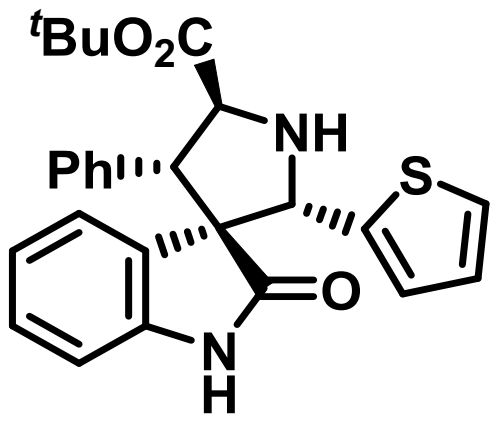

IR (ART): 2977, 1708, 1619, 1598, 1498, 1470,1393, 1368, 1324, 1243, 1158, 844, 756, 697 cm^{-1}.

^{1}H-NMR (400 MHz, $CDCl_3$): δ 8.22 (d, *J*= 7.6, 1H), 7.90 (s, 1H), 7.83 (d, *J*= 3.6, 1H), 7.71 (s, 1H), 7.63-7.60 (m, 3H), 7.49 (d, *J*= 7.2, 1H), 7.02-7.05 (m, 4H), 6.89 (t, *J*= 7.6 Hz, 2H), 5.52 (s, 1H), 4.84 (d, *J*= 7.2 Hz, 1H), 3.94 (d, *J*= 7.2 Hz, 1H), 1.55 (s, 9H).

^{13}C-NMR (150 MHz, $CDCl_3$): δ 173.0, 140.2, 135.1, 128.4 (2C), 128.3, 127.7 (2C), 127.7, 127.2, 126.3, 126.2, 124.4, 124.0, 121.8, 109.4, 81.9, 65.9, 65.3, 62.3, 58.8, 27.8 (3C).

ESI-HRMS $[M+Na]^+$: calcd for $C_{26}H_{26}N_2Na_1O_3S_1$ 469.15618, found 469.15054.

Enantiometric excess was determined by HPLC with a Chiralpack OD-H column (90:10 hexane: 2-propanol, 0.7 mL/min, 254 nm); major enantiomer t_r = 9.5 min, minor enantiomer t_r = 7.1 min; 59% ee

tert-Butyl (2'*S*,3*R*,4'*S*,5'*S*)-2'-(furan-2-yl)-2-oxo-4'-phenylspiro [indoline-3,3'-pyrrolidine]-5'-carboxylate **(3b)**; 68%

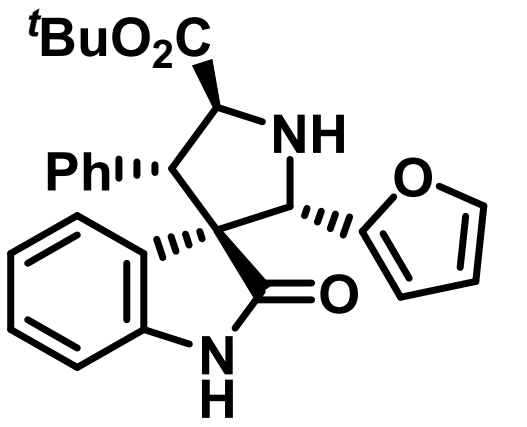

IR (ART): 3241, 2977, 2929, 1708, 1618, 1598, 1499, 1470, 1393, 1366, 1329, 1242, 1220, 1186, 1148, 1076, 1012, 1000, 946, 842 cm^{-1}.

^{1}H-NMR (400 MHz, $CDCl_3$) δ 7.55 (d, *J* = 7.3 Hz, 1H), 7.15 (br, 1H), 7.11-6.98 (7H), 6.94 (t, *J* = 7.5 Hz, 1H), 6.50 (d, *J* = 7.3 Hz, 1H), 6.08 (m, 2H), 5.23 (s, 1H), 4.52 (d, *J* = 10.5 Hz, 1H), 4.06 (d, *J* = 10.5 Hz, 1H), 3.18 (br, 1H), 1.23 (s, 9H).

^{13}C-NMR (150 MHz, $CDCl_3$): δ178.51, 171.65, 151.60, 142.16, 140.75, 137.41, 129.00, 128.17(2C), 127.38(2C), 125.06, 122.11, 110.33, 109.45, 107.32, 81.93, 66.11, 65.82, 60.49, 57.67, 27.96(3C), 14.28.

ESI-HRMS $[M+Na]^+$: calcd for $C_{26}H_{26}N_2Na_1O_4$ 453.17903, found 453.17262.

Enantiometric excess was determined by HPLC with a Chiralpack OD-H column (90:10 hexane: 2-propanol, 0.7 mL/min, 254 nm); major enantiomer t_r = 9.4 min, minor enantiomer t_r = 7.5 min; 87% ee.

tert-Butyl (2'*S*,3*R*,4'*S*,5'*S*)-2-oxo-4'-phenyl-2'-(5-bromothiophen-2-yl)spiro [indoline-3,3'-pyrrolidine]-5'-carboxylate **(3c)**; 70%

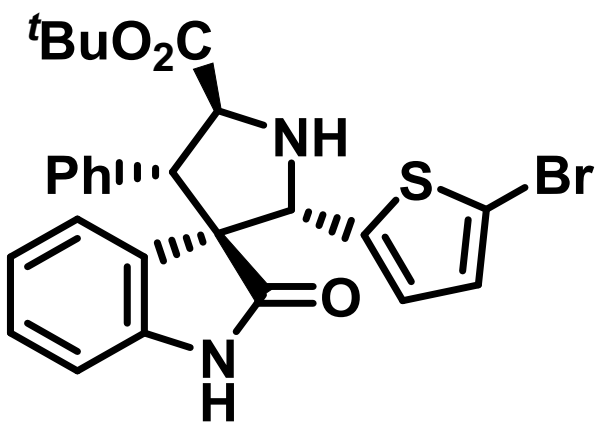

IR (ART) 2976, 1708, 1620, 1471, 1368, 1329, 1243, 1153, 1019, 968, 841, 794, 746, 697 cm^{-1}.

^{1}H-NMR (400 MHz, $CDCl_3$): δ 8.22 (d, *J*= 7.6, 1H), 7.90 (s, 1H), 7.83 (d, *J*= 3.6, 1H), 7.71 (s, 1H), 7.63-7.60 (m, 3H), 7.49 (d, *J*= 7.2, 1H), 7.02-7.05 (m, 4H), 6.89 (t, *J*= 7.6 Hz, 2H), 5.52 (s, 1H), 4.84 (d, *J*= 7.2 Hz, 1H), 3.94 (d, *J*= 7.2 Hz, 1H), 1.55 (s, 9H).

^{13}C-NMR (600 MHz, $CDCl_3$): δ 173.0, 140.2, 135.1, 128.4 (2C), 128.3, 127.7 (2C), 127.7, 127.2,

126.3, 126.2, 124.4, 124.0, 121.8, 109.4, 81.9, 65.9, 65.3, 62.3, 58.8, 27.8 (3C).

ESI-HRMS $[M+Na]^+$: calcd for $C_{26}H_{25}Br_1N_2Na_1O_3S_1$ 547.06670, found 547.06882.

Enantiometric excess was determined by HPLC with a Chiralpack OD-H column (90:10 hexane: 2-propanol, 0.7 mL/min, 254 nm); major enantiomer t_r =12.9 min, minor enantiomer t_r = 7.3 min; 85% ee.

tert-Butyl (2'*S*,3*R*,4'*S*,5'*S*)-2-oxo-4'-phenyl-2'-(5-bromofuran-2-yl)spiro [indoline-3,3'-pyrrolidine]-5'-carboxylate **(3d)**; 69%

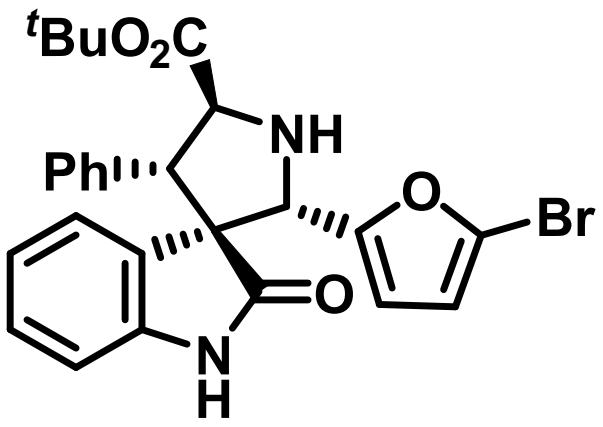

IR(ATR): 3217.65, 2977.55, 1718.26, 1617.98, 1466.6, 1330.64, 1244.83, 1159.01, 1013.41, 957.484, 842.74.

^{1}H-NMR (400 MHz, $CDCl_3$) δ 7.53 (d, J = 7.5 Hz, 1H), 7.47 (br, 1H), 6.94-7.05 (m, 7H), 6.55 (d, J = 7.5 Hz, 1H), 6.04 (d, J = 3.4 Hz, 1H), 5.95 (d, J = 3.4 Hz, 1H), 5.16 (s, 1H), 4.50 (d, J = 11.0 Hz, 1H), 4.03 (d, J = 11.0 Hz, 1H), 1.23 (s, 9H).

^{13}C-NMR (600 MHz, $CDCl_3$) δ 177.08, 172.84, 154.91, 140.42, 134.63, 128.48(2C), 128.45, 127.74(2C), 127.36, 126.91, 126.39, 121.82, 120.60, 111.56, 109.65, 109.63, 81.93, 64.41, 62.95, 62.71, 59.16, 27.83(3C).

ESI-HRMS $[M+Na]^+$: calcd for $C_{26}H_{25}Br_1N_2Na_1O_4$ 531.08954, found 531.09008.

Enantiometric excess was determined by HPLC with a Chiralpack AD-H column (90:10 hexane: 2-propanol, 0.7 mL/min, 254 nm); major enantiomer t_r = 11.4 min, minor enantiomer t_r = 32.3 min; 88% ee.

tert-Butyl (2'*S*,3*R*,4'*S*,5'*S*)-2-oxo-4'-phenyl-2'-(5-Iodothiophen-2-yl)spiro [indoline-3,3'-pyrrolidine]-5'-carboxylate **(3e)**; 32%

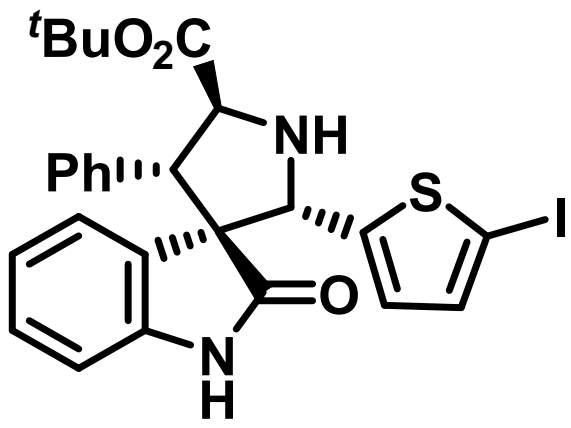

IR (ART): 3210, 2978, 1619, 1470, 1368, 1332, 1242, 1153, 1129, 1019, 842, 775, 752, 699 cm^{-1}.

^{1}H-NMR (400 MHz, $CDCl_3$) δ 7.66 (d, *J* = 7.3 Hz, 1H), 7.04 (m, 7H), 6.86 (d, *J* = 3.7 Hz, 1H), 6.49 (t, *J* = 4.1 Hz, 2H), 5.45 (s, 1H), 4.52 (d, *J* = 10.5 Hz, 1H), 4.05 (d, *J* = 10.5 Hz, 1H), 3.29 (br, 1H), 1.23 (s, 9H).

^{13}C-NMR (600 MHz, $CDCl_3$) δ 176.78, 172.92, 149.05, 140.22, 136.34, 134.88, 128.58, 128.39(2C), 127.77(2C), 127.52, 127.36, 125.98, 125.55, 122.12, 109.65, 81.97, 72.75, 65.70, 65.39, 62.23, 58.89, 27.85(3C).

ESI-HRMS $[M+H]^+$: calcd for $C_{26}H_{26}I_1N_2O_3S_1$ 573.07088, found 573.06381.

Enantiometric excess was determined by HPLC with a Chiralpack OD-H column (90:10 hexane: 2-propanol, 0.7 mL/min, 254 nm); major enantiomer t_r = 17.2 min, minor enantiomer t_r = 8.4 min; 93% ee.

tert-Butyl (2'*S*,3*R*,4'*S*,5'*S*)-2-oxo-4'-phenyl-2'-(5-Iodofuran-2-yl)spiro [indoline-3,3'-pyrrolidine]-5'-carboxylate **(3f)**; 79%

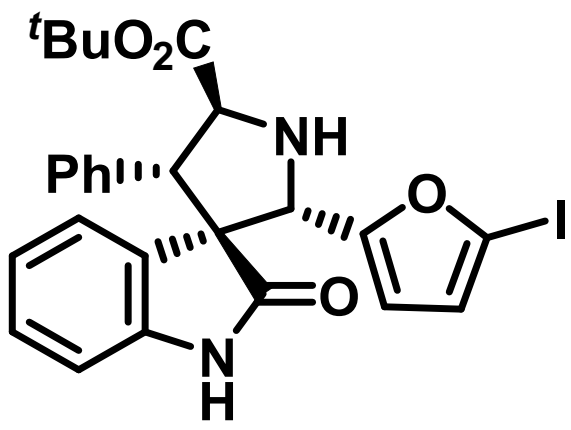

IR(ATR) 3208, 2977, 1709, 1619, 1470, 1368, 1244, 1154, 1012, 843, 786, 747.

^{1}H-NMR (400 MHz, $CDCl_3$) δ 7.52 (d, *J* = 7.8 Hz, 1H), 6.94-7.21 (m, 7H), 6.55 (d, *J* = 7.8 Hz, 1H), 6.19 (d, *J* = 3.2 Hz, 1H), 5.99 (d, *J* = 3.2 Hz, 1H), 5.20 (s, 1H), 4.50 (d, J = 11.0 Hz, 1H), 4.03 (d, *J* = 11.0 Hz, 1H), 3.42-3.02 (br, 1H), 1.22 (s, 9H).

^{13}C-NMR (600 MHz, $CDCl_3$) δ 177.01, 172.83, 158.45, 140.39, 134.66, 128.50(2C), 128.44, 127.74(2C), 127.36, 126.92, 126.41, 121.80, 120.50, 110.00, 109.57, 86.34, 81.92, 64.49, 62.98, 62.77, 59.13, 27.83(3C).

ESI-HRMS $[M+Na]^+$: calcd for $C_{26}H_{25}I_1N_2Na_1O_4$ 579.07567, found 579.08289.

Enantiometric excess was determined by HPLC with a Chiralpack IA column (90:10 hexane: 2-propanol, 0.7 mL/min, 254 nm); major enantiomer t_r = 7.5 min, minor enantiomer t_r = 15.2 min; 91% ee.

tert-Butyl (2'*R*,3*R*,4'*S*,5'*S*)-4'-(5-bromothiophene-2-yl)-2-oxo-2'-phenylspiro[indoline-3,3'-pyrrolidine]-5'-carboxylate **(3i)**; 30%

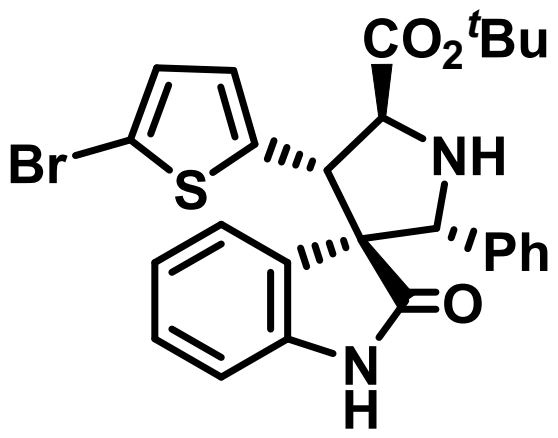

IR (ART) 2977, 2936, 1704, 1620, 1471, 1368, 1332, 1242, 1153, 1023, 842, 794, 748, 698 cm^{-1}.

^{1}H-NMR (400 MHz, $CDCl_3$) δ 7.59 (d, J = 6.9 Hz, 1H), 7.38-7.25 (3H), 7.11-7.01 (4H), 7.01-6.94 (1H), 6.69 (d, J = 3.4 Hz, 1H), 6.54 (d, J = 3.4 Hz, 1H), 6.52 (d, J = 6.9 Hz, 1H), 5.17 (s, 1H), 4.36 (d, J = 10.1 Hz, 1H), 4.30 (d, J = 10.1 Hz, 1H), 2.94 (br, 1H), 1.38 (s, 9H).

^{13}C-NMR (600 MHz, $CDCl_3$) δ 177.04, 173.04, 153.03, 140.12, 137.43, 128.28, 127.69(2C), 127.56, 127.13, 126.86(2C), 126.43, 121.85, 120.43, 111.64, 109.84, 109.25, 82.44, 68.71, 64.15, 62.09, 51.51, 28.06(3C).

ESI-HRMS $[M+H]^+$: calcd for $C_{26}H_{26}Br_1N_2O_3S_1$ 525.08475, found 525.08527.

Enantiometric excess was determined by HPLC with a Chiralpack AD-H column (90:10 hexane: 2-propanol, 0.7 mL/min, 254 nm); major enantiomer t_r = 16.8 min, minor enantiomer t_r = 23.9 min; 66% ee.

tert-Butyl (2'*R*,3*R*,4'*S*,5'*S*)-4'-(5-bromofuran-2-yl)-2-oxo-2'-phenylspiro[indoline-3,3'-pyrrolidine]-5'-carboxylate **(3j)**; 21%

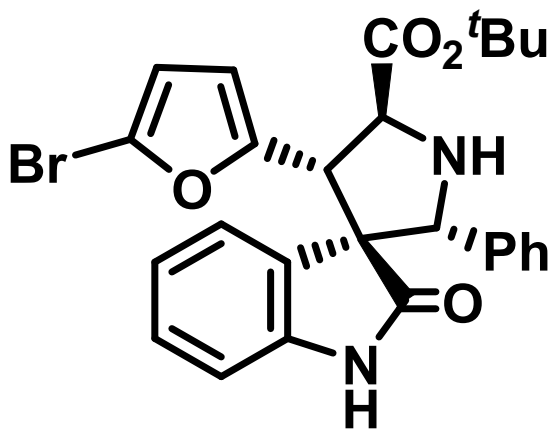

IR(ATR) 3210, 2978, 1620, 1470, 1368, 1331, 1242, 1153, 1129, 1019, 842, 775, 752.

^{1}H-NMR (400 MHz, $CDCl_3$) δ 7.57 (br, 1H), 7.50 (d, *J* = 7.3 Hz, 1H), 7.32-7.21 (3H), 7.11-6.96 (4H), 6.95-6.87 (1H), 6.53 (d, *J* = 7.3 Hz, 1H), 5.95 (d, *J* = 3.5 Hz, 1H), 5.80 (d, J = 3.5 Hz, 1H), 5.10 (s, 1H), 4.41 (d, *J* = 9.6 Hz, 1H), 4.22 (d, *J* = 9.6 Hz, 1H), 1.45 (s, 9H).

^{13}C-NMR (600 MHz, $CDCl_3$) δ 177.04, 173.04, 153.03, 140.12, 137.43, 128.28, 127.69(2C), 127.56, 127.13, 126.86(2C), 126.43, 121.85, 120.43, 111.64, 109.84, 109.25, 82.44, 68.71, 64.15, 62.09, 51.51, 28.06(3C).

ESI-HRMS $[M+Na]^+$: calcd for $C_{26}H_{25}Br_1N_2Na_1O_4$ 531.08954, found 531.09564.

Enantiometric excess was determined by HPLC with a Chiralpack IA column (90:10 hexane: 2-propanol, 0.7 mL/min, 254 nm); major enantiomer t_r = 10.6 min, minor enantiomer t_r = 9.5 min; 27% ee.

tert-Butyl (2'*R*,3*R*,4'*S*,5'*S*)-4'-(5-Iodothiophene-2-yl)-2-oxo-2'-phenylspiro[indoline-3,3'-pyrrolidine]-5'-carboxylate **(3k)**; 16%

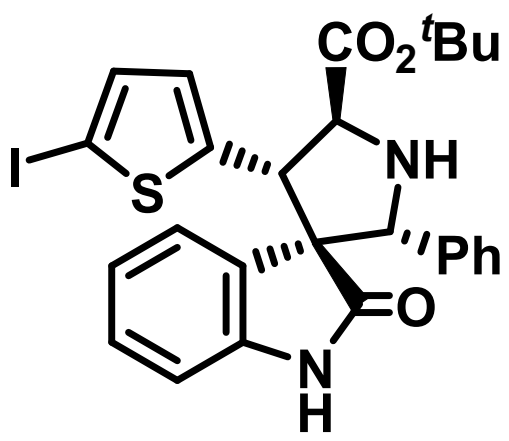

IR (ART): 2977, 2936, 1704, 1620, 1471, 1368, 1332, 1242, 1153, 1023, 842, 794, 748, 698 cm^{-1}.

^{1}H-NMR (400 MHz, $CDCl_3$) δ 7.59 (d, *J* = 6.9 Hz, 1H), 7.15-6.94 (m, 9H), 6.88 (d, *J* = 4.1 Hz, 1H), 6.50 (d, *J* = 6.9 Hz, 1H), 6.45 (d, *J* = 4.1 Hz, 1H), 5.17 (s, 1H), 4.42 (d, *J* = 10.1 Hz, 1H), 4.31 (d, *J* = 10.1 Hz, 1H), 2.94 (br, 1H), 1.48 (s, 9H).

^{13}C-NMR (600 MHz, $CDCl_3$) δ 179.52, 172.15, 141.59, 140.72, 136.34, 128.41, 128.16, 128.07(2C), 127.87(2C), 127.77, 127.64, 126.41, 125.51, 122.16, 109.31, 81.82, 67.35, 64.49, 64.15, 52.74, 28.06, 27.84(3C).

ESI-HRMS $[M+Na]^+$: calcd for $C_{26}H_{25}I_1N_2Na_1O_3S_1$ 595.05282, found 595.05179.

Enantiometric excess was determined by HPLC with a Chiralpack AD-H column (80:20 hexane: 2-propanol, 0.7 mL/min, 254 nm); major enantiomer t_r = 11.3 min, minor enantiomer t_r = 14.9 min; 68% ee.

tert-Butyl (2'*R*,3*R*,4'*S*,5'*S*)-4'-(5-Iodofuran-2-yl)-2-oxo-2'-phenylspiro[indoline-3,3'-pyrrolidine]-5'-carboxylate **(3l)**; 34%

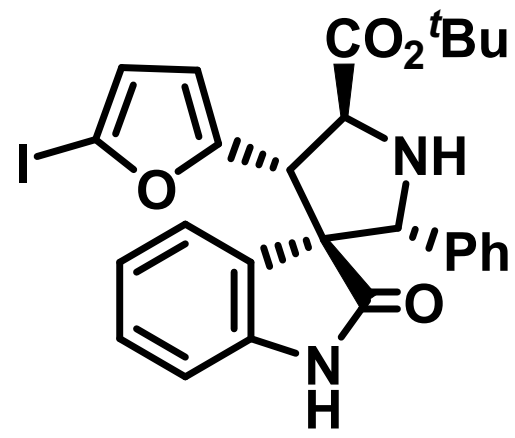

IR(ATR): 3212, 2978, 1620, 1470, 1368, 1335, 1242, 1153, 1129, 1018, 842, 775, 752.

^{1}H-NMR (400 MHz, $CDCl_3$) δ 7.49 (d, J = 7.8 Hz, 1H), 7.39 (br, 1H), 7.27-7.24 (m, 2H), 7.04-7.04 (m, 3H), 6.99 (td, J = 7.8, 1.4 Hz, 1H), 6.90 (t, J = 7.1 Hz, 1H), 6.52 (d, J = 7.8 Hz, 1H), 6.18 (d, J = 3.2 Hz, 1H), 5.75 (d, J = 3.2 Hz, 1H), 5.10 (s, 1H), 4.41 (d, J = 9.6 Hz, 1H), 4.26 (d, J = 9.6 Hz, 1H), 1.45 (s, 9H).

^{13}C-NMR (600 MHz, $CDCl_3$) δ 177.06, 173.13, 156.53, 140.03, 137.56, 128.24, 127.67(2C), 127.51, 127.12, 126.85(2C), 126.50, 121.79, 120.58, 110.14, 109.18, 86.06, 82.37, 68.63, 64.21, 62.24, 51.52, 28.08(3C).

ESI-HRMS $[M+Na]^+$: calcd for $C_{26}H_{25}I_1N_2Na_1O_4$ 579.07567, found 579.07734.

Enantiometric excess was determined by HPLC with a Chiralpack AD-H column (80:20 hexane: 2-propanol, 0.7 mL/min, 254 nm); major enantiomer t_r = 16.8 min, minor enantiomer t_r = 21.9 min; 24% ee.

Compound **3g, h**

General procedure

To a solution of **3c** (9.2 mg, 0.018 mmol) in n-BuOH (0.86 ml) were added (5-Indole)cyclic-triolborate Pottasium Salt (9.9 mg, 0.036 mmol), $Pd(OAc)_2$ (0.39 mg, 10 mol%) and 2-dicyclohexylphosphino-2',6'-dimethoxybiphenyl (1.44 mg, 20 mol%). The reaction mixture was stirred for 7 h at 100 °C and filtered through celite. After filtration, the resulting residue was purified by silicagel column chromatography (hexane/EtOAc = 3/1) to afford **3g** (5.3 mg, 52%) as a white amorphous solid.

tert-Butyl (2'*S*,3*R*,4'*S*,5'*S*)-2-oxo-4'-phenyl-2'-(5-indolethiophen-2-yl)spiro [indoline-3,3'-pyrrolidine]-5'-carboxylate **(3g)**; 52%

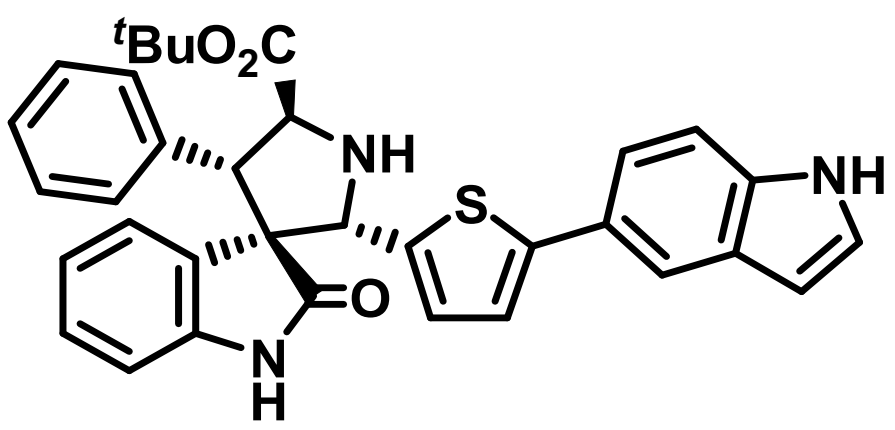

IR (ART) 3323, 2976, 2920,2853, 1710, 1620, 1470,1368, 1343, 1244, 1156, 798, 751, 729, 698 cm^{-1}.

^{1}H-NMR (400 MHz, $CDCl_3$) δ 8.12 (s, 1H), 7.76 (t, 1H), 7.69 (s, 1H), 7.30 (m, 3H), 7.19 (t, 1H), 7.10 (s, 1H), 7.06 (m, 4H), 7.01 (t, J = 3.9 Hz, 1H), 6.91 (d, J = 3.7 Hz, 1H), 6.81 (d, J = 3.7 Hz, 1H), 6.51 (t, 1H), 6.45-6.47 (m, 1H), 5.47 (s, 1H), 4.58 (d, J = 10.5 Hz, 1H), 4.12 (d, J = 10.5 Hz, 1H), 1.26 (s, 9H).

^{13}C-NMR (600 MHz, $CDCl_3$) δ 176.66, 173.17, 144.84, 140.10, 135.25, 128.46, 128.40(2C), 128.25, 127.76(2C), 127.27, 126.30, 124.97, 122.03, 121.22, 120.70, 117.81, 111.23, 109.40, 103.04, 81.85, 65.85, 65.65, 62.34, 58.89, 27.88(3C).

ESI-HRMS $[M+Na]^+$: $C_{34}H_{31}N_3Na_1O_3S_1$ found 584.21535.

tert-Butyl (2'*S*,3*R*,4'*S*,5'*S*)-2-oxo-4'-phenyl-2'-(5-indolefuran-2-yl)spiro [indoline-3,3'-pyrrolidine]-5'-carboxylate **(3h)**; 58%

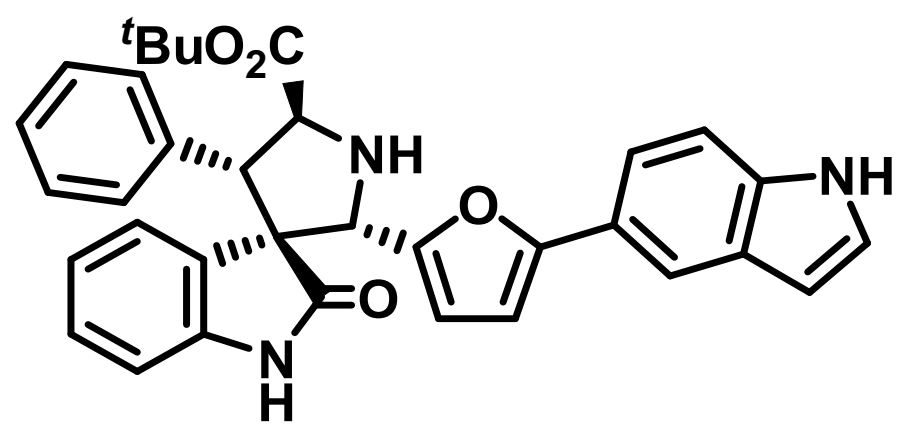

IR (ART) 3325, 3060, 2976, 2929, 1706, 1618, 1597, 1470,1393, 1368, 1325, 1243, 1155, 844, 745, 696 cm^{-1}.

^{1}H-NMR (400 MHz, $CDCl_3$) δ 8.18 (s, 1H), 7.79 (s, 1H), 7.34-7.40 (m, 2H), 7.21 (t, *J* = 2.7 Hz, 1H), 7.14-7.03 (6H), 7.00 (d, *J* = 7.8 Hz, 1H), 6.70-6.74 (m, 1H), 6.58 (d, *J* = 7.8 Hz, 1H), 6.52-6.55 (m, 4H), 4.89 (d, *J* = 11.0 Hz, 1H), 4.85 (s, 1H), 3.98 (d, *J* = 11.0 Hz, 1H), 1.28 (s, 9H).; ^{13}C-NMR (151 MHz, $CDCl_3$) δ 176.63, 173.86, 142.76, 140.24, 135.06, 128.42(2C), 128.32, 127.76(2C), 127.28, 126.30, 124.43, 124.20, 123.97, 122.06, 121.85, 120.81, 119.88, 111.07, 109.36, 102.73, 81.87, 65.91, 65.36, 62.36, 58.83, 27.86(3C).

ESI-HRMS $[M+H]^+$: $C_{34}H_{32}N_3O_4$ found 546.23302.

Compound **4d**

(2'*S*,3*R*,4'*S*,5'*S*)-2-oxo-4'-phenyl-2'-(5-bromofuran-2-yl)spiro [indoline-3,3'-pyrrolidine]-5'-carboxylate (4d)

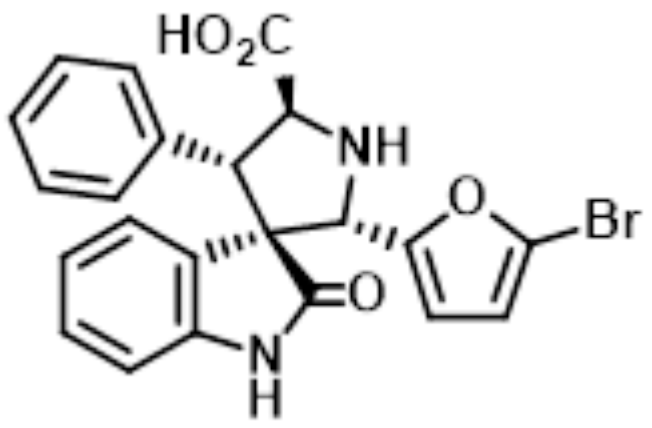

To a solution of **3d**(6.4 mg, 0.013 mmol) in 1,2-dichloroethane (300 μl) was added TFA (100 μl). The reaction mixture was stirred for 2 h at 40 °C and evaporated. The resulting residue was purified by HPLC (75% MeOH) to afford **4d** (2.4 mg, 41%) as a white amorphous solid.

^{1}H-NMR (400 MHz, $CDCl_3$) δ 10.12 (s, 1H) 7.53 (d, *J* = 7.5 Hz, 1H), 7.47 (br, 1H), 6.94-7.05 (m, 7H), 6.55 (d, *J* = 7.5 Hz, 1H), 6.04 (d, *J* = 3.4 Hz, 1H), 5.95 (d, *J* = 3.4 Hz, 1H), 5.16 (s, 1H), 4.50 (d, *J* = 11.0 Hz, 1H), 4.03 (d, *J* = 11.0 Hz, 1H).

Compound **3d'**

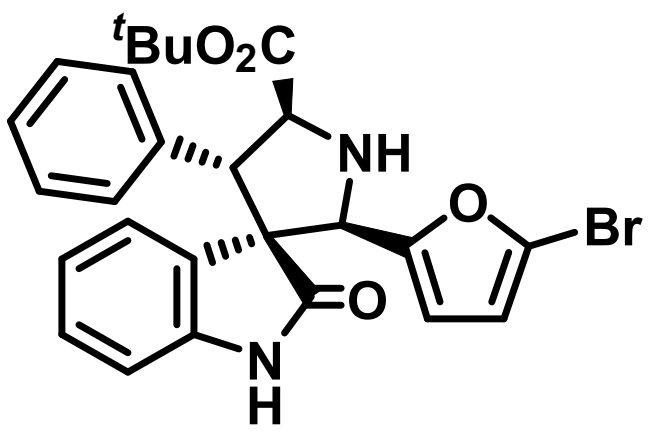

To a solution of (*R*)-1-[(*S*)-2(diphenylphosphino)ferrocenyl]ethlamine (6.6 mg, 10.8 μmol) and $CuPF_6(CH_3CN)_4$ (2.0 mg, 5.4 μmol) in MeOH (5.2 mL) were added subsequently iminoester (**2f**; 203.3 mg, 706 μmol), triethylamine (8.8 μL, 109 μmol) and methyleneindolinone (**1a**; 120 mg, 543 μmol) was added at 0 °C. After being stirred for 72 h at room temperature, the reaction mixture was quenched by NH_4Cl aq. and extracted with ethyl acetate. The organic layer was dried over Na_2SO_4. After filtration and concentration, the resulting residue was purified by silicagel column chromatography (hexane/EtOAc = 88/12 to 0/100) and HPLC with a COSMOSIL Packed Column πNAP (MeOH/H_2O = 70/30, 5.0 mL/min, 254 nm) to afford **3d'** (45.3 mg, 89.1 μmol, 16%) as a colorless amorphous solid.

^{1}H-NMR (500 MHz, CHLOROFORM-D) δ 7.7 (s, 1H), 7.15 (t, *J* = 6.2 Hz, 1H), 7.15 (d, *J* = 7.4 Hz, 2H), 7.10 (dt, *J* = 1.4, 7.4 Hz , 1H), 7.05 (d, *J* = 6.2 Hz, 1H), 7.05 (d, *J* = 7.0 Hz, 1H), 6.87

(t, *J* = 7.4 Hz, 1H), 6.83 (t, *J* = 7.4 Hz, 1H), 6.70 (d, *J* = 7.7 Hz, 1H), 6.36 (d, *J* = 3.44 Hz, 1H), 6.20 (d, *J* = 3.44 Hz, 1H), 4.64 (s, 1H), 4.44 (d, *J* = 8.3 Hz, 1H), 4.00 (d, *J* = 8.3 Hz, 1H), 3.29 (s, br, 1H), 1.33 (s, 9H).

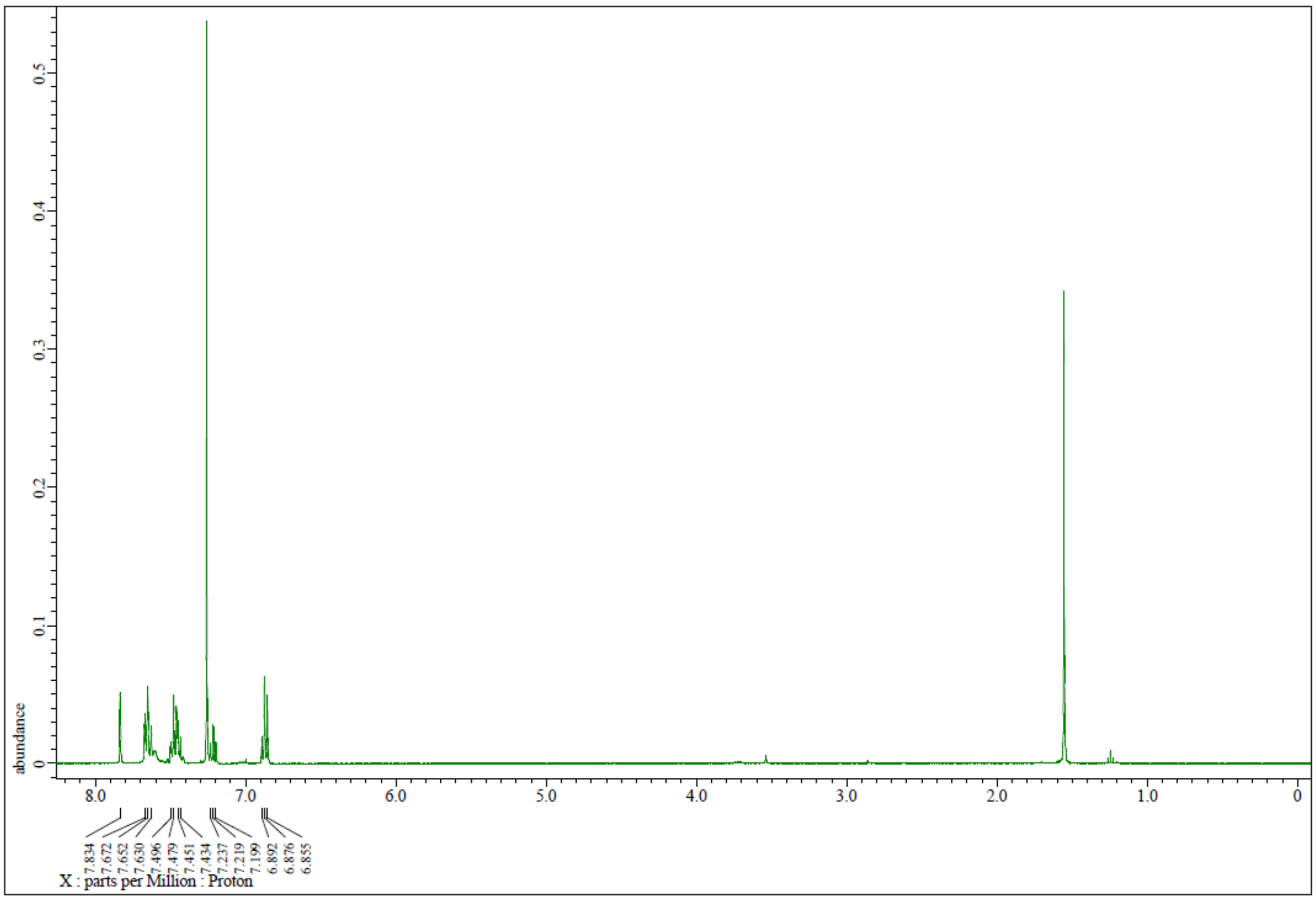

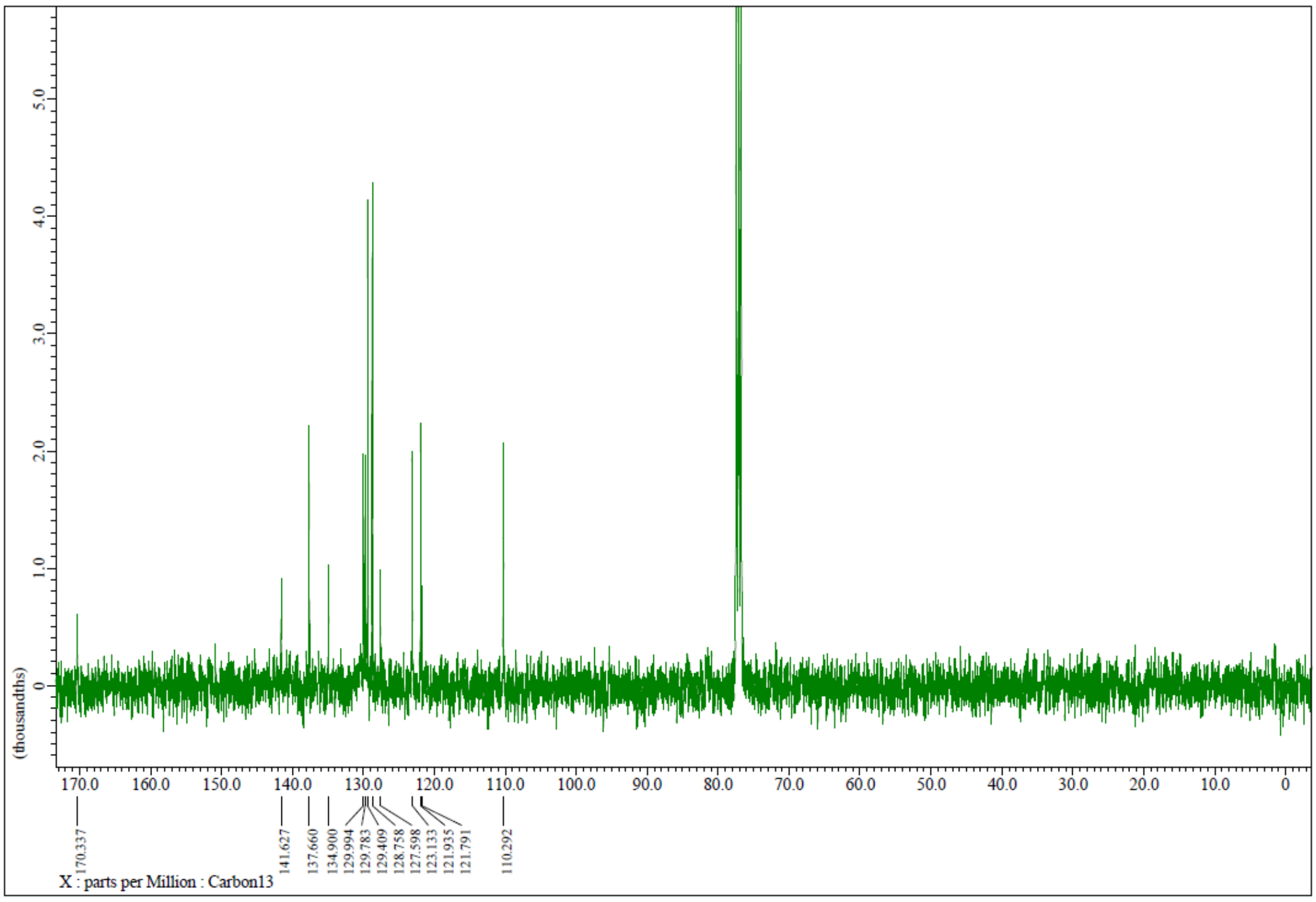

Fig. S21 ^{1}H-NMR and ^{13}C-NMR of 1a

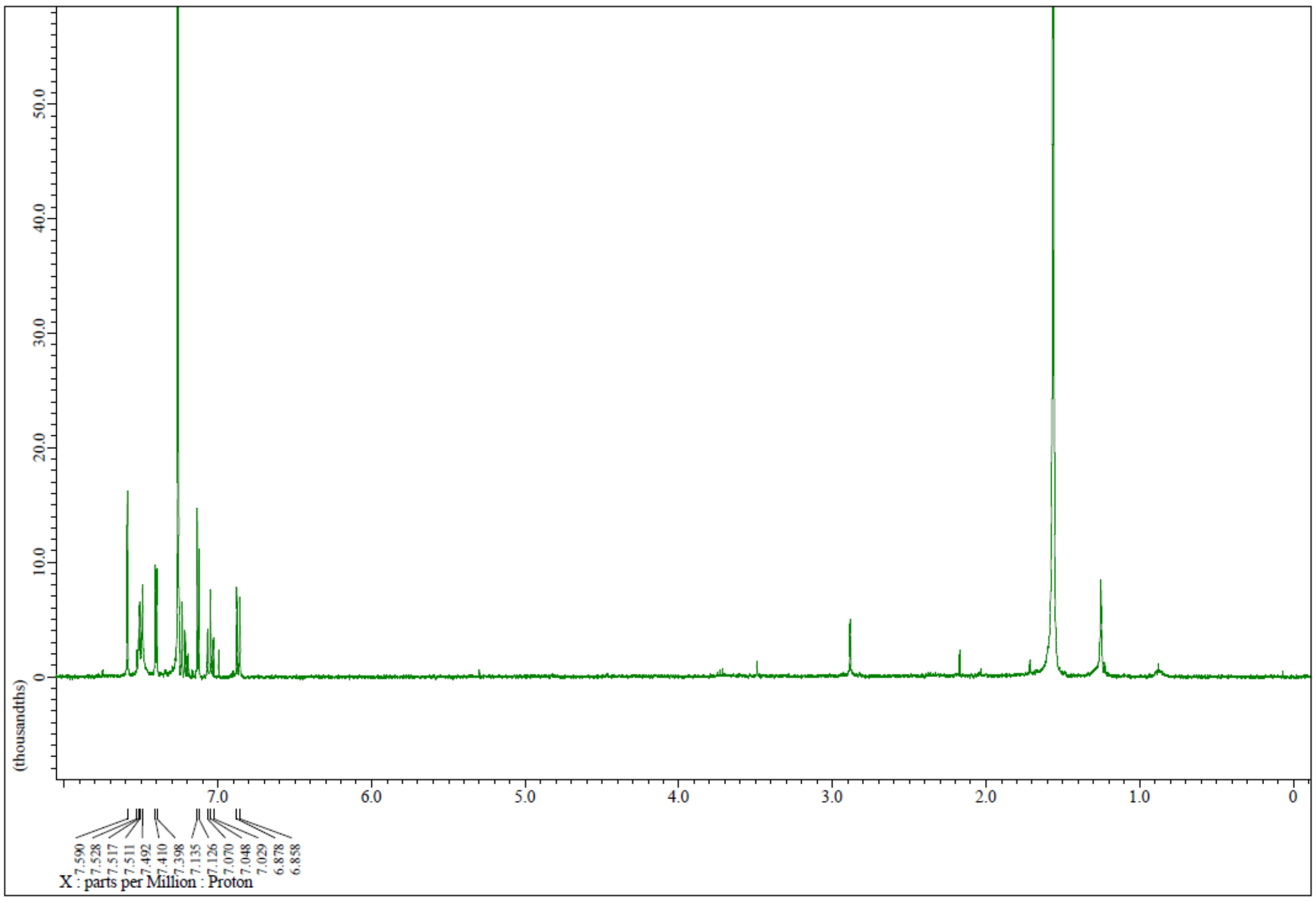

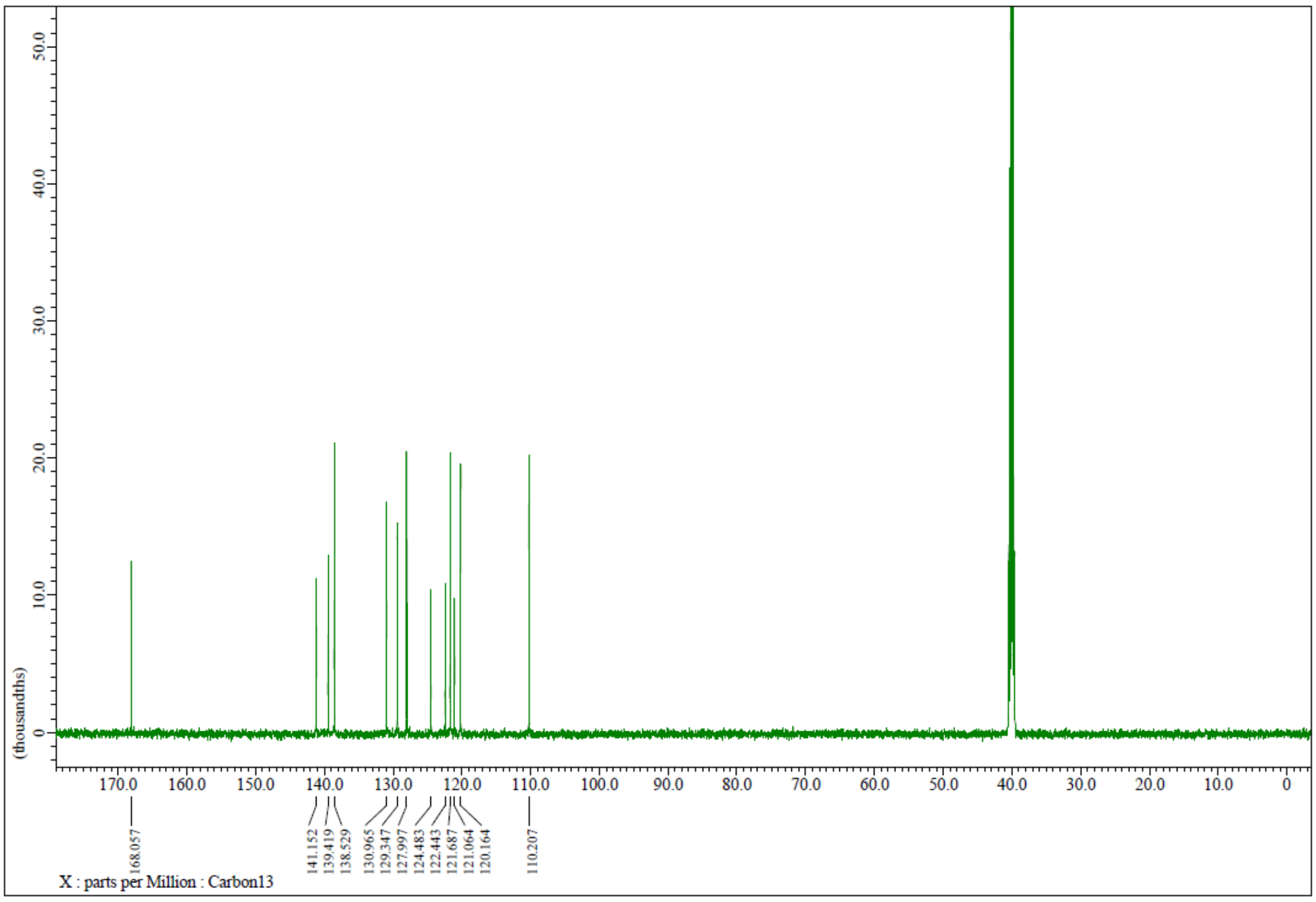

Fig. S22 ^{1}H-NMR and ^{13}C-NMR of 1b

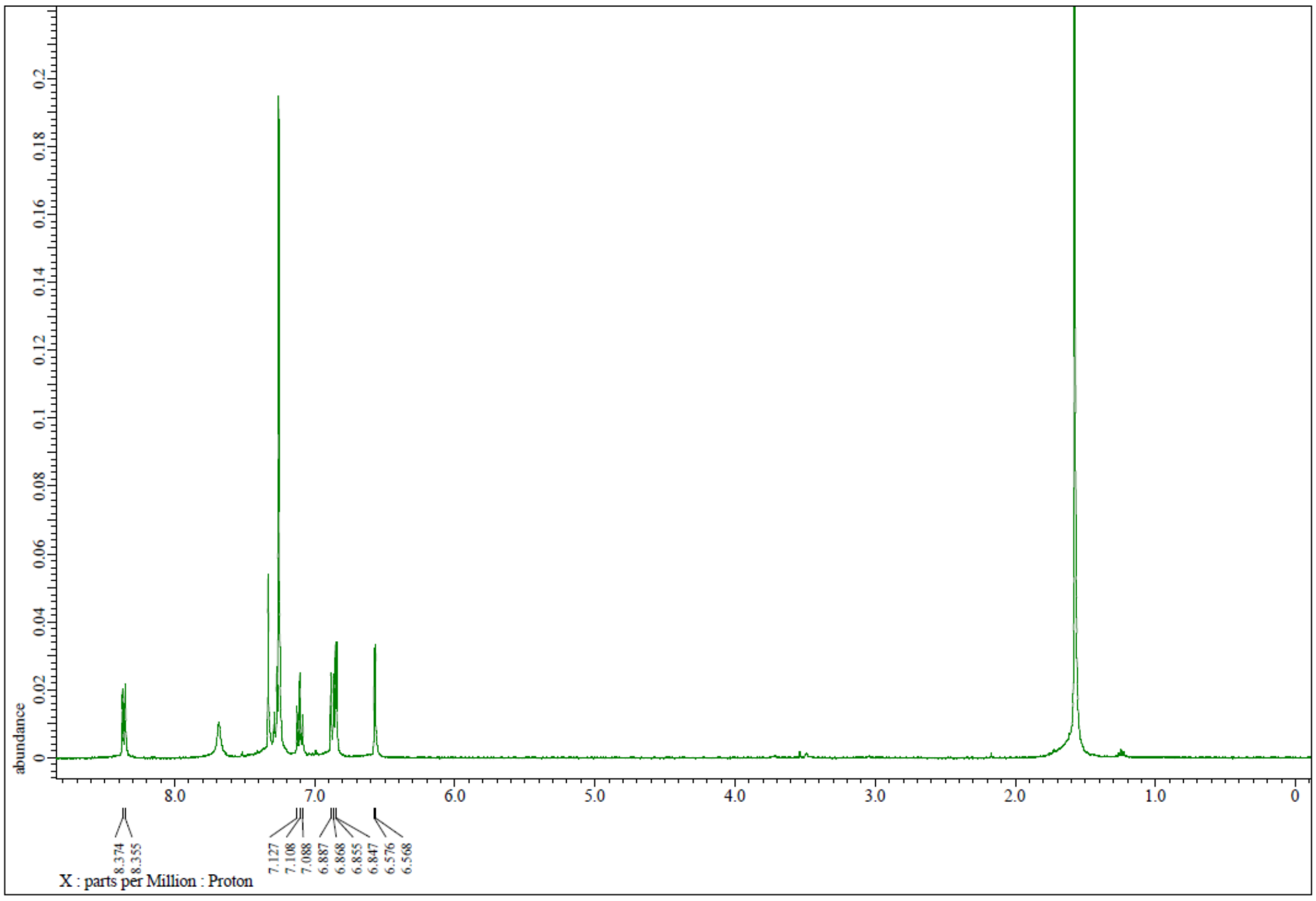

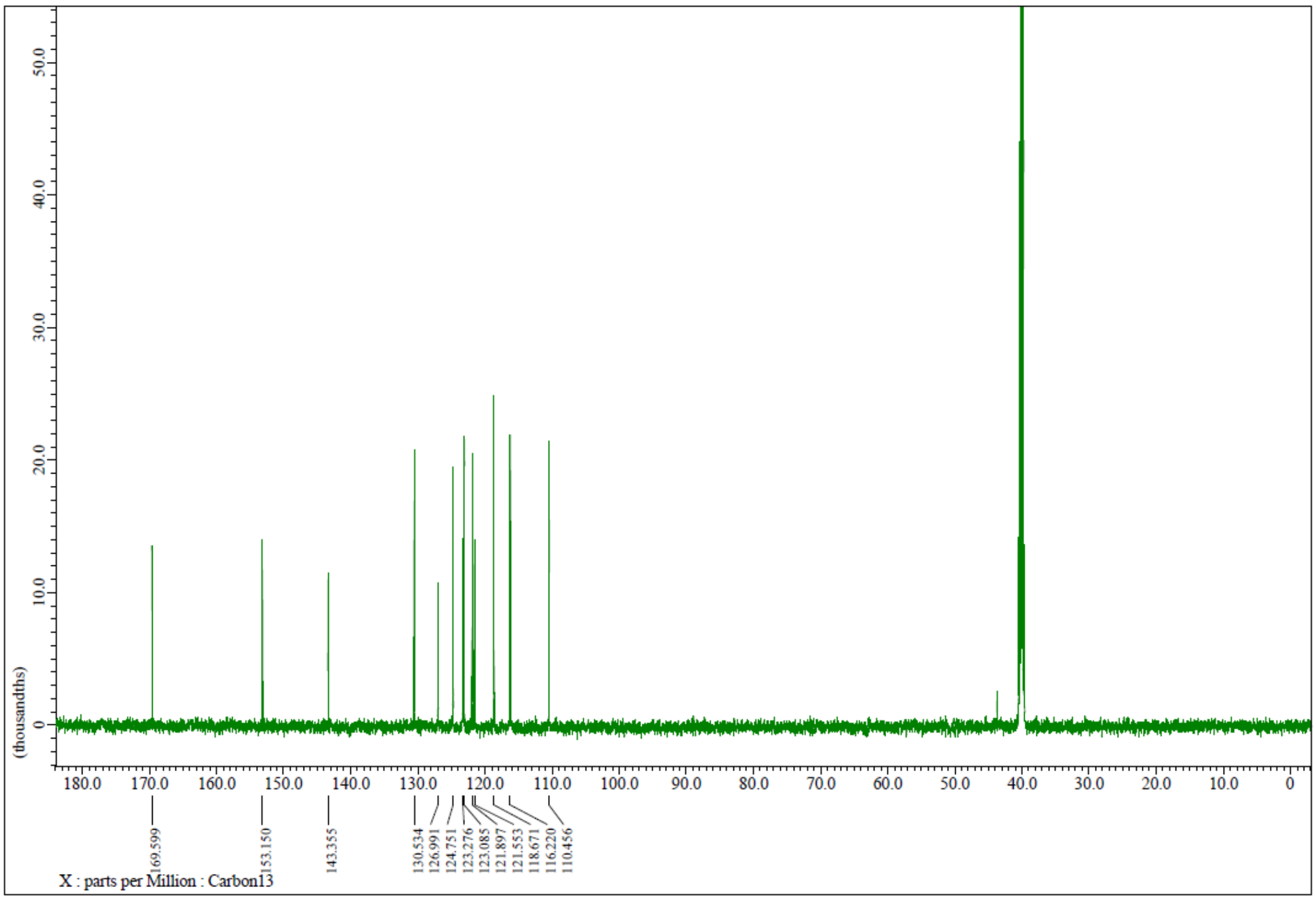

Fig. S23 ^{1}H-NMR and ^{13}C-NMR of 1c

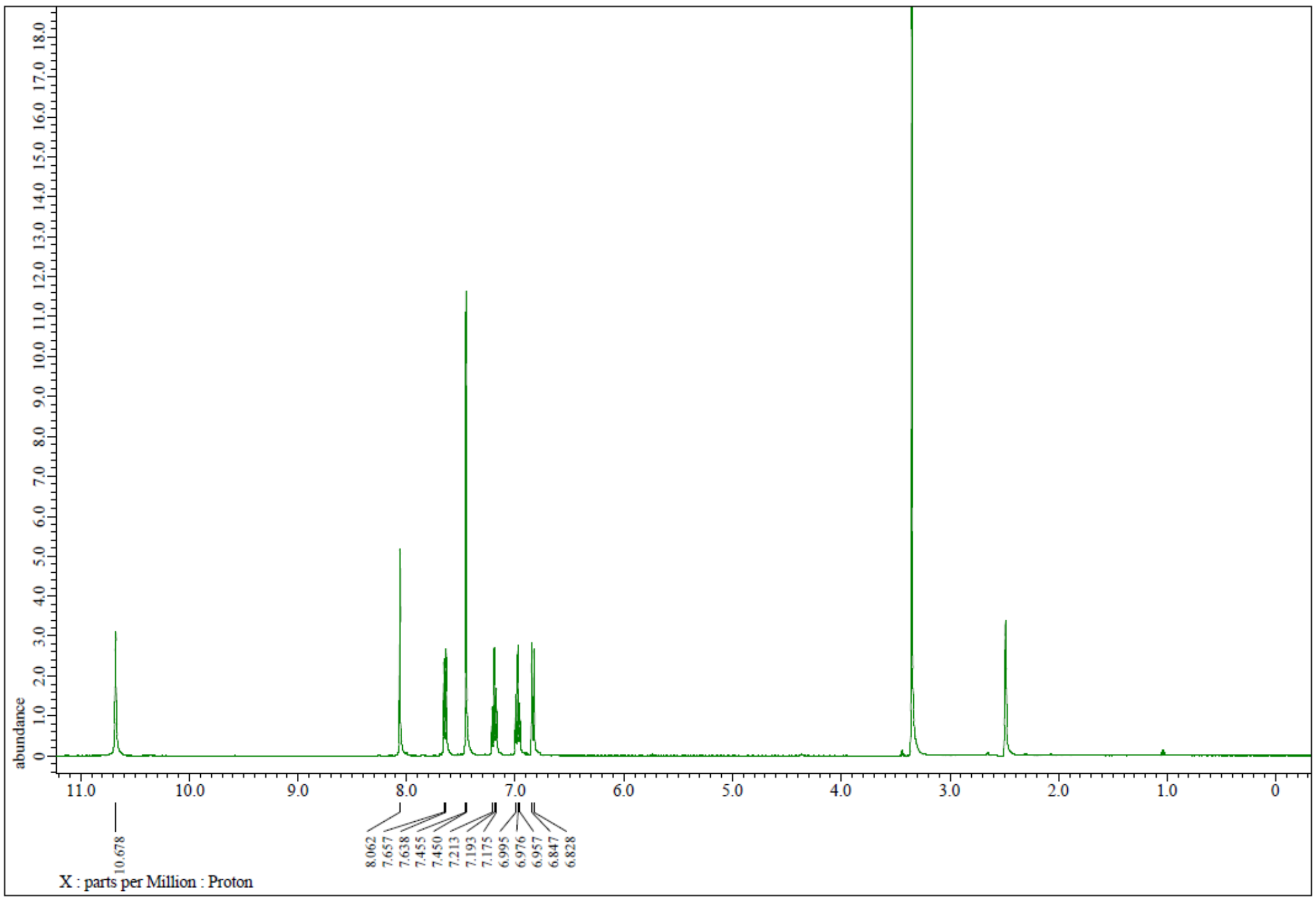

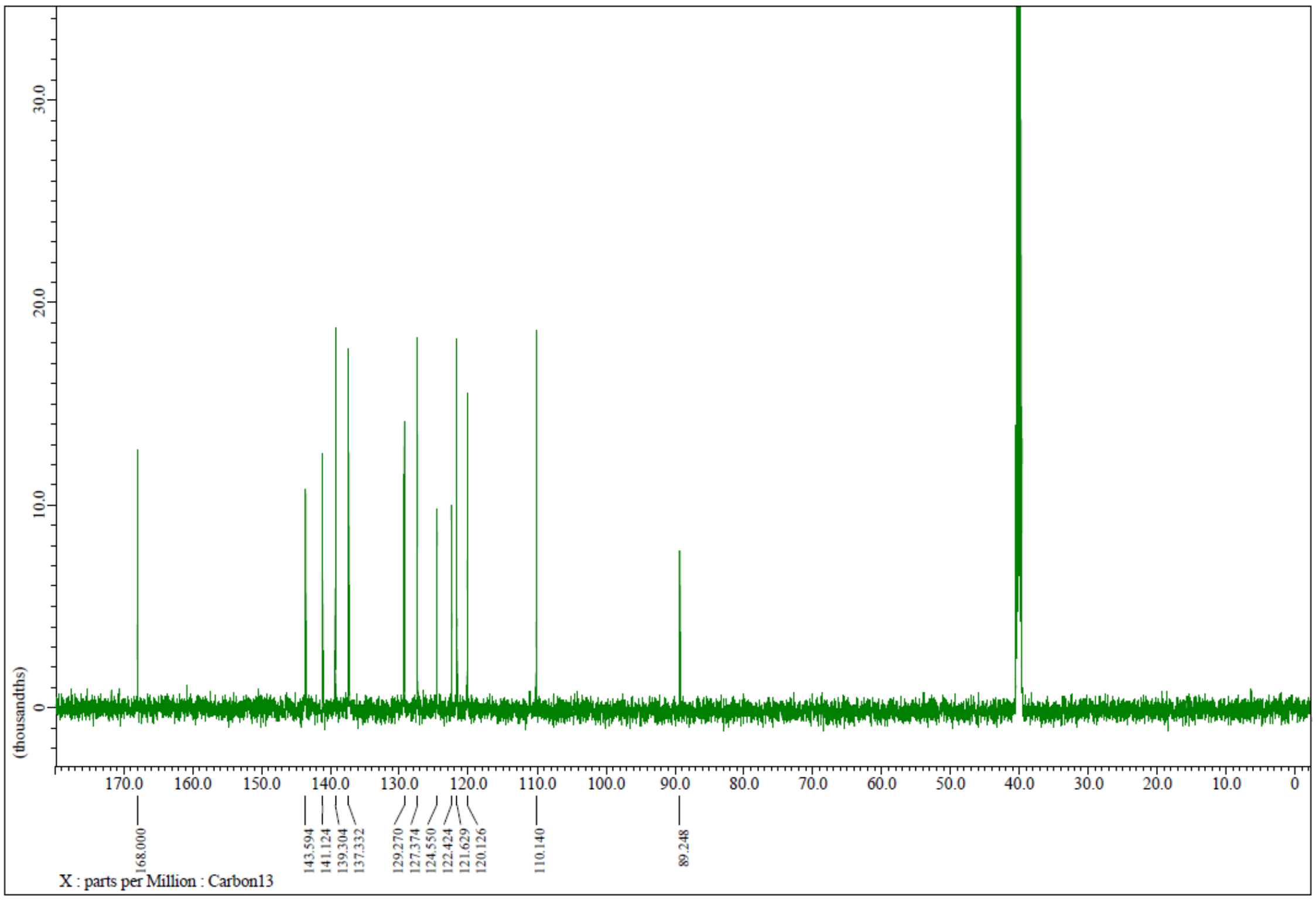

Fig. S24 ^{1}H-NMR and ^{13}C-NMR of 1d

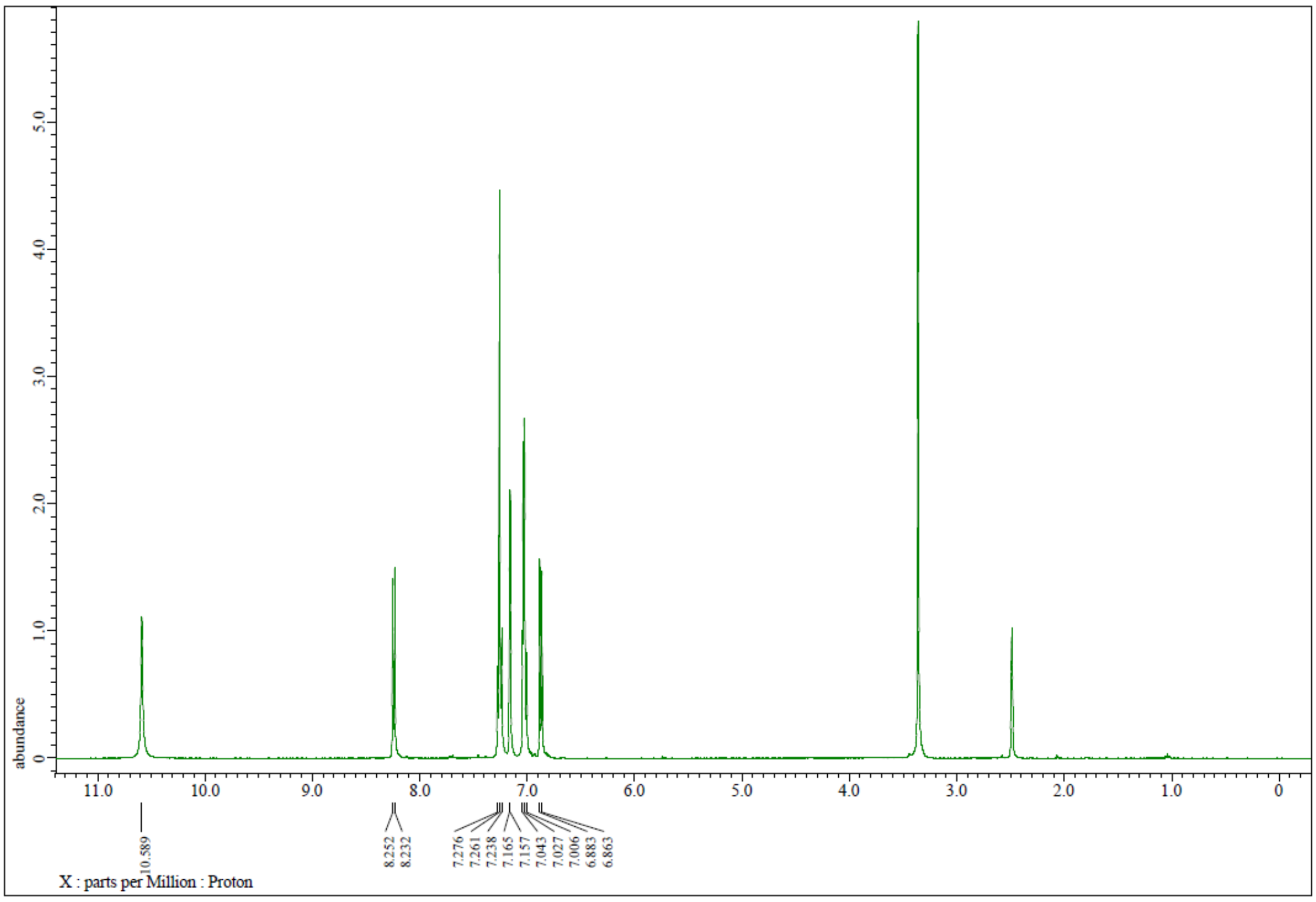

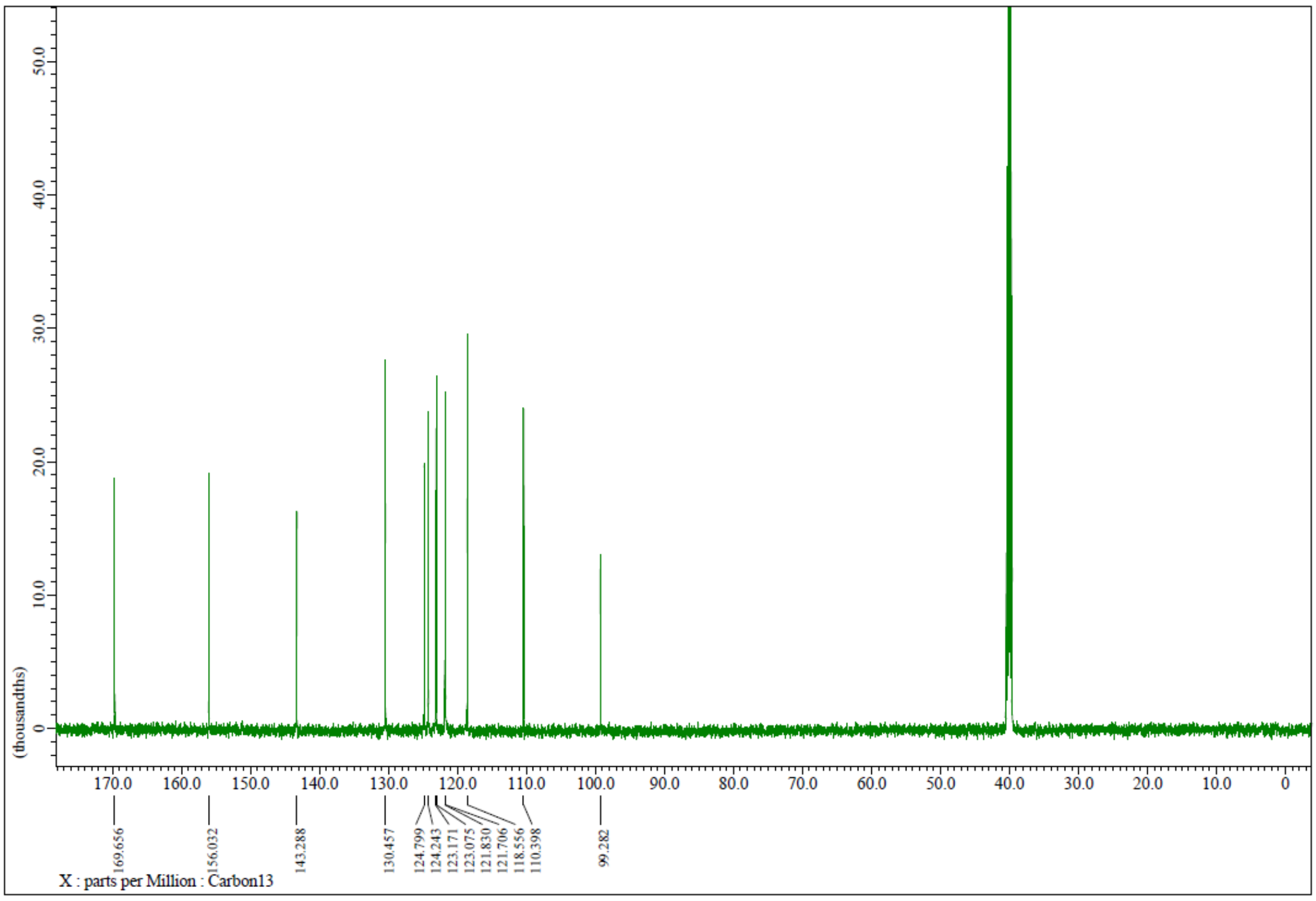

Fig. S25 ^{1}H-NMR and ^{13}C-NMR of 1e

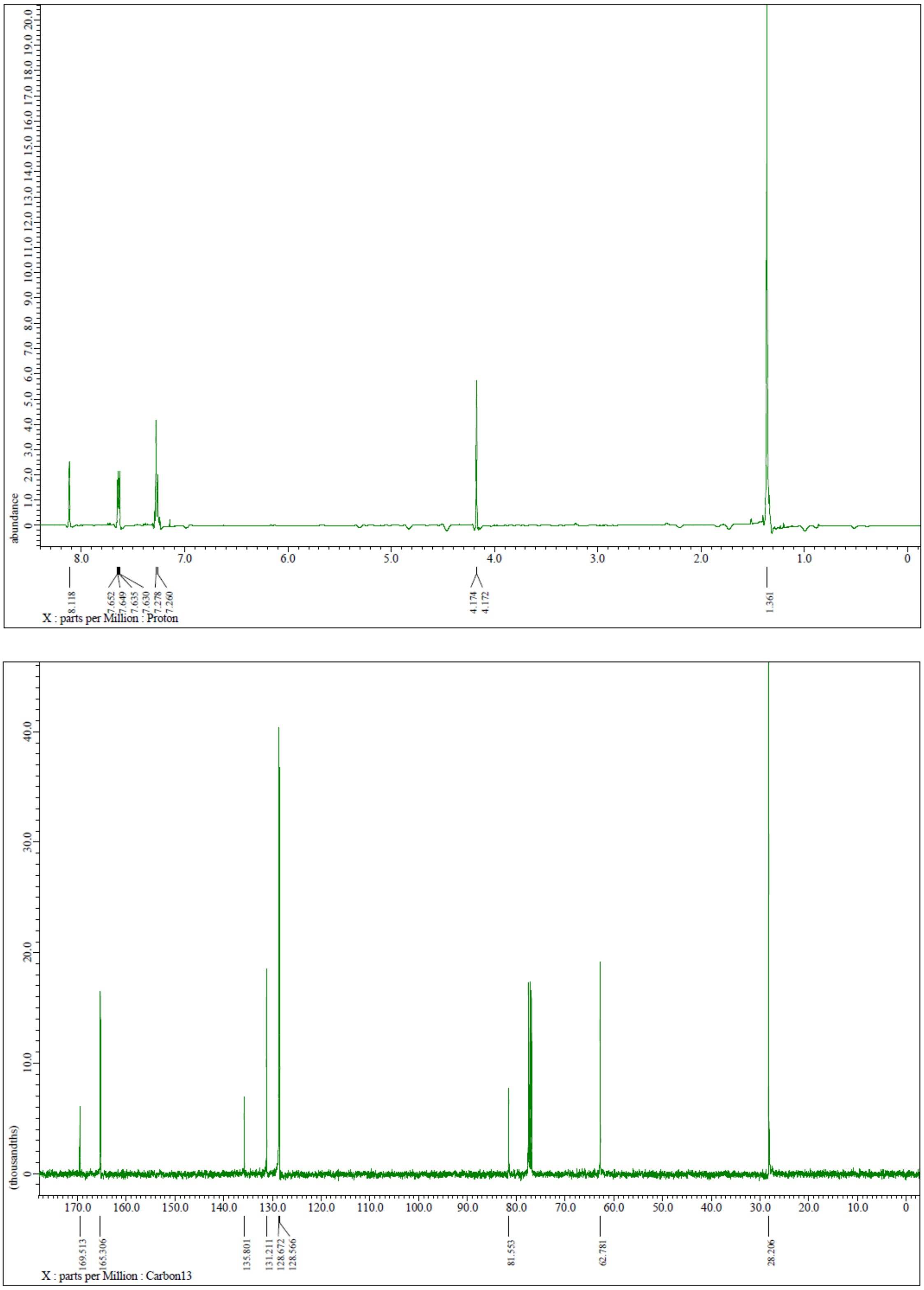

Fig. S26 ^{1}H-NMR and ^{13}C-NMR of 2a

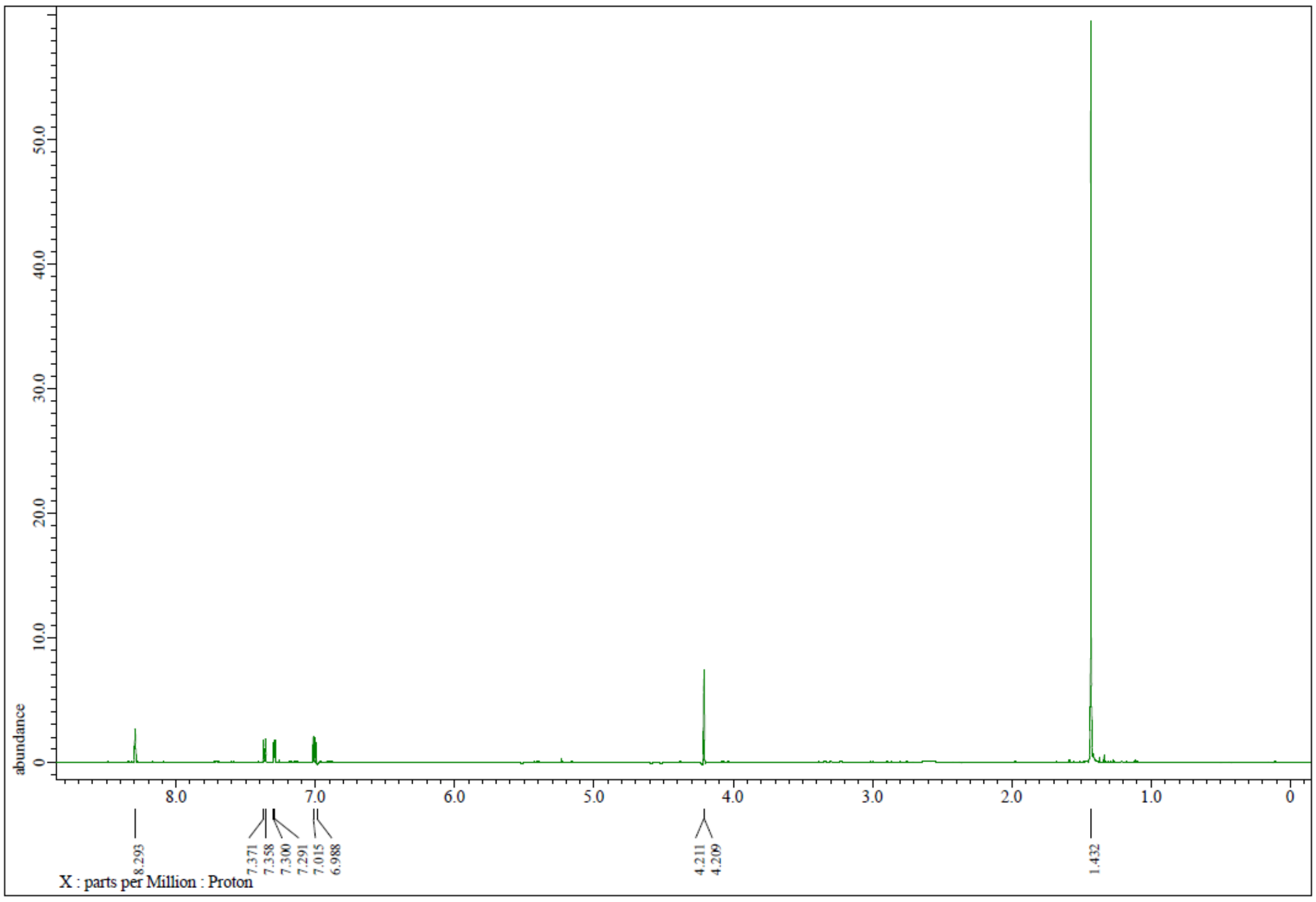

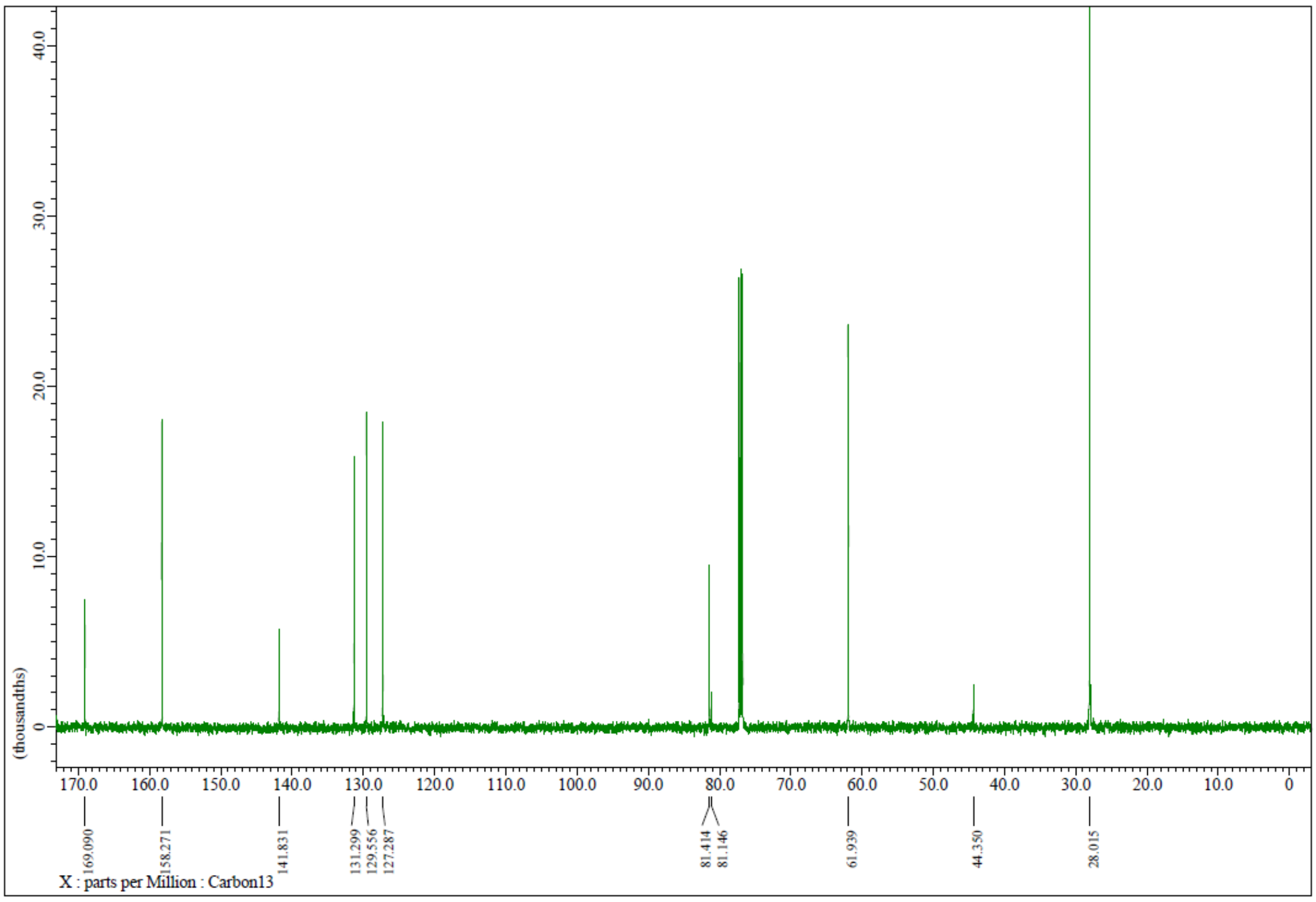

Fig. S27 ^{1}H-NMR and ^{13}C-NMR of 2b

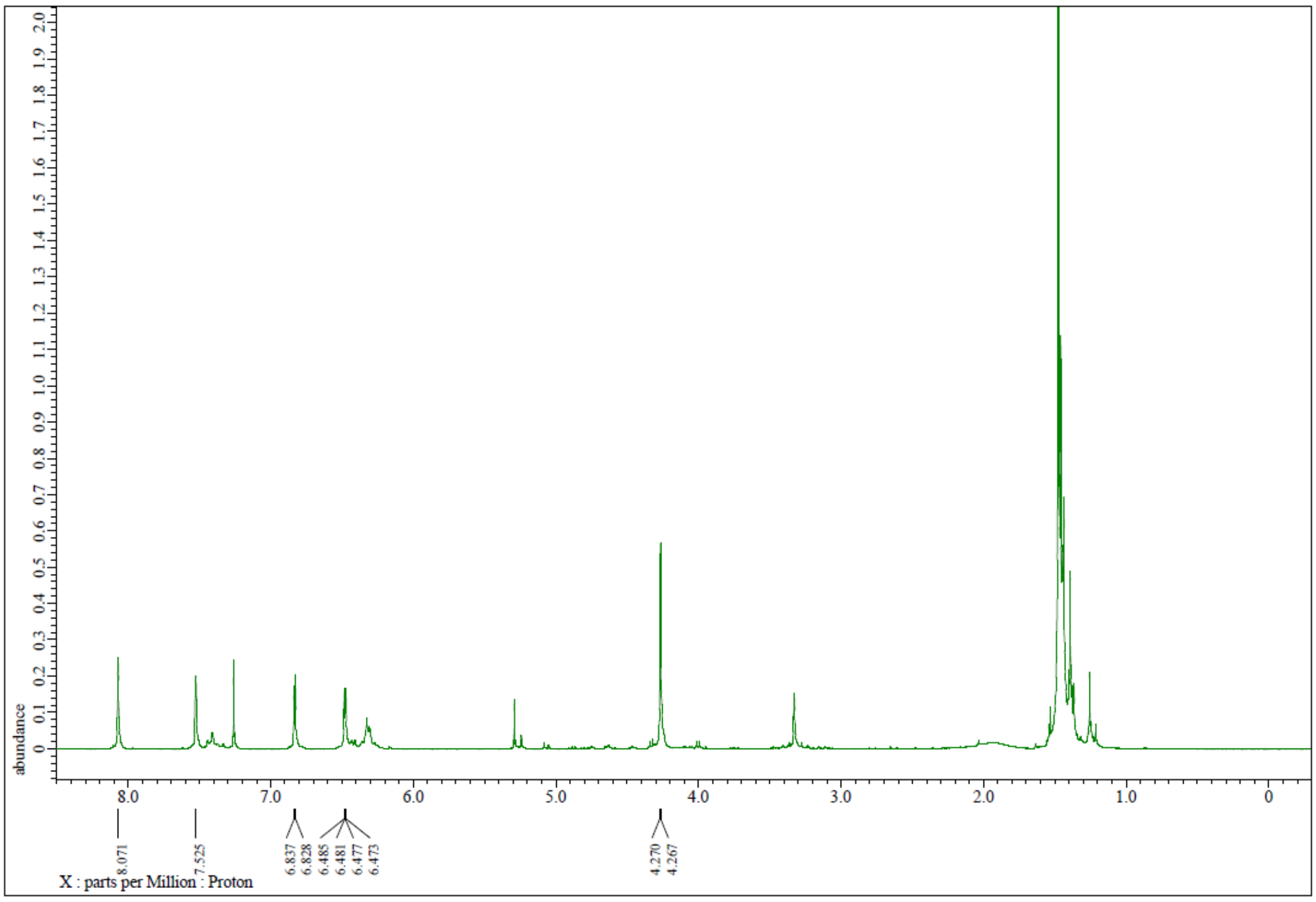

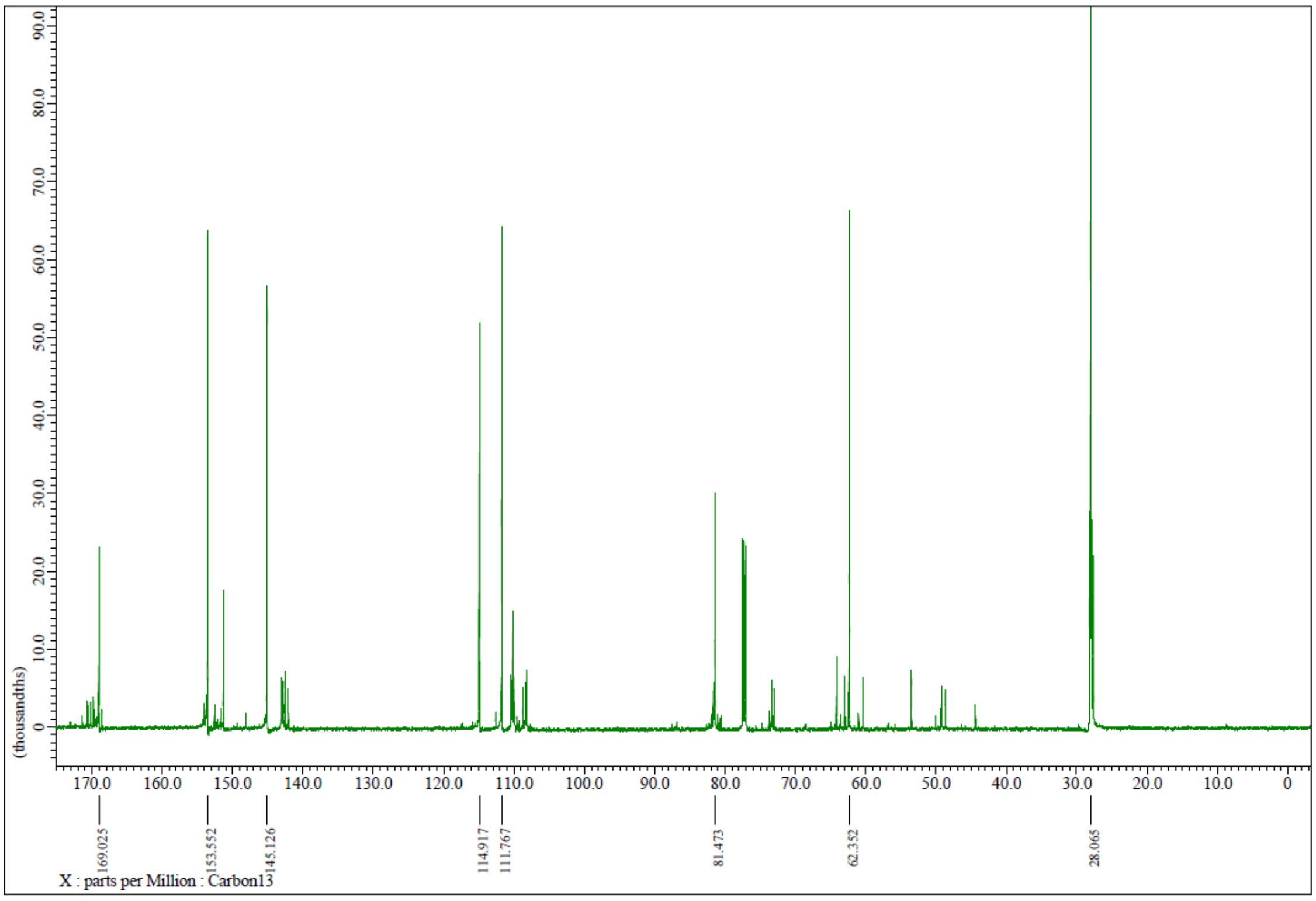

Fig. S28 ^{1}H-NMR and ^{13}C-NMR of 2c

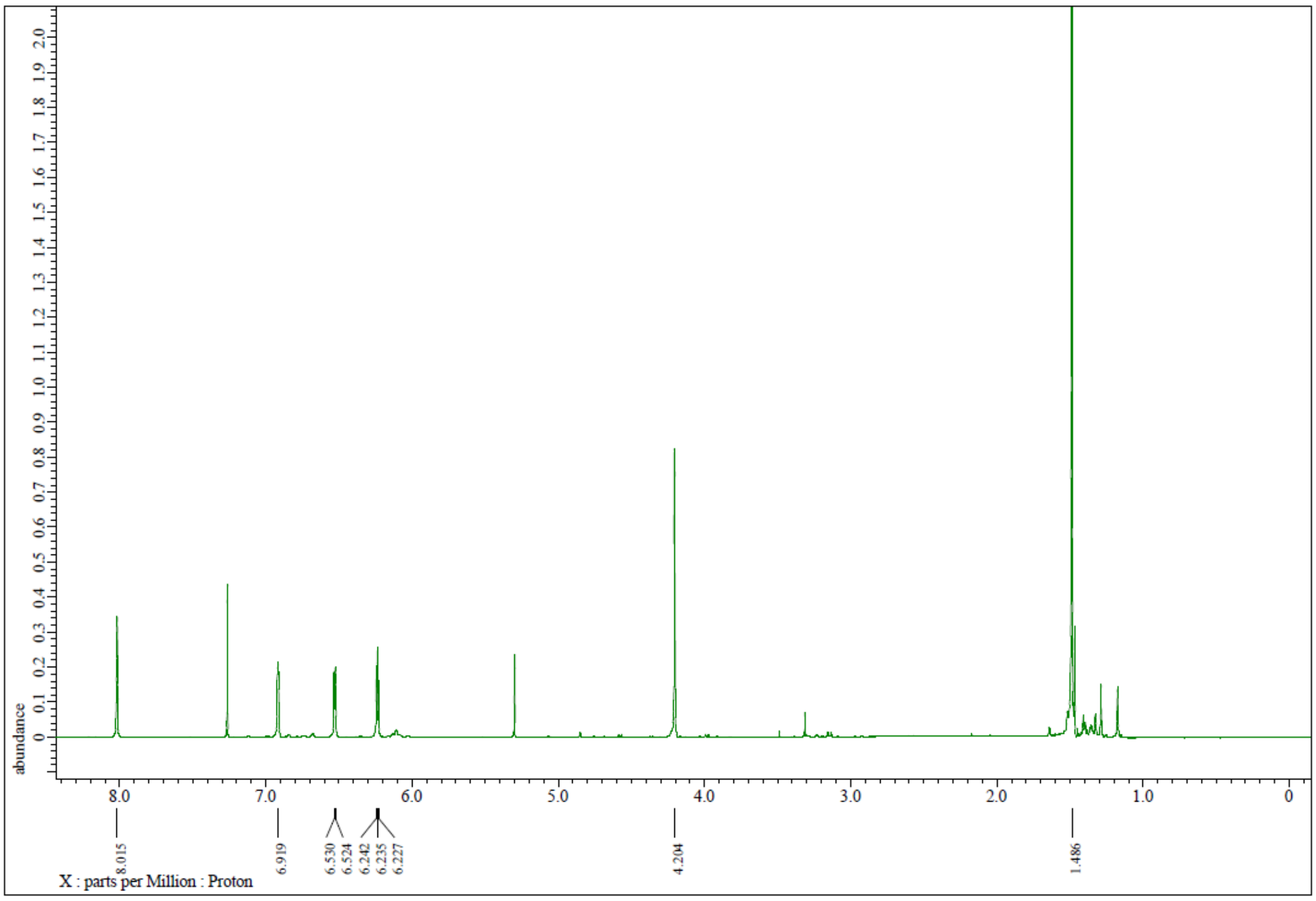

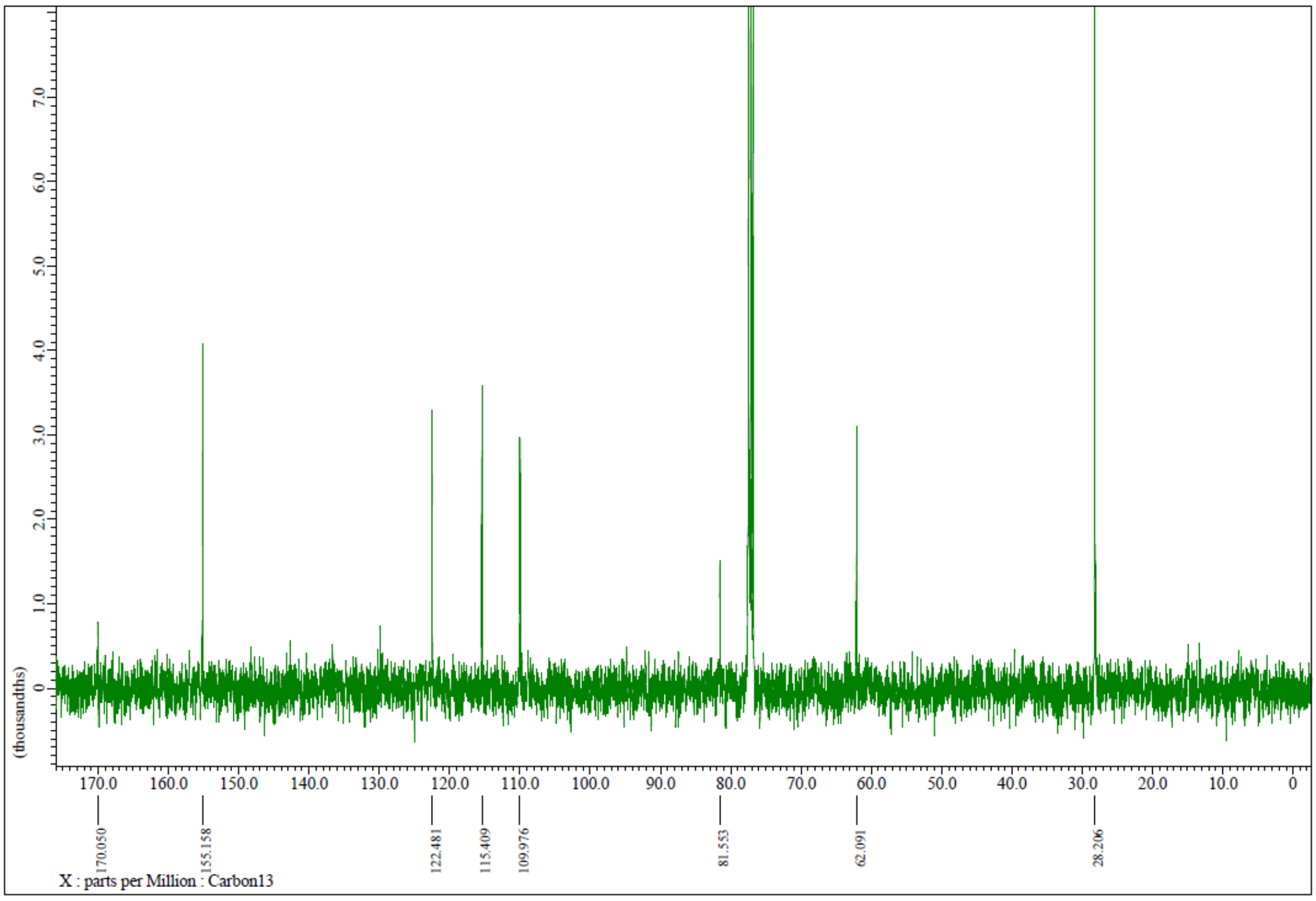

Fig. S29 ^{1}H-NMR and ^{13}C-NMR of 2d

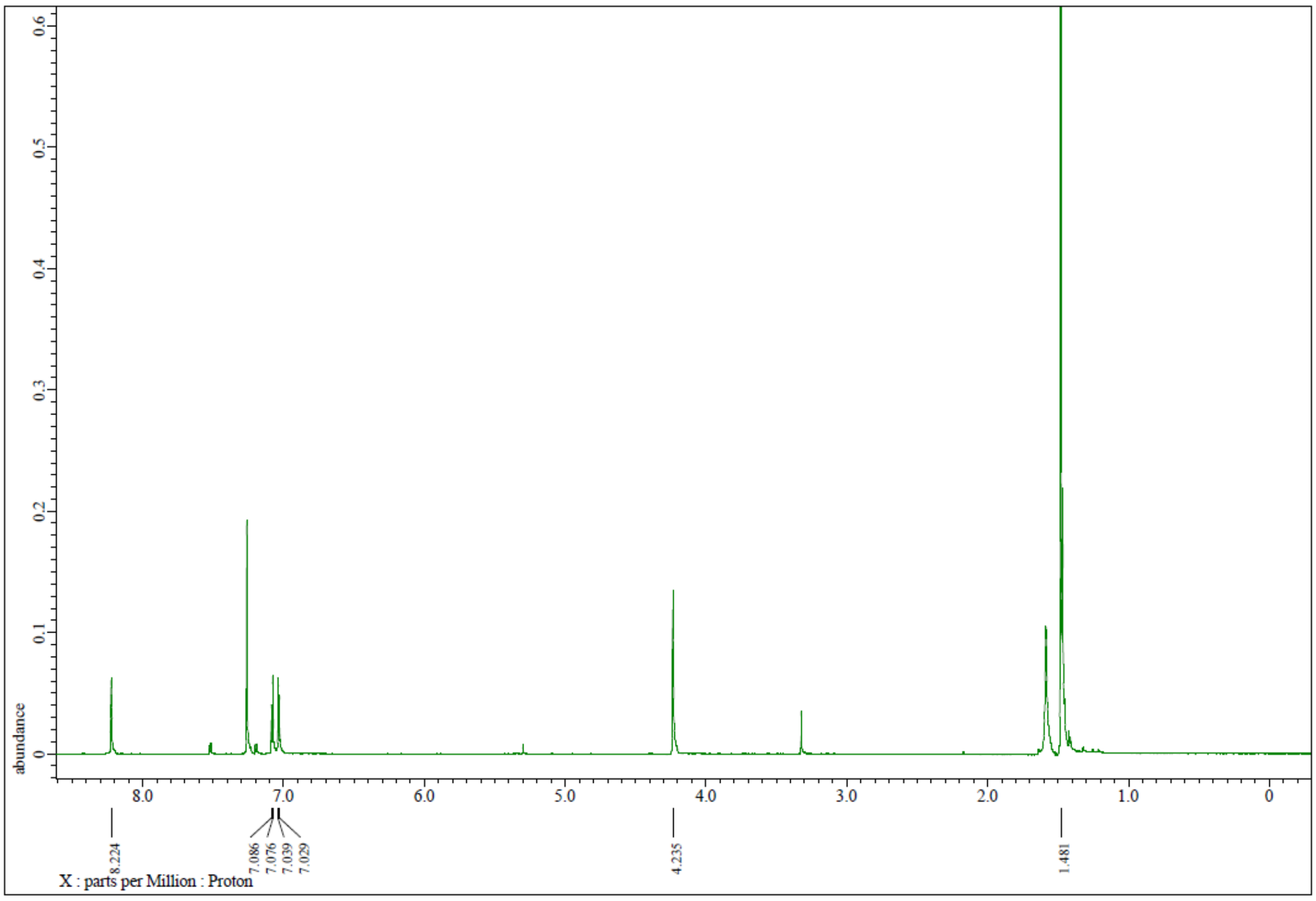

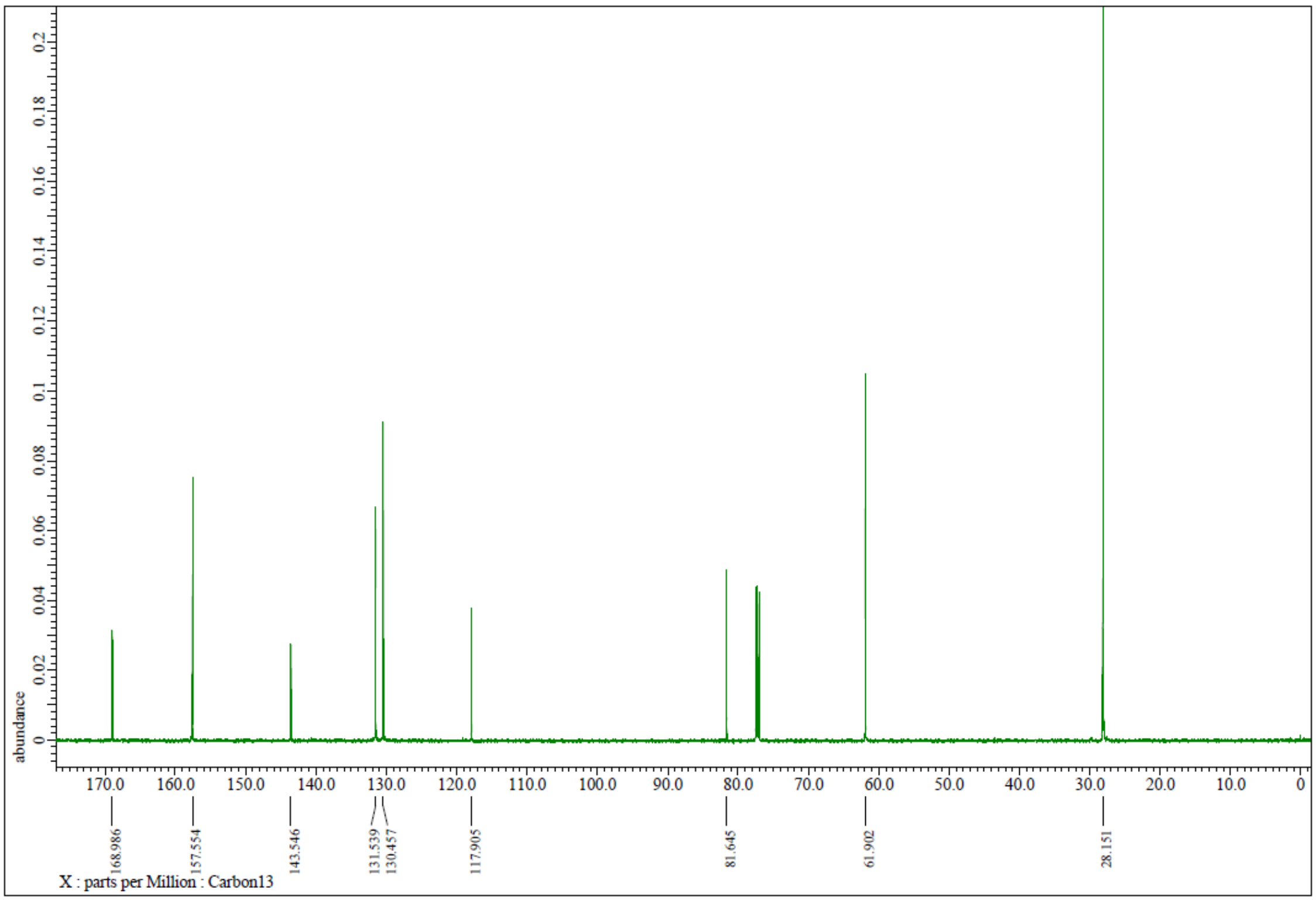

Fig. S30 ^{1}H-NMR and ^{13}C-NMR of 2e

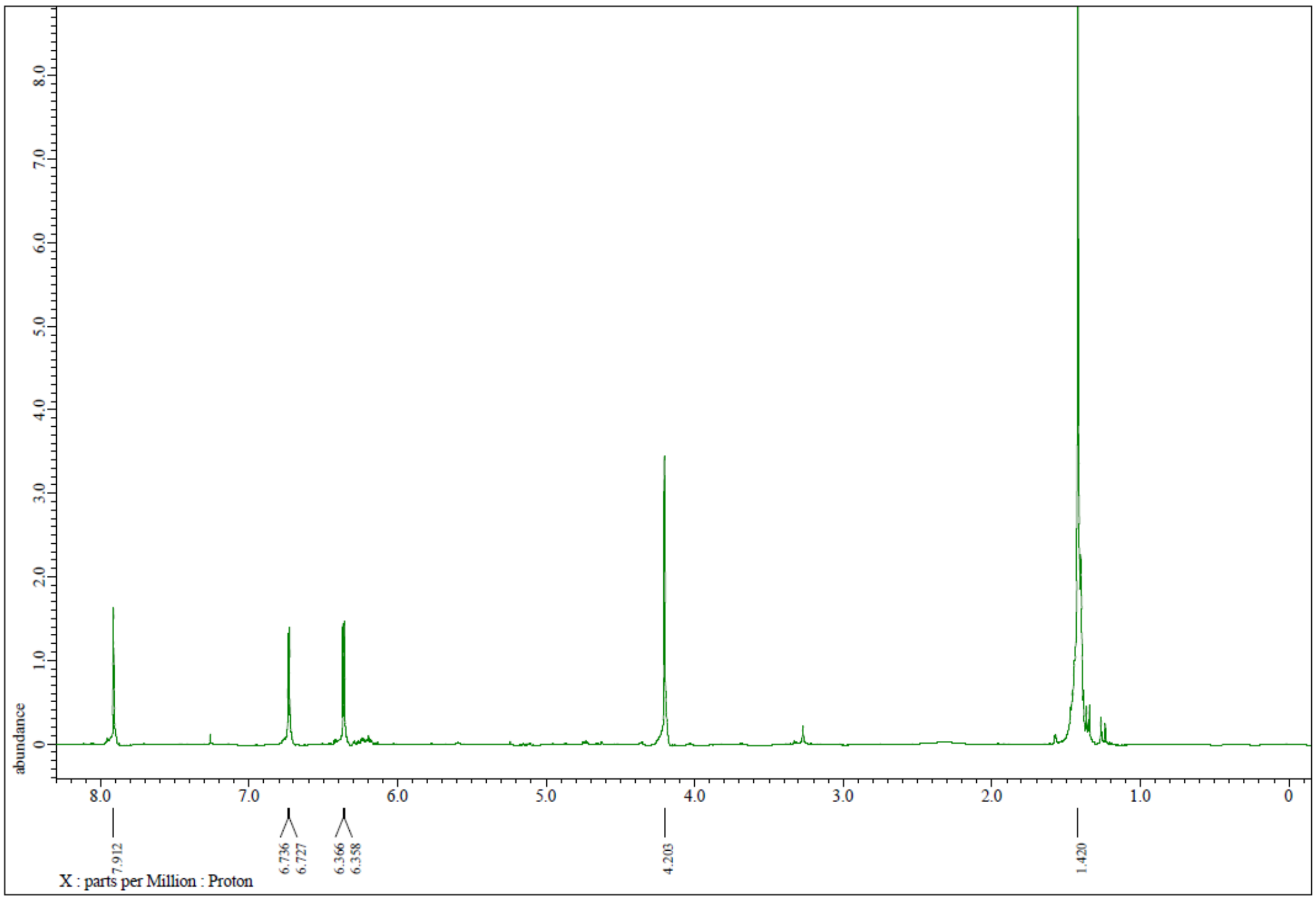

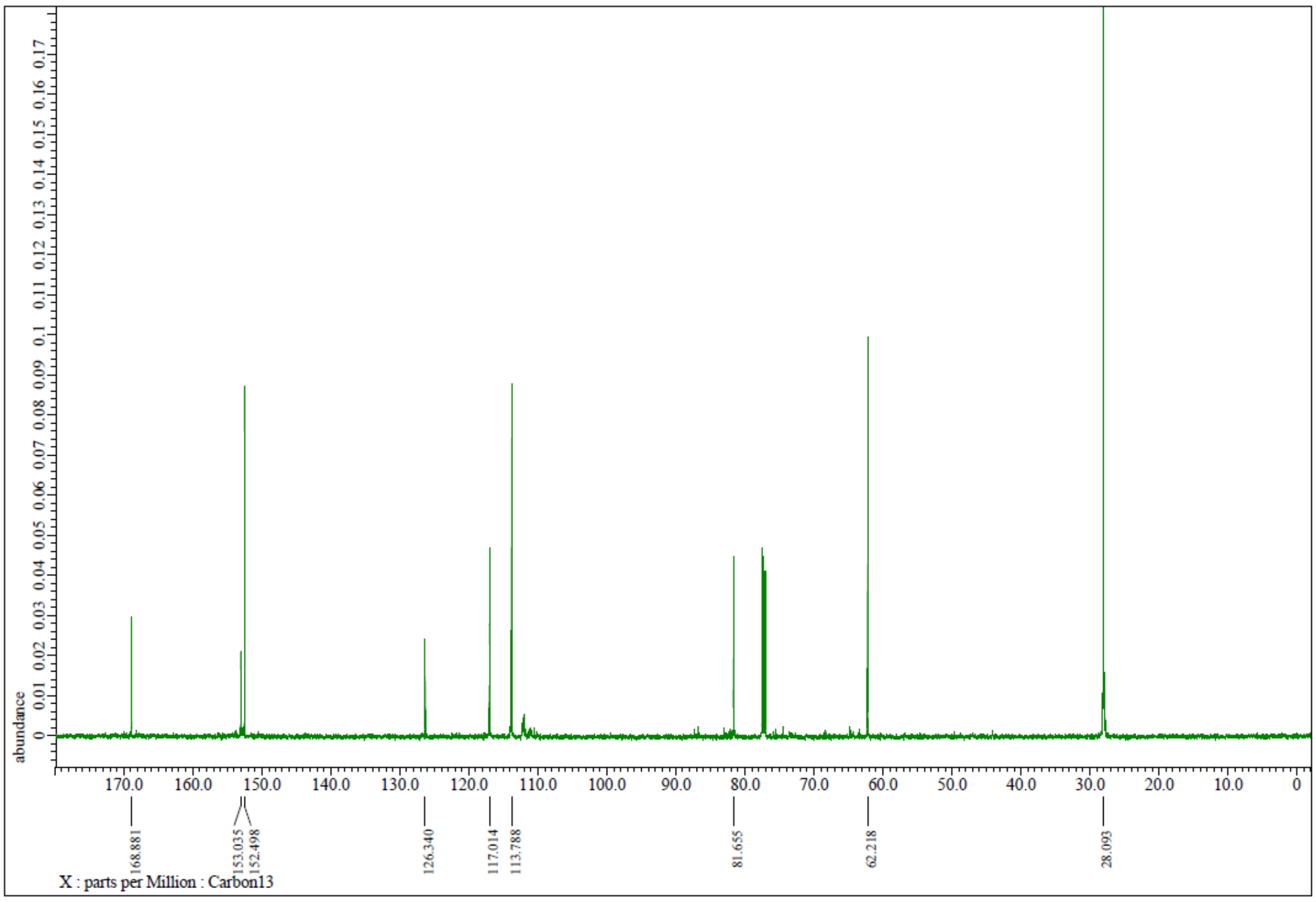

Fig. S31 ^{1}H-NMR and ^{13}C-NMR of 2f

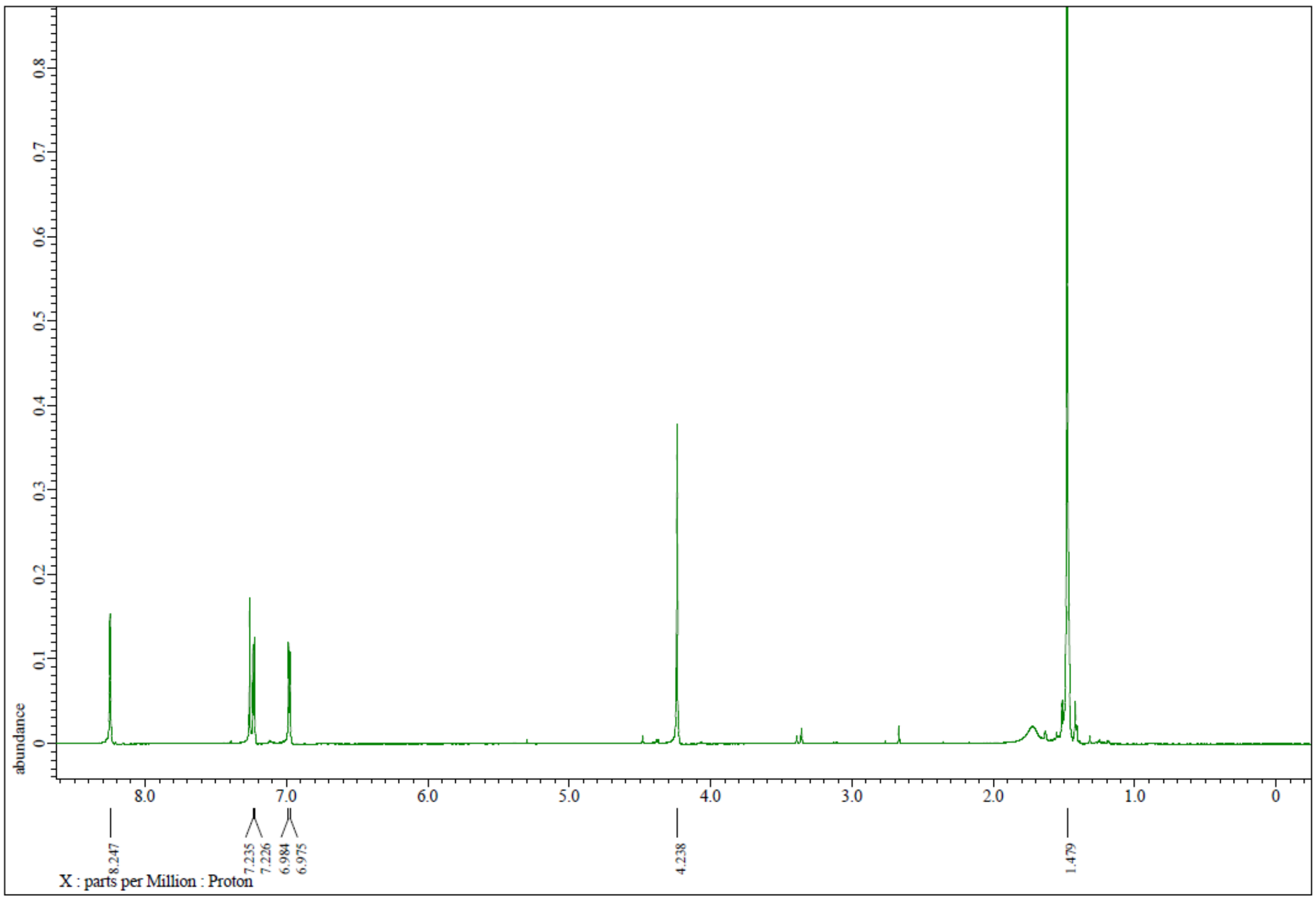

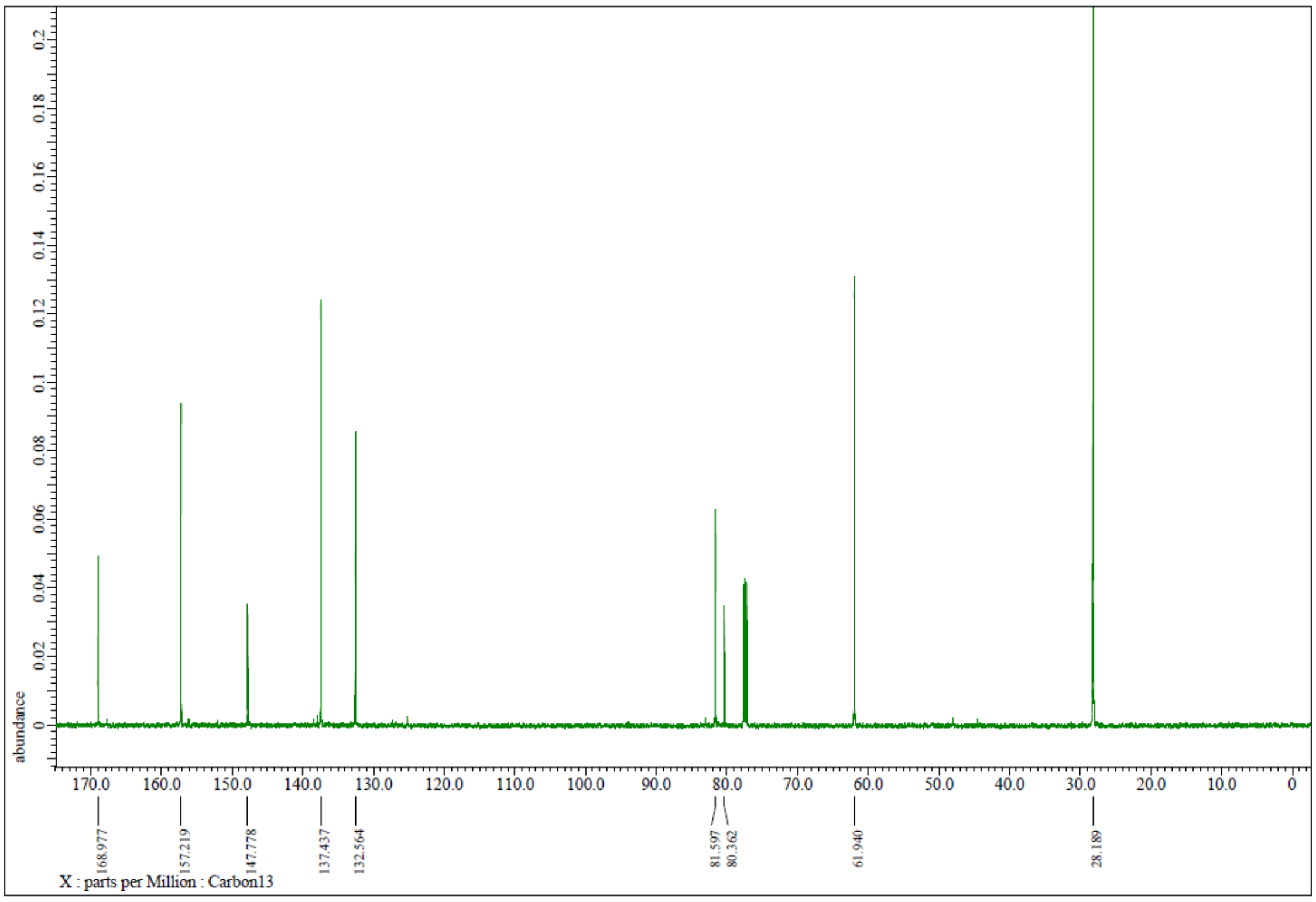

Fig. S32 ^{1}H-NMR and ^{13}C-NMR of 2g

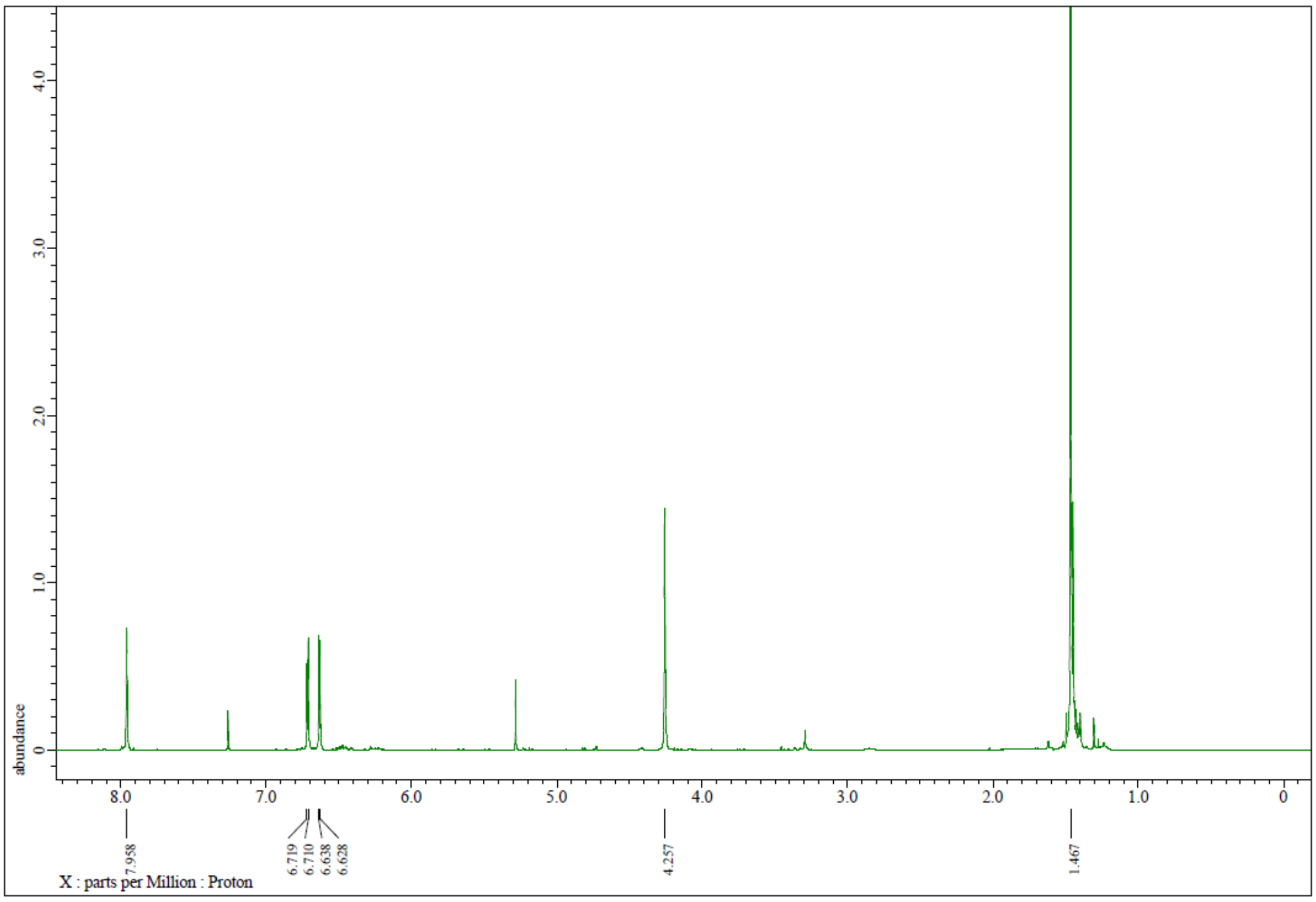

Fig. S33 ^{1}H-NMR of 2h

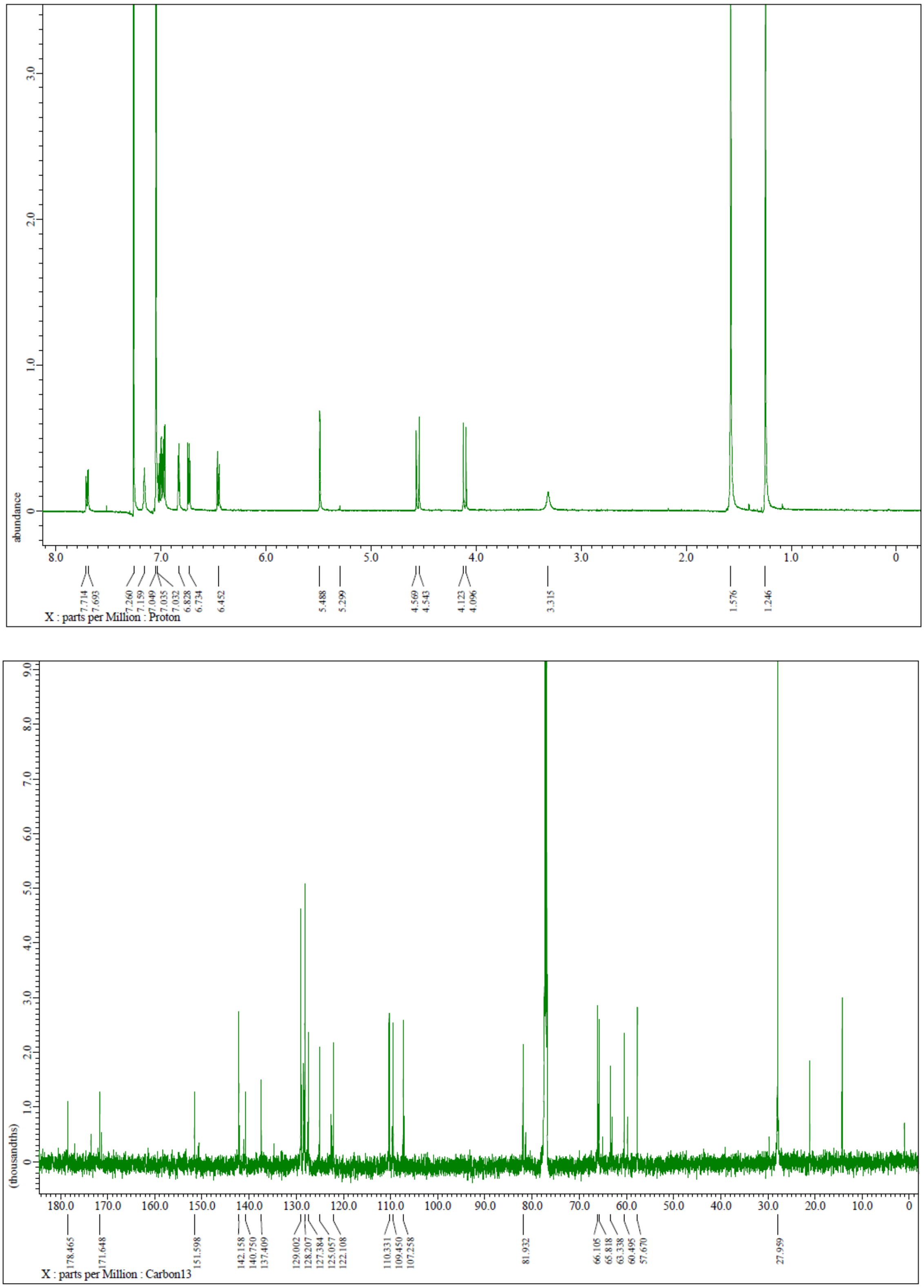

Fig. S34 ^{1}H-NMR and ^{13}C-NMR of 3a

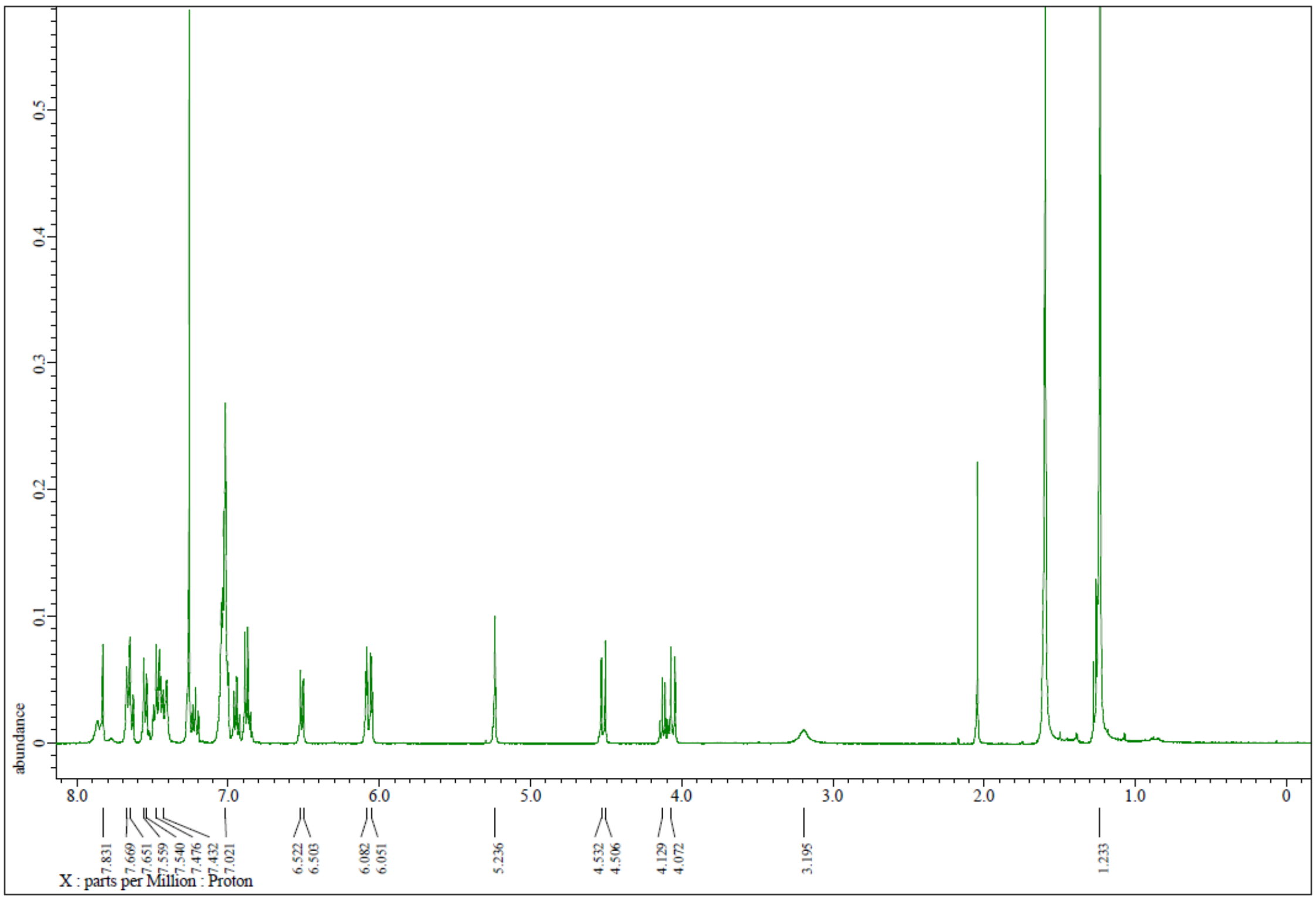

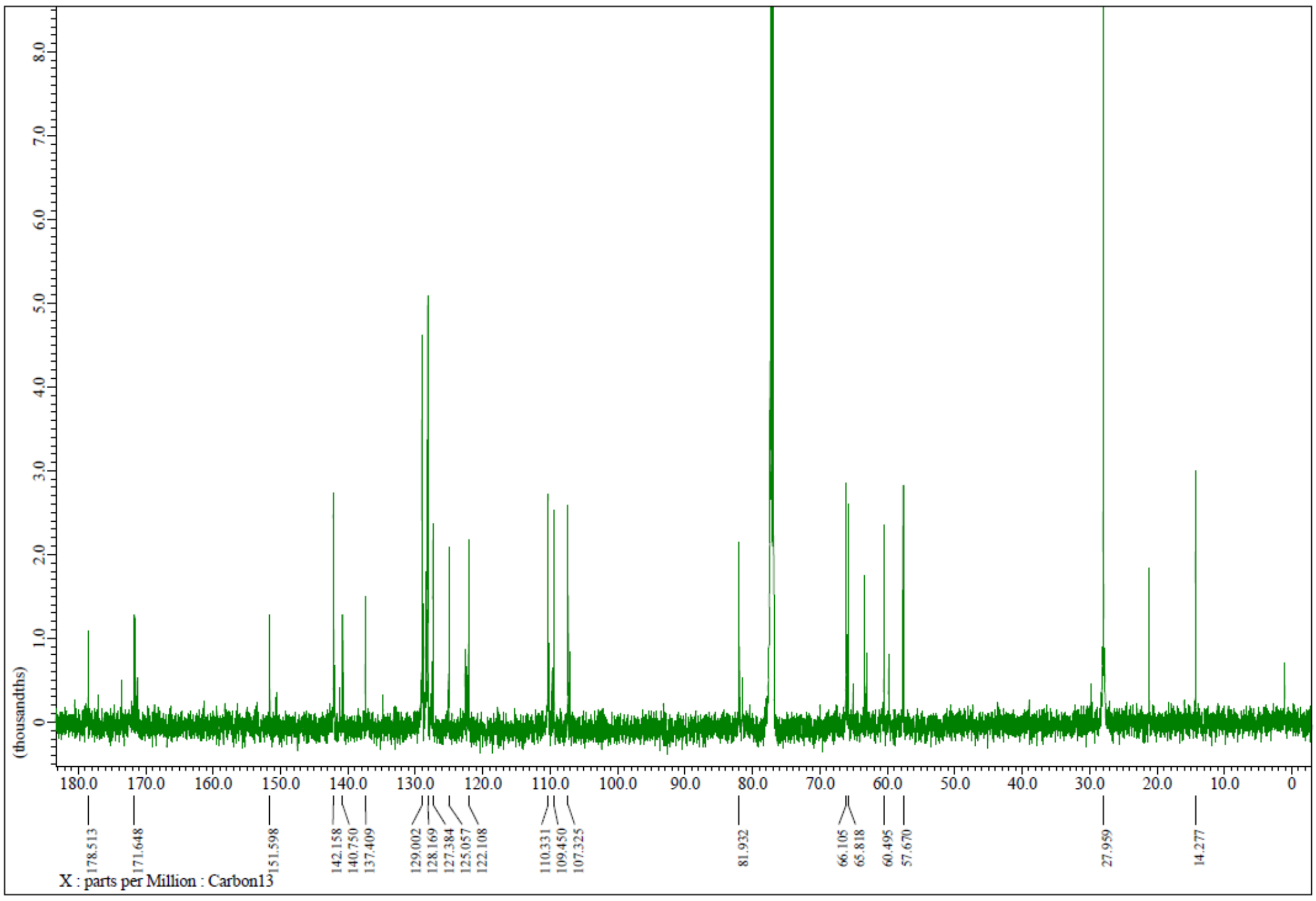

Fig. S35 ^{1}H-NMR and ^{13}C-NMR of 3b

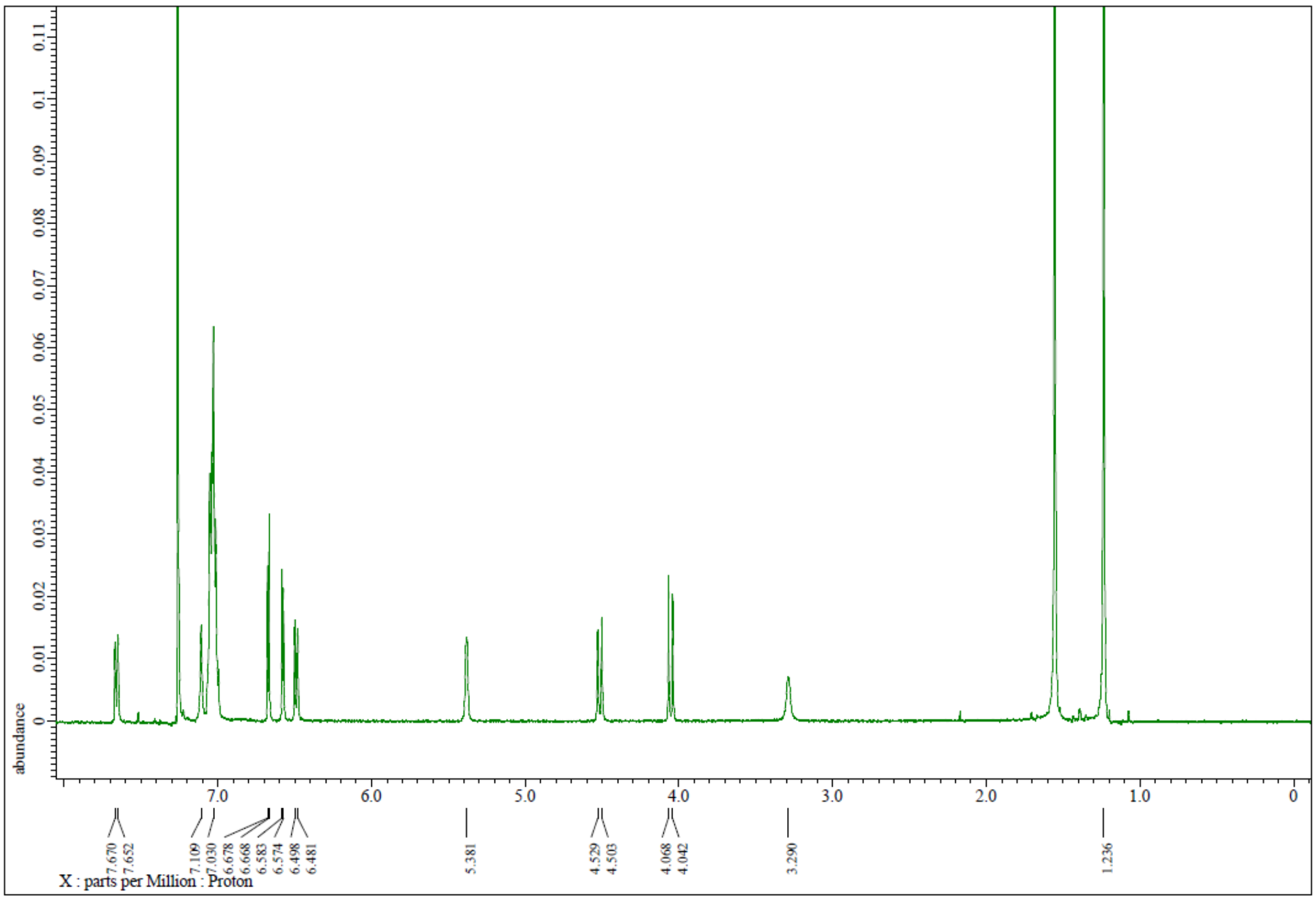

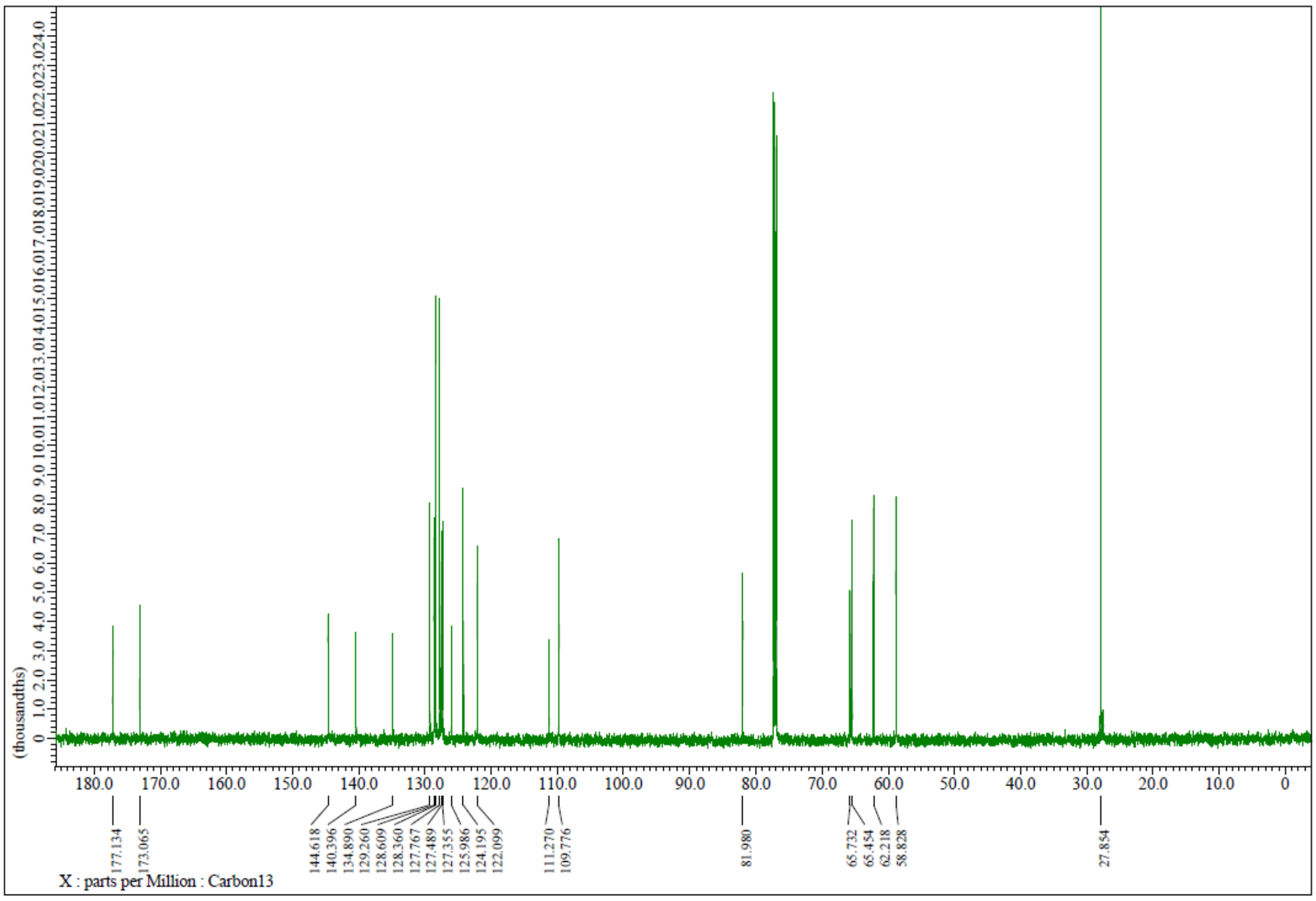

Fig. S36 ^{1}H-NMR and ^{13}C-NMR of 3c

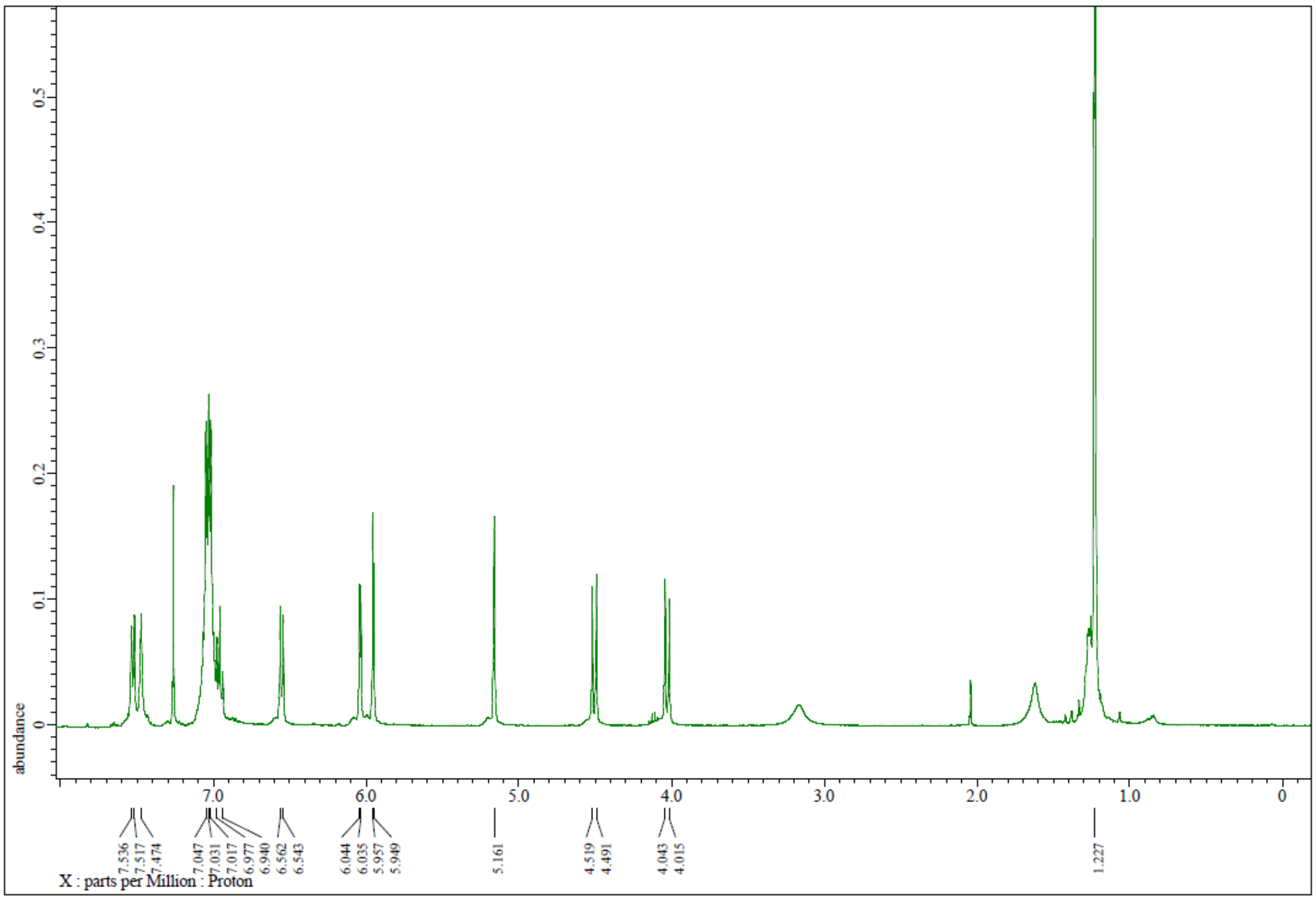

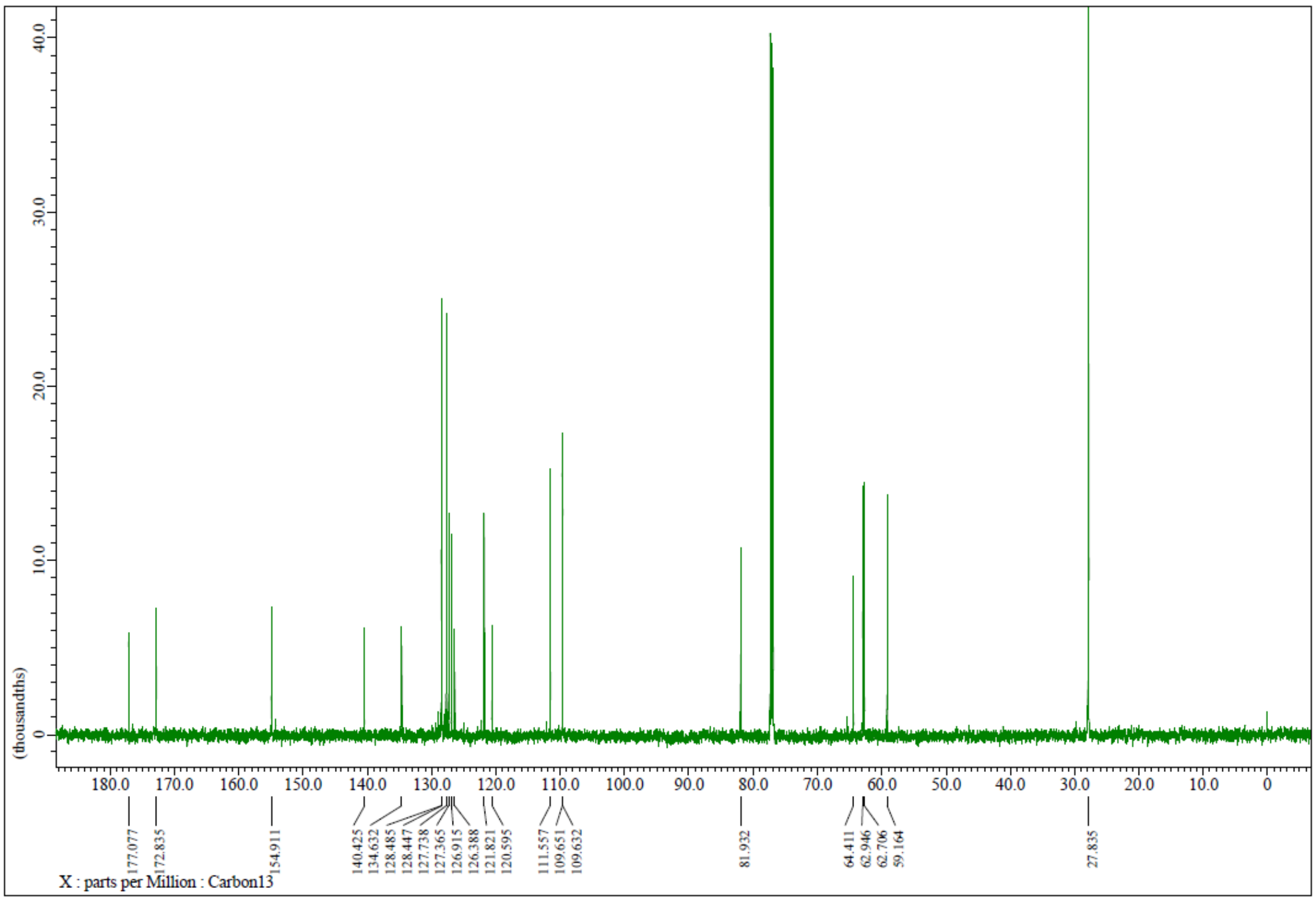

Fig. S37 ^{1}H-NMR and ^{13}C-NMR of 3d

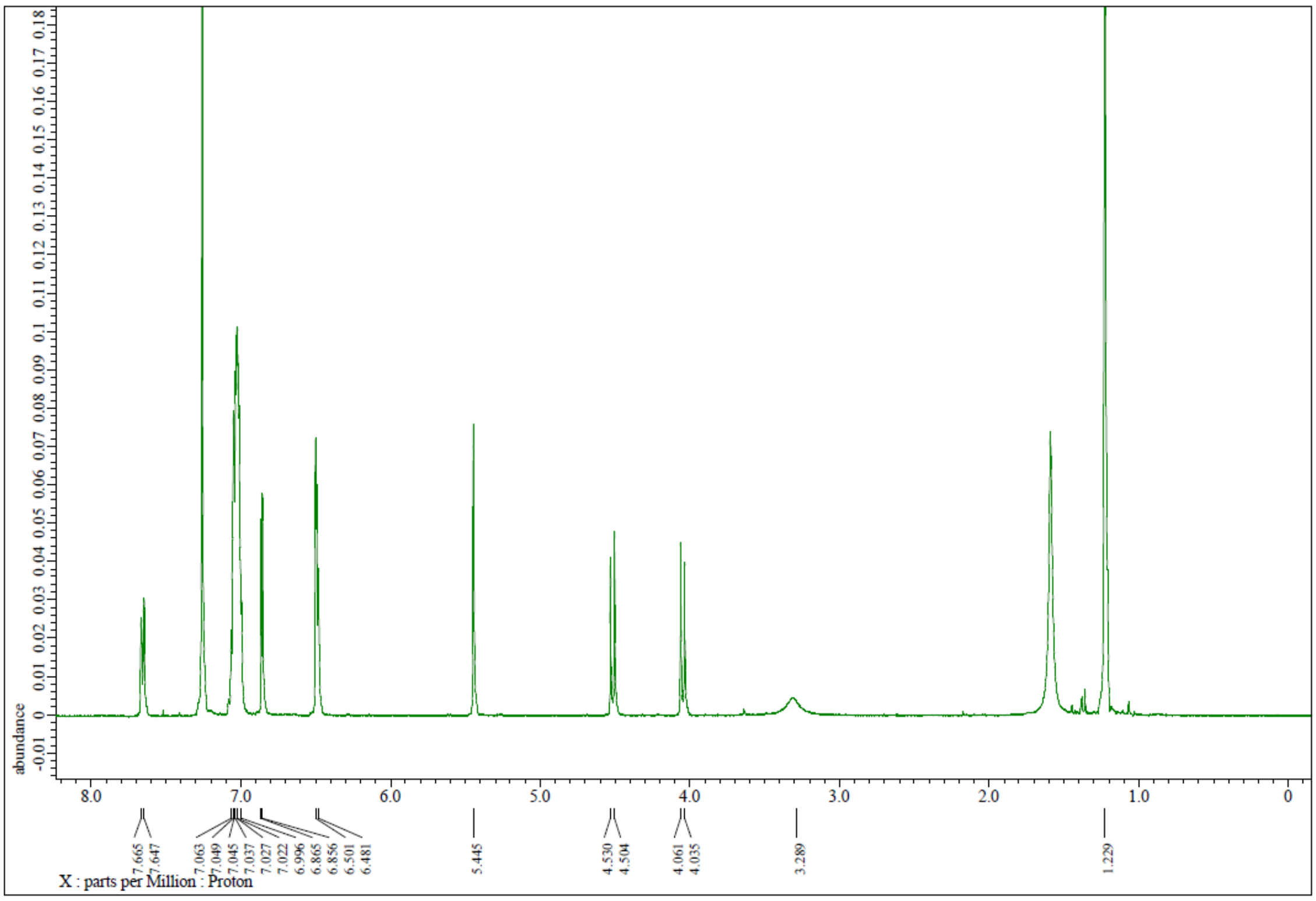

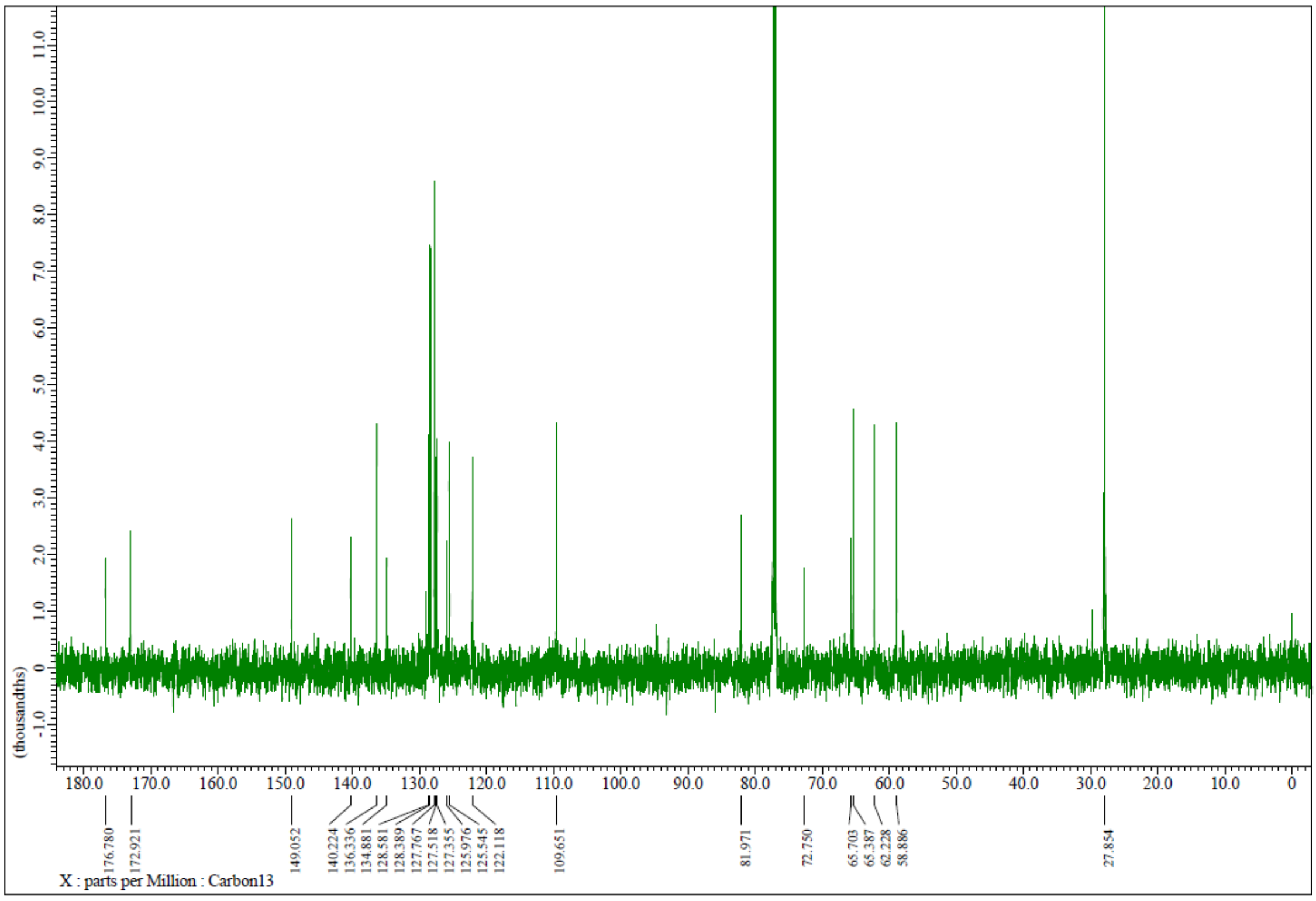

Fig. S38 ^{1}H-NMR and ^{13}C-NMR of 3e

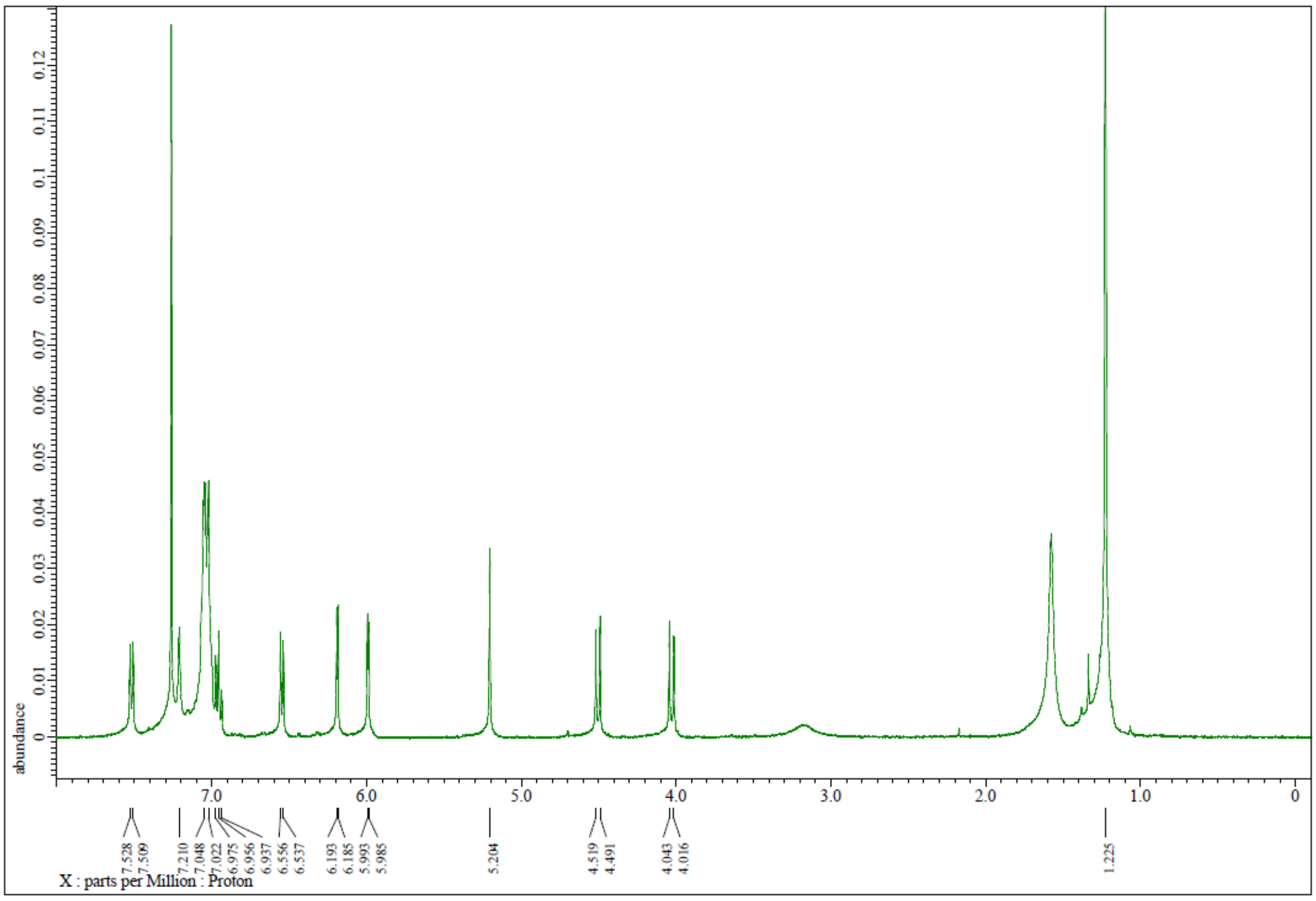

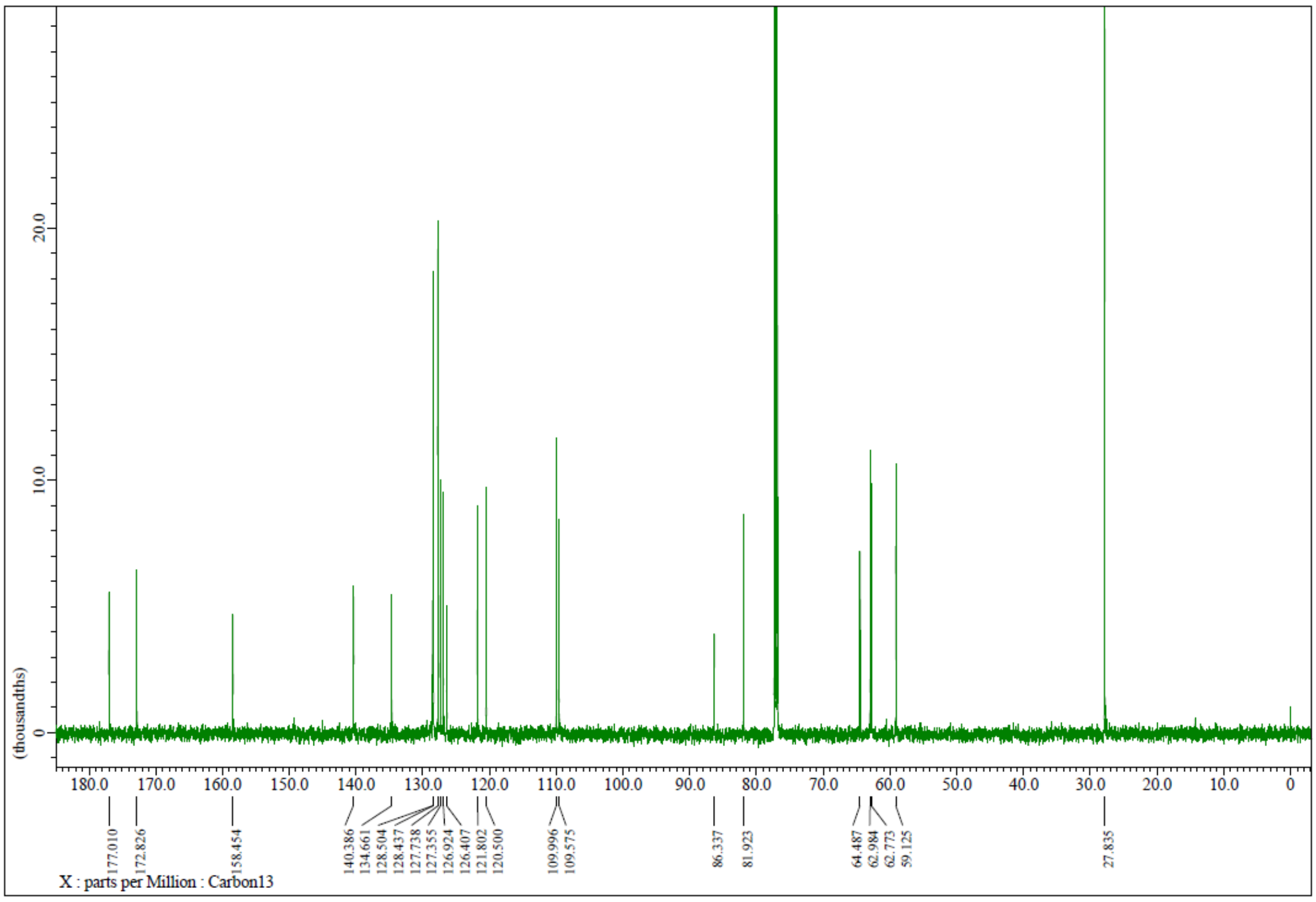

Fig. S39 ¹H-NMR and ¹³C-NMR of 3f

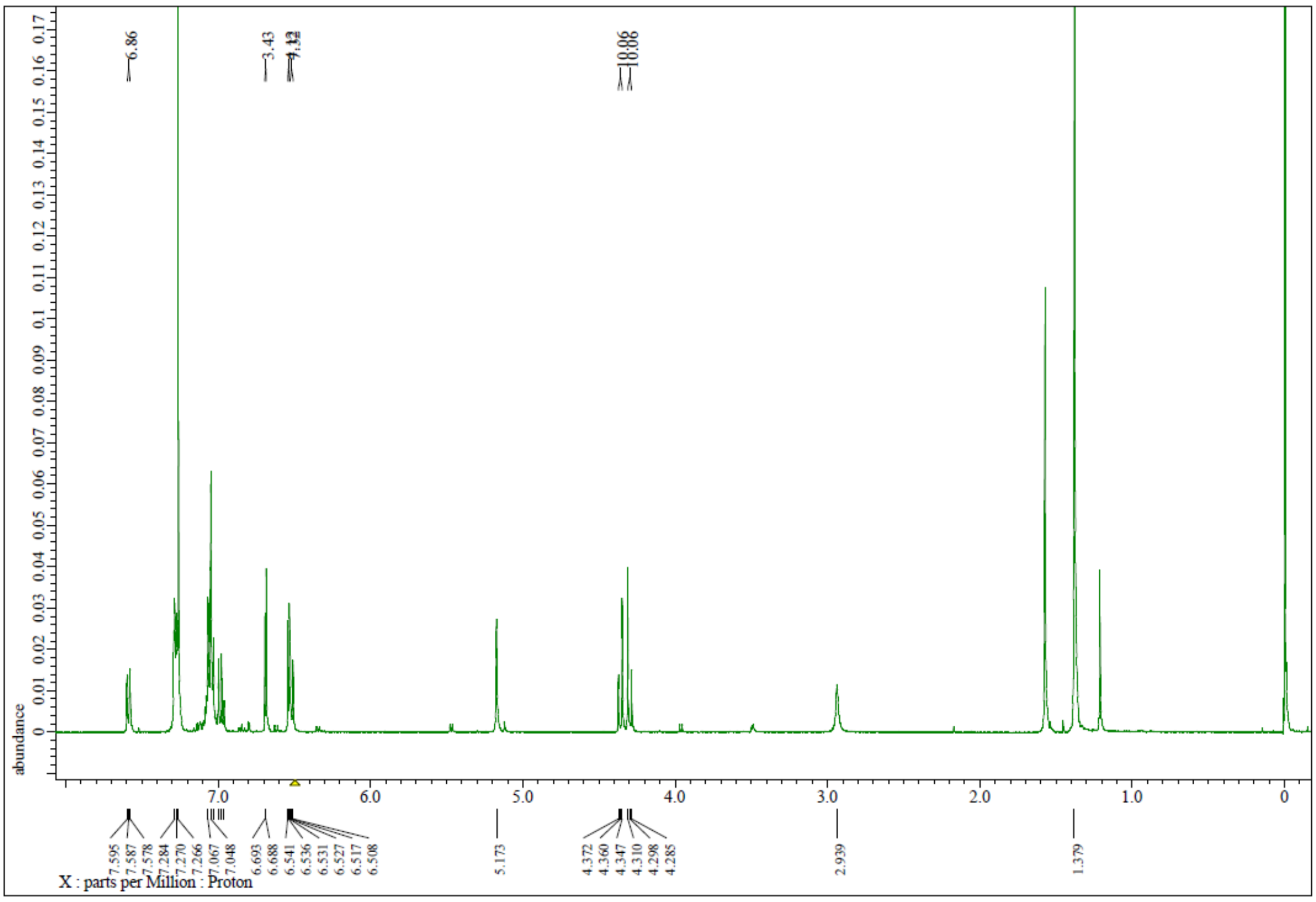

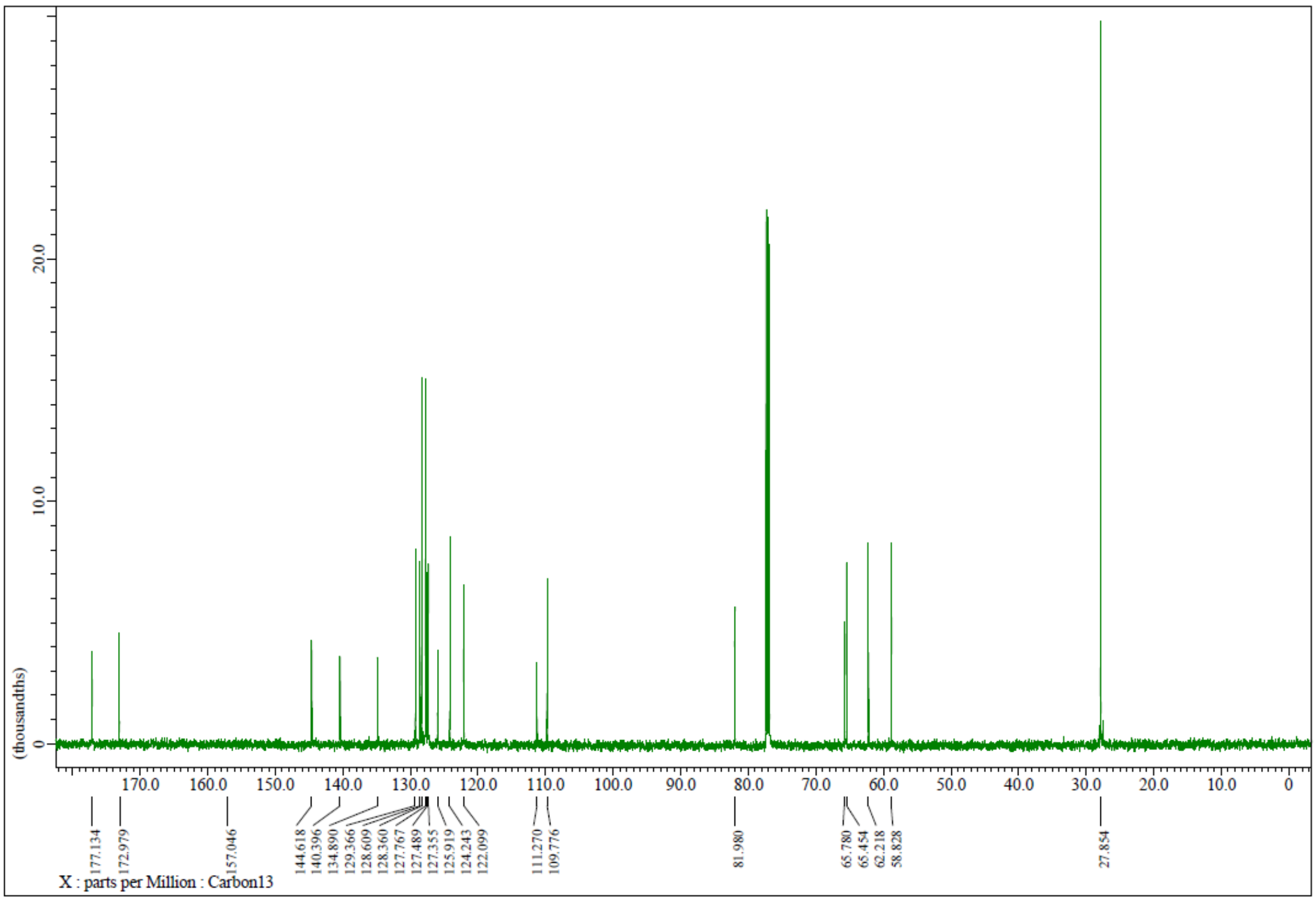

Fig. S40 ^{1}H-NMR and ^{13}C-NMR of 3i

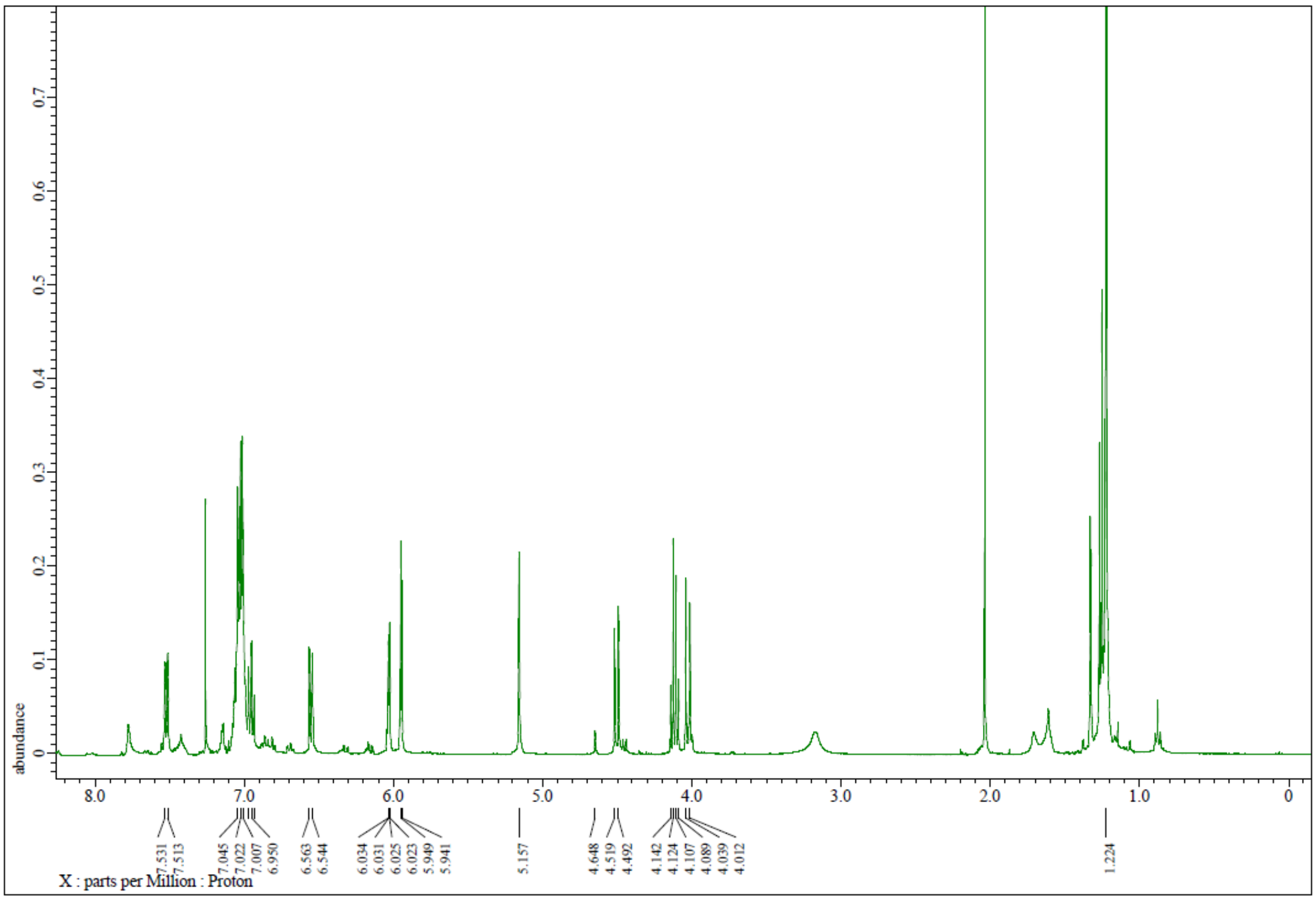

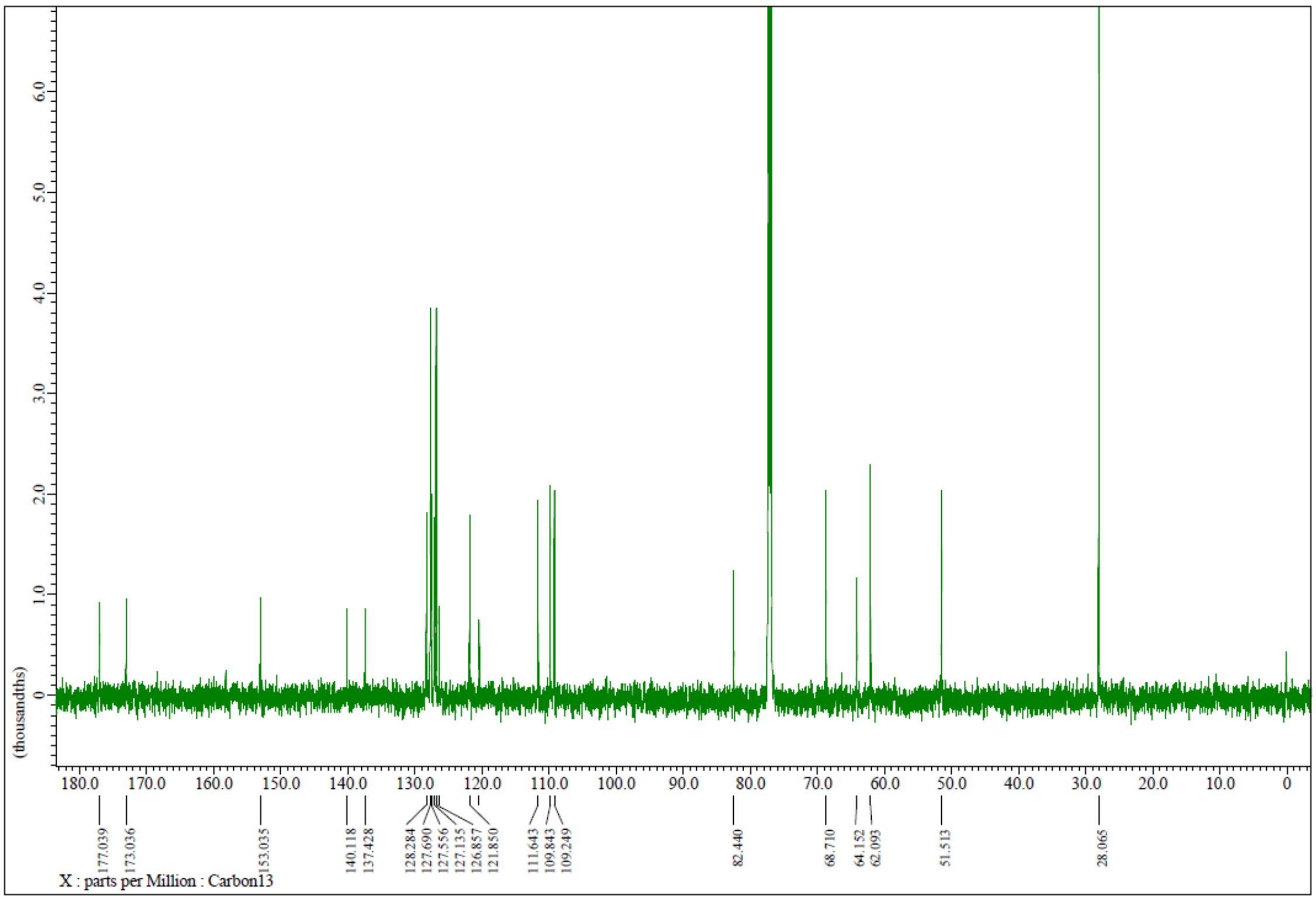

Fig. S41 ^{1}H-NMR and ^{13}C-NMR of 3j

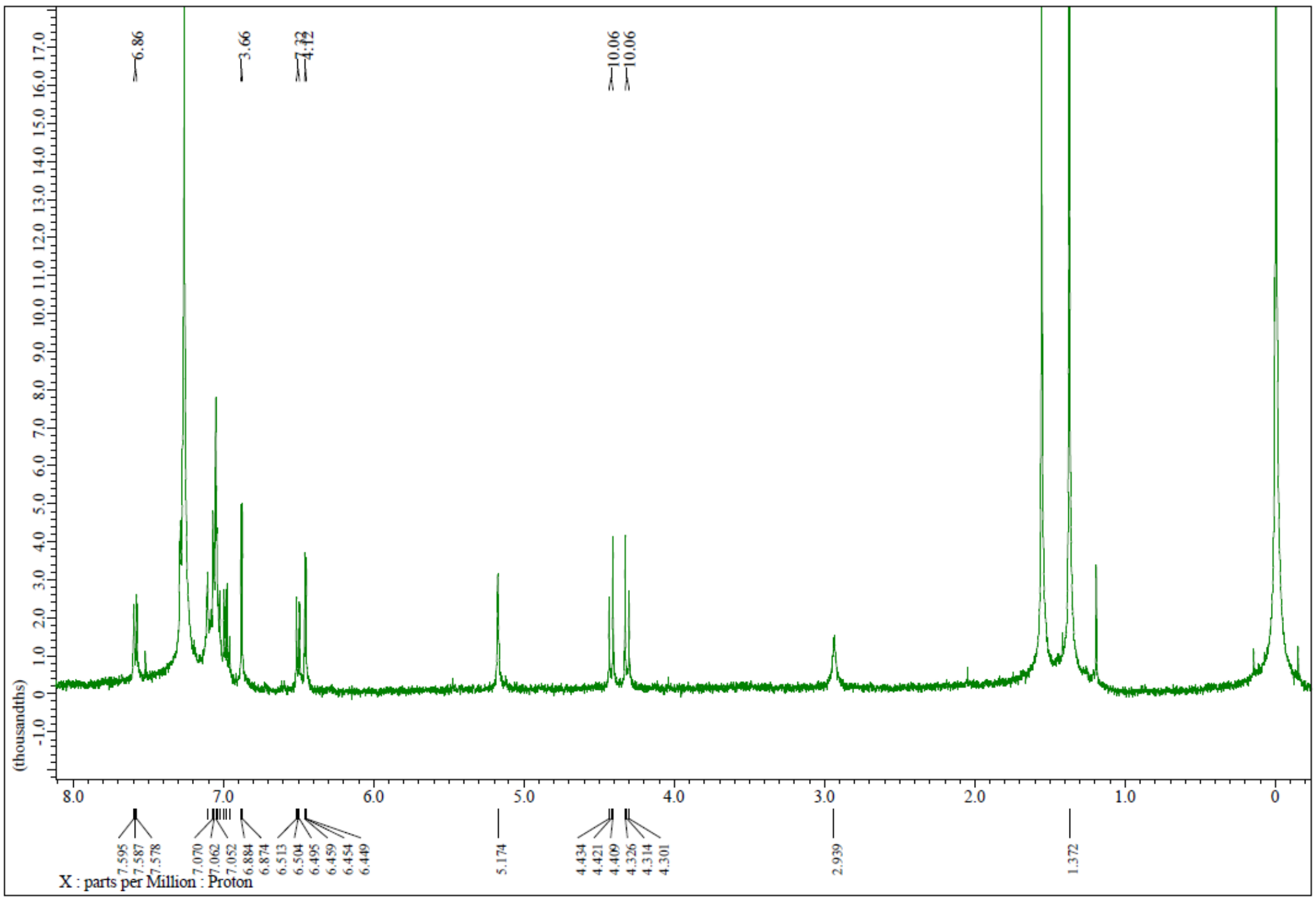

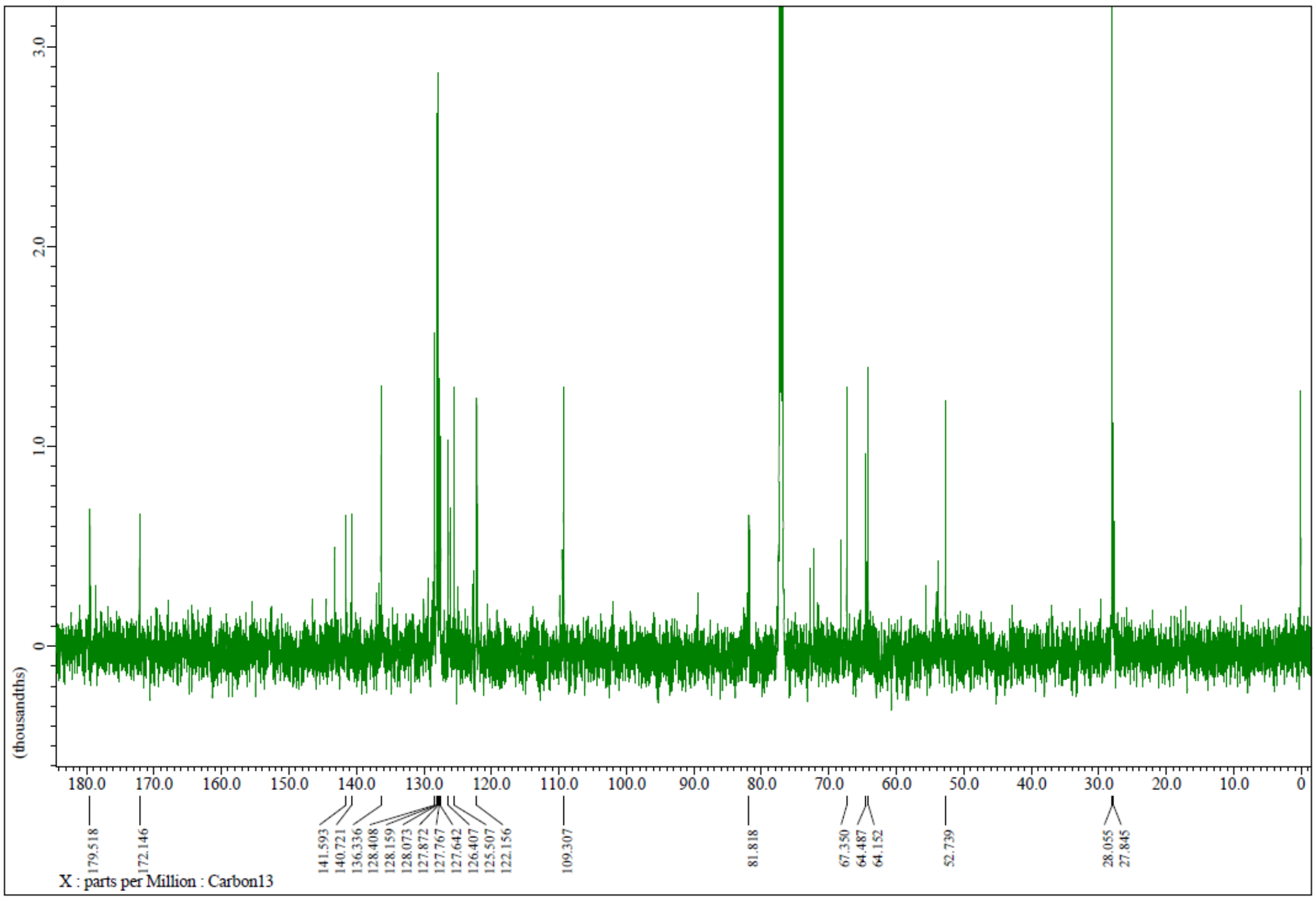

Fig. S42 ^{1}H-NMR and ^{13}C-NMR of 3k

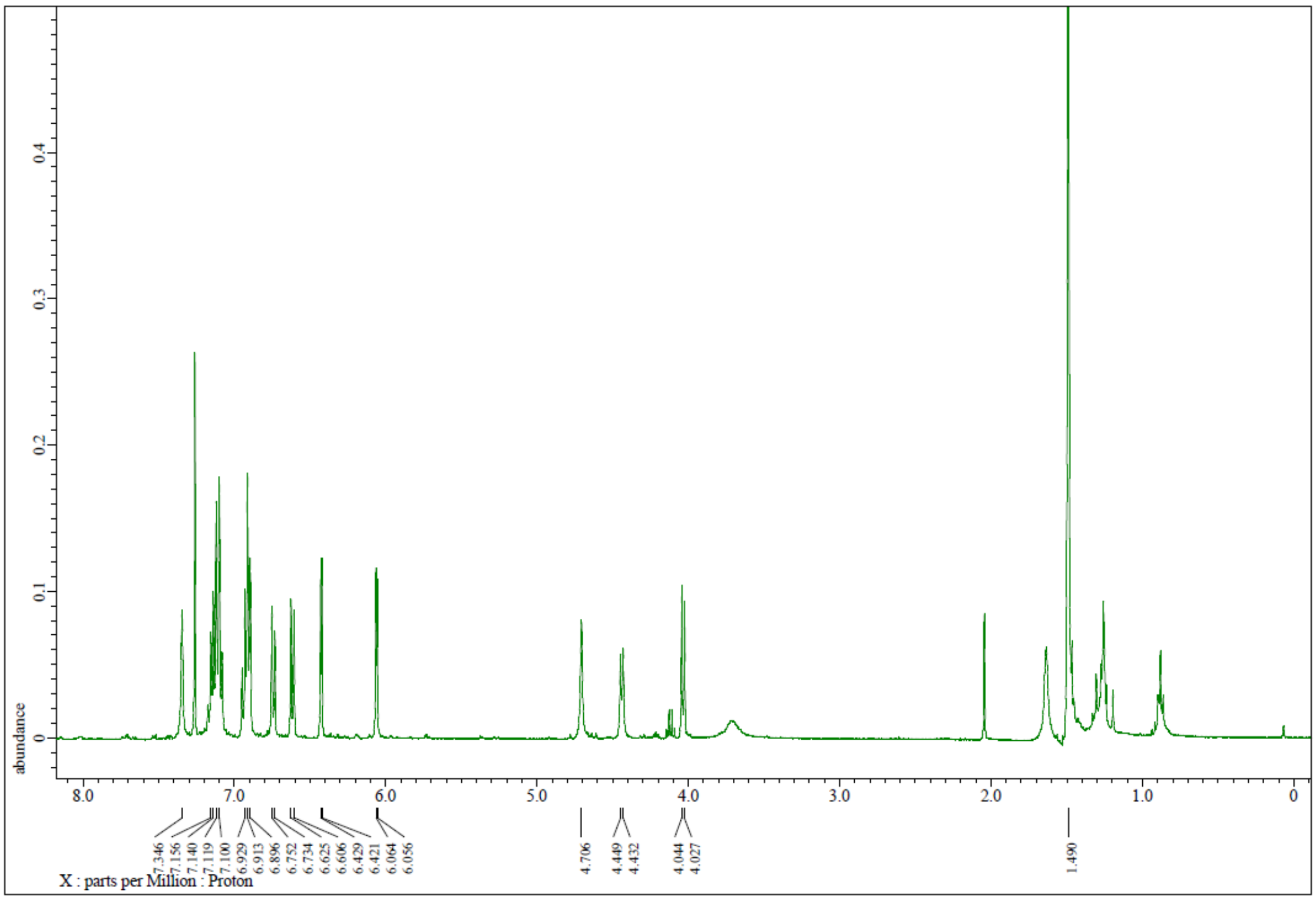

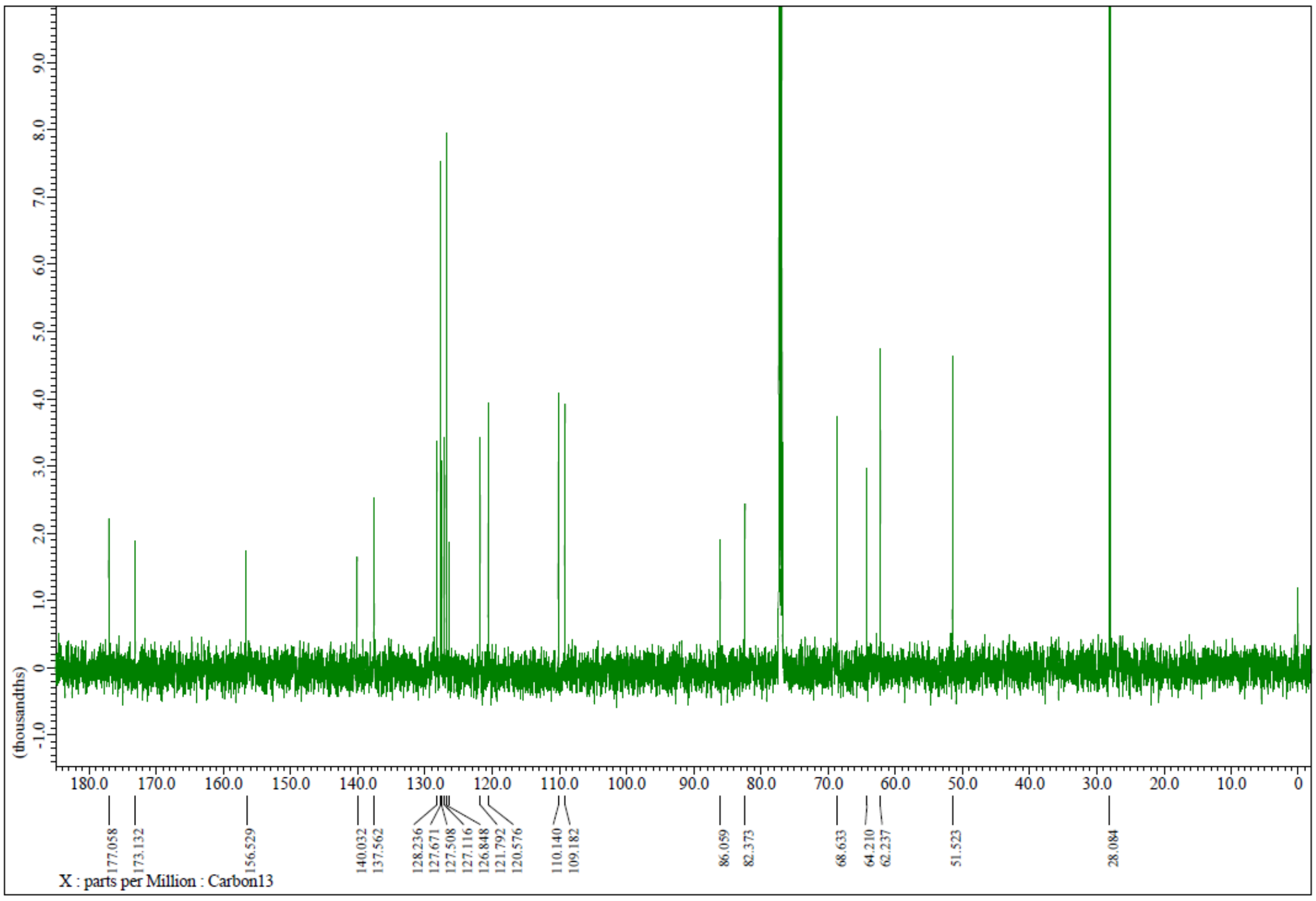

Fig. S43 ^{1}H-NMR and ^{13}C-NMR of 3l

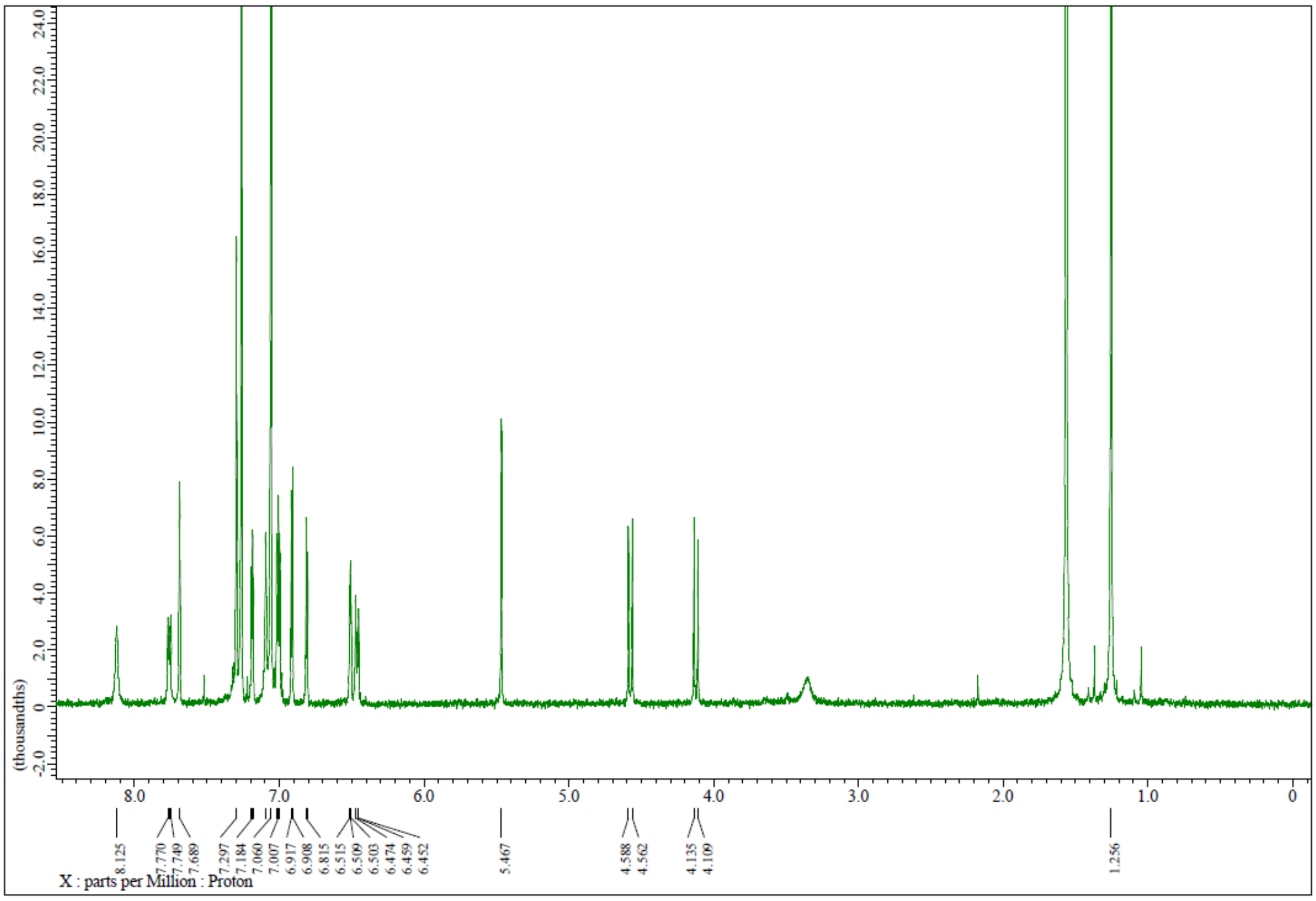

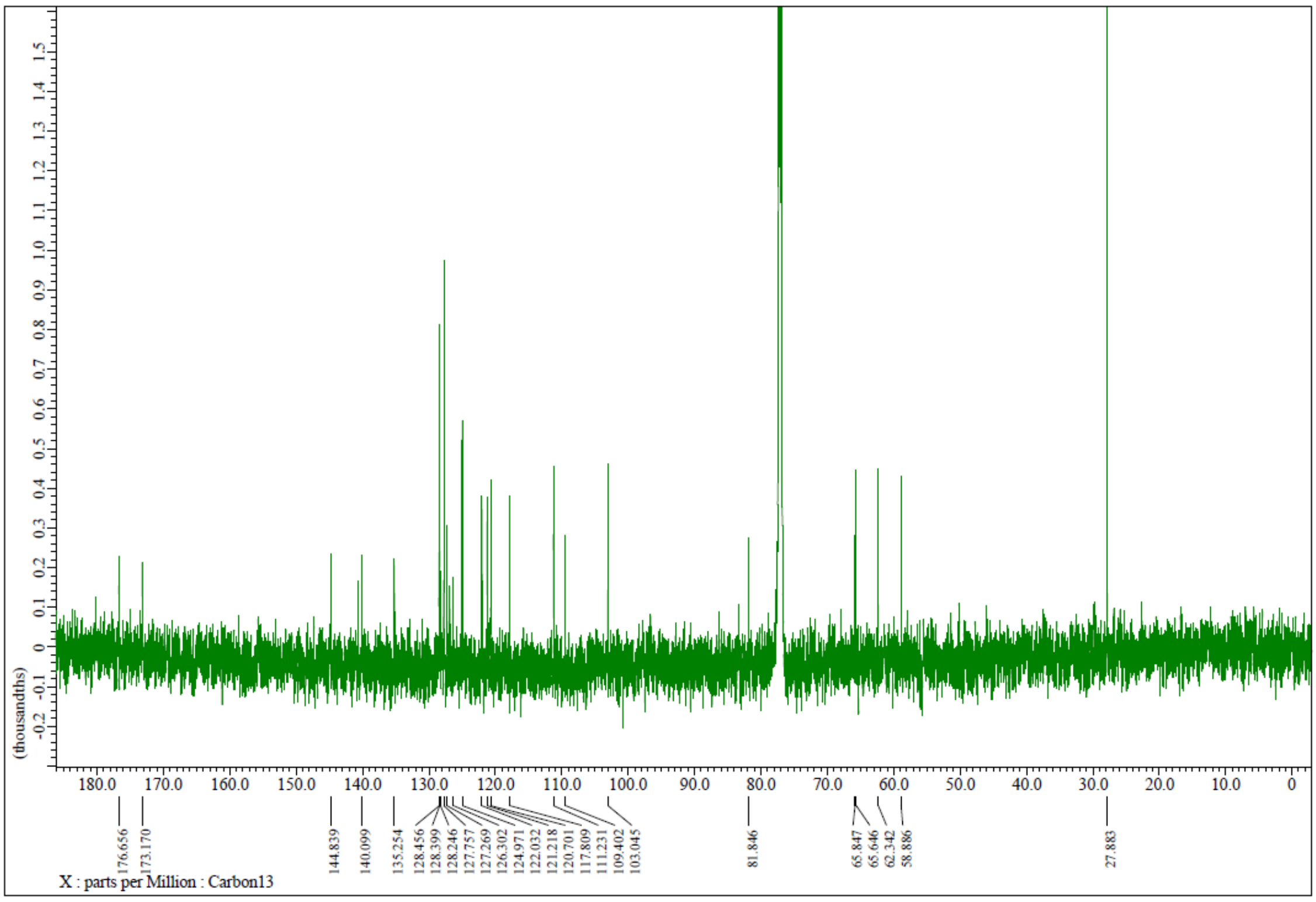

Fig. S44 ^{1}H-NMR and ^{13}C-NMR of 3g

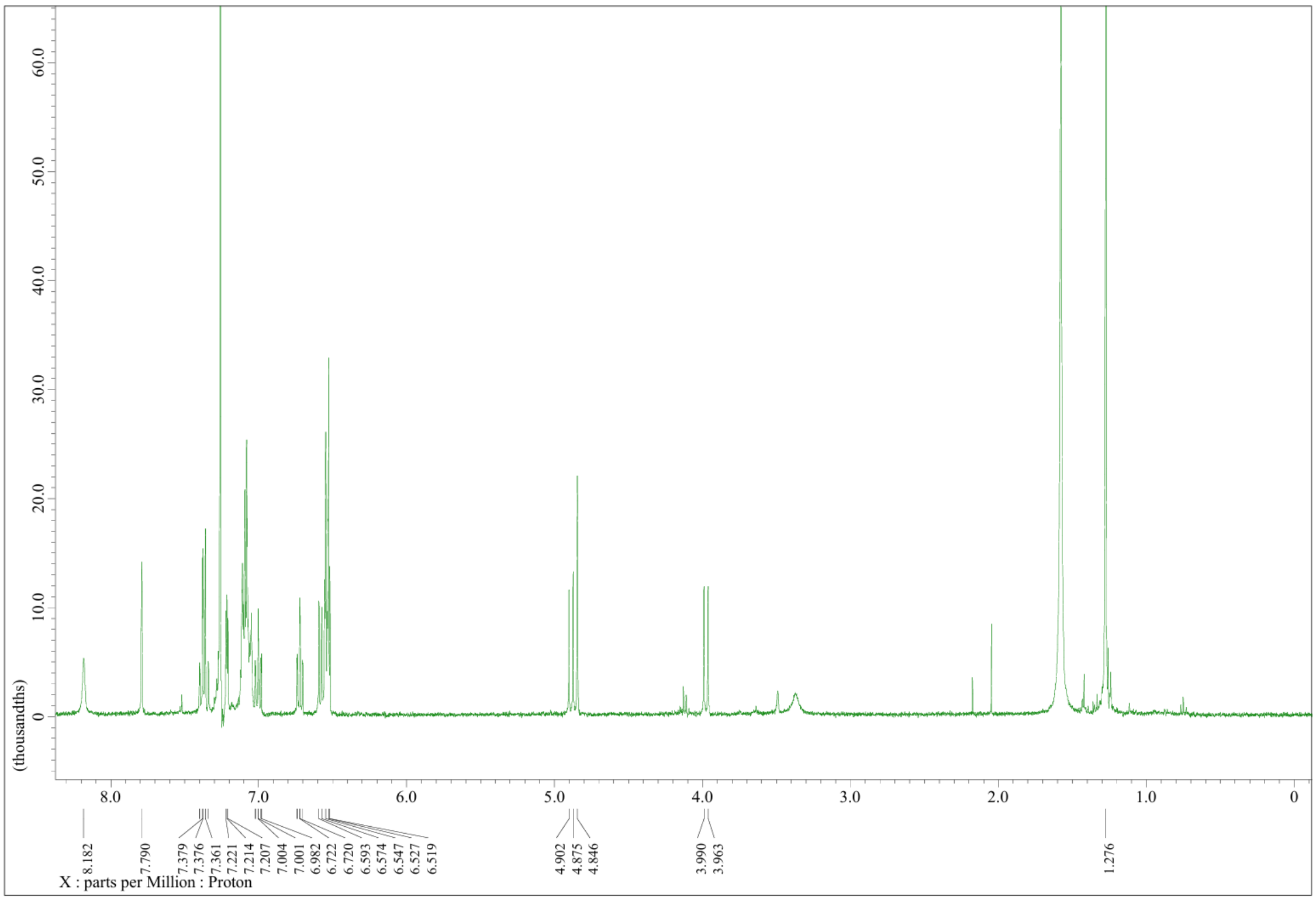

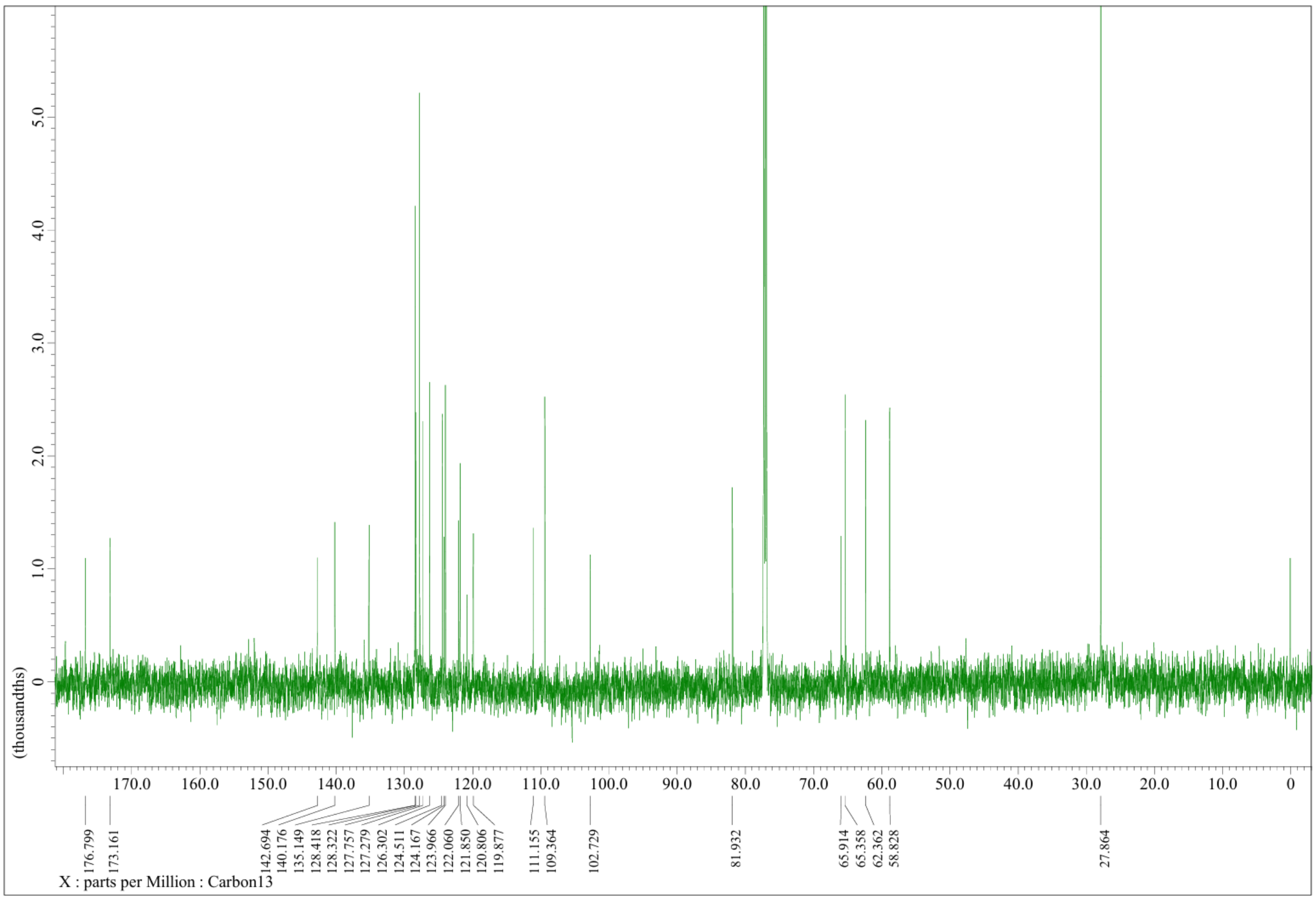

Fig. S45 ^{1}H-NMR and ^{13}C-NMR of 3h

Crystallographic data collection and refinement statistics.

Radiation type	CuKα
Measurement device type	Rigaku R-AXIS RAPID
Space group	P-1
Cell length a	8.6046
Cell length b	14.0825
Cell length c	14.3158
Cell angle alpha	76.91
Cell angle beta	76.91
Cell angle gamma	74.9688
Cell volume	1438.97
Temperature (K)	93
Measured fraction theta max	0.977
Measured fraction theta full	0.977
Total reflections	5143
Unique reflections	2391
R factor all	0.1742
wR factor ref	0.2791
